# Supplementary material for: Genetic control of DNA methylation is largely shared across European and East Asian populations
Source: Nat Commun. 2024 Mar 28;15:2713. doi: 10.1038/s41467-024-47005-0 (PMC10978881; doi:10.1038/s41467-024-47005-0)

Supplementary Material

Genetic control of DNA methylation is largely shared across European and East Asian populations
Hatton et al

Table of Contents

***Supplementary Note 1: Systems Genomics of Parkinson's Disease Collaborators*...............2**
***Supplementary Note 2: Cohort acknowledgements*...3**
Brisbane Systems Genomics Study (BSGS)... 3
Lothian Birth Cohorts of 1921 and 1936 (LBC) 3
Systems Genomics of Parkinson's Disease (SGPD)... 3
Chinese Motor Neuron Disease Cohort (CHNMND) 3
Tibetan Han Chinese high-altitude (THCH) 4
***Supplementary Tables*..5**
***Supplementary Figures* 8**

Supplementary Note 1: Systems Genomics of Parkinson's Disease Collaborators

Tim Anderson (New Zealand Brain Research Institute, Christchurch, New Zealand; Department of Medicine, University of Otago, Christchurch, New Zealand), Steven Bentley (Griffith Institute for Drug Discovery, Griffith University, Brisbane, Australia), John Dalrymple-Alford (Dept. Psychology, University of Canterbury, New Zealand Brain Research Institute), Javed Fowdar (Griffith Institute for Drug Discovery, Griffith University, Brisbane, Australia), Jacob Gratten (Mater Research Institute, University of Queensland, Brisbane, Australia), Glenda Halliday (Brain and Mind Centre, Sydney Medical School, University of Sydney, Sydney, Australia), Anjali K. Henders (Institute for Molecular Bioscience, University of Queensland, Brisbane, Australia), Ian Hickie (Brain and Mind Centre, Sydney Medical School, University of Sydney, Sydney, Australia), Irfahan Kassam (Institute for Molecular Bioscience, University of Queensland, Brisbane, Australia), Martin Kennedy (Department of Pathology, University of Otago, Christchurch, New Zealand), John Kwok (Brain and Mind Centre, Sydney Medical School, University of Sydney, Sydney, Australia), Simon Lewis (Brain and Mind Centre, Sydney Medical School, University of Sydney, Sydney, Australia), George Mellick (Griffith Institute for Drug Discovery, Griffith University, Brisbane, Australia), Grant Montgomery (Institute for Molecular Bioscience, University of Queensland, Brisbane, Australia), John Pearson (Department of Pathology, University of Otago, Christchurch, New Zealand), Toni Pitcher (New Zealand Brain Research Institute, Christchurch, New Zealand; Department of Medicine, University of Otago, Christchurch, New Zealand), Julia Sidorenko (Institute for Molecular Bioscience, University of Queensland, Brisbane, Australia), Peter A. Silburn (Queensland Brain Institute, University of Queensland, Brisbane, Australia), Costanza L. Vallerga (Laboratory of Population Genomics, Department of Internal Medicine, Erasmus MC, Rotterdam, The Netherlands), Peter M. Visscher (Institute for Molecular Bioscience, University of Queensland, Brisbane, Australia; Queensland Brain Institute, University of Queensland, Brisbane, Australia), Leanne Wallace, (Institute for Molecular Bioscience, University of Queensland, Brisbane, Australia), Naomi R. Wray (Institute for Molecular Bioscience, University of Queensland, Brisbane, Australia; Queensland Brain Institute, University of Queensland, Brisbane, Australia), Angli Xue (Garvan-Weizmann Centre for Cellular Genomics, Garvan Institute of Medical Research, Sydney, Australia; School of Biomedical Sciences, University of New South Wales, Sydney, Australia), Jian Yang (Institute for Molecular Bioscience, University of Queensland, Brisbane, Australia; Queensland Brain Institute, University of Queensland, Brisbane, Australia), Futao Zhang (Institute for Molecular Bioscience, University of Queensland, Brisbane, Australia)

Supplementary Note 2: Cohort acknowledgements

Brisbane Systems Genomics Study (BSGS)

The study samples were collected in the context of the BSGS within the Brisbane Longitudinal Twin Study 1992-2016. This work was supported by the Australian National Health and Medical Research Council (NHMRC) (project grant 1087407, 1031119, 1010374, 496667 and 1046880), the National Institutes of Health (NIH) (grants GM057091 and GM099568), Australian Research Council (A7960034, A79906588, A79801419, DP0212016, DP0343921, DP1093900) and NHMRC Medical Bioinformatics Genomics Proteomics Program (grant 389891) for building and maintaining the adolescent twin family resource, through which samples were collected. We gratefully acknowledge the participation of the twins and their families. We thank Marlene Grace, Ann Eldridge, and Kerrie McAloney for sample collection and processing and the staff of the Molecular Epidemiology Laboratory at QIMR for DNA sample processing and preparation.

Lothian Birth Cohorts of 1921 and 1936 (LBC)

The authors thank all LBC1921 and LBC1936 study participants and research team members who have contributed, and continue to contribute, to ongoing studies. LBC1921 was supported by the UK's Biotechnology and Biological Sciences Research Council (BBSRC), The Royal Society, and The Chief Scientist Office of the Scottish Government. LBC1936 is supported by the BBSRC, and the Economic and Social Research Council [BB/W008793/1] (which supports SEH), Age UK (Disconnected Mind project), and the University of Edinburgh. SRC is supported by a Sir Henry Dale Fellowship jointly funded by the Wellcome Trust and the Royal Society (221890/Z/20/Z). Genotyping of the cohorts was funded by the BBSRC (BB/F019394/1). Methylation typing was supported by Centre for Cognitive Ageing and Cognitive Epidemiology (Pilot Fund award), Age UK, The Wellcome Trust Institutional Strategic Support Fund, The University of Edinburgh, and The University of Queensland.

Systems Genomics of Parkinson's Disease (SGPD)

We thank all SGPD participants for donating their time, data, and biological samples, without which this study would not have been possible. This research was supported by the National Health and Medical Research Council (NHMRC: 1078037, 1078901, 1103418, 1107258, 1127440, 1113400), the Australian Research Council (ARC: DP160102400 and FT180100186) and the Mater Foundation. Support also came from ForeFront, a large collaborative research group dedicated to the study of neurodegenerative diseases and funded by the NHMRC (Program Grant 1132524, Dementia Research Team Grant 1095127, NeuroSleep Centre of Research Excellence 1060992) and ARC (Centre of Excellence in Cognition and its Disorders Memory Program CE10001021). The Queensland Parkinson's Project (QPP) was supported by a grant from the Australian National Health and Medical Research Council (1084560). The New Zealand Brain Research Institute (NZBRI) cohort was funded by a University of Otago Research Grant, together with financial support from the Jim and Mary Carney Charitable Trust (Whangarei, New Zealand). We thank Allison Miller for processing and handling of NZBRI samples.

Chinese Motor Neuron Disease Cohort (CHNMND)

We also thank staff and researchers at the Peking University Third Hospital (Beijing, China) and Matthew A. Brown for their role in generating this dataset.

Tibetan Han Chinese high-altitude (THCH)

The research was partly supported by the Leading Innovative and Entrepreneur Team Introduction Program of Zhejiang (2021R01013, JY), the National Natural Science Foundation of China (81522014, 82125007, ZBJ) and the Dr Jian Zhou Memorial Scholarship (FFC). We thank the High-Performance Computing Center and the Research Center for Industries of the Future (RCIF) at Westlake University for their assistance in computing.

Supplementary Tables

Supplementary Table 1: Characteristics of the study cohorts.

Abbreviations: SGPD, Systems Genomics of Parkinson's Disease; LBC, Lothian Birth Cohorts; BSGS, Brisbane Systems Genomics Study; CHNMND, Chinese Motor Neuron Disease Cohort; THCH, Tibetan-Han Chinese high-altitude; PD, Parkinson's disease; ALS, Amyotrophic lateral sclerosis; EUR, European; EAS, East Asian; DNAm DNA methylation; SNPs, single nucleotide polymorphisms; #, Number; QC, quality control.

Cohort	SGPD	LBC	BSGS	CHNMND	THCH
Sample size	1659	1437	605	651	1448
Data type	PD case-control	Longitudinal	Familial	ALS case-control	Cohort study
Ancestry	EUR (Australian, New Zealand)	EUR (Scottish)	EUR (Australian)	EAS (Chinese)	EAS (Chinese)
DNAm array	Illumina 450K	Illumina 450K	Illumina 450K	Illumina 450K	Illumina 450K
Genotype array	Illumina PsychArray-B.bpm	Illumina 610-Quad Beadchip arrays	Illumina 610-Quad Beadchip arrays	Illumina Human OmniZhong Hua	Illumina HumanCoreExome-12 BeadChip
# SNPs post imputation and QC	7,664,320	8,297,026	8,240,066	7,330,123	6,461,686

Supplementary Table 2: Relationship between increase in sample size and reduction in fine mapping causal set size determined via simulation. Subsets of the UK Biobank (UKB) were selected to match the number of individuals in mQTL cohorts; EUR (n=3,701) and combined across ancestries (n=5,800), as well as increases by 1,000 sample increments (n=6,800, 7,800, 8,800, 9,800). 4,528 mQTLs in the stringent set with the same lead SNP in both ancestries were utilised for simulation, with a single DNAm phenotype simulated for each for mQTL assuming the lead EUR SNP as causal. The resulting mQTLs were fine mapped using SuSiE, with mean credible set size and standard error of the mean (SE) recorded across DNAm probes and UKB subsets and the mean percentage reduction calculated. This has been compared to the observed data for the same mQTLs.

	Sample	Mean causal set size (SE)	Mean % reduction
Real mQTL data	EUR n=3,701	3.1 (0.04)	
	EAS n=2,099	3.3 (0.05)	
	Cross-ancestry n=5,800	1.5 (0.01)	26.1% compared to EUR n=3,701<br>29.9% compared to EAS n=2,099
Simulated mQTL data	UKB n=3,701	2.9 (0.06)	
	UKB n=5,800	2.3 (0.05)	5.7% compared to UKB n=3,701
	UKB n=6,800	2.2 (0.05)	8.4% compared to UKB n=3,701
	UKB n=7,800	2.1 (0.04)	11.0% compared to UKB n=3,701
	UKB n=8,800	2.0 (0.04)	13.4% compared to UKB n=3,701
	UKB n=9,800	1.9 (0.03)	14.7% compared to UKB n=3,701

Supplementary Table 3: Identification of ancestry-specific mQTL at 21,084 EUR and 7,841 EAS DNAm probes. a) EUR and b) EAS ancestry-specific mQTL. Ancestry-specific mQTLs were identified using the Bonferroni corrected, two-sided p-values threshold of $p<10^{-10}$ in the given ancestry and $p>10^{-6}$ in the other. P-values were calculated at the cohort level from linear regression and mixed linear regression models, and at the ancestry level using inverse variance-weighted meta-analysis. For a given ancestry, the ancestry specific mQTL are classified by the number of cohorts of that ancestry in which the mQTL was significant prior to meta-analysis.

a) 21,084 EUR ancestry-specific mQTL

# of EUR cohorts	# DNAm probes with ancestry-specific mQTL (%)
All 3 cohorts	1,255 (6.0%)
2 cohort	5,014 (23.8%)
1 cohort	7,929 (37.6%)
No cohort	6,886 (32.7%)

b) 7,841 EAS ancestry-specific mQTL

# of EAS cohorts	# DNAm probes with ancestry-specific mQTL (%)
Both cohorts	2,250 (28.7%)
1 cohort	3,252 (41.5%)
No cohort	2,339 (29.8%)

Supplementary Figures

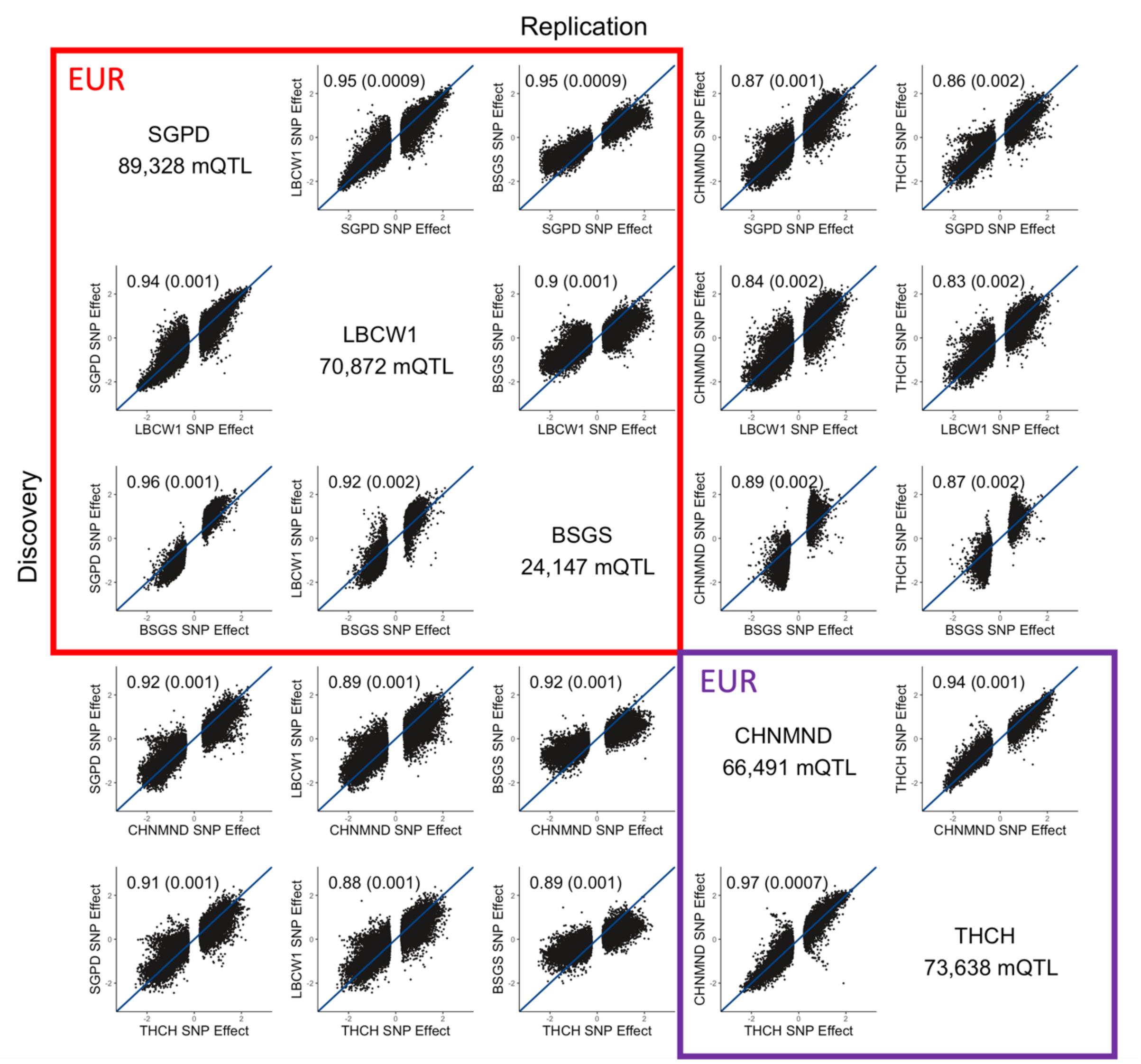

Supplementary Figure 1: The correlation (r_b) of cis-mQTL SNP effects across cohorts. Shown are effect sizes of the lead SNPs from the discovery cohort and the corresponding SNP effect in the replication cohort. Correlations are presented with corresponding standard errors in parentheses. Cohorts of the same ancestry, boxed in red (EUR) and purple (EAS), have more similar effect sizes than across ancestries.

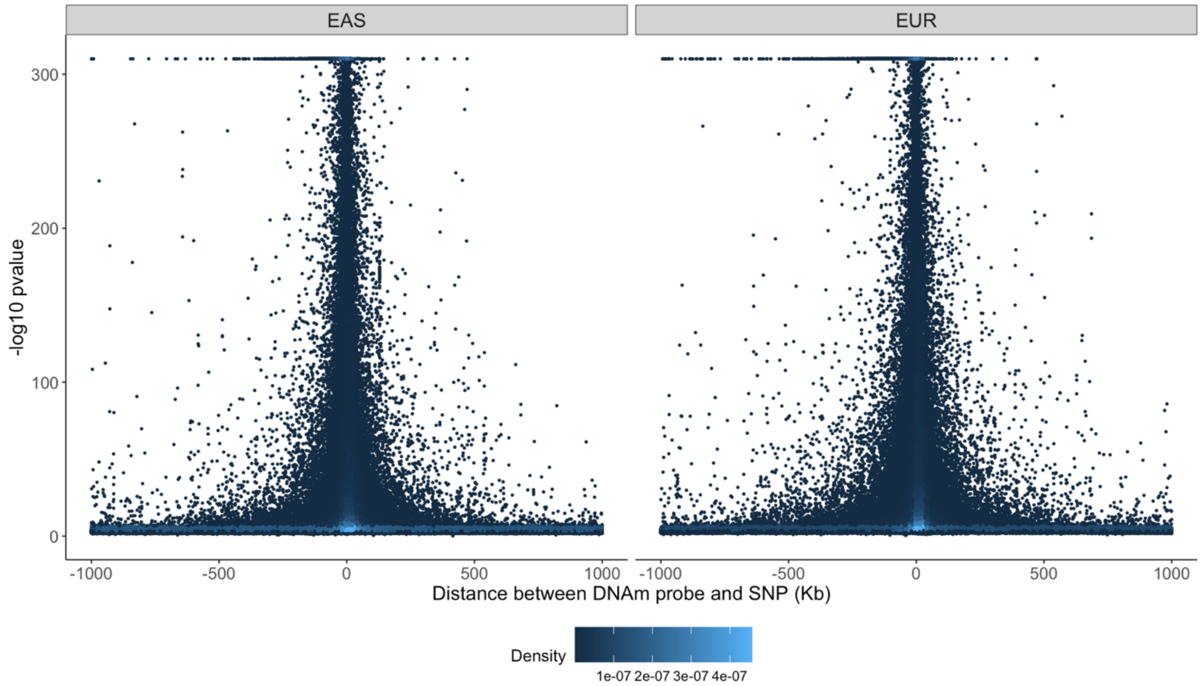

Supplementary Figure 2: The significance of *cis*-mQTL associations increases as the distance between the genetic variant and DNAm site decreases (n=404,503). Shown is the relationship for between mQTL significance (–log10 p-value) and distance between the DNAm probe and genetic variant in each ancestry. The colour of the points reflects the density of observations at that location. The two-sided p-values from an inverse variance-weighted meta-analysis with the the ceiling in –log10 p-value due to numerical limitations in analysis software.

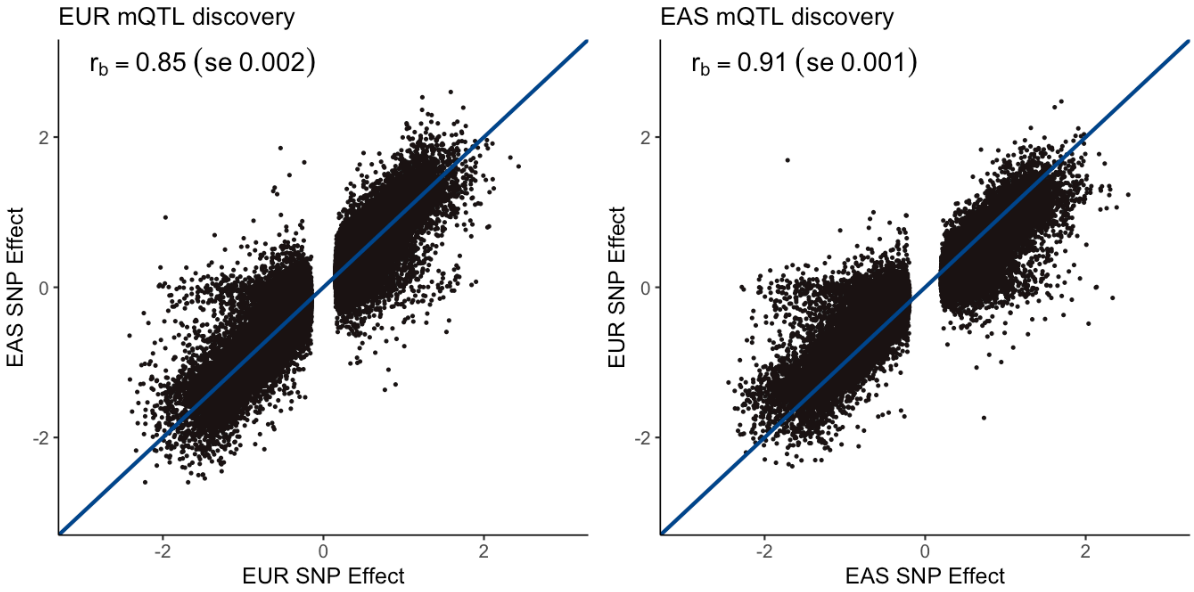

Supplementary Figure 3: The correlation (r_b) of *cis*-mQTL SNP effects between meta-analysed ancestries. Shown are effect sizes of the lead SNPs from the discovery ancestry and the corresponding SNP effect in the other ancestry. Correlations are presented with corresponding standard errors in parentheses. EUR mQTL discovery (n=113,976), EAS mQTL discovery (n=95,583).

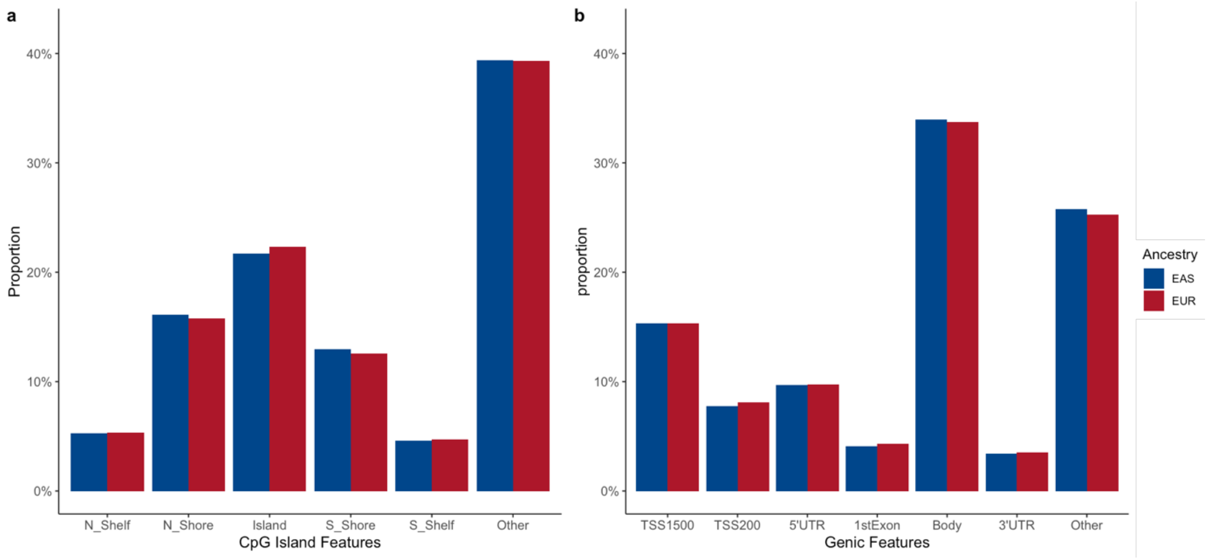

Supplementary Figure 4: Comparing the distribution of DNAm probes associated with mQTL between genic and CpG island regions by ancestry. Bar-plots demonstrating the location of DNAm probes with associated mQTL in individuals of EUR ancestry (blue bars; n=113,976) compared to individuals of EAS ancestry (red bars; n=95,583).

a) annotation relative to CpG islands. Shores are defined as island flanking regions ranging to up to 2,000 bp and shelves are defined as island flanking regions ranging from 2,001 bp to 4,000 bp. Northern and southern shores and shelves (noted N_ and S_) are respectively defined as the upstream and downstream shores or shelves according to chromosomal coordinates.

b) gene region feature category describing the CpG position. Genic features include

Gene features listed include: promoter region ranging from 1 bp to 200 bp upstream of the TSS (TSS200), promoter region ranging from 201 bp to 1,500 bp upstream of the TSS (TSS1500), 5' untranslated region (5'UTR), first exons (1stExon), gene bodies, excluding the 5' and 3' UTRs and first exons (Body) and 3' untranslated region (3'UTR).

We observed no difference in the distribution of DNAm sites associated with mQTLs between ancestries, across genic or CpG island features.

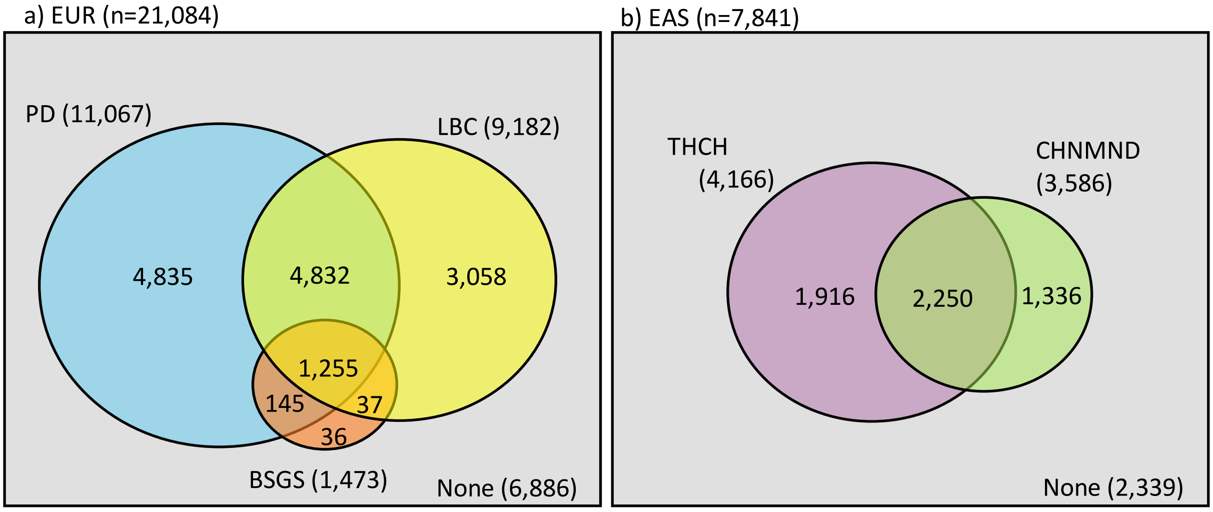

Supplementary Figure 5: Identification of 21,084 EUR and 7,841 EAS DNAm probes with ancestry-specific mQTL. Shown is the number of those mQTL-probes which were significant in each of the cohorts of the same ancestry and the overlap between cohorts.

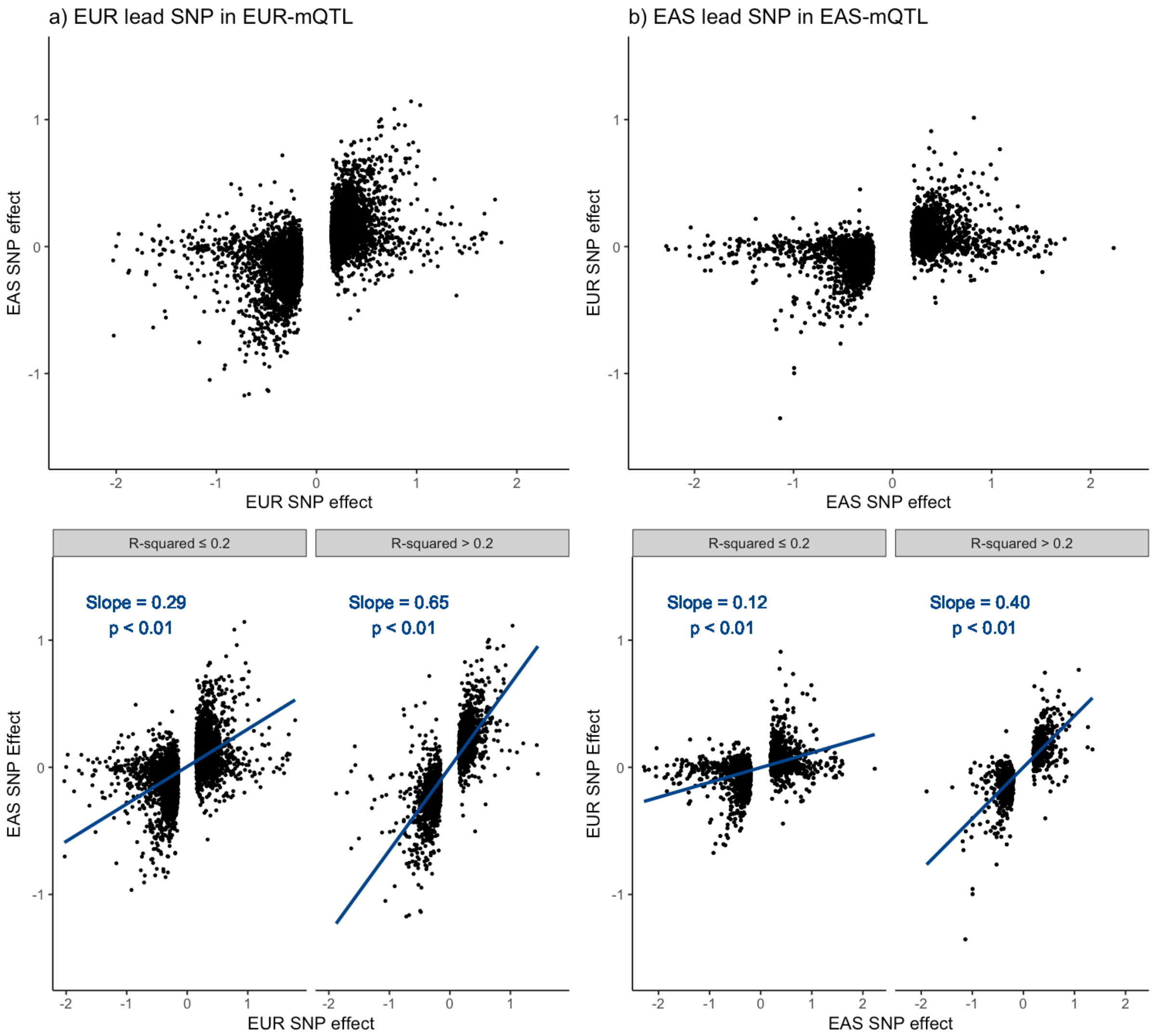

Supplementary Figure 6: Concordance of lead SNP effects by LD between lead SNPs for a) EUR lead SNP in EUR-mQTL and b) EAS lead SNP in EAS-mQTLs. Top: Agreement in SNP effects. Bottom: Agreement in SNP effect by LD between lead SNPs. We observe there is greater agreement between SNP effects when there is stronger LD, indicating some residual overlap in effect sizes attributable to mQTL that did not achieve significance due to power constraints.

Supplementary Figure 7: Manhattan plots of the 163 ancestry-specific pleiotropic associations.

For each mQTL-trait pair the Manhattan plots display:

a) mQTL associations in individuals of EUR ancestry
b) mQTL associations in individuals of EAS ancestry
c) Trait associations in individuals of EUR ancestry
d) Trait associations in individuals of EUR ancestry

The blue line indicates the significance threshold ($p < 10^{-10}$) in mQTL analysis, the grey line is the replication threshold ($p < 10^{-6}$), and the green line indicated the significance threshold ($p < 5x10^{-8}$) in GWAS. The SNP highlighted in red is the ancestry-specific lead SNP used as the exposure outcome in SMR analysis.

Trait A10: probe cg13096128

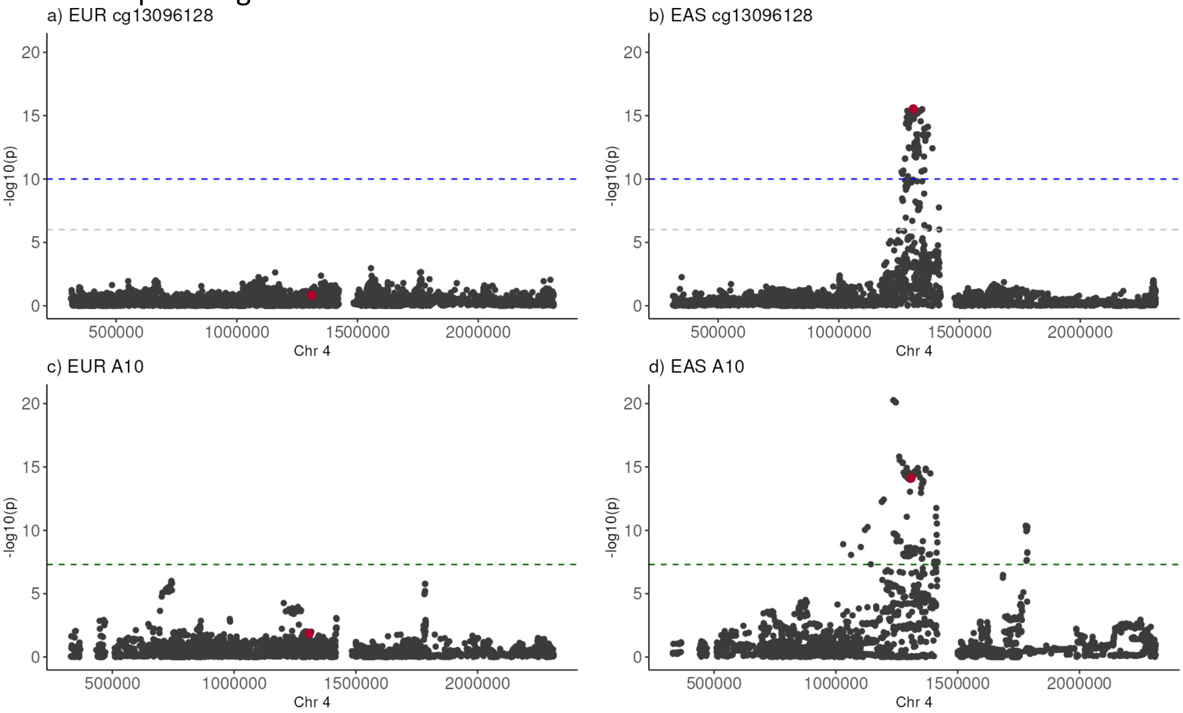

Trait AD: probe cg09621958

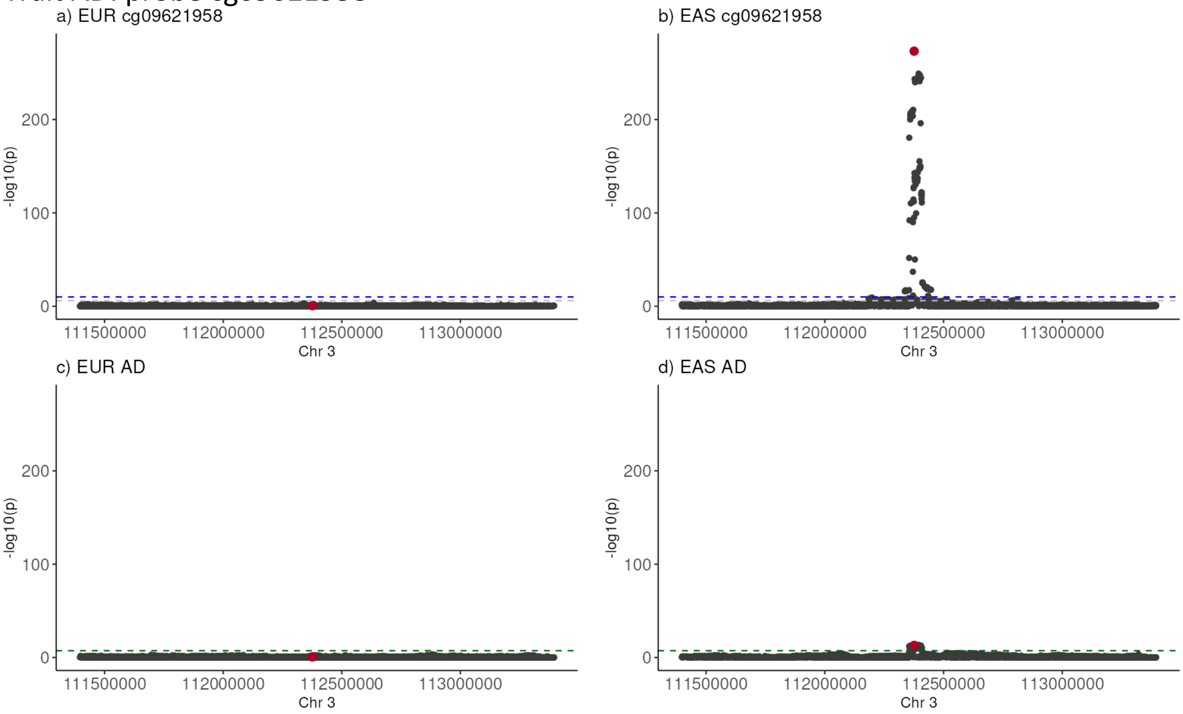

Trait AD: probe cg16799191

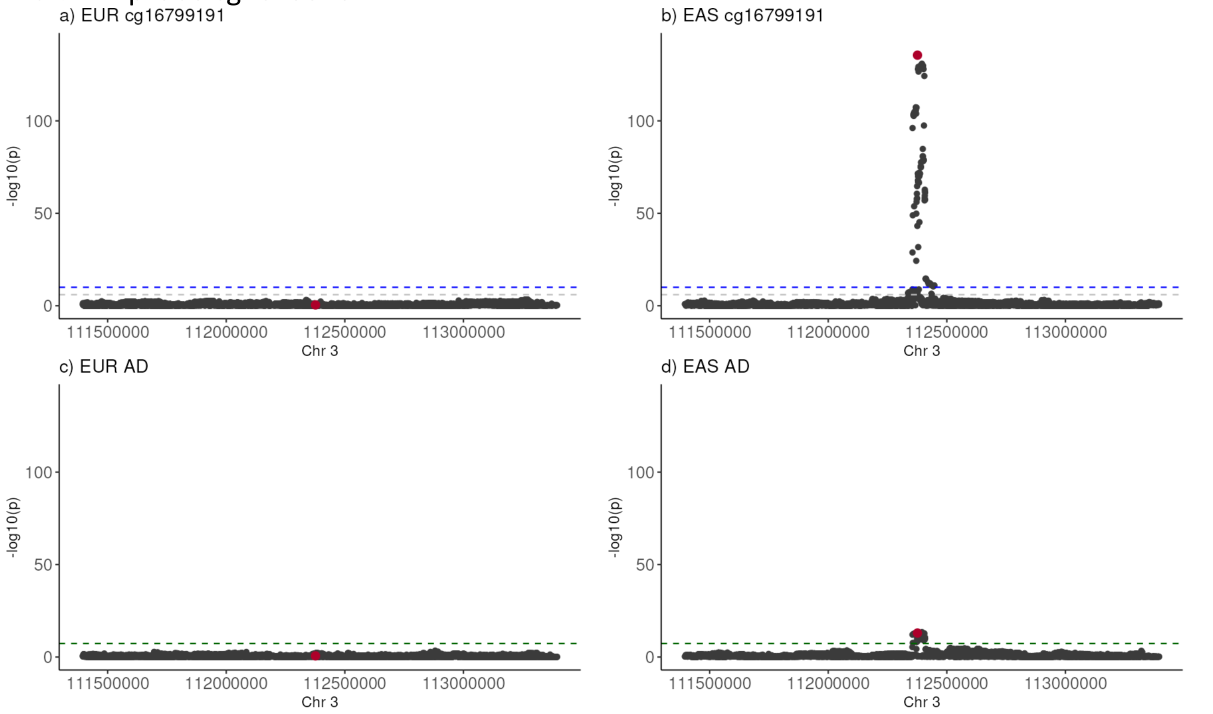

Trait B01A: probe cg20327444

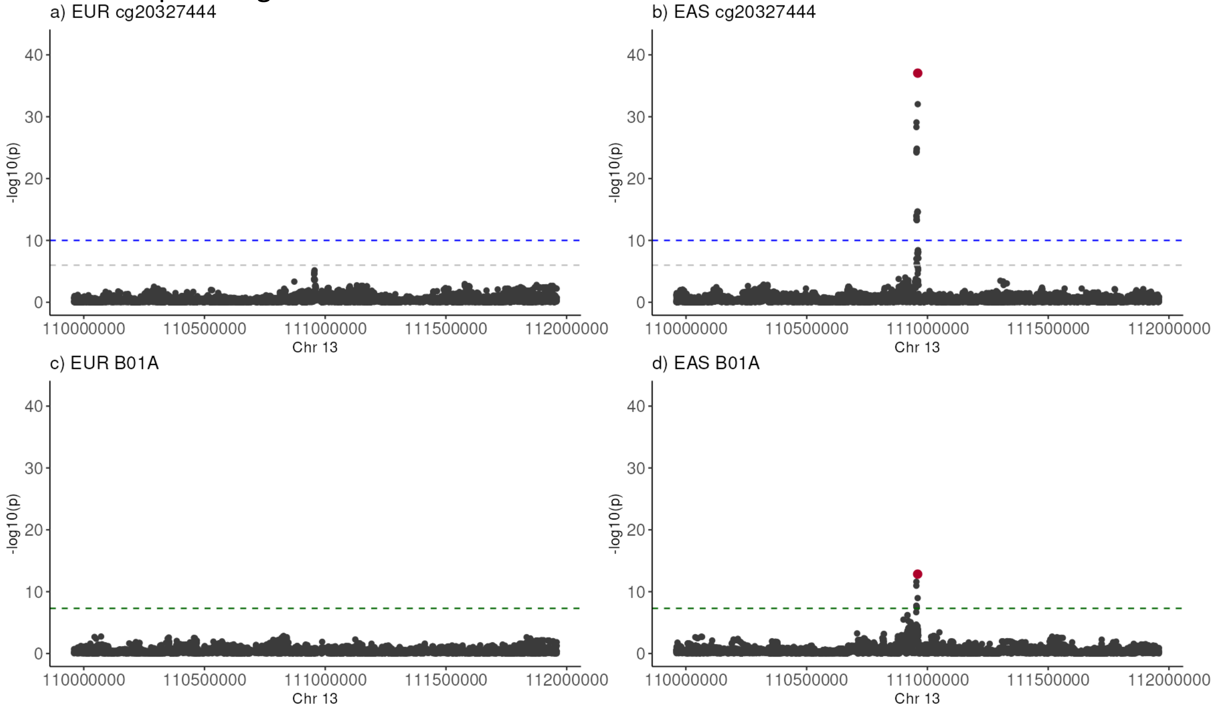

Trait BMI: probe cg06801483

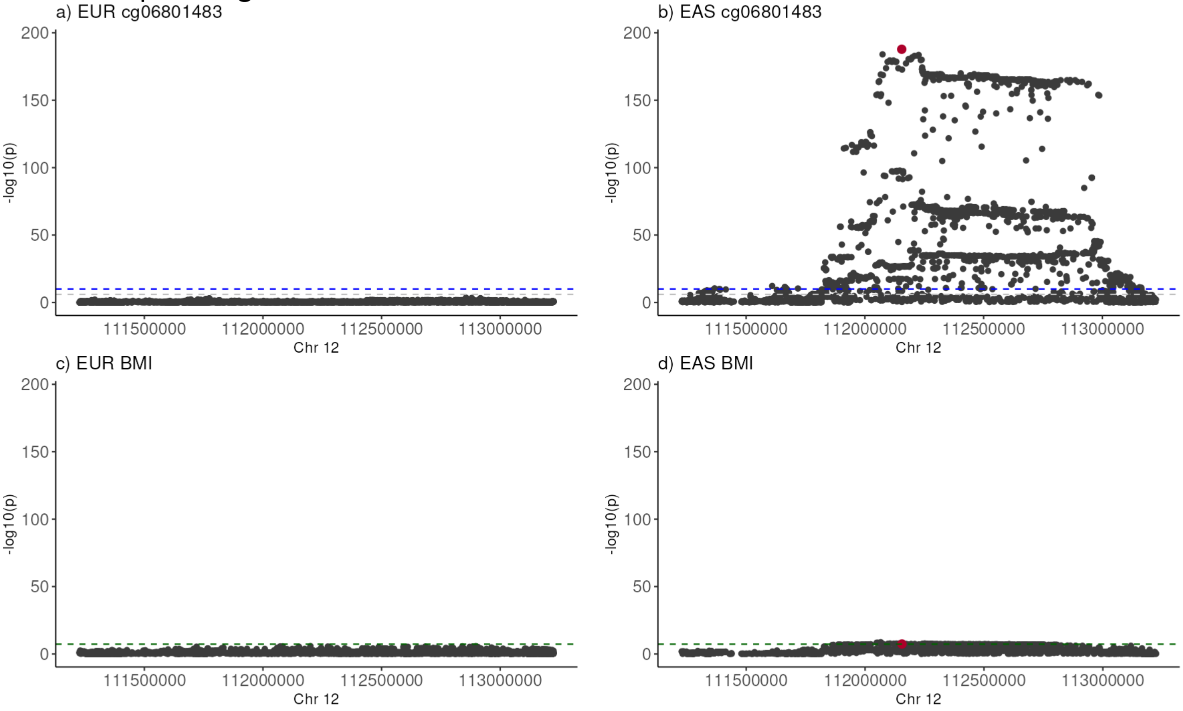

Trait C07: probe cg02525926

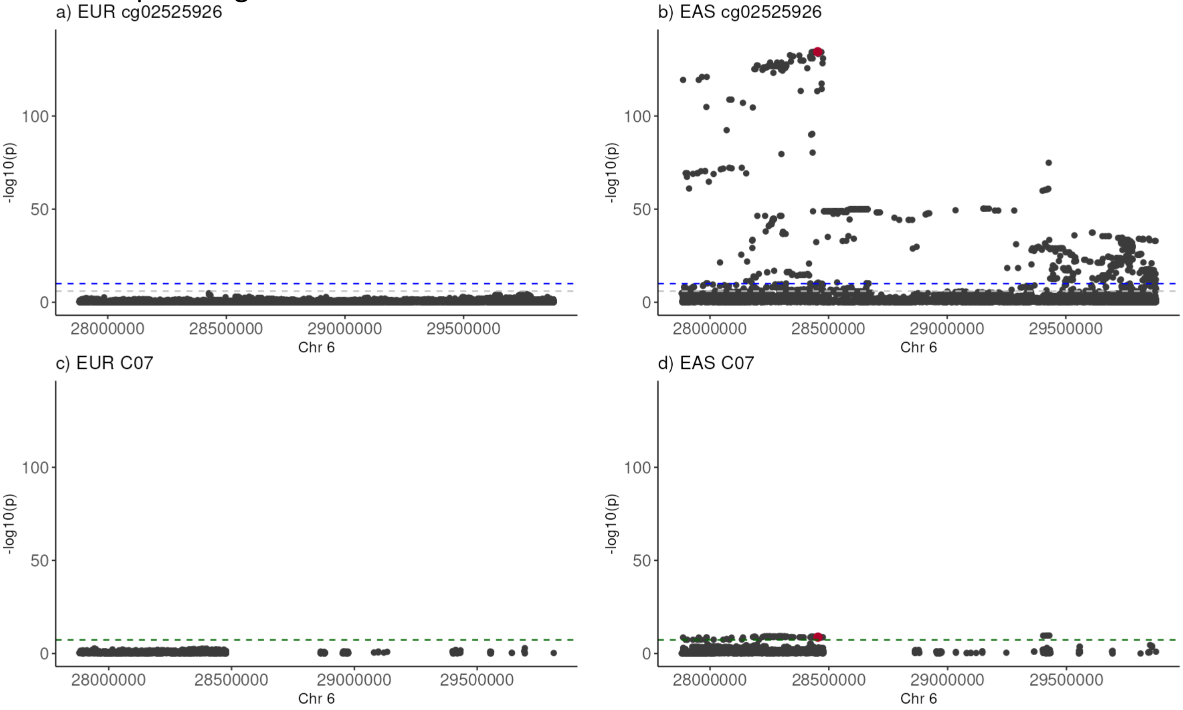

Trait C09: probe cg02525926

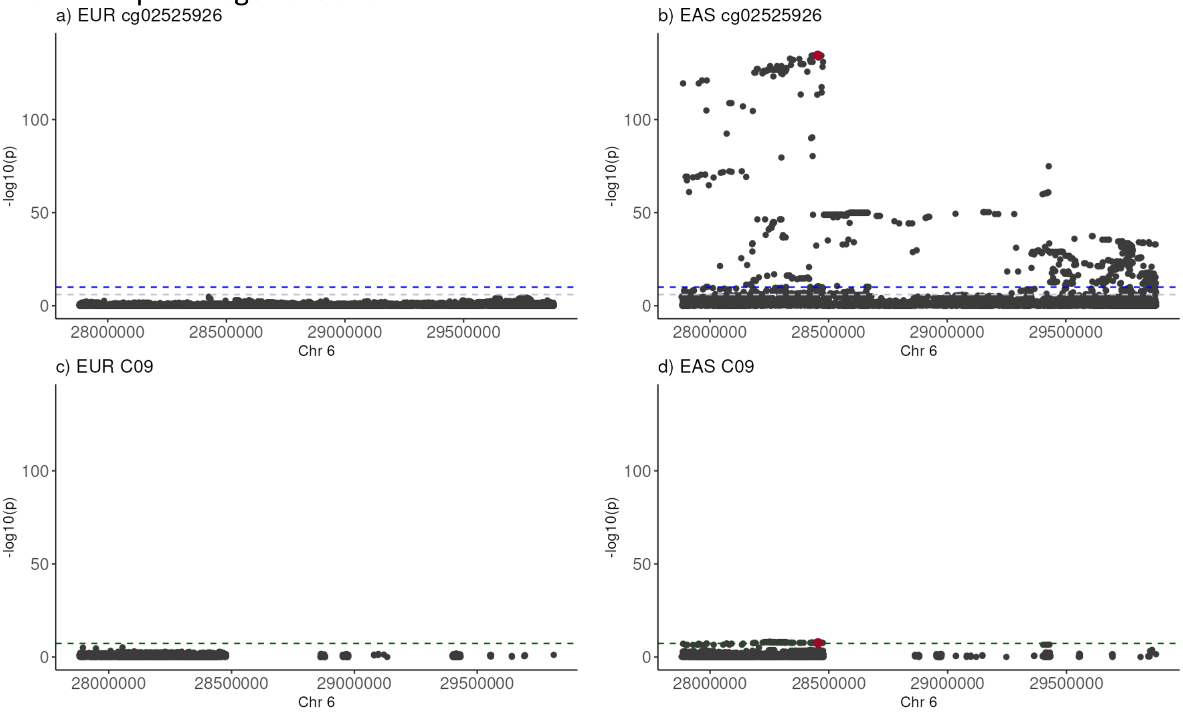

Trait GGT: probe cg06853455

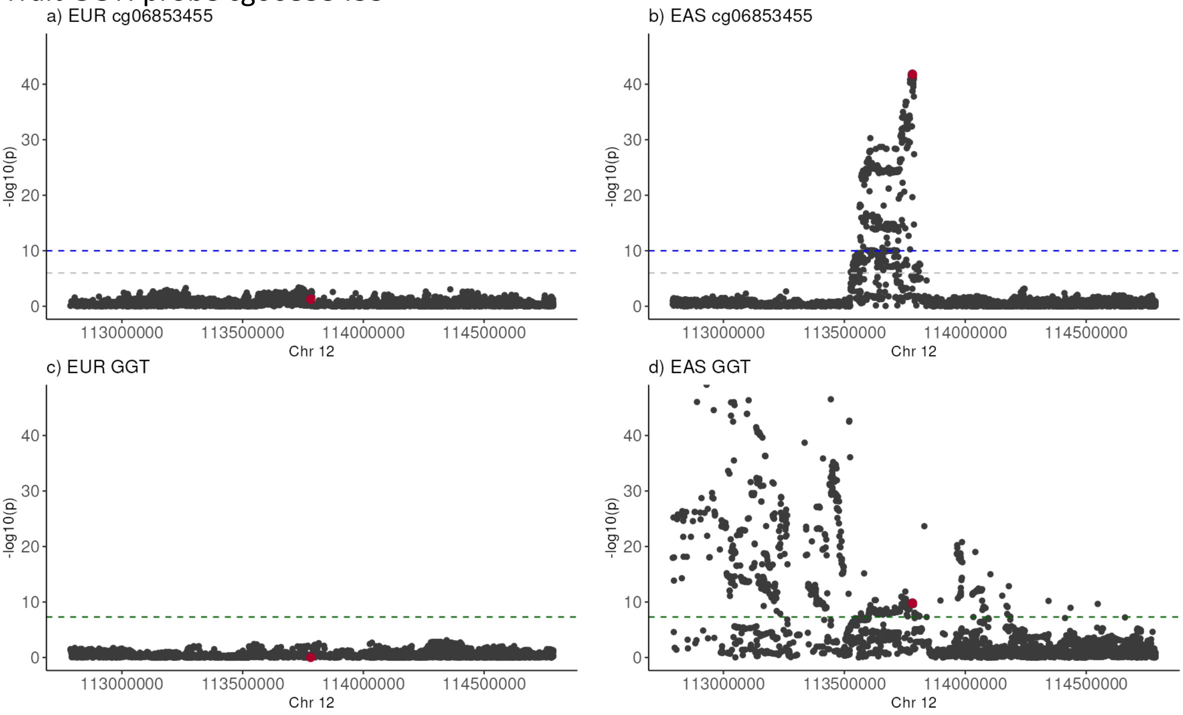

Trait IS: probe cg20327444

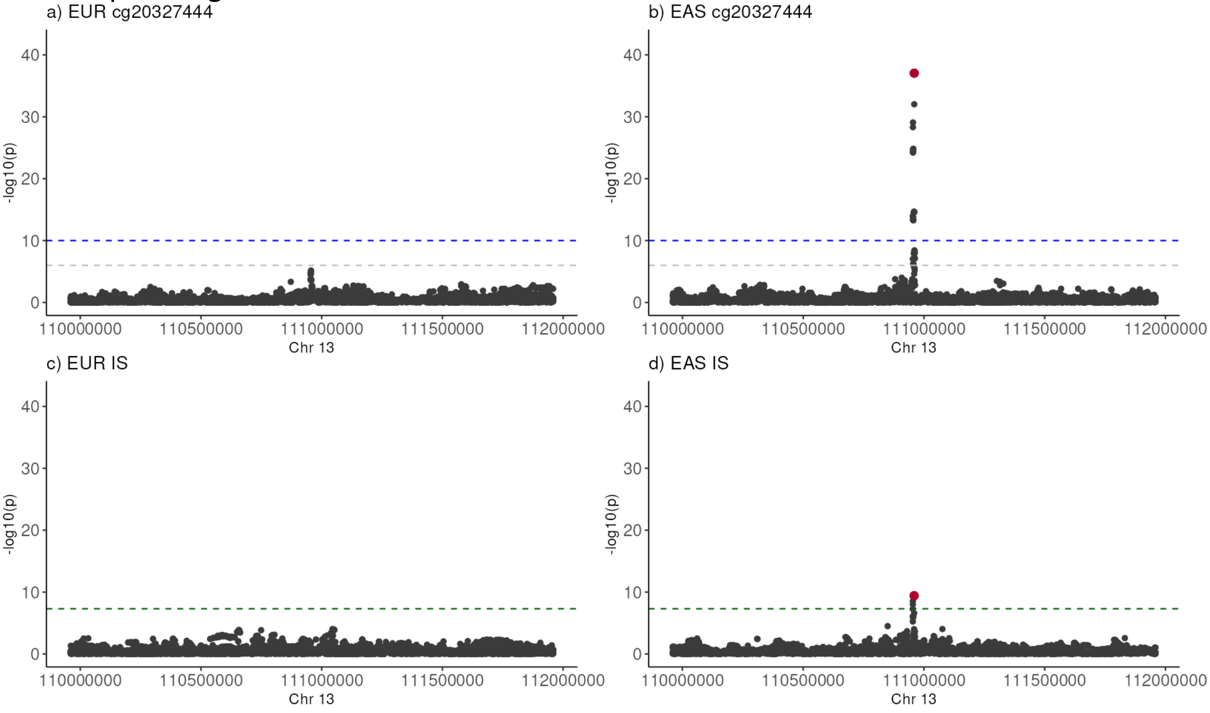

Trait N02BA: probe cg20327444

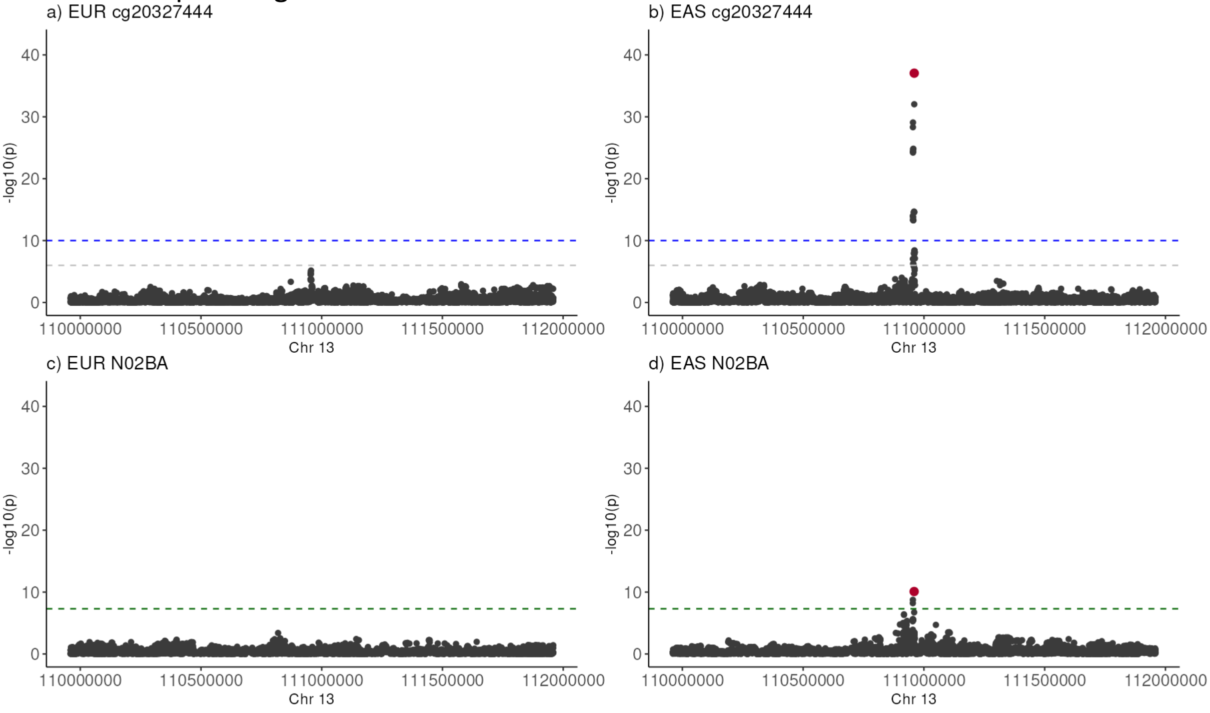

Trait RBC: probe cg22683016

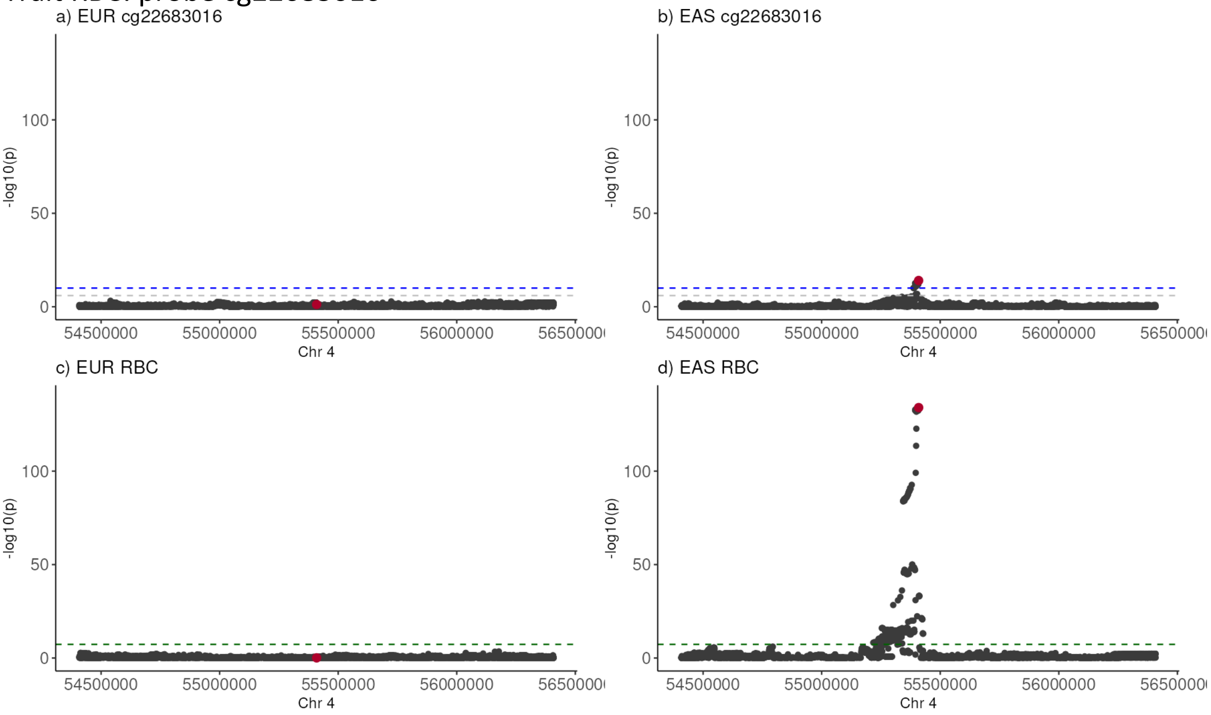

Trait RBC: probe cg18700744

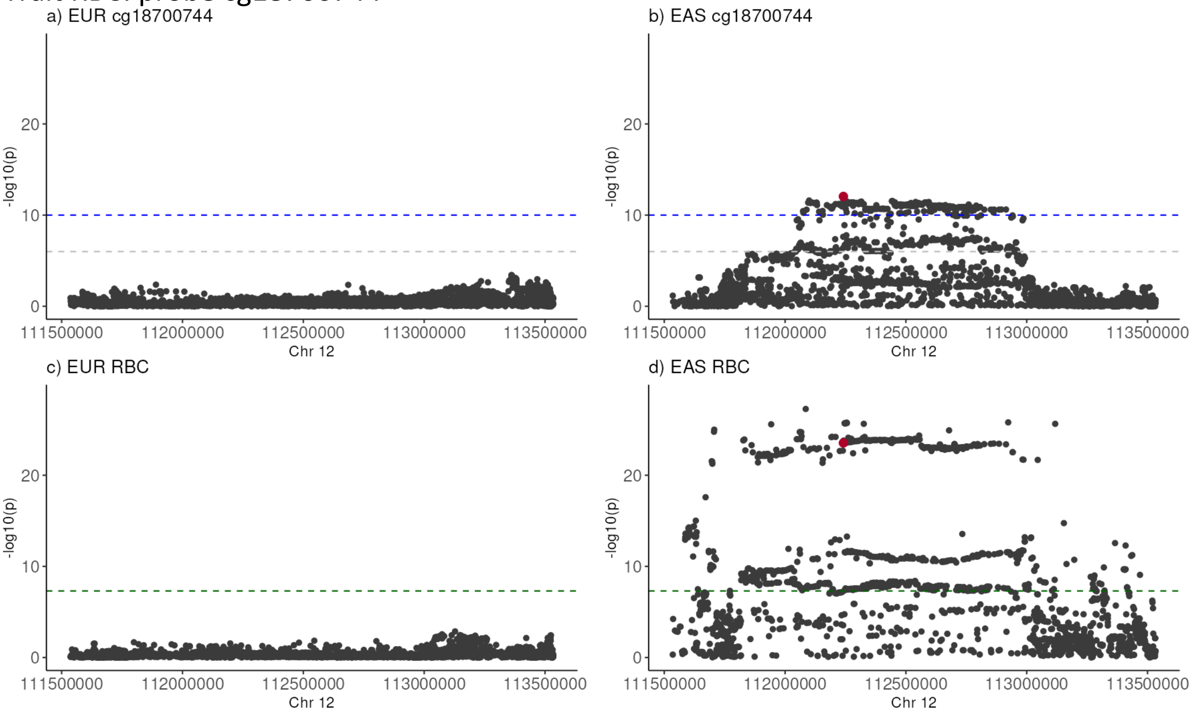

Trait SAP: probe cg24148767

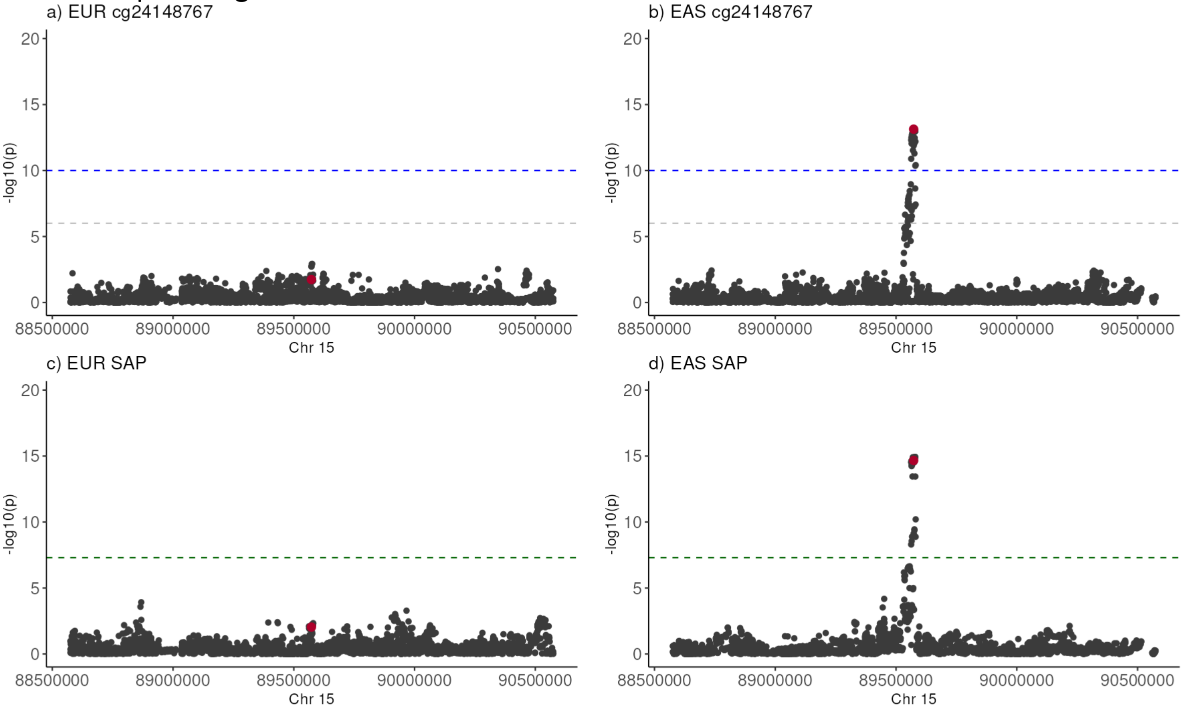

Trait T2D: probe cg13096128

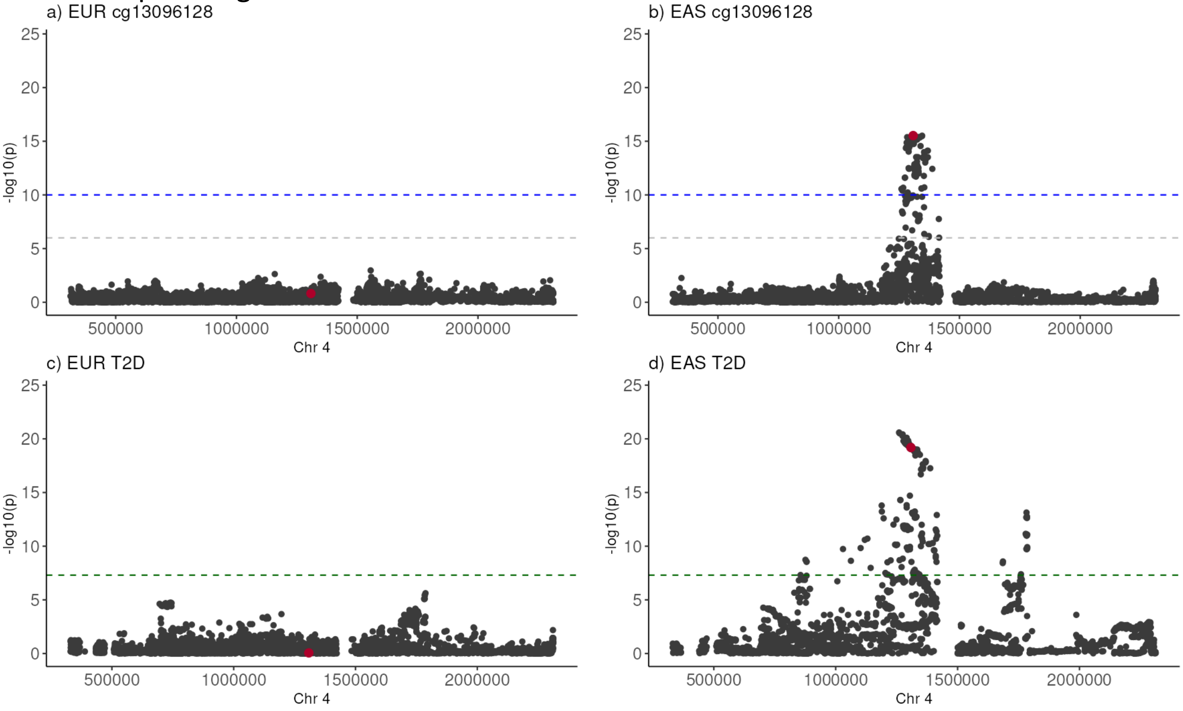

Trait T2D: probe cg04847110

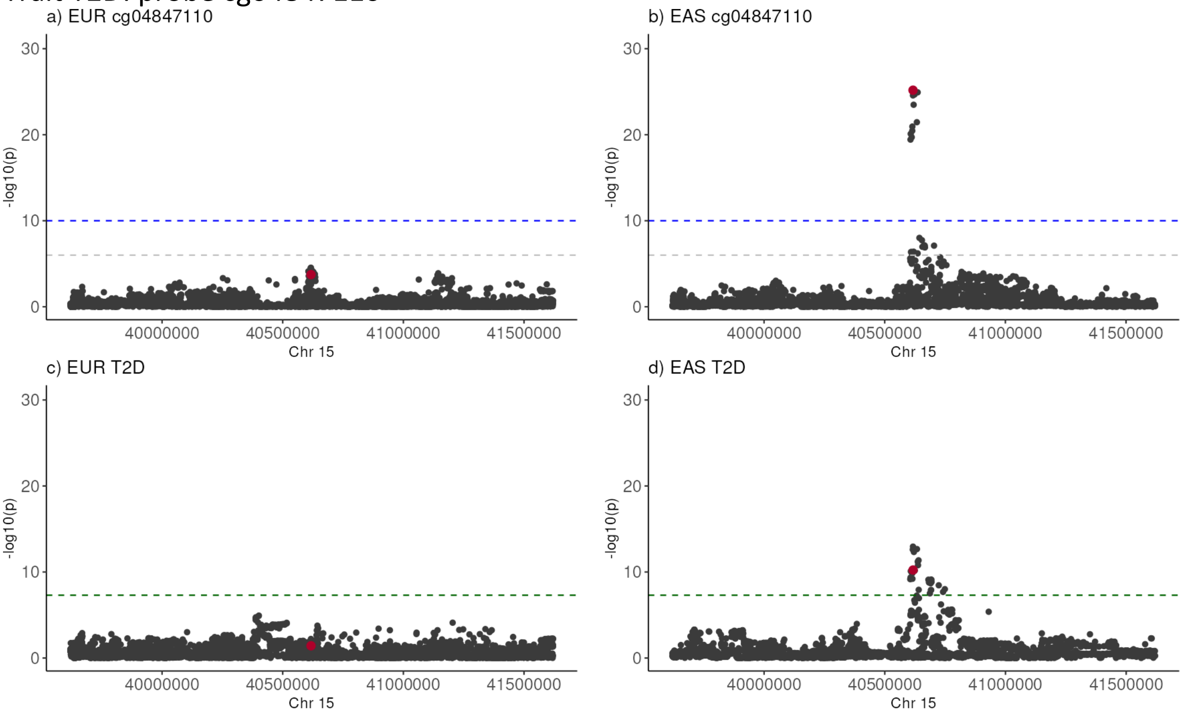

Trait WBC: probe cg13474750

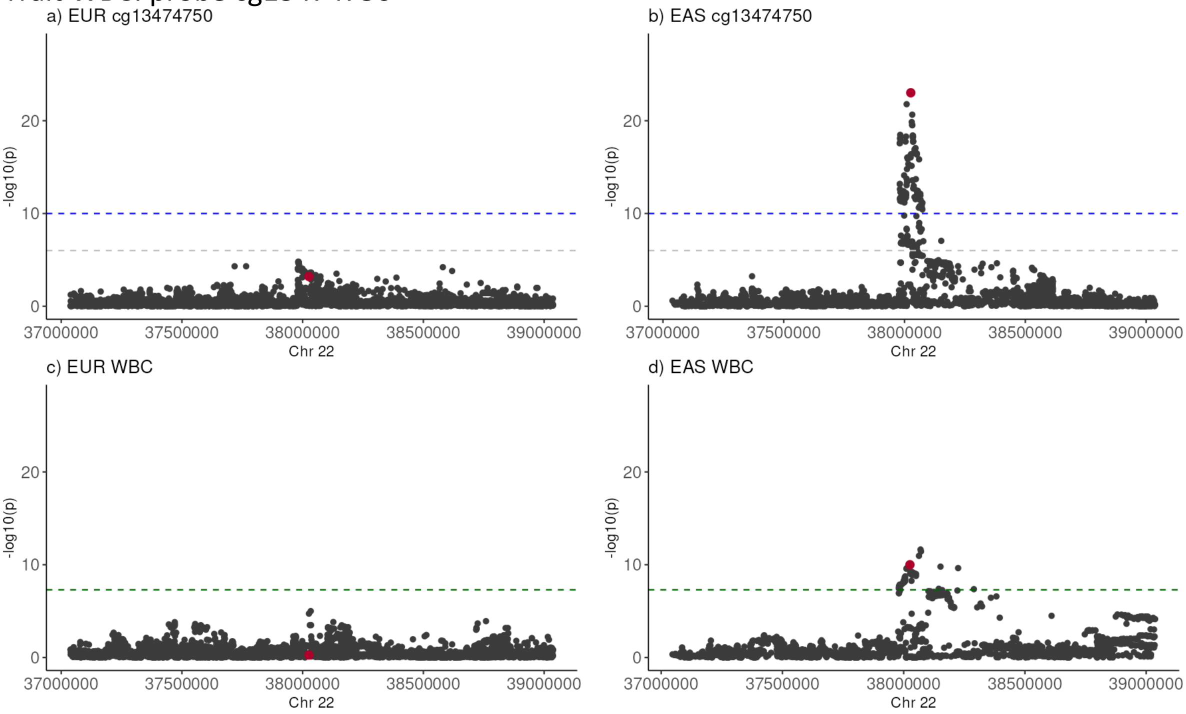

Trait ALP: probe cg16958793

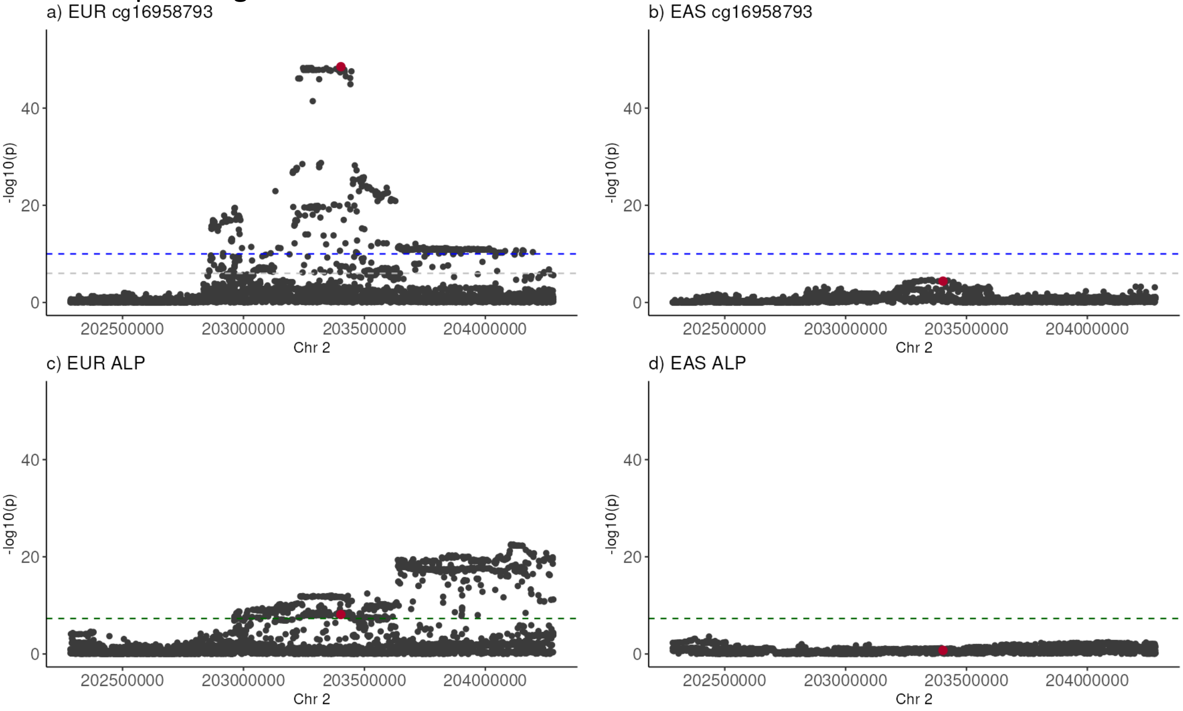

Trait ALP: probe cg09642020

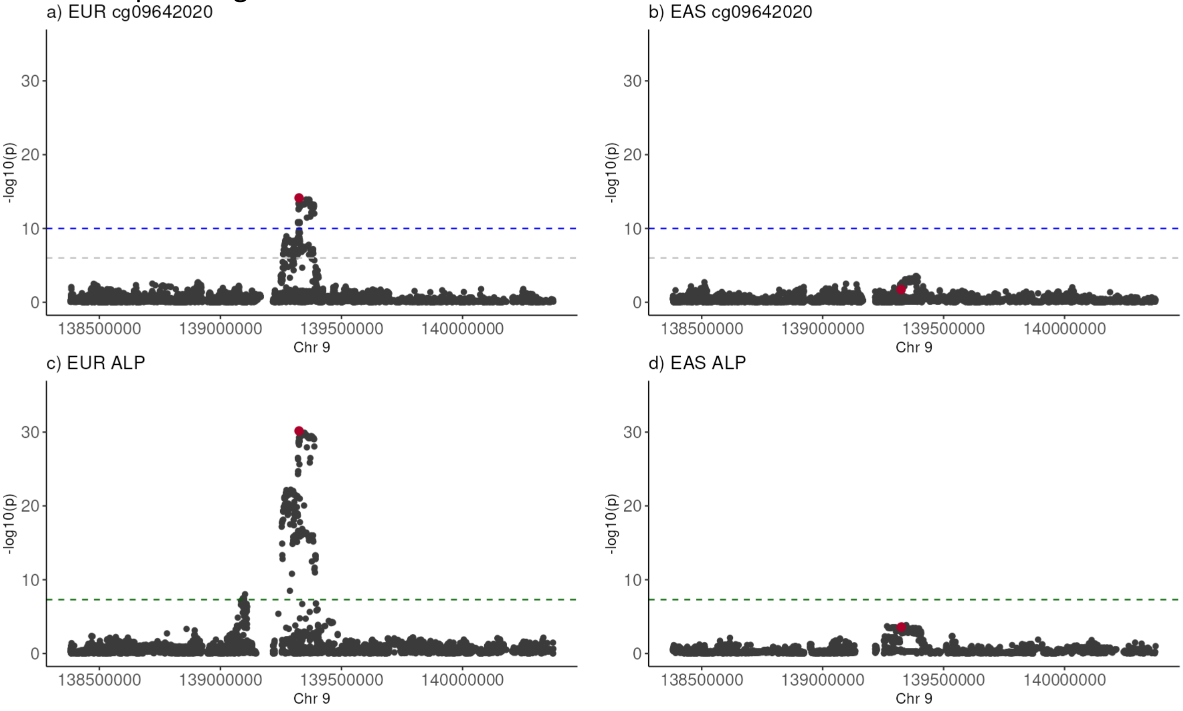

Trait ALP: probe cg13879547

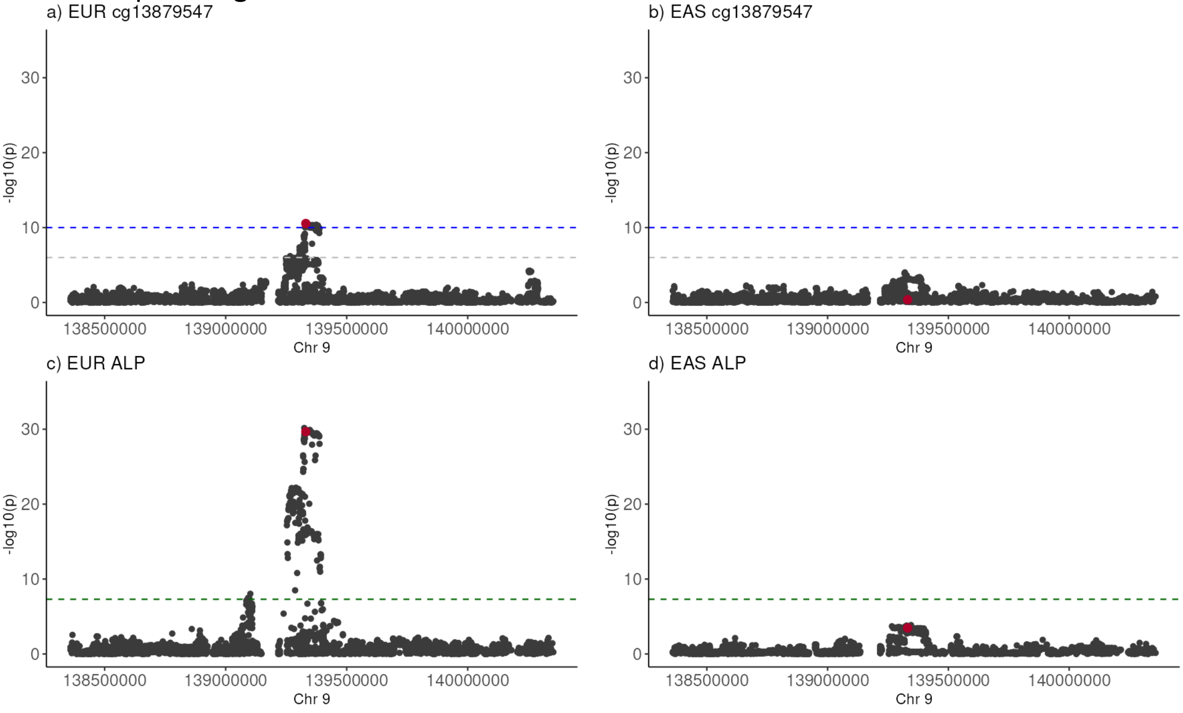

Trait ALP: probe cg02991085

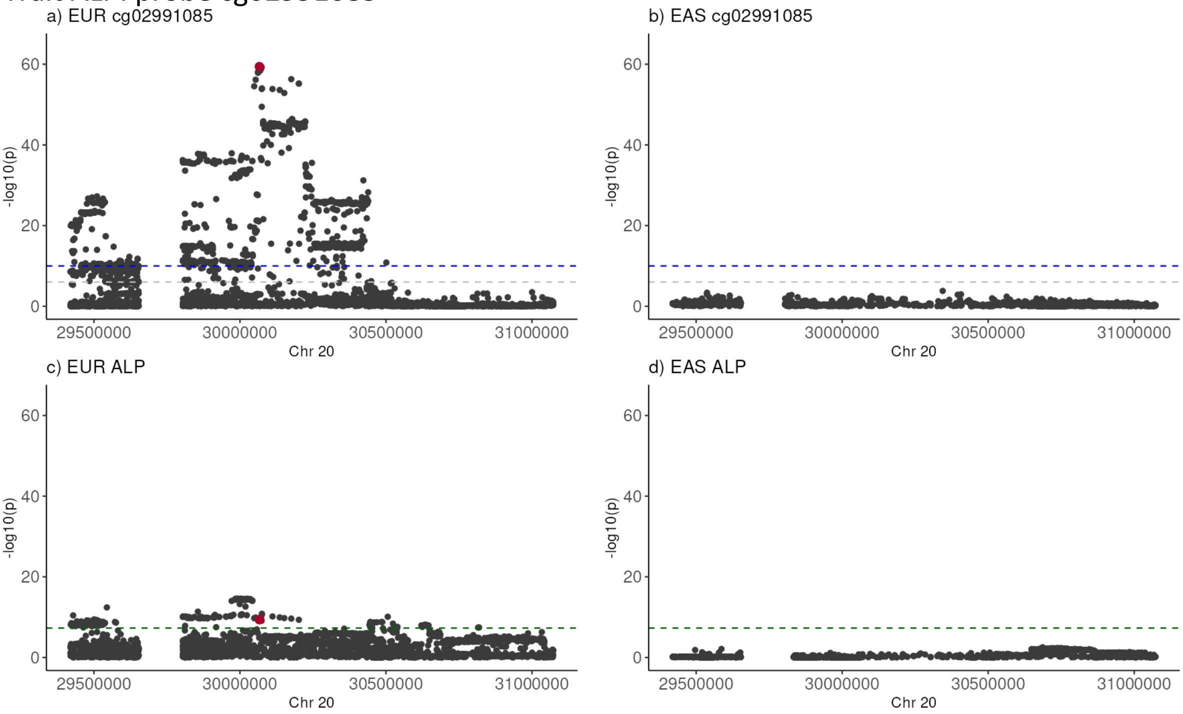

Trait ALP: probe cg13159946

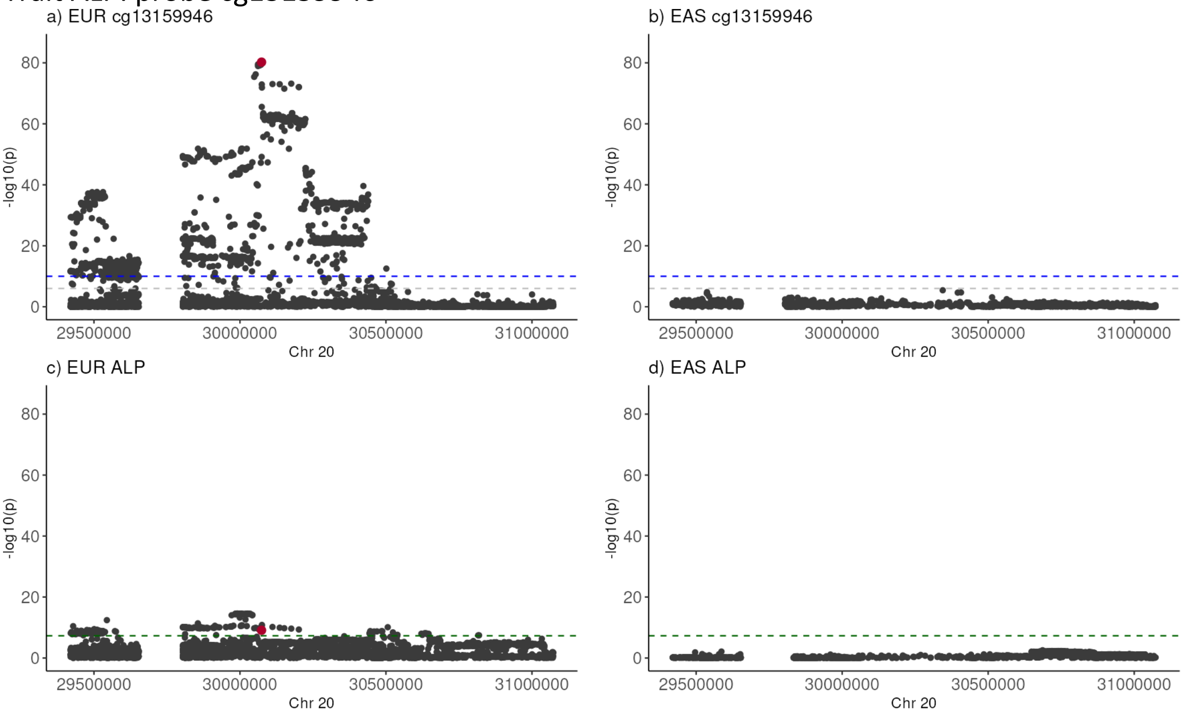

Trait ALP: probe cg25502144

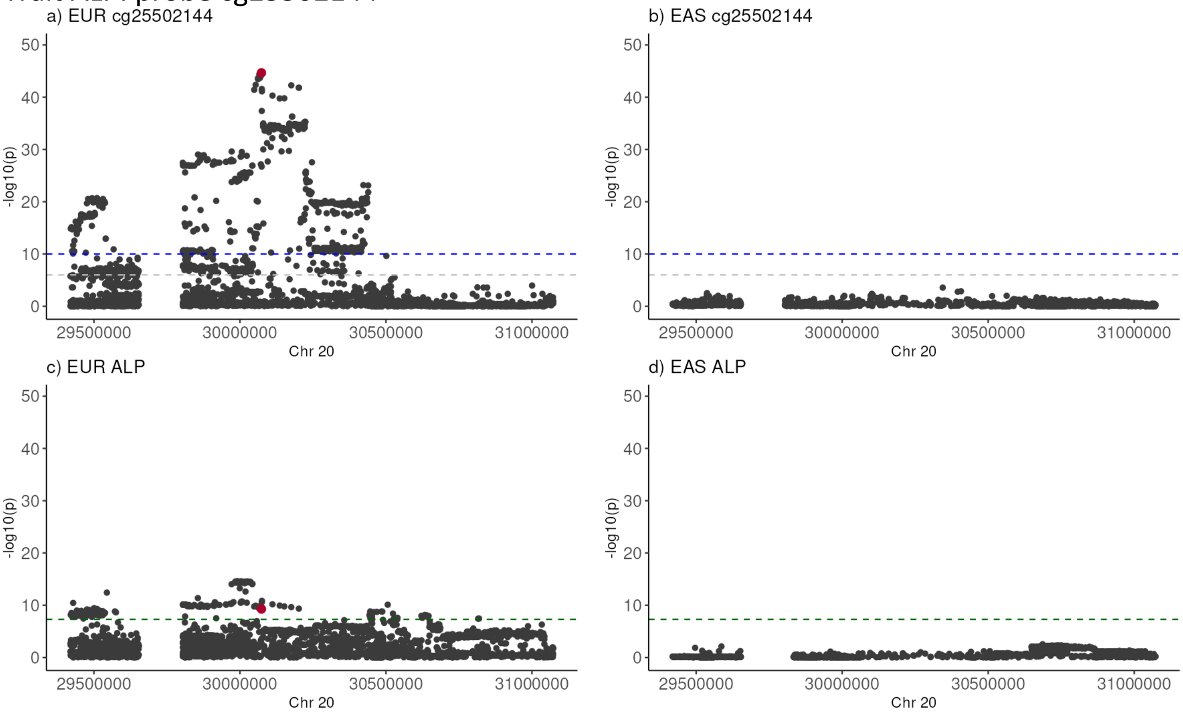

Trait ALT: probe cg05914712

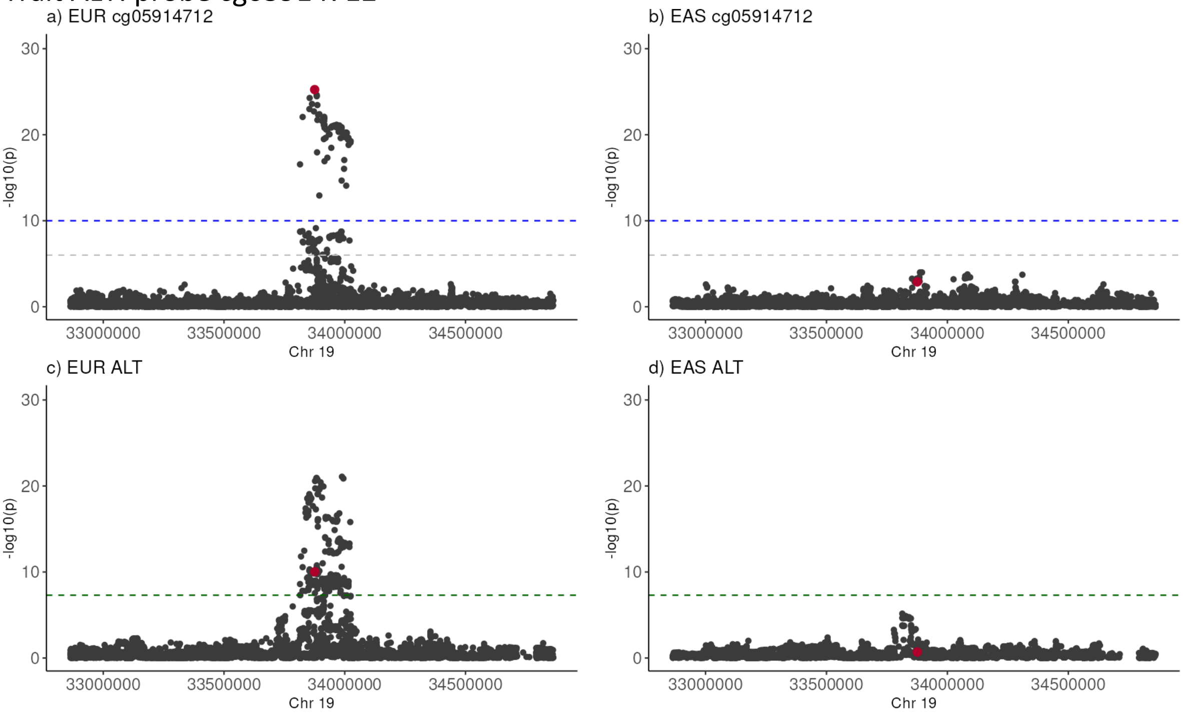

Trait As: probe cg18318560

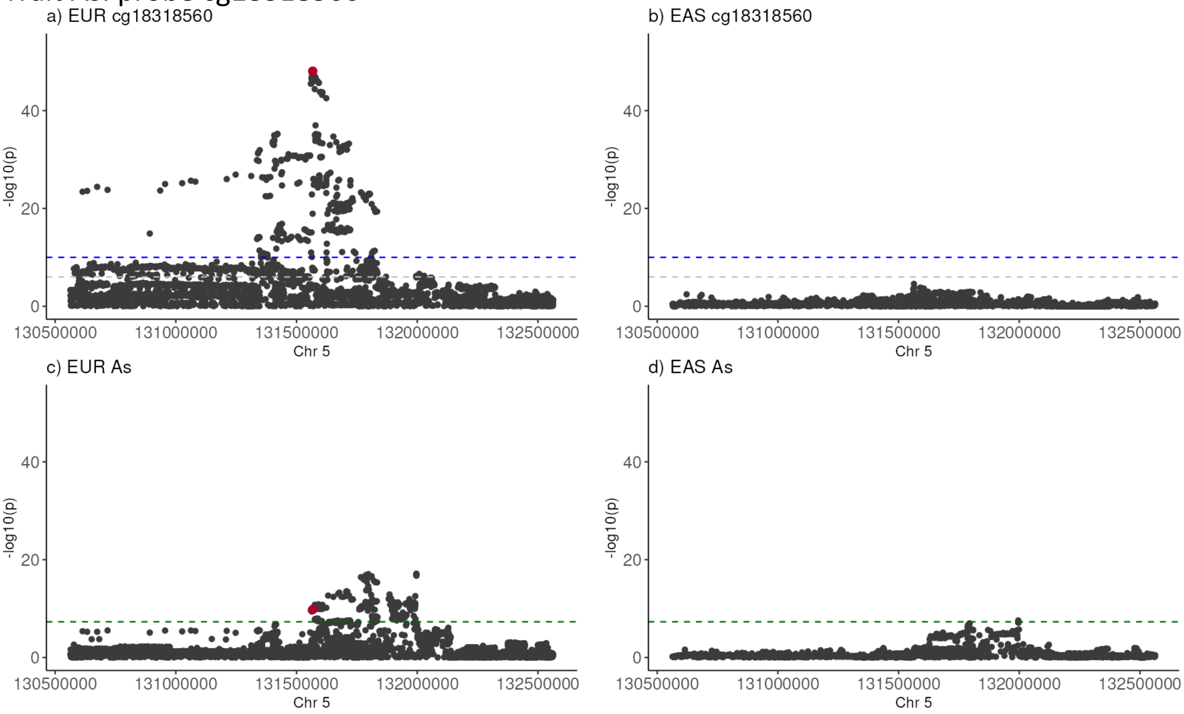

Trait As: probe cg24032190

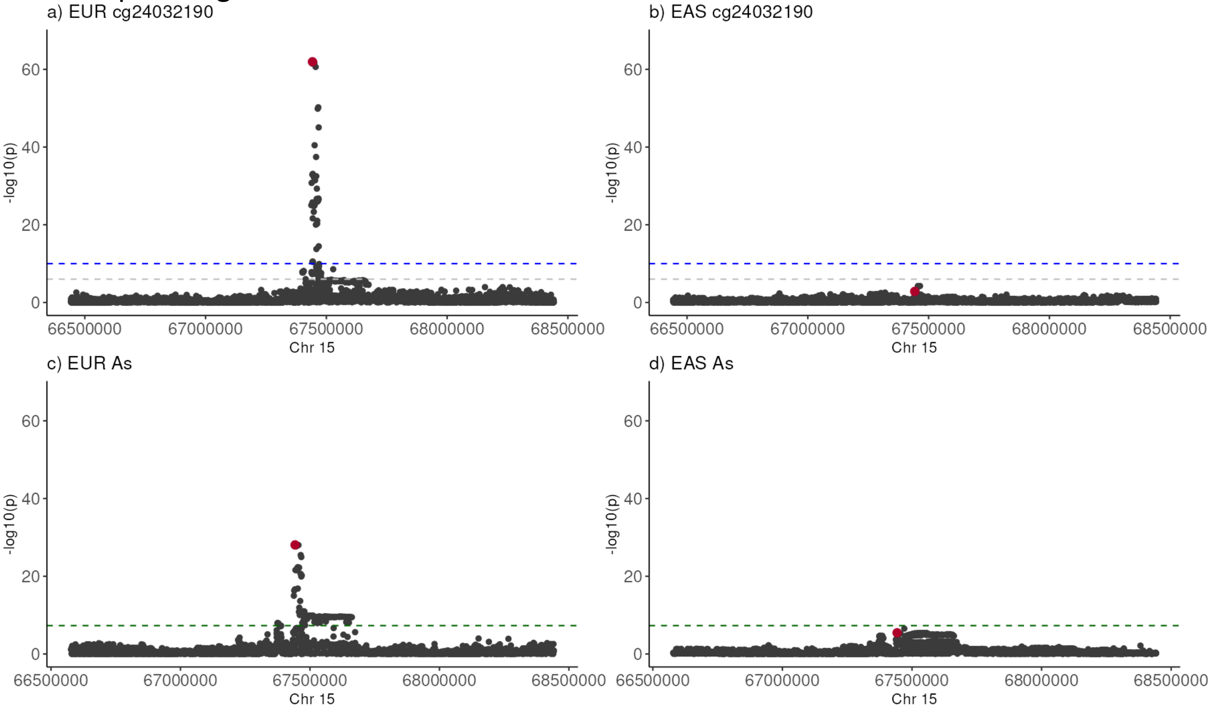

Trait AST: probe cg17642041

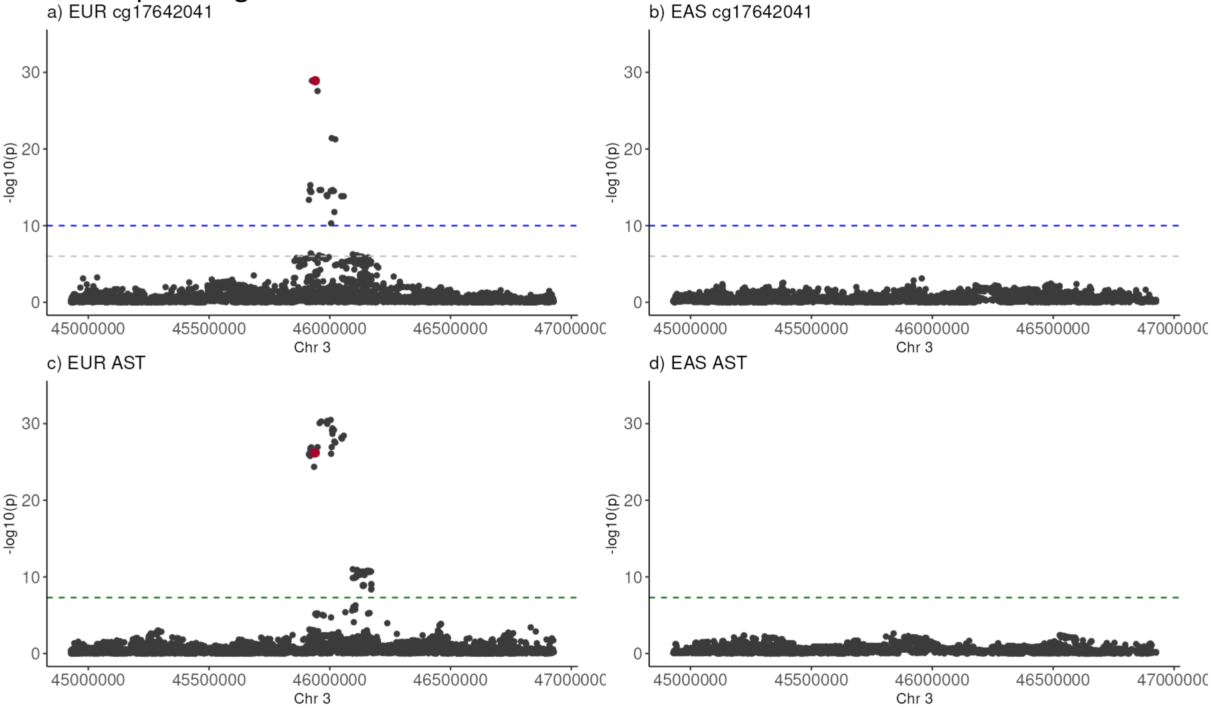

Trait AST: probe cg22022041

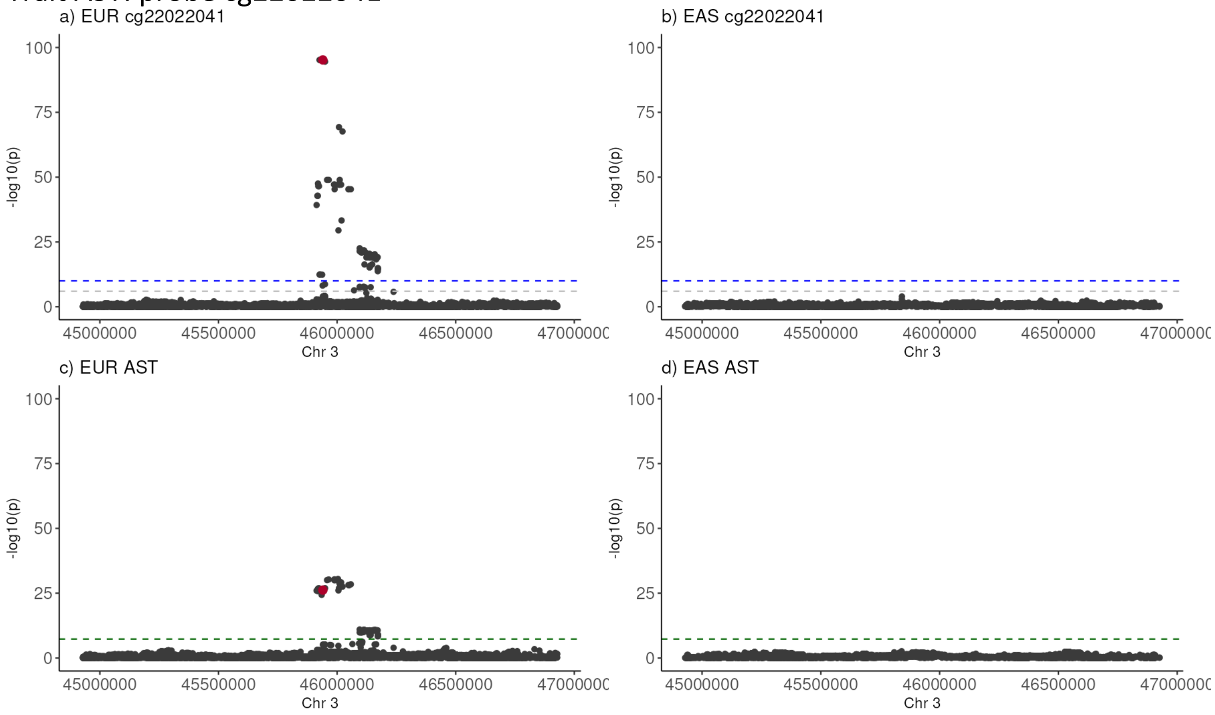

Trait AST: probe cg15742123

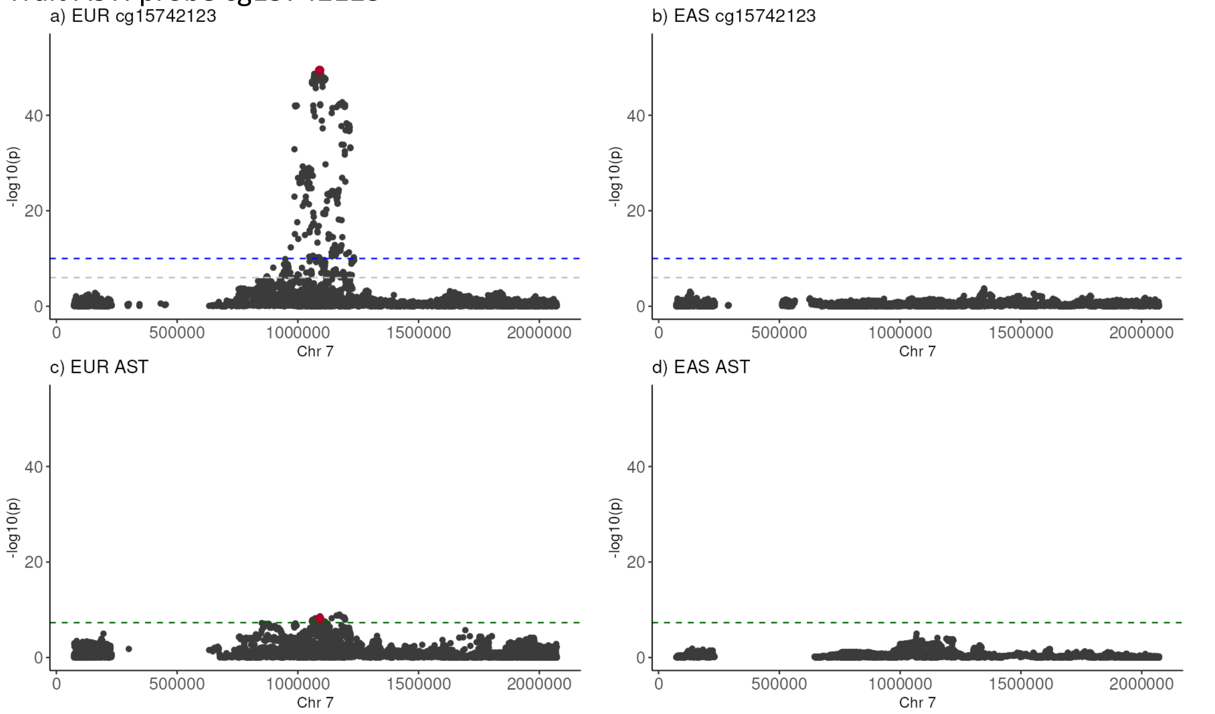

Trait AST: probe cg21664854

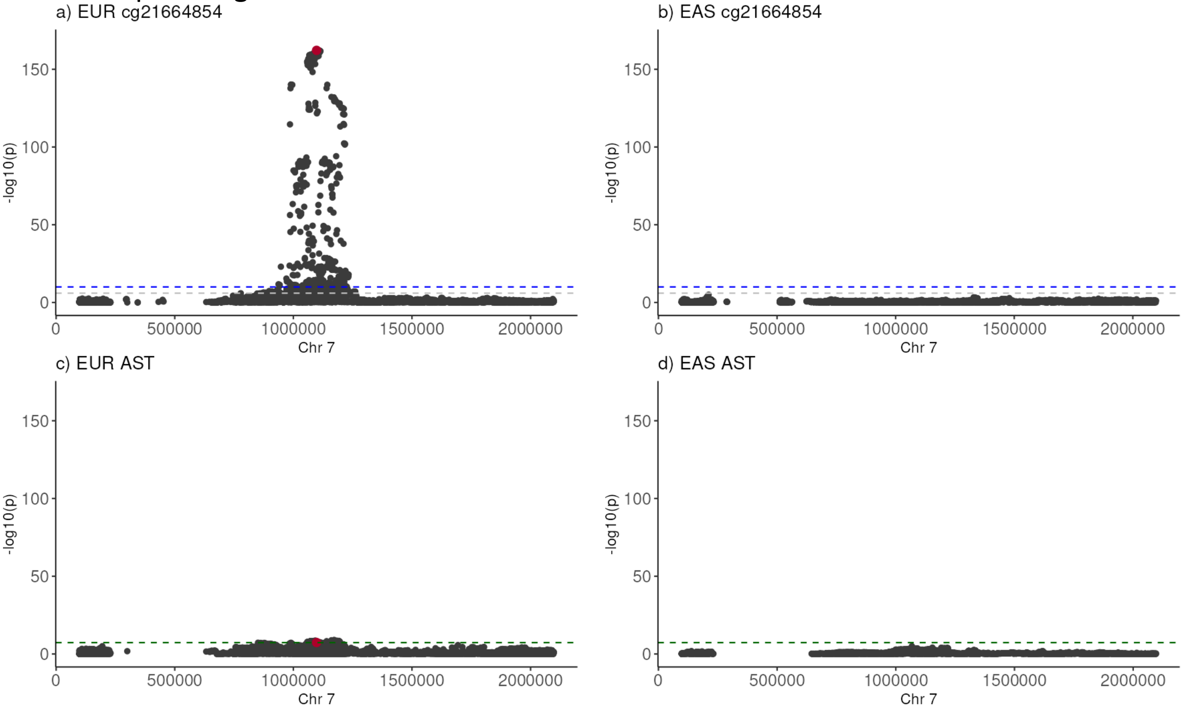

Trait AST: probe cg25863070

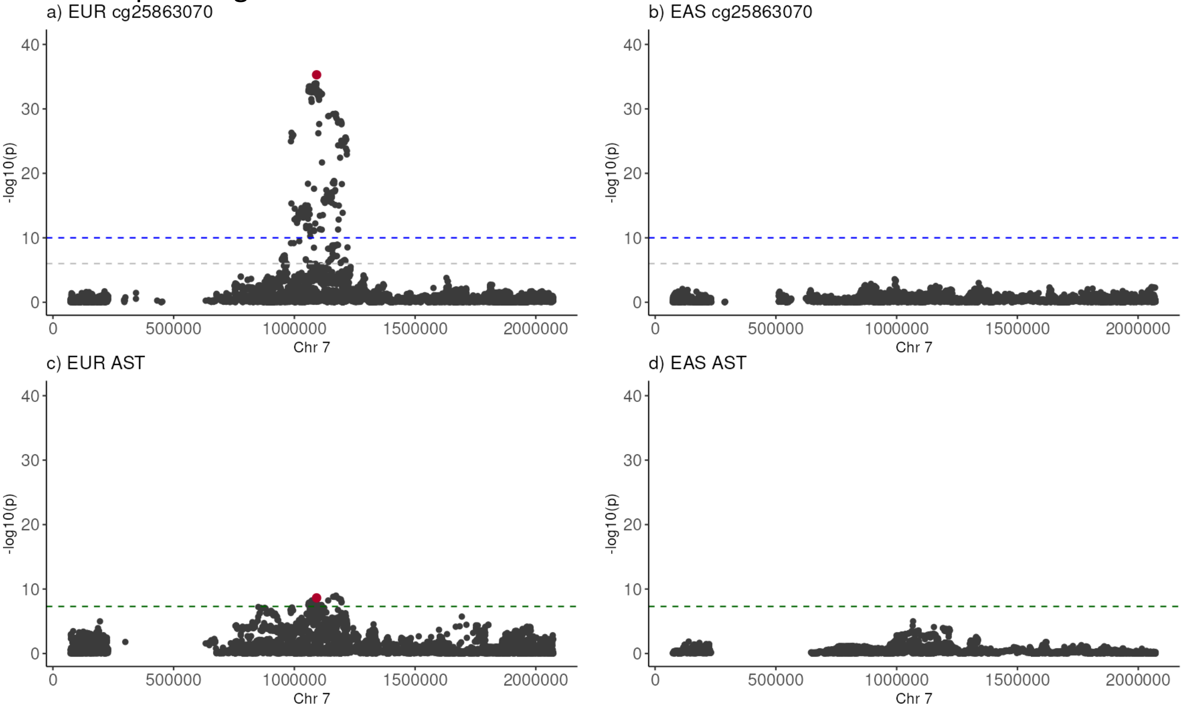

Trait AST: probe cg03058187

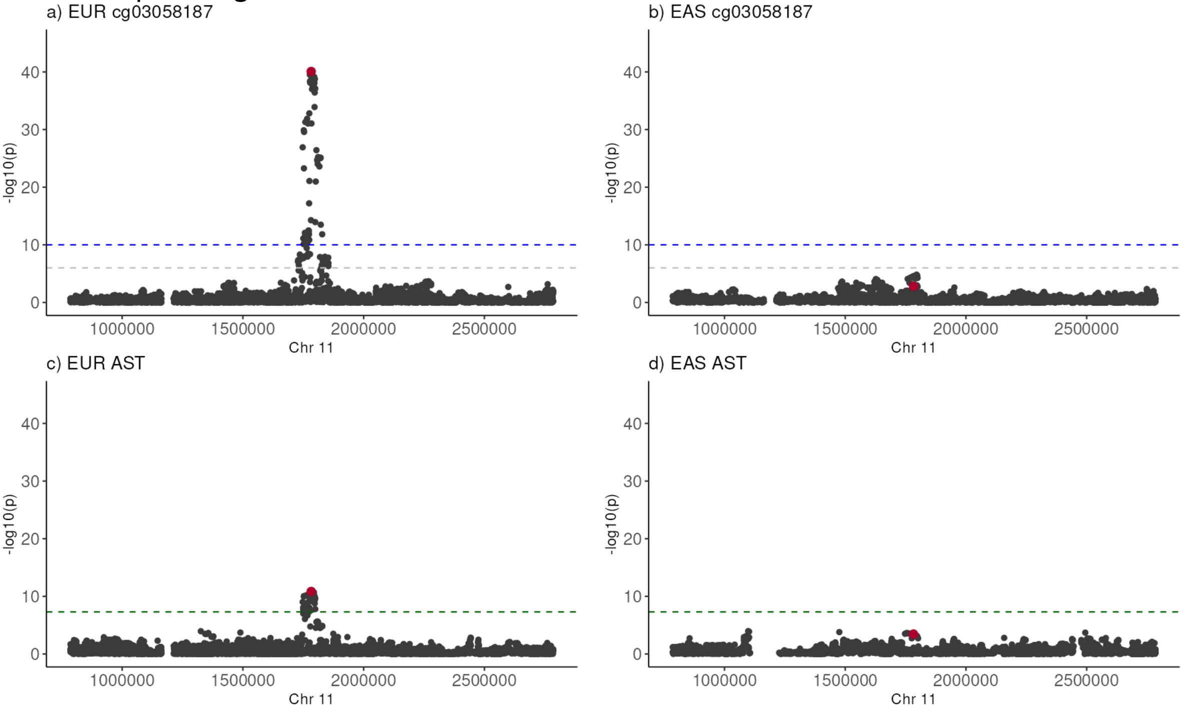

Trait AST: probe cg25044734

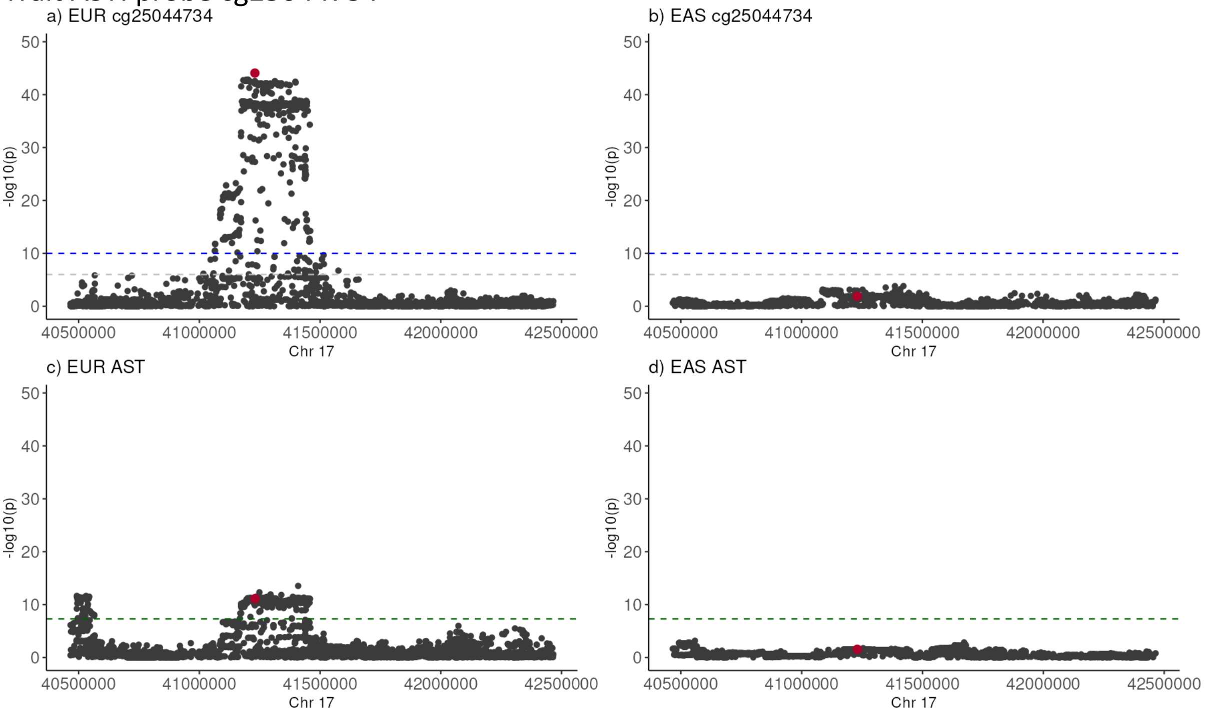

Trait AST: probe cg09479818

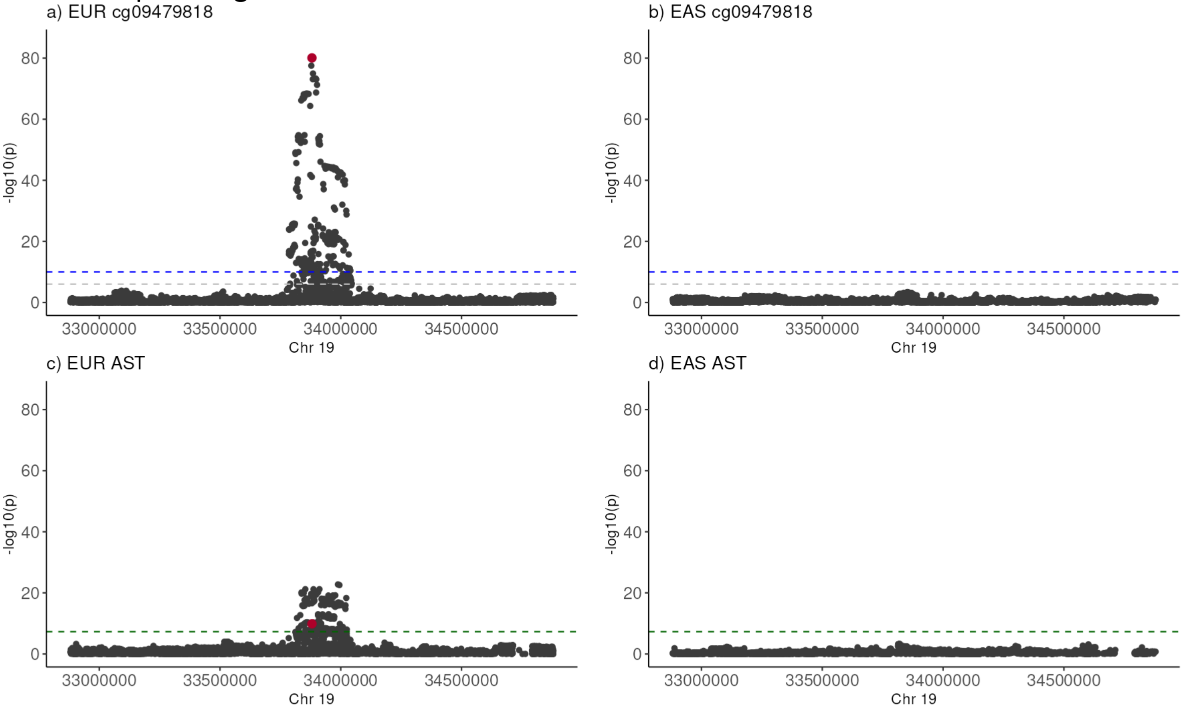

Trait BC: probe cg10900703

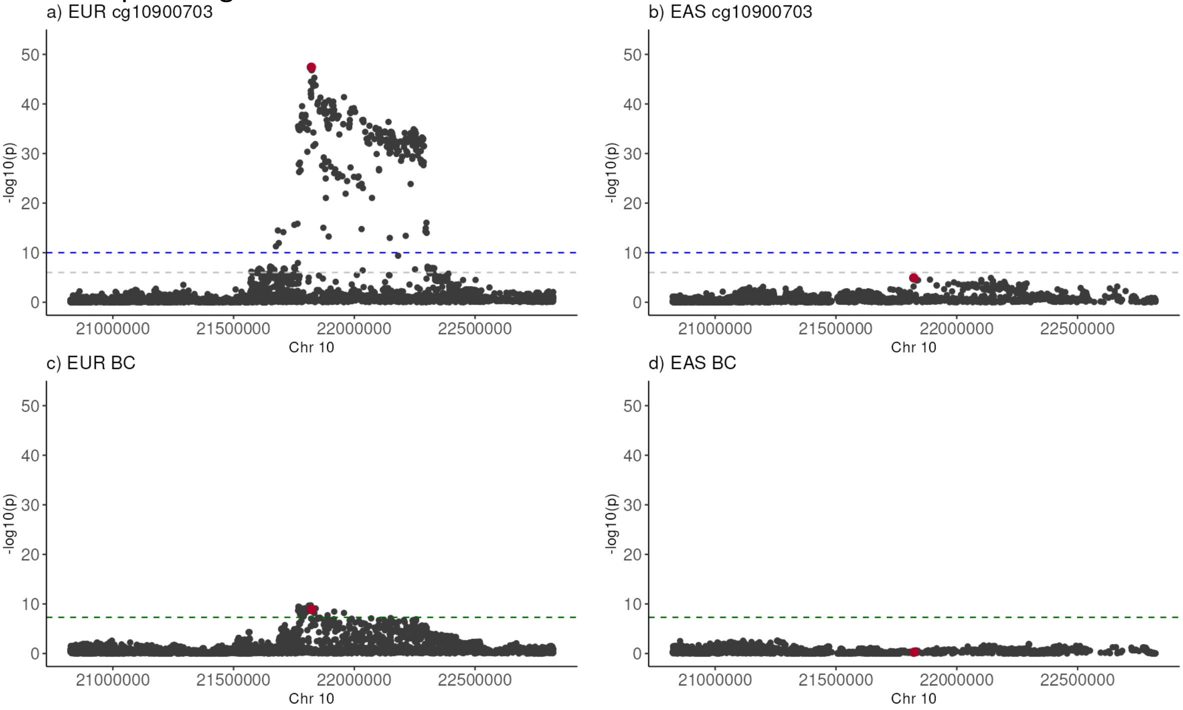

Trait BC: probe cg16440331

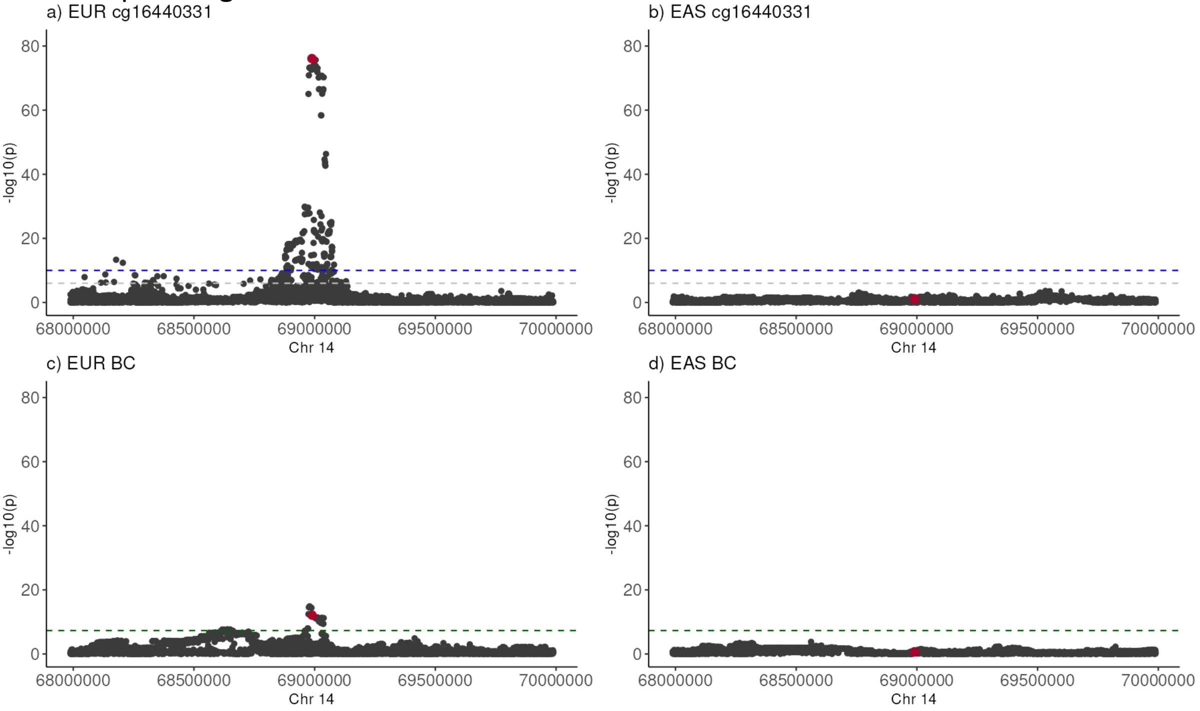

Trait BC: probe cg03733026

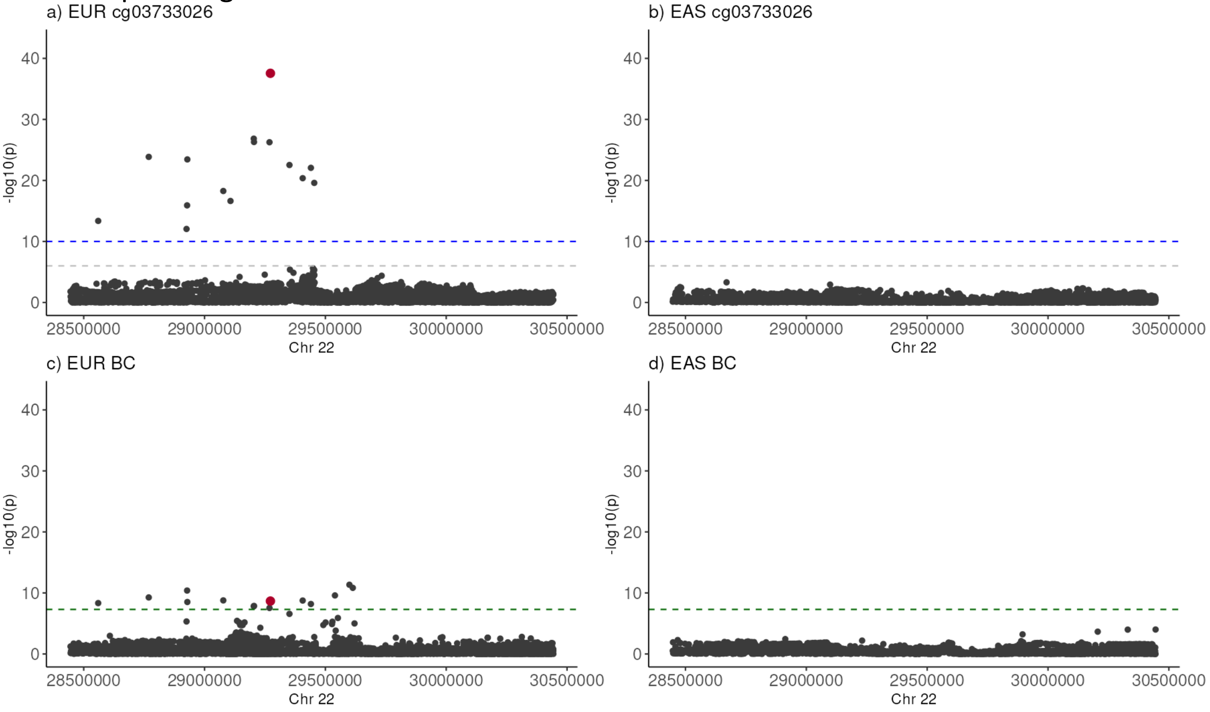

Trait BMI: probe cg05790579

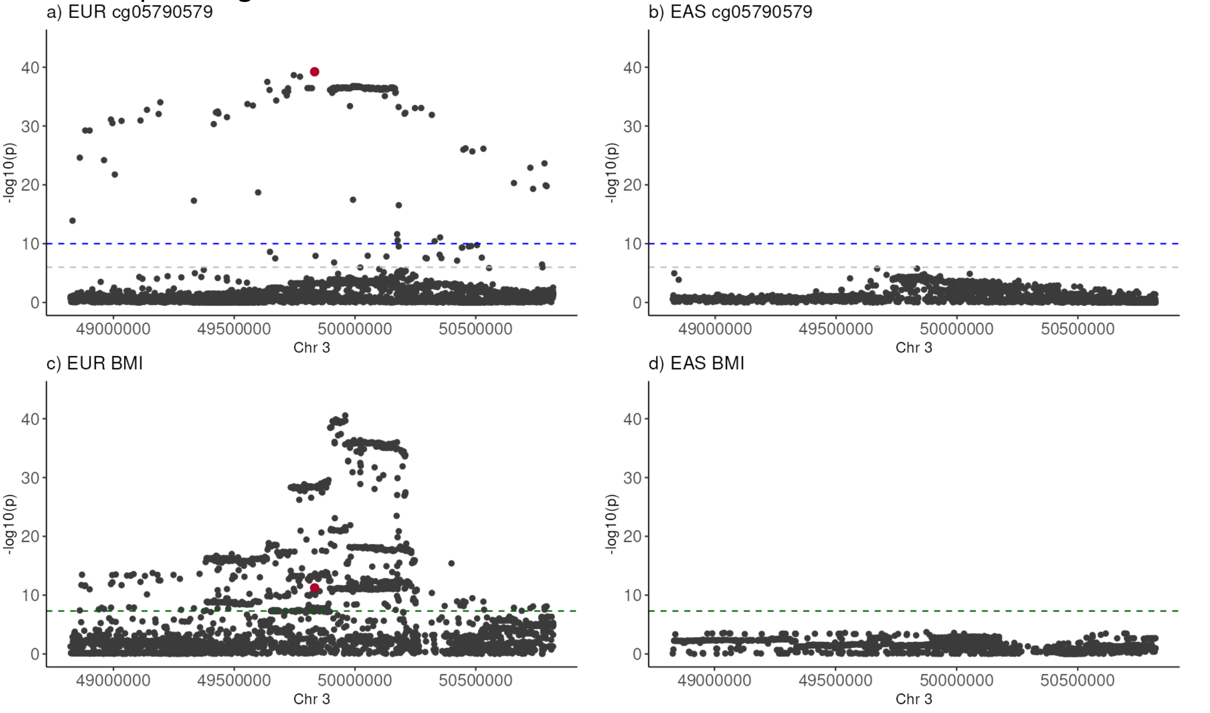

Trait BUN: probe cg16413030

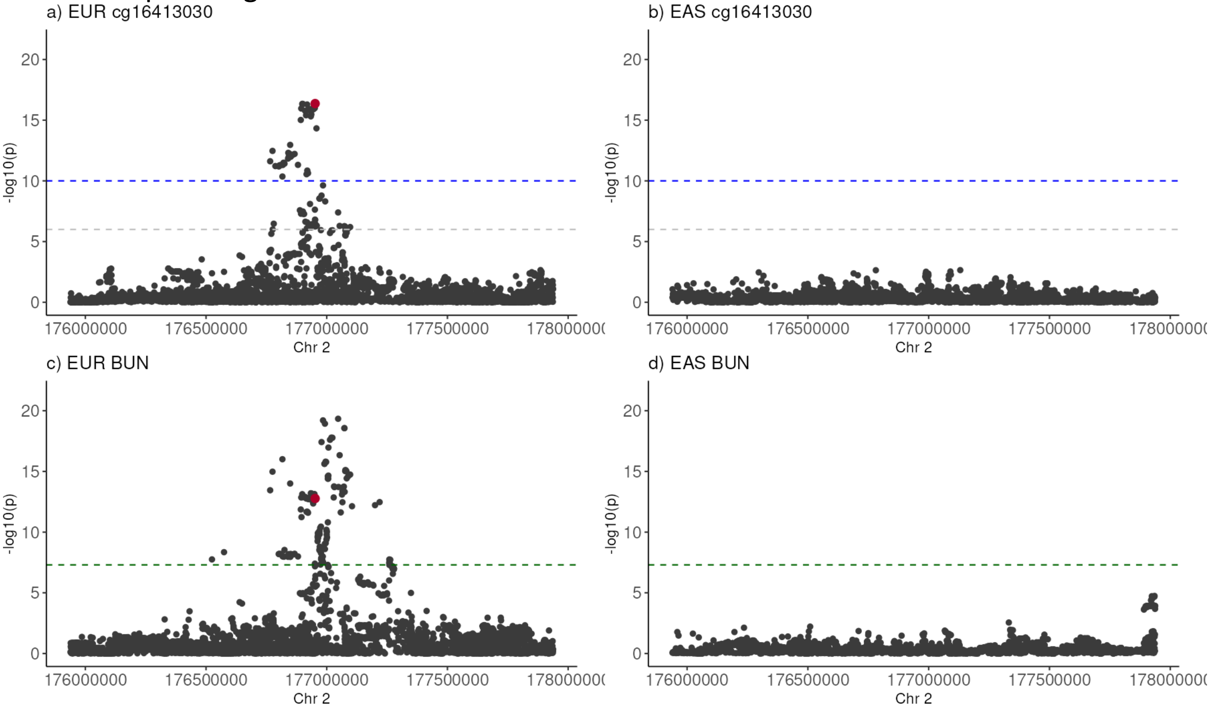

Trait BW: probe cg05845840

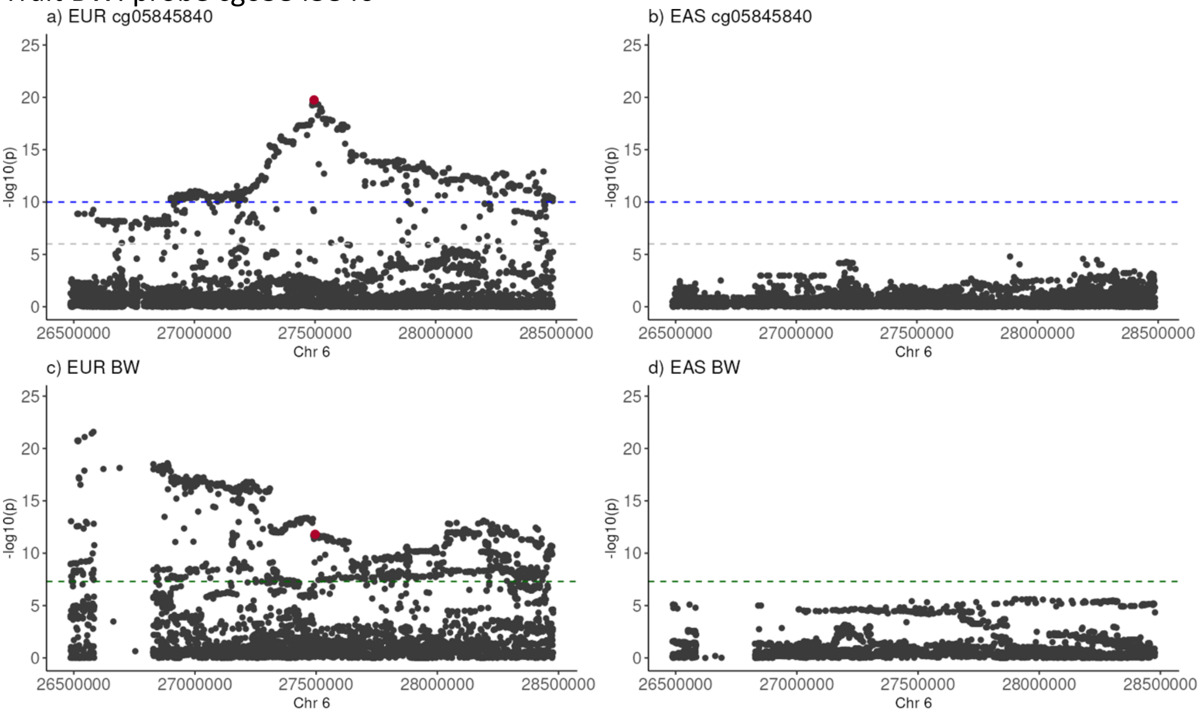

Trait BW: probe cg15325629

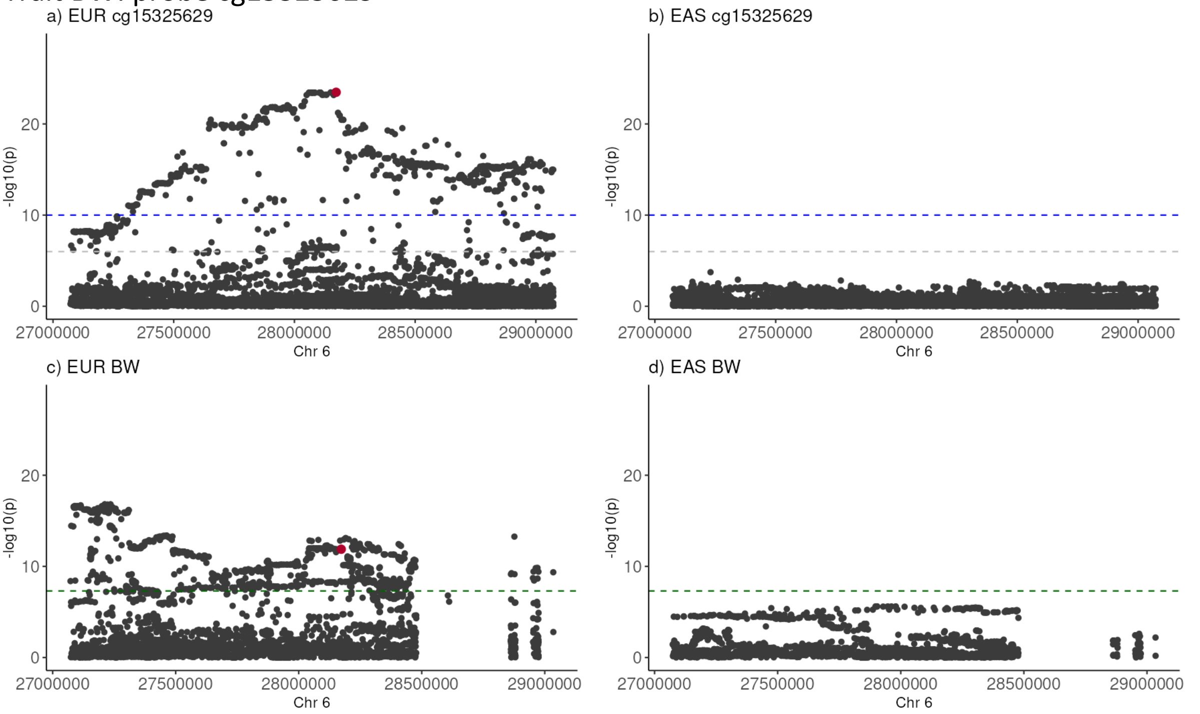

Trait BW: probe cg15967709

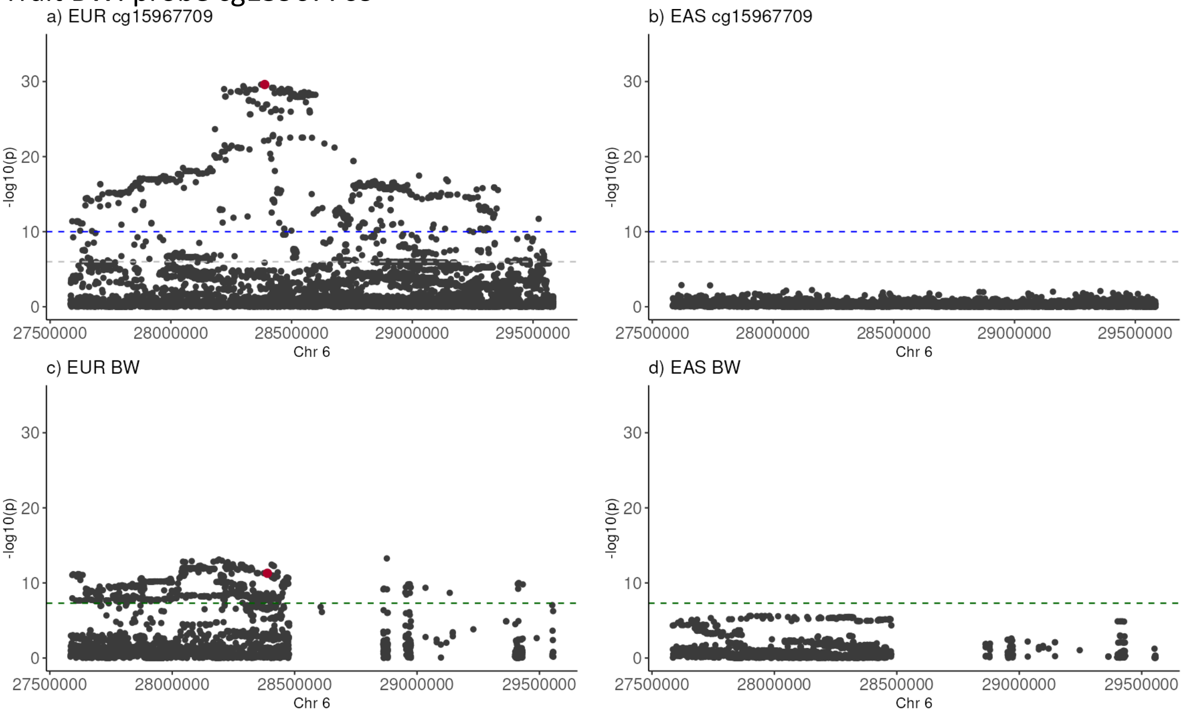

Trait BW: probe cg22087367

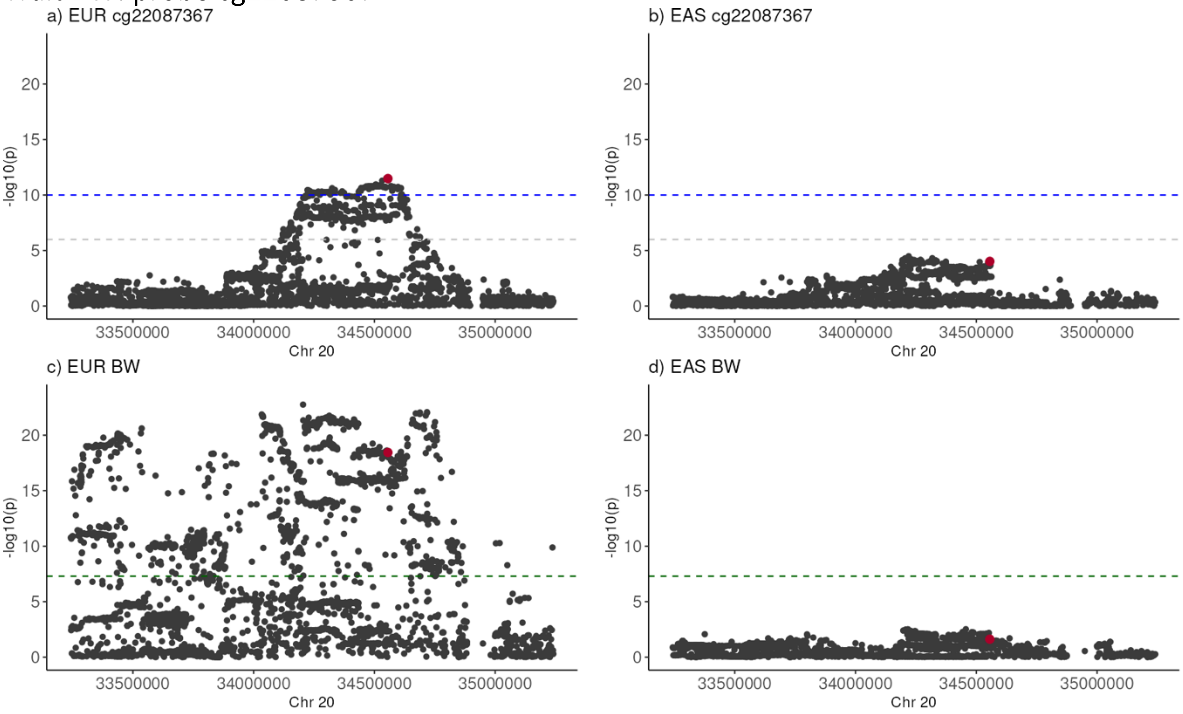

Trait C03: probe cg06068085

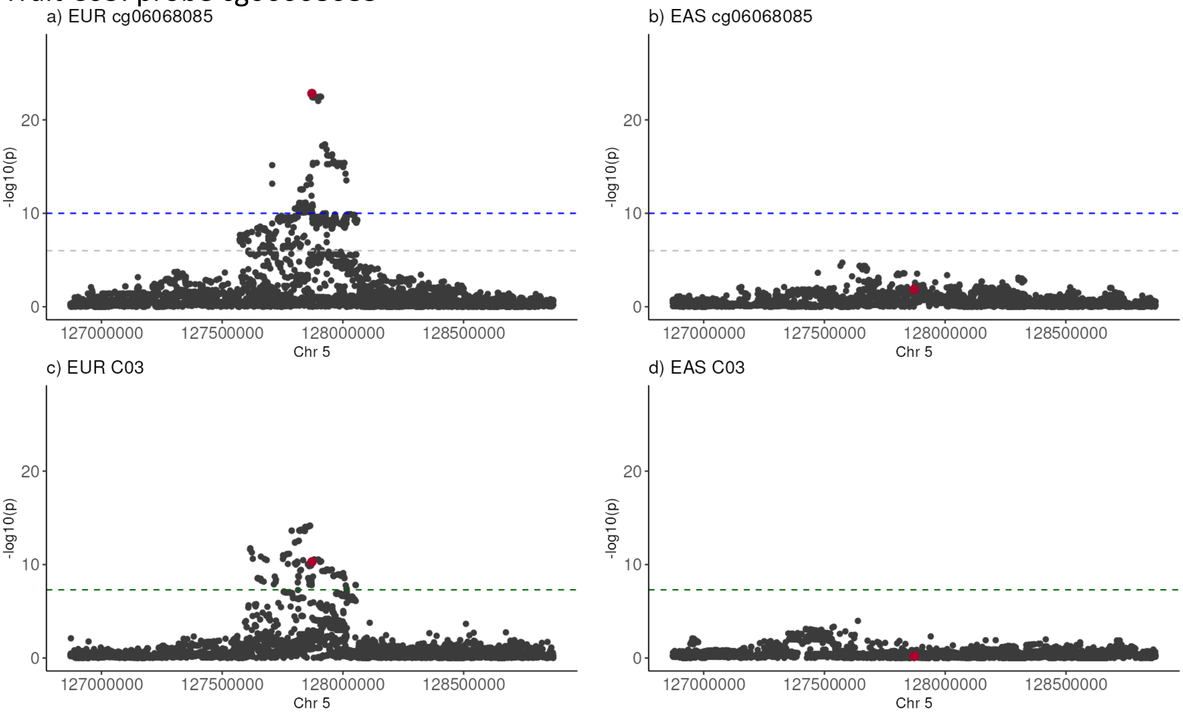

Trait C07: probe cg06068085

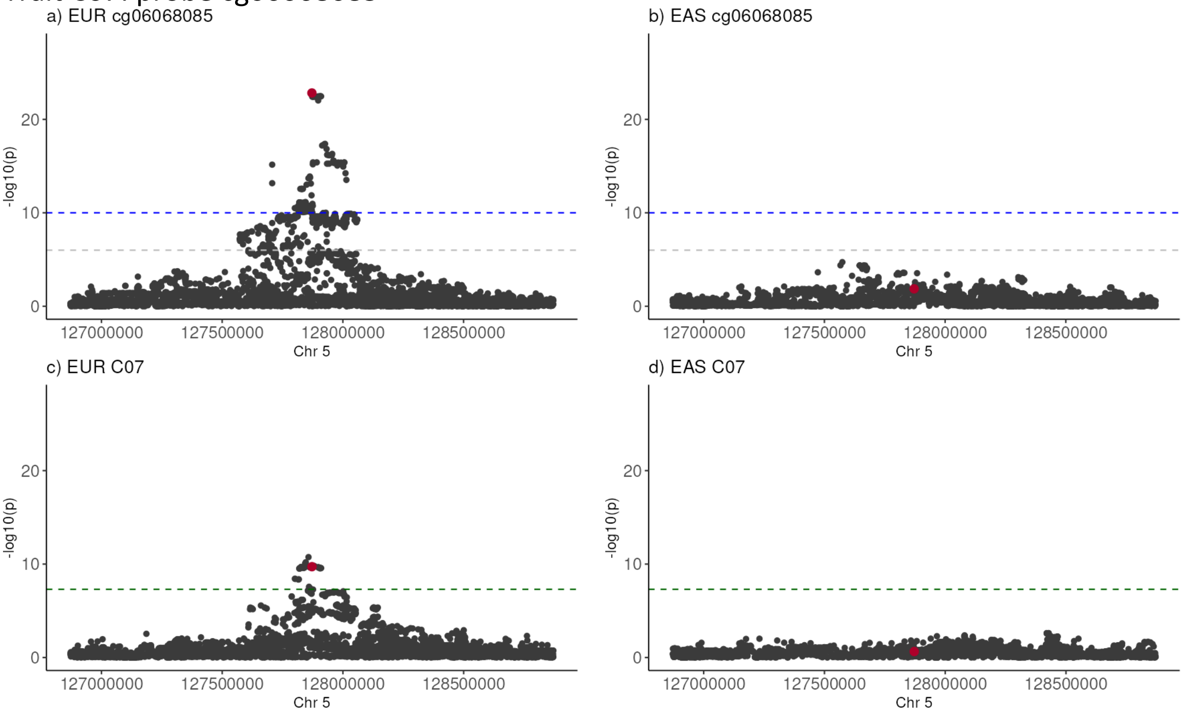

Trait C08: probe cg06068085

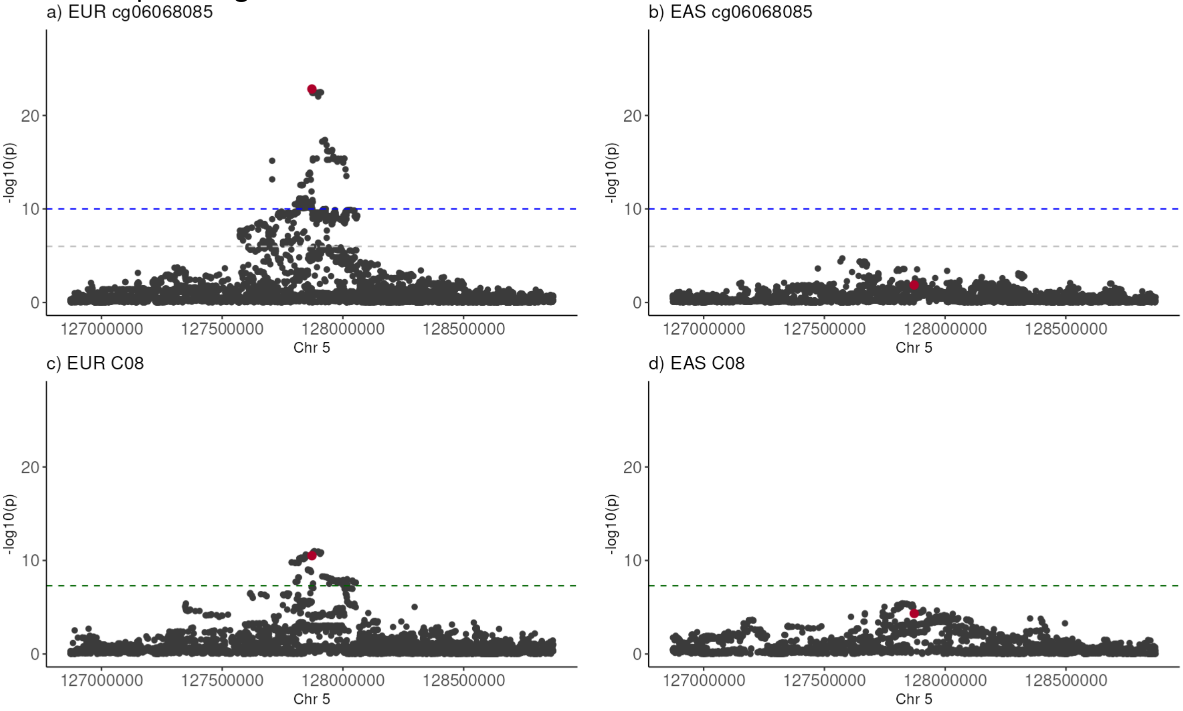

Trait C09: probe cg06068085

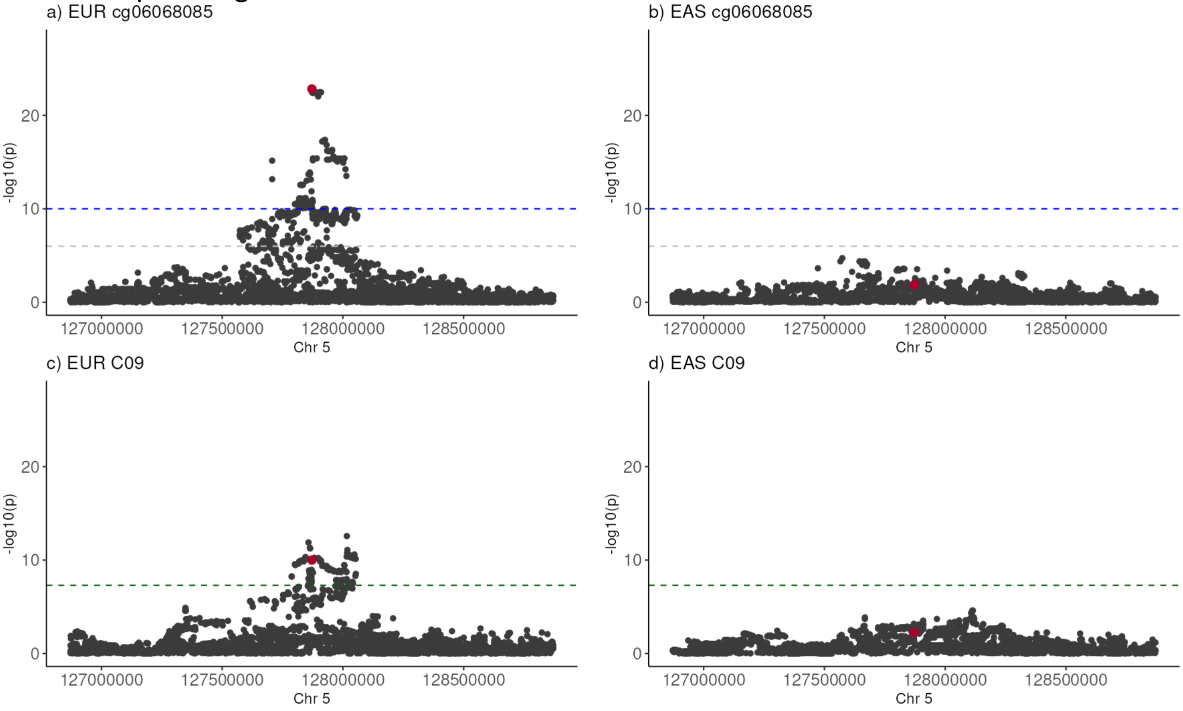

Trait C09: probe cg01046511

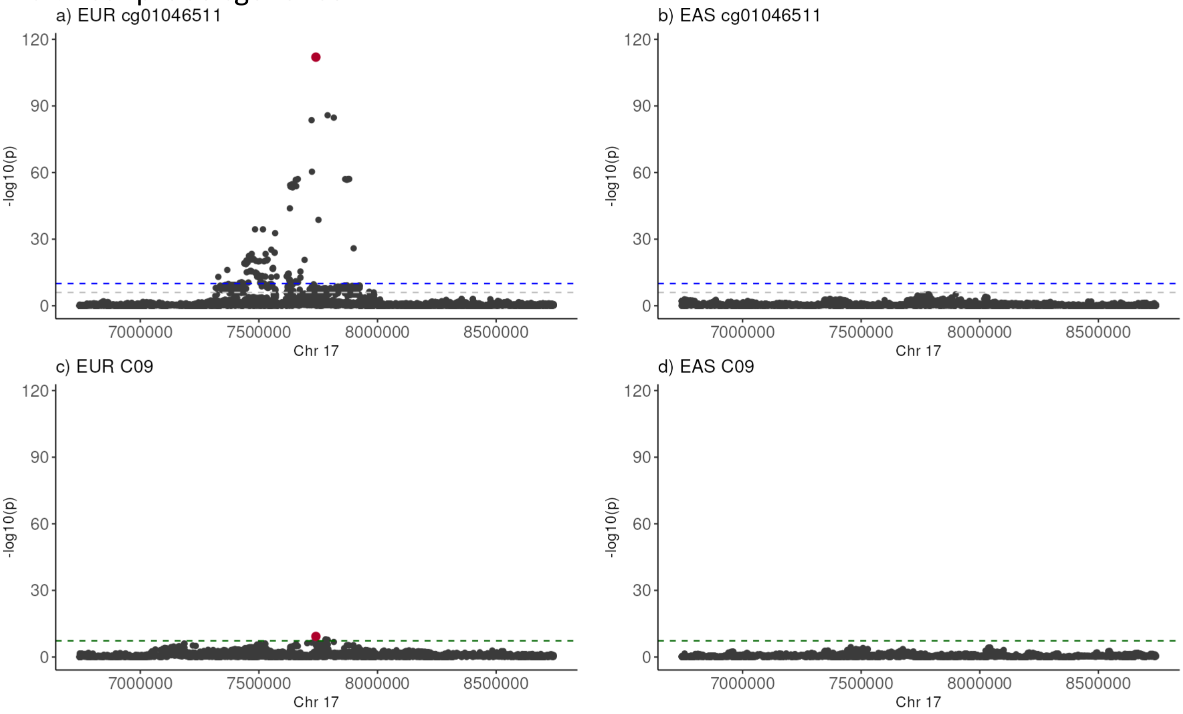

Trait C09: probe cg01656853

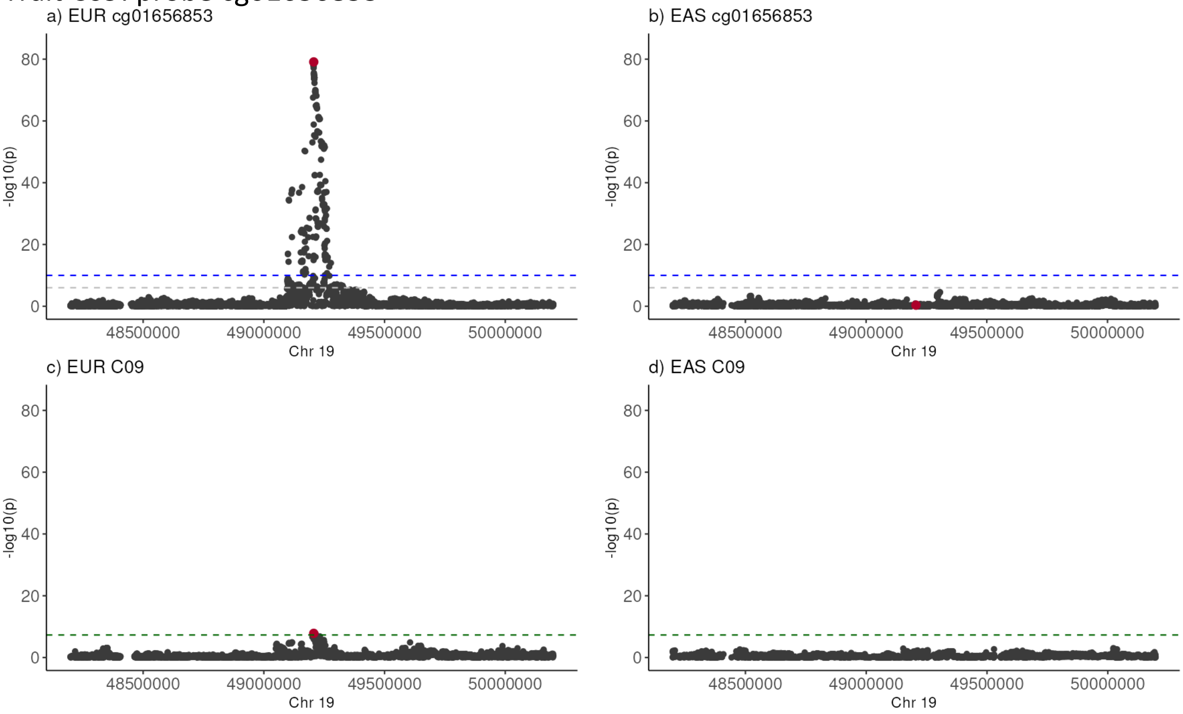

Trait C09: probe cg08873673

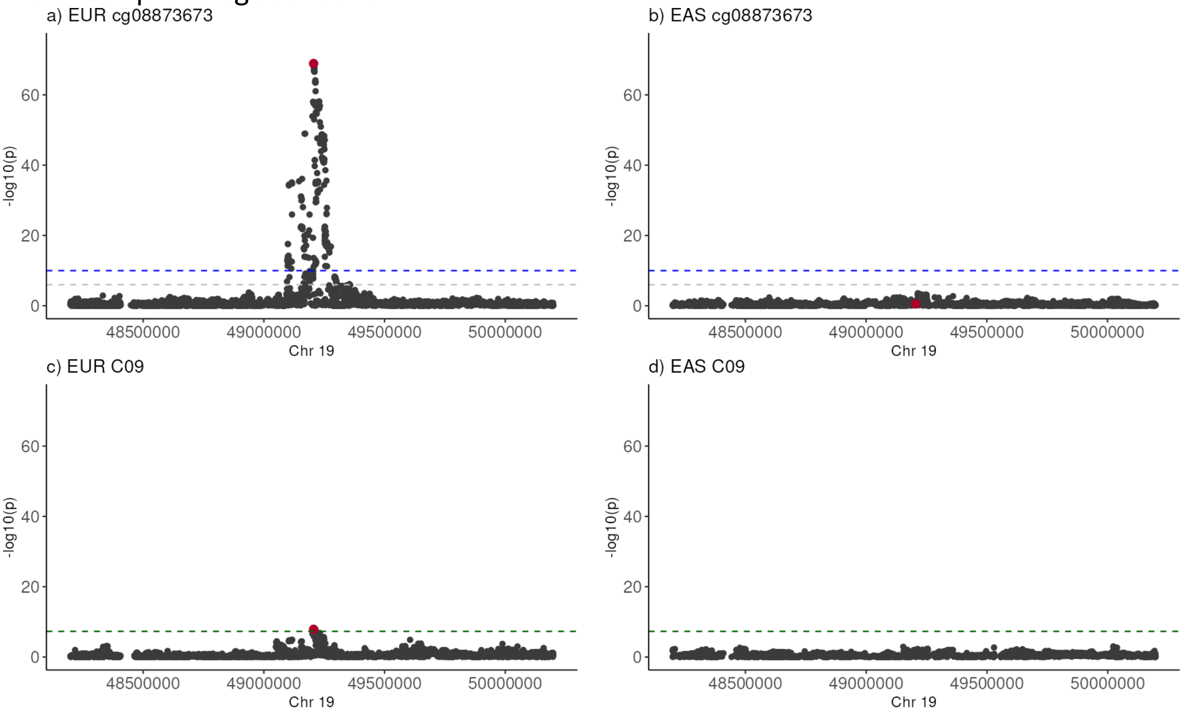

Trait C10AA: probe cg01656853

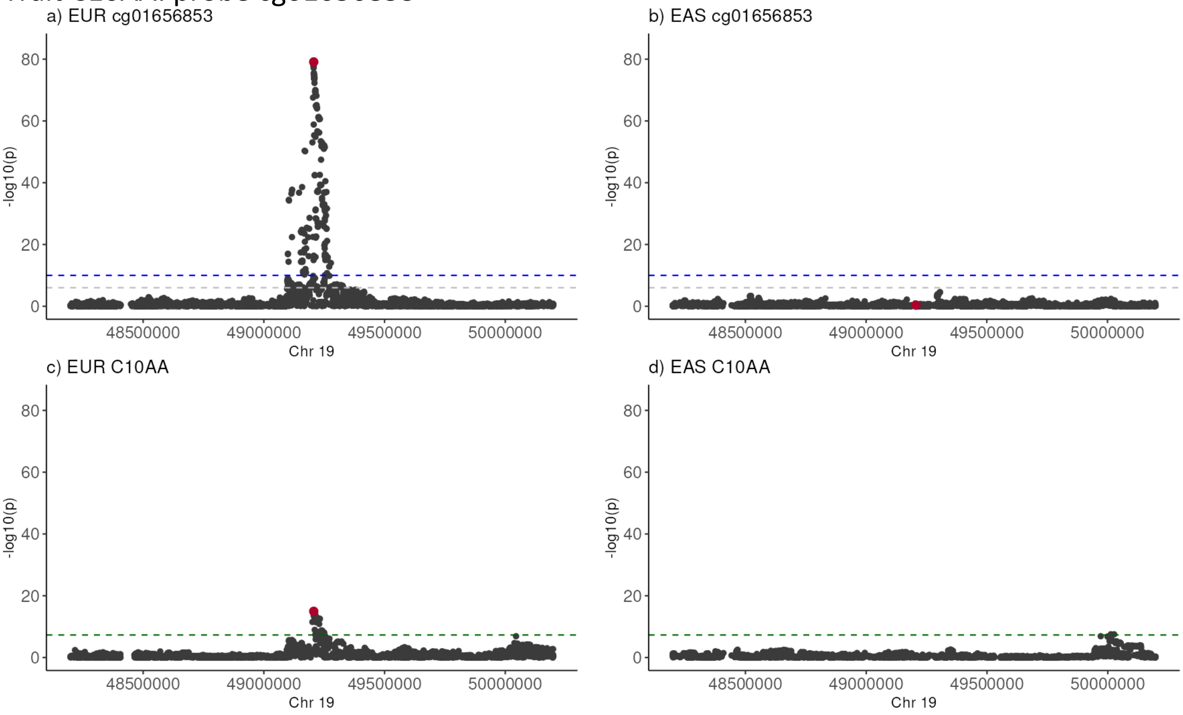

Trait C10AA: probe cg08873673

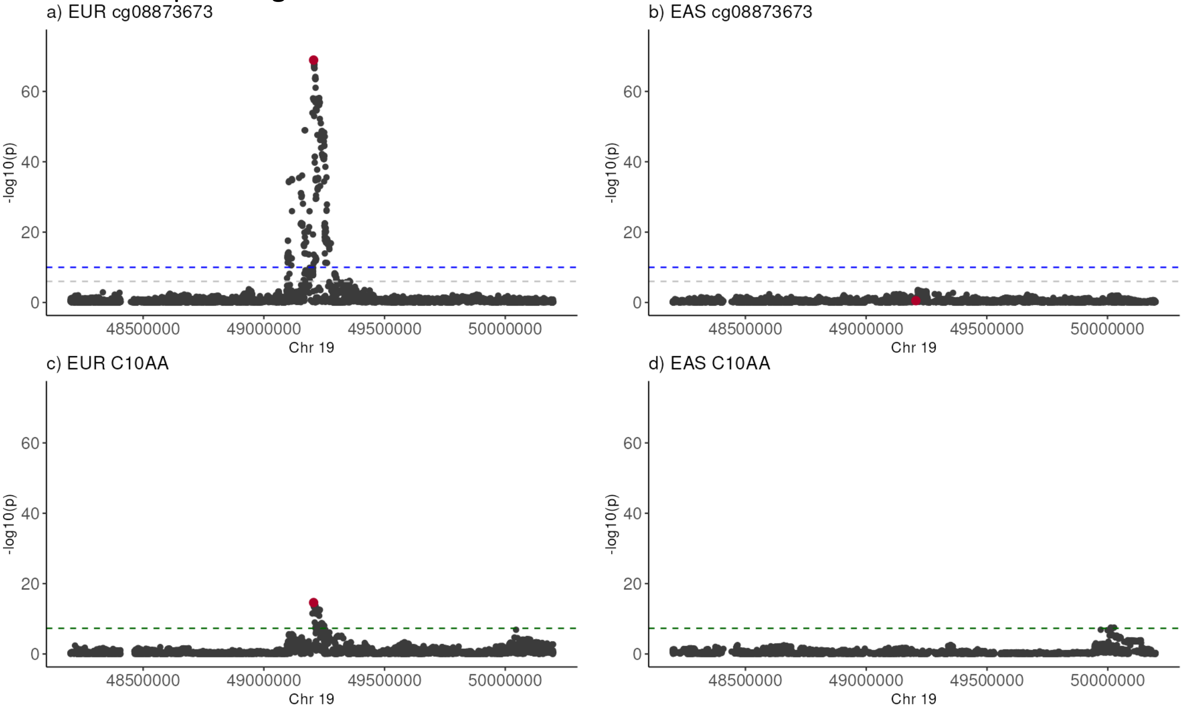

Trait C10AA: probe cg20441902

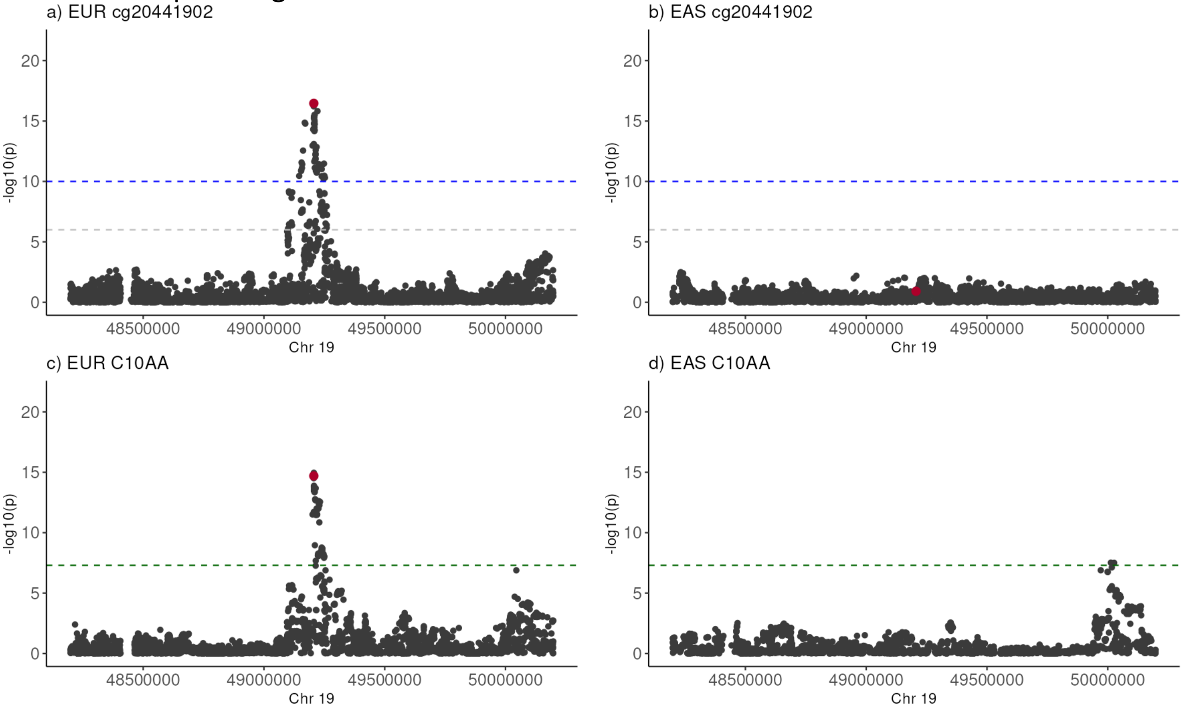

Trait Ca: probe cg00228796

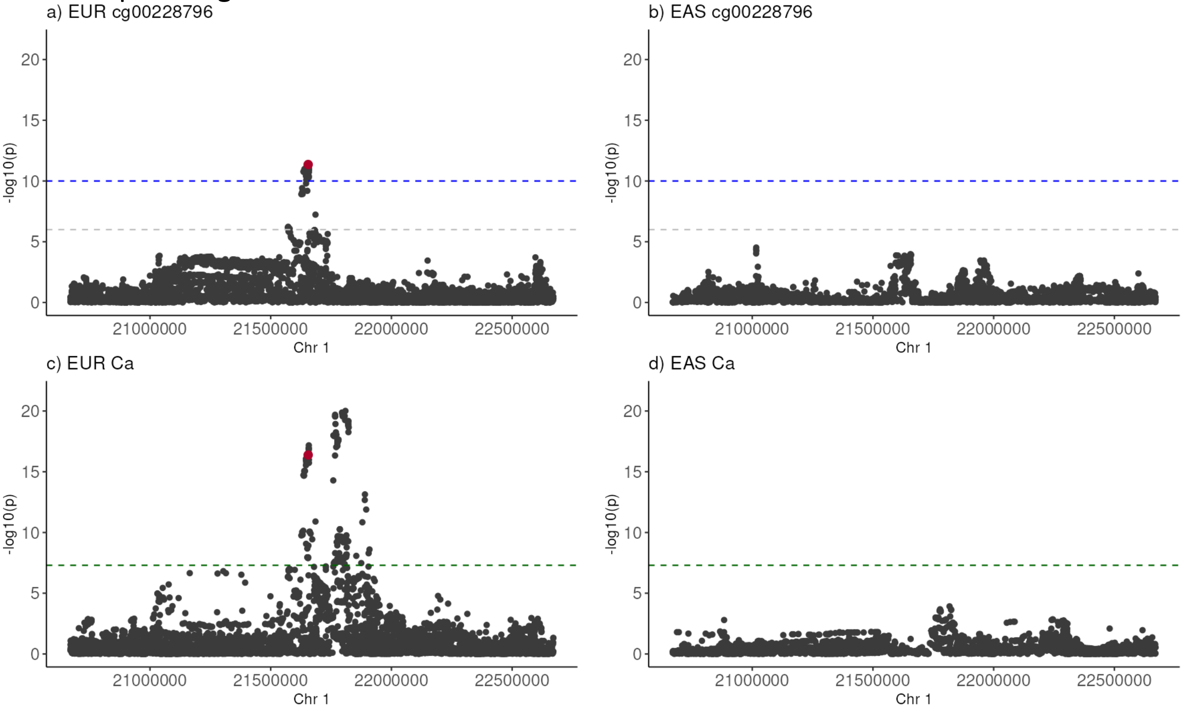

Trait Ca: probe cg03020951

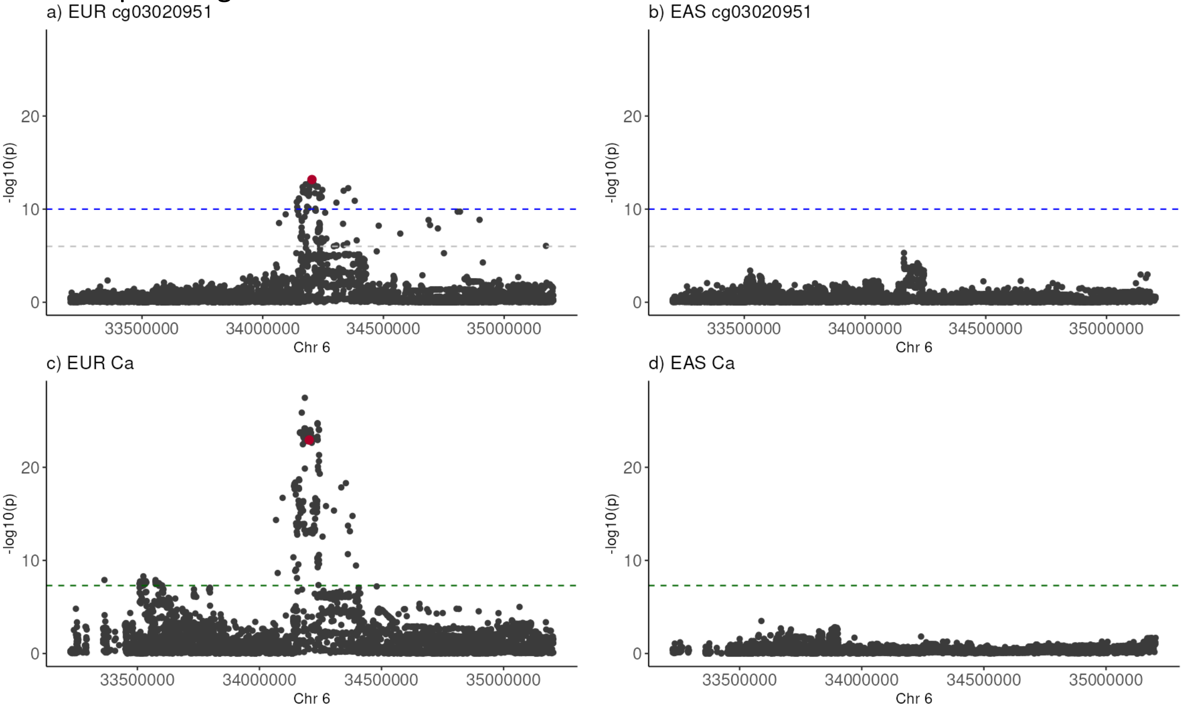

Trait Cho: probe cg01656853

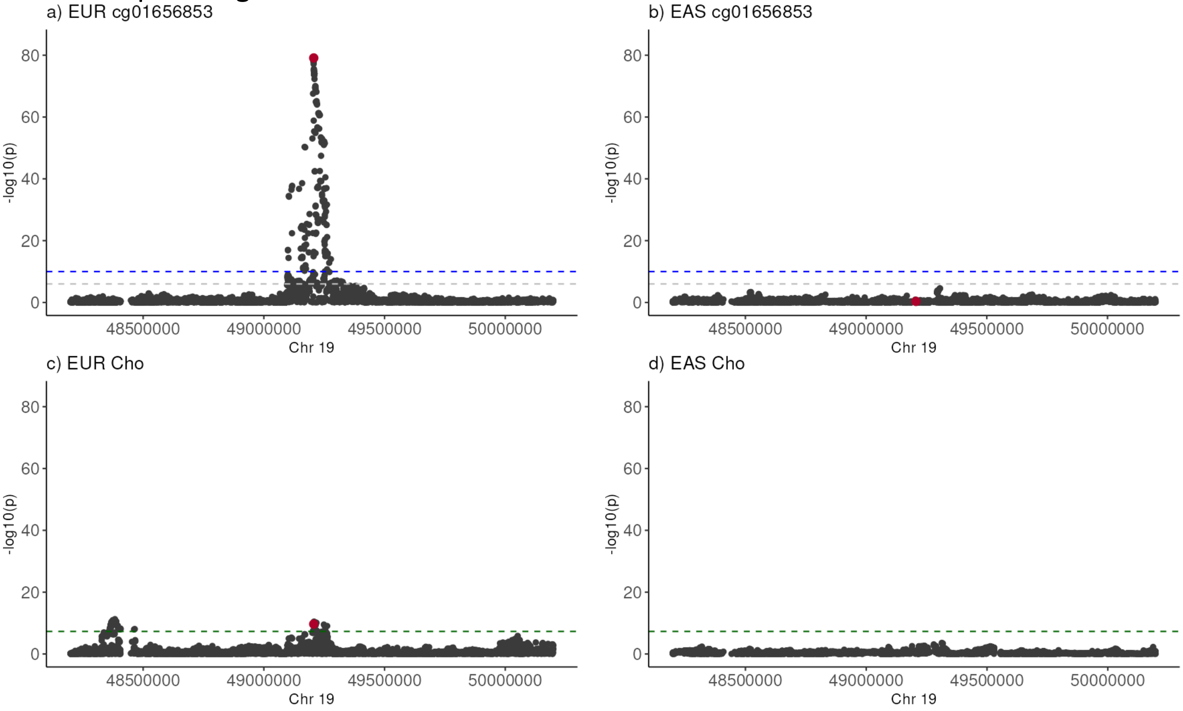

Trait Cho: probe cg08873673

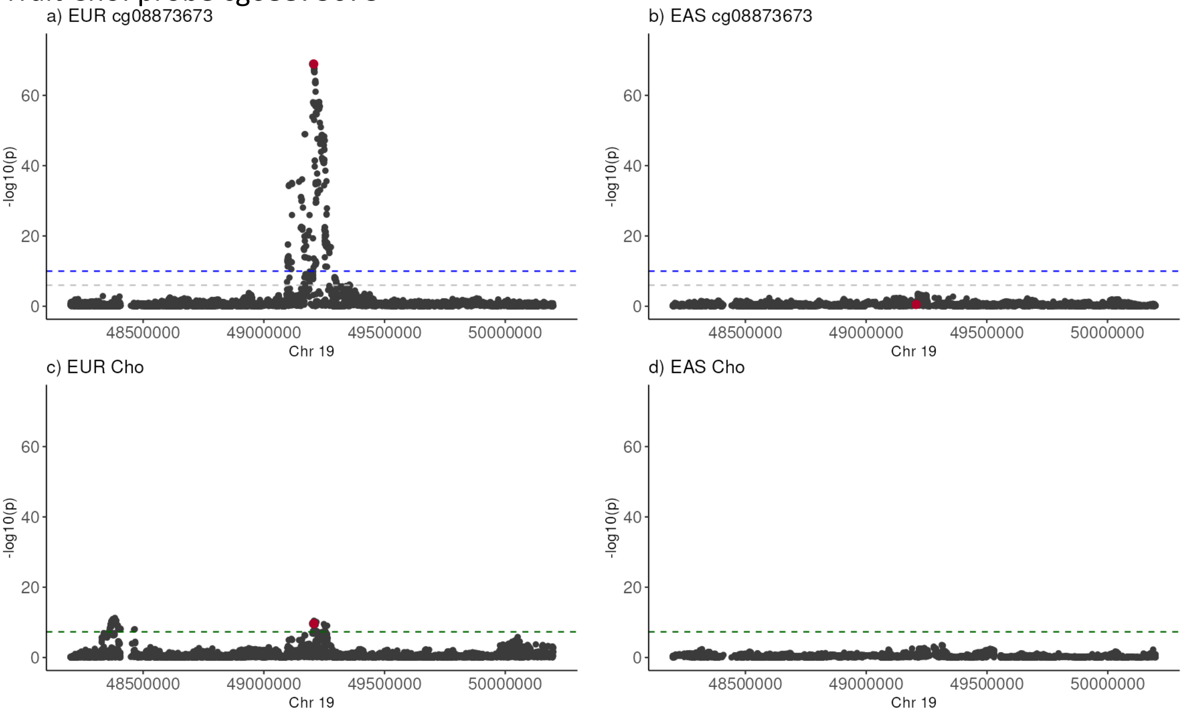

Trait CRP: probe cg23571126

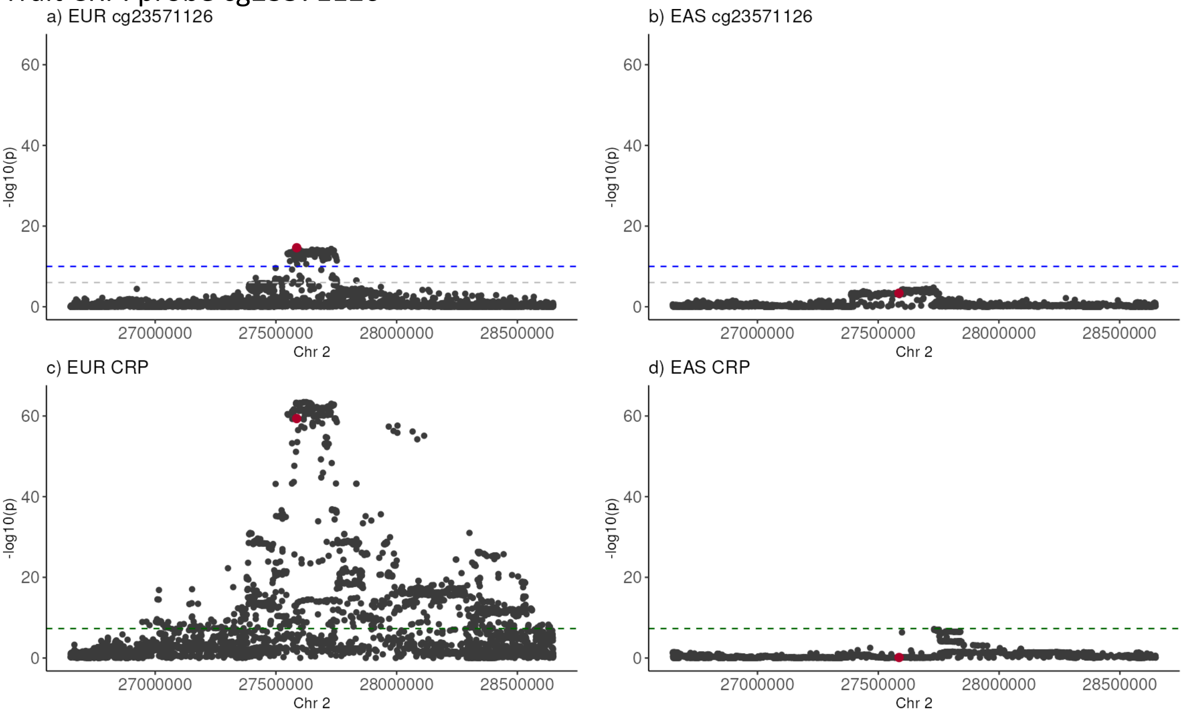

Trait CRP: probe cg09642020

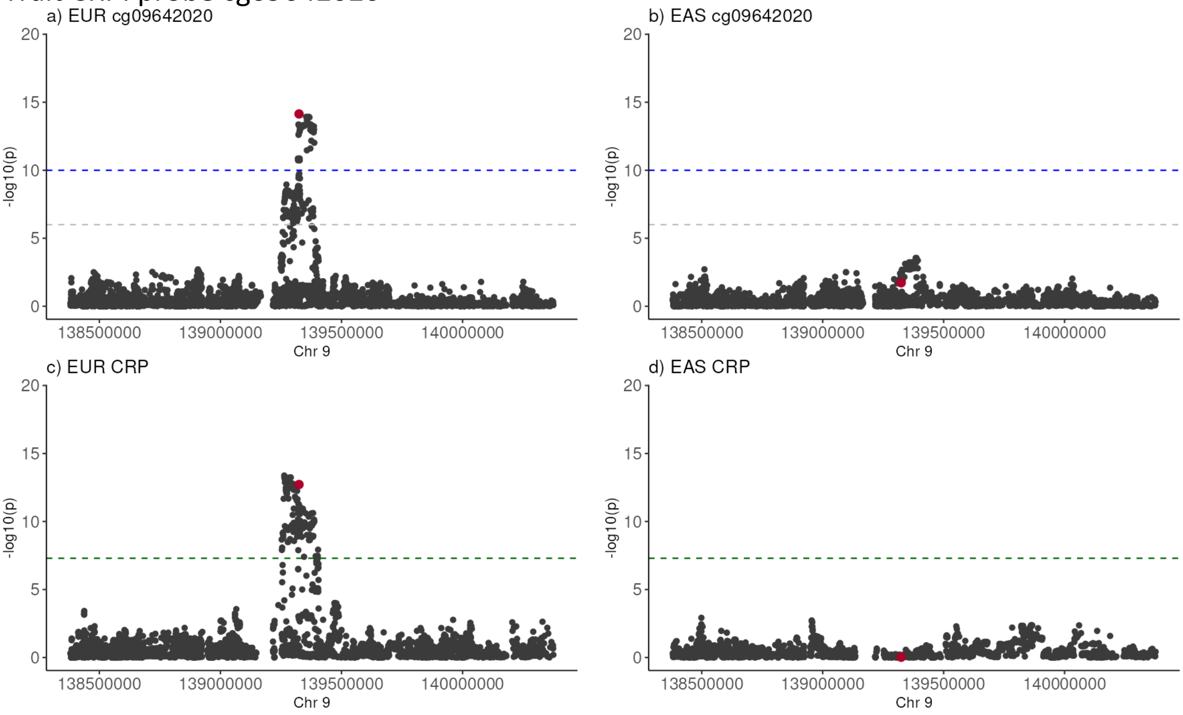

Trait DBP: probe cg22988851

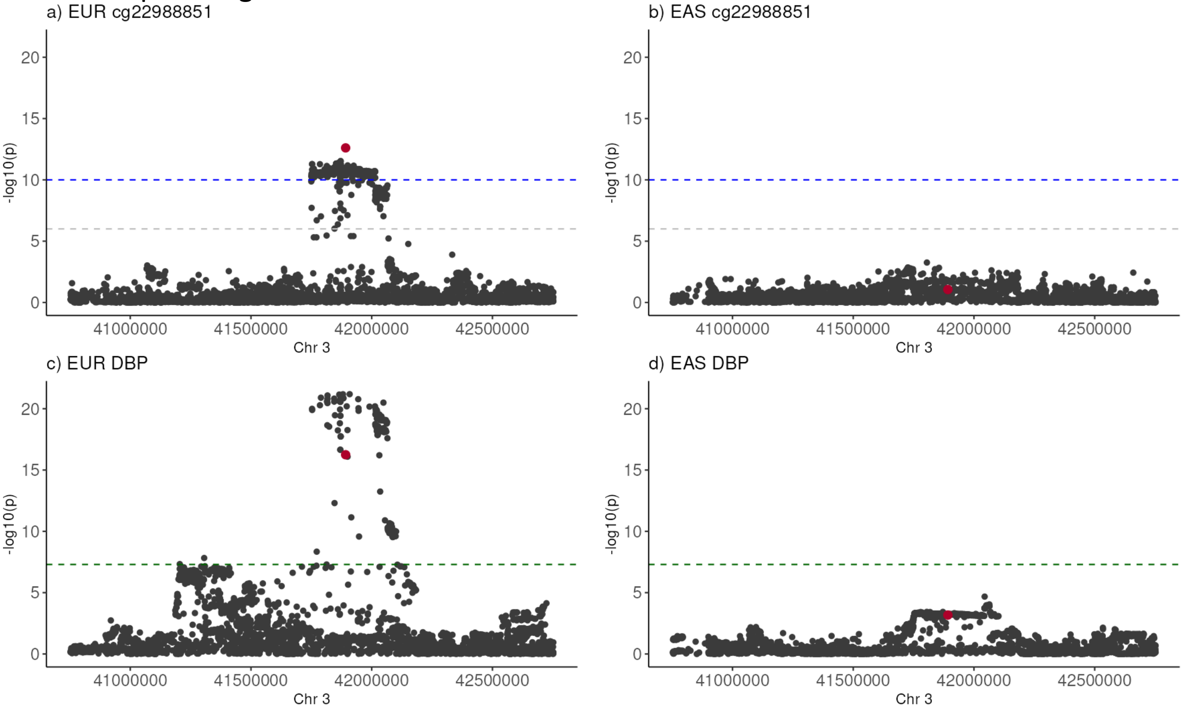

Trait DBP: probe cg21959090

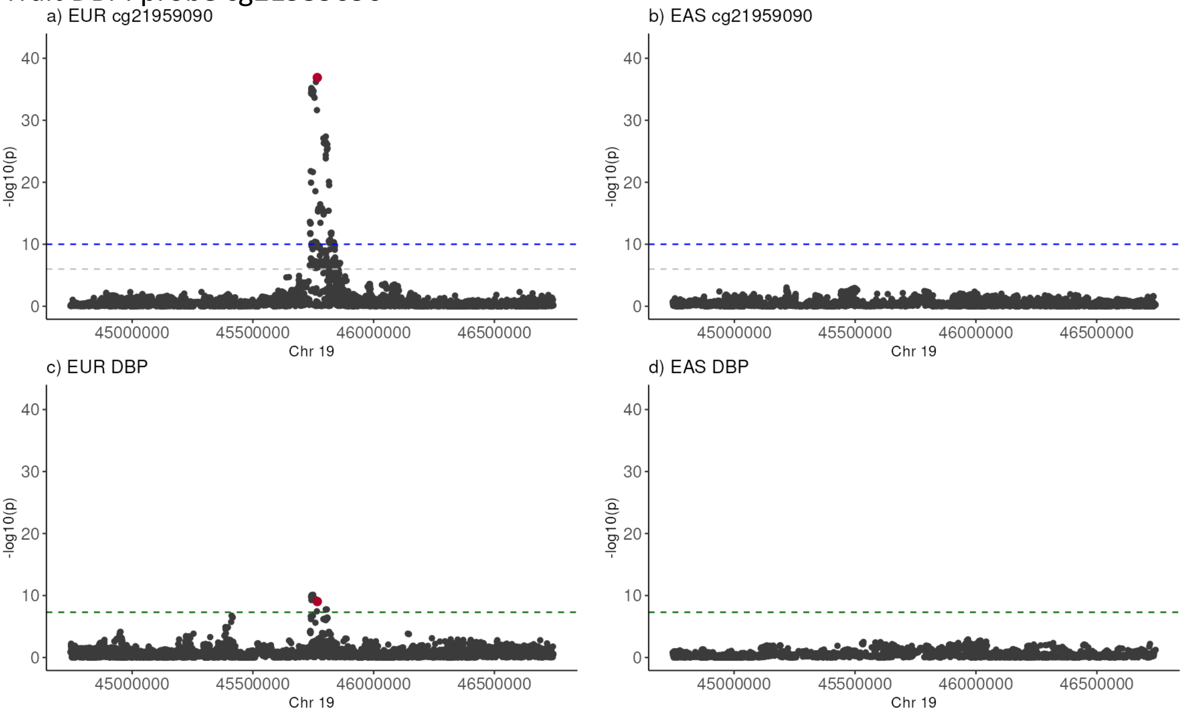

Trait Eosino: probe cg06760507

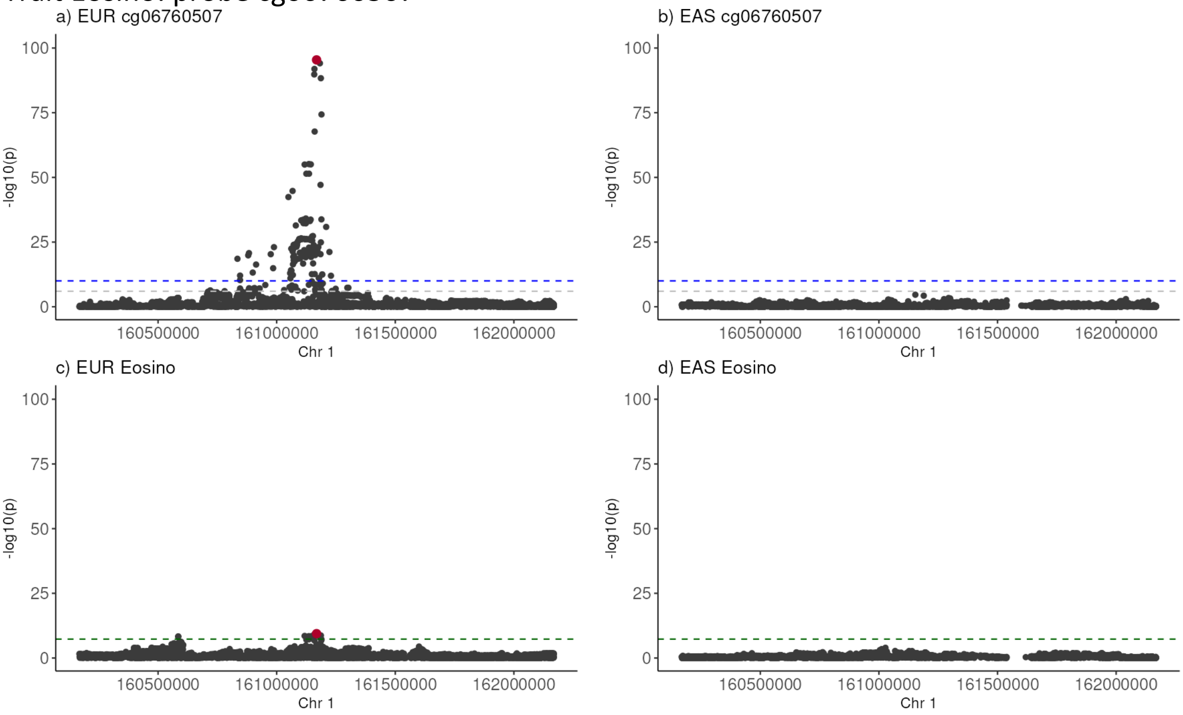

Trait Eosino: probe cg16597045

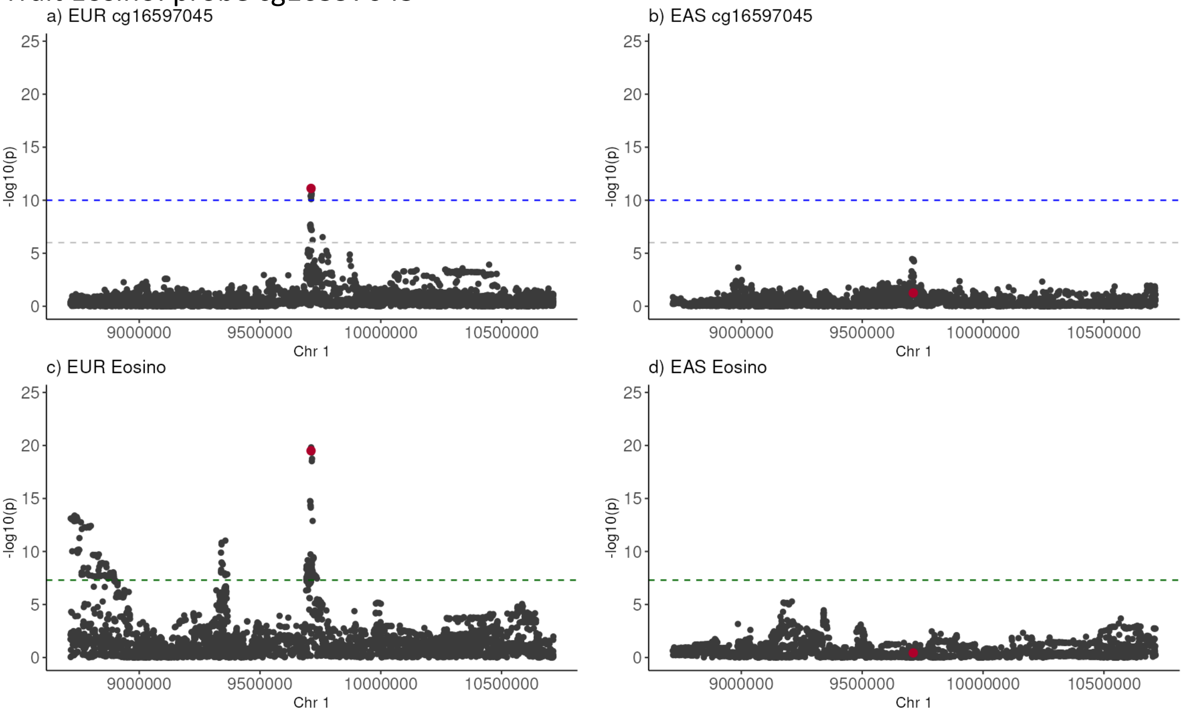

Trait Eosino: probe cg01765152

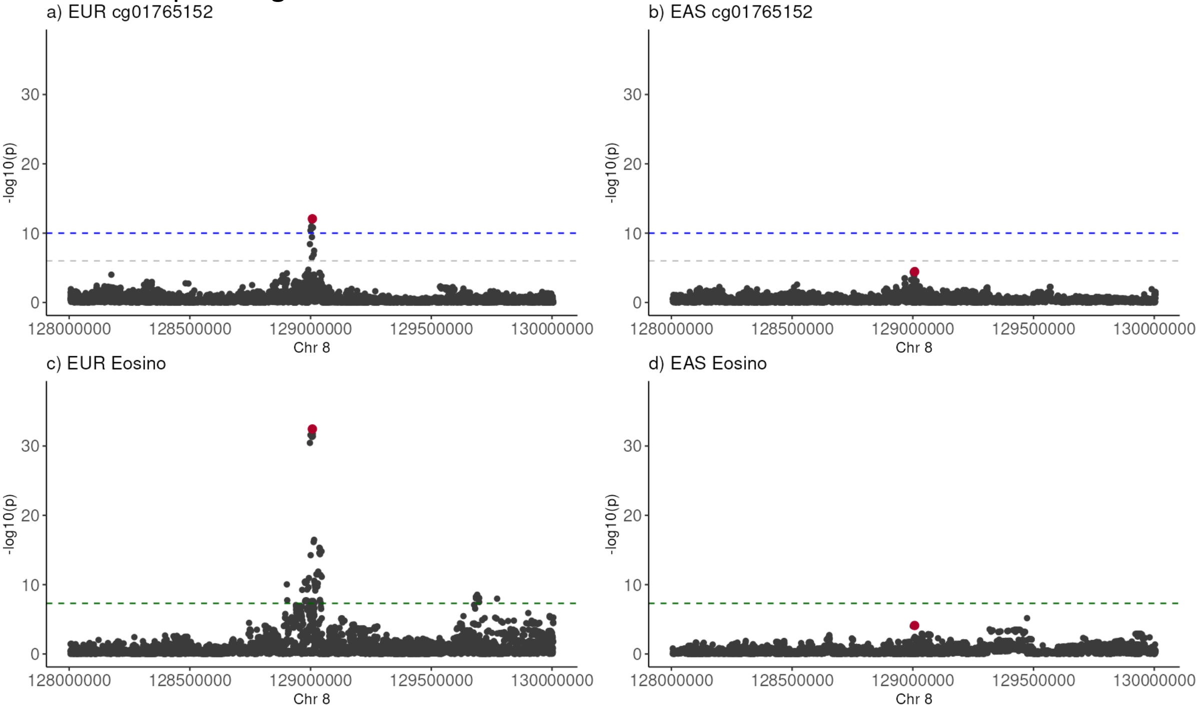

Trait Eosino: probe cg22504024

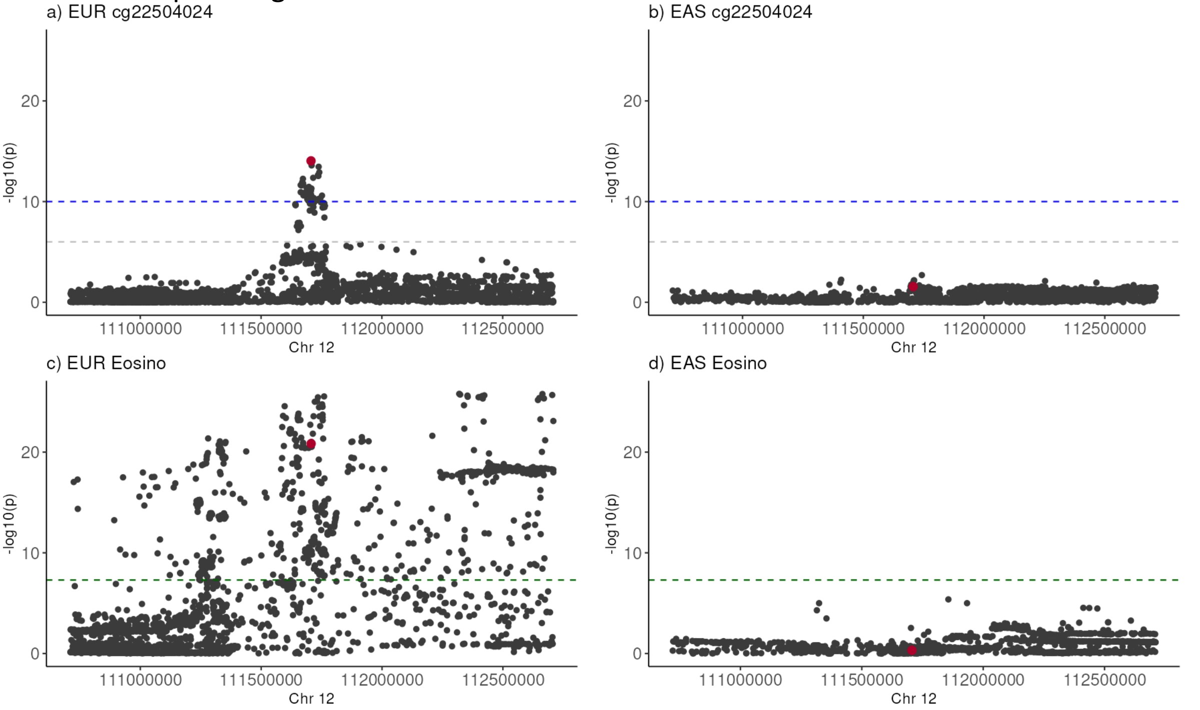

Trait Eosino: probe cg01692252

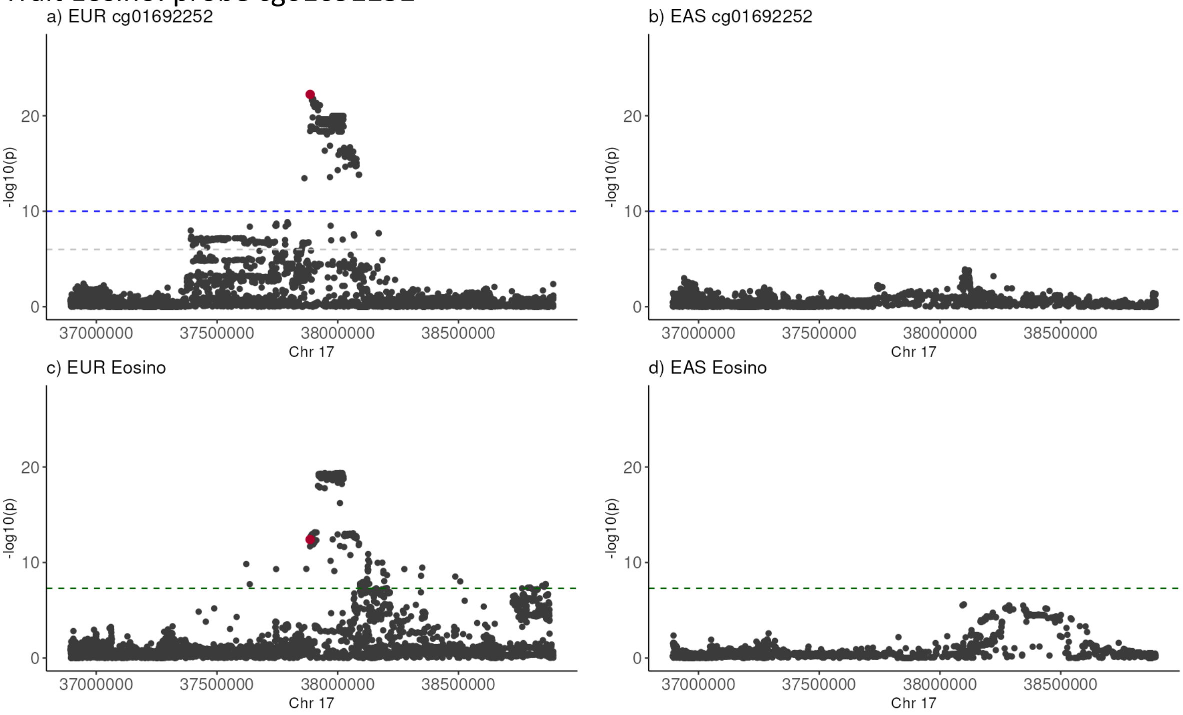

Trait Eosino: probe cg14112635

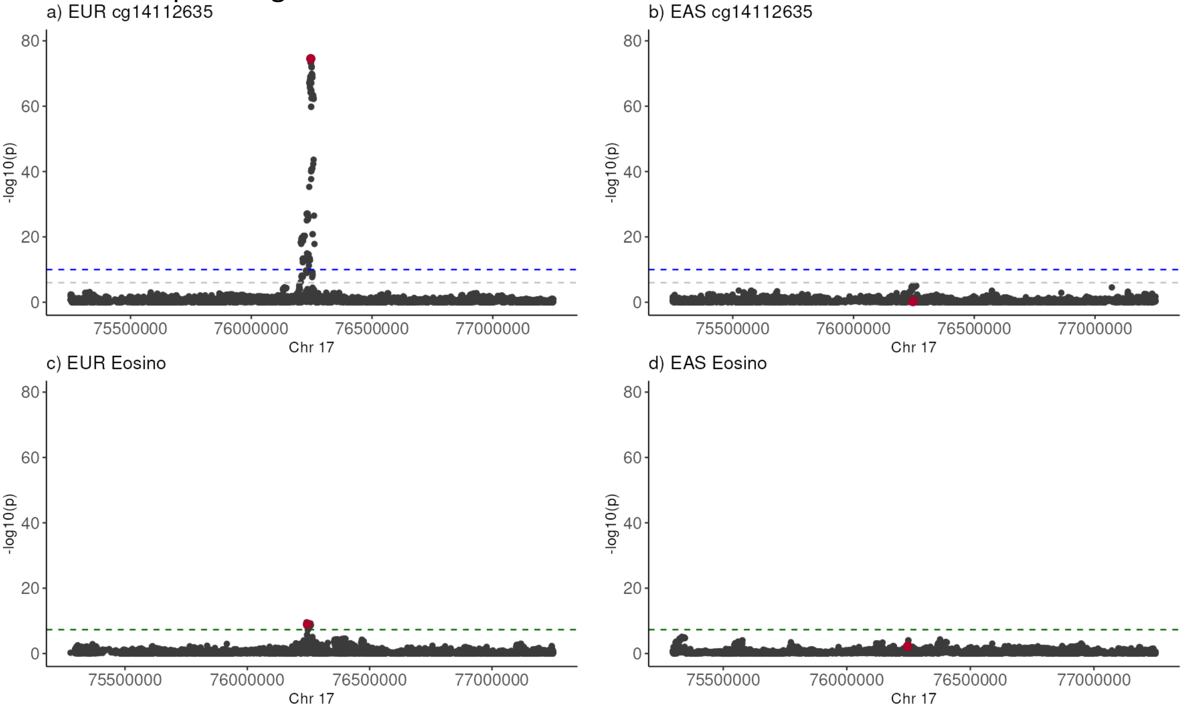

Trait Eosino: probe cg21959090

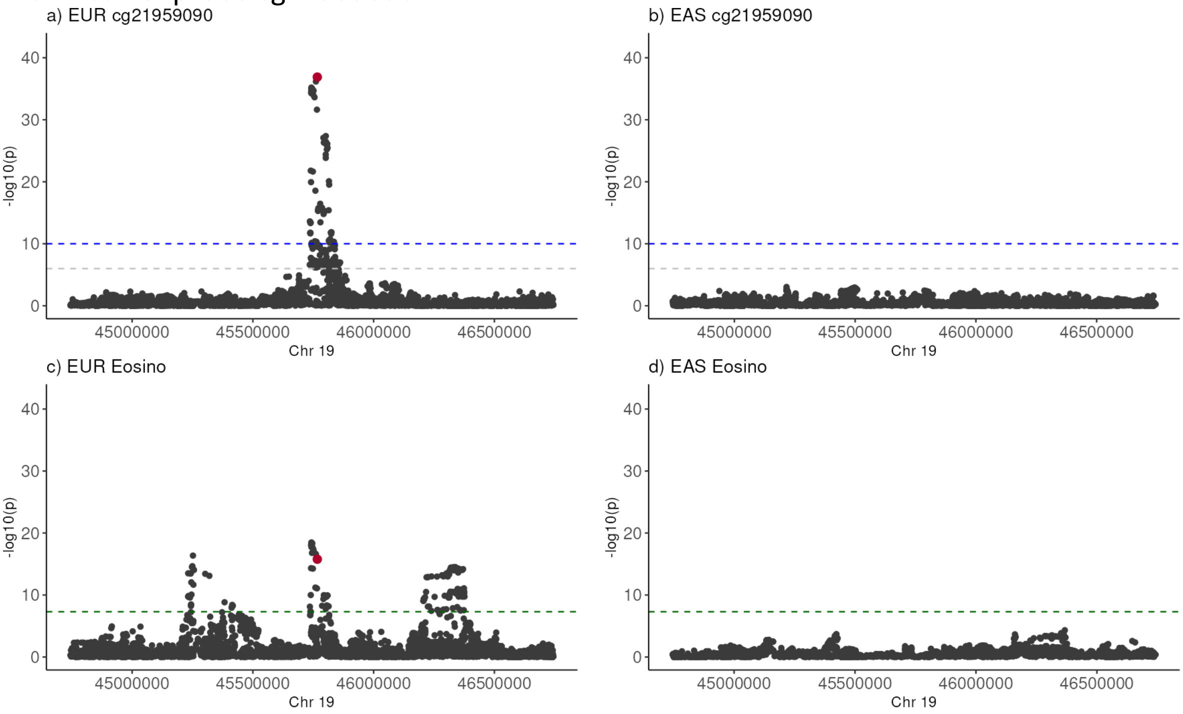

Trait GGT: probe cg13235717

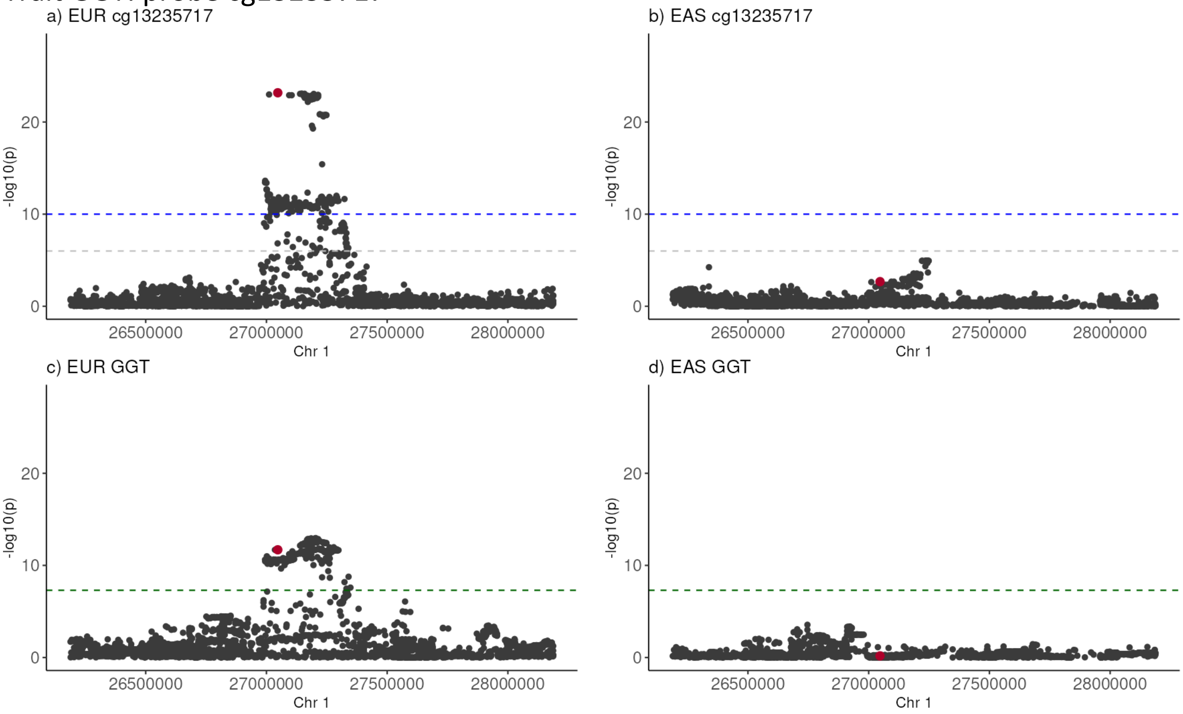

Trait GGT: probe cg00409636

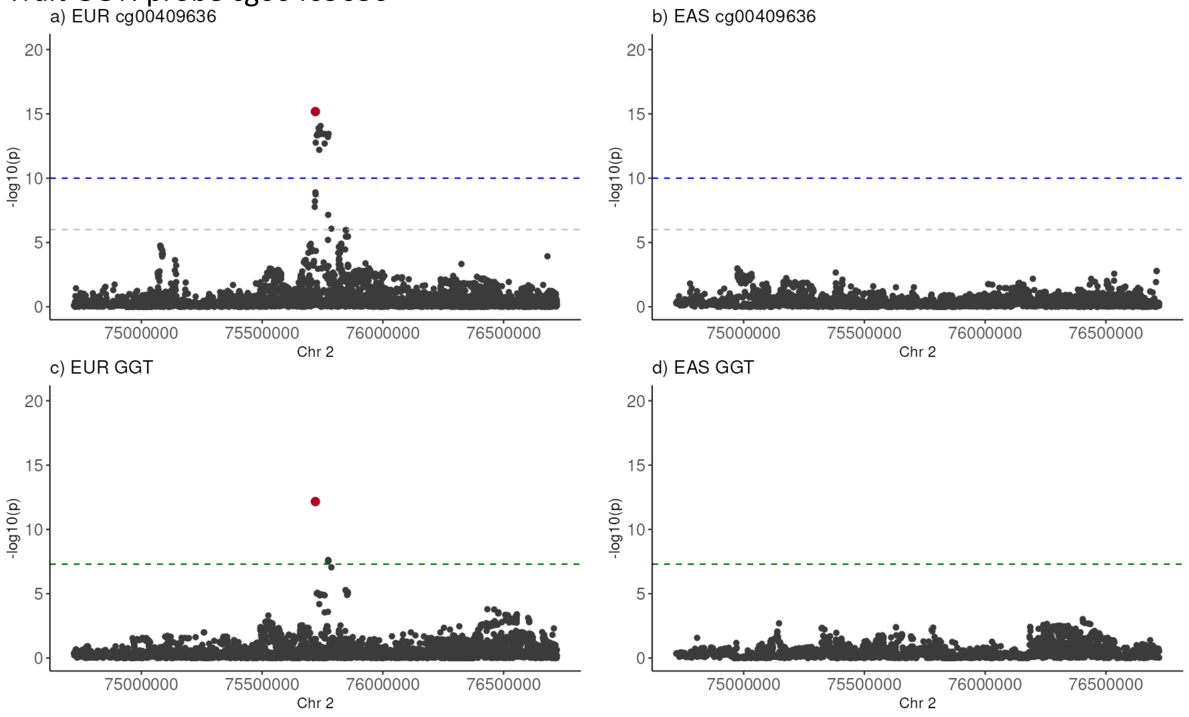

Trait GGT: probe cg05914712

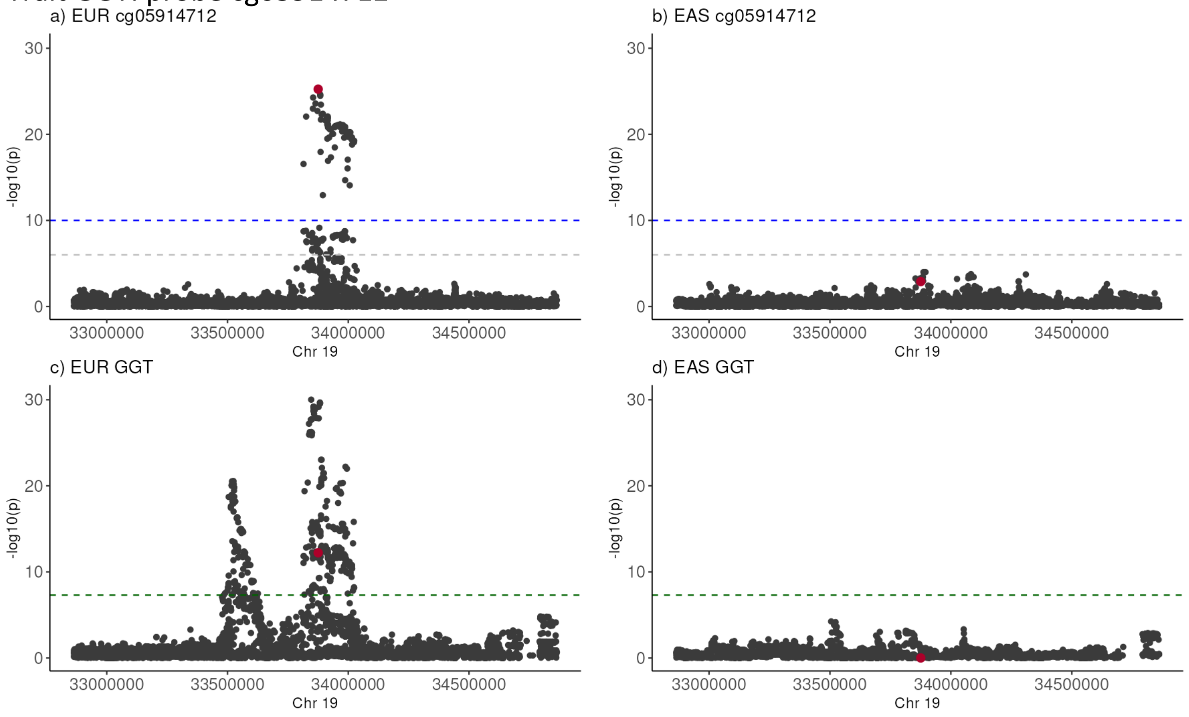

Trait GGT: probe cg09479818

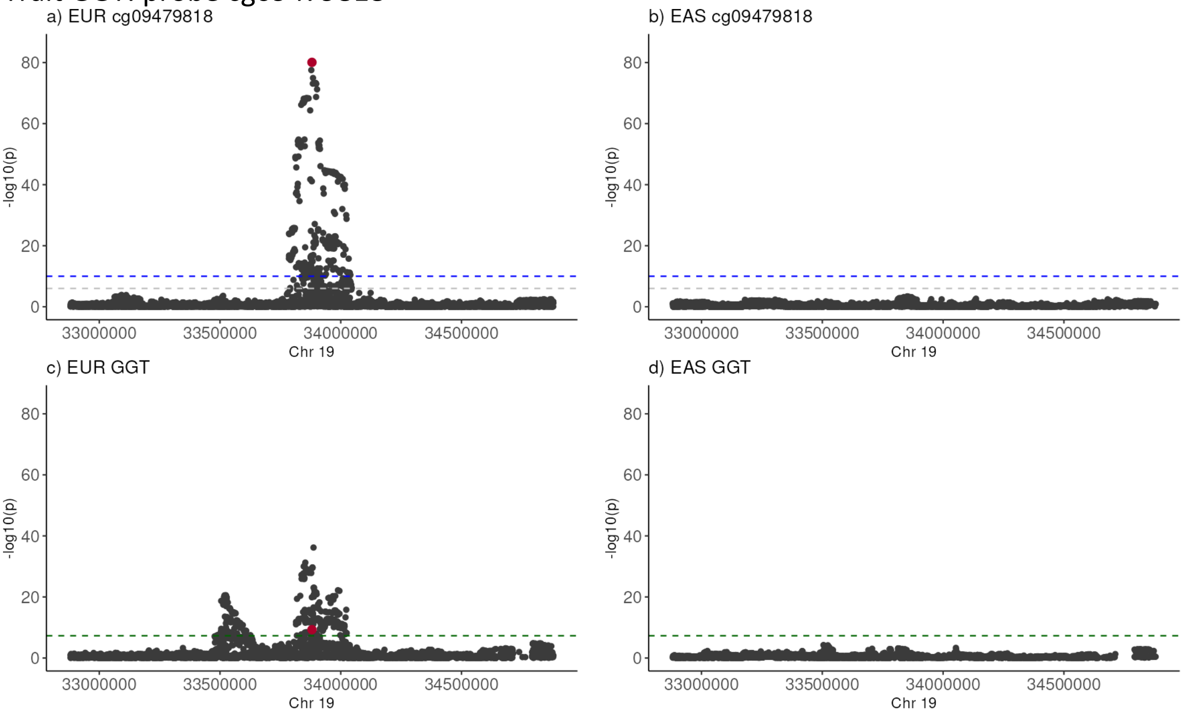

Trait Gla: probe cg24023194

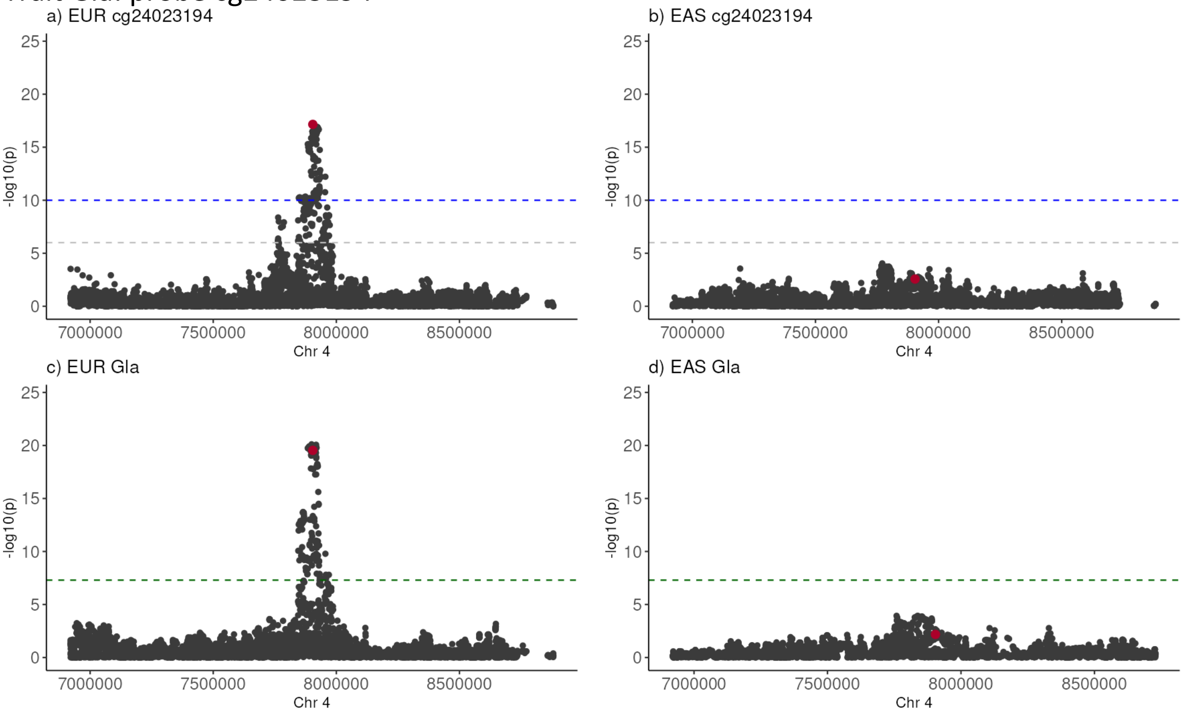

Trait Goi: probe cg11258943

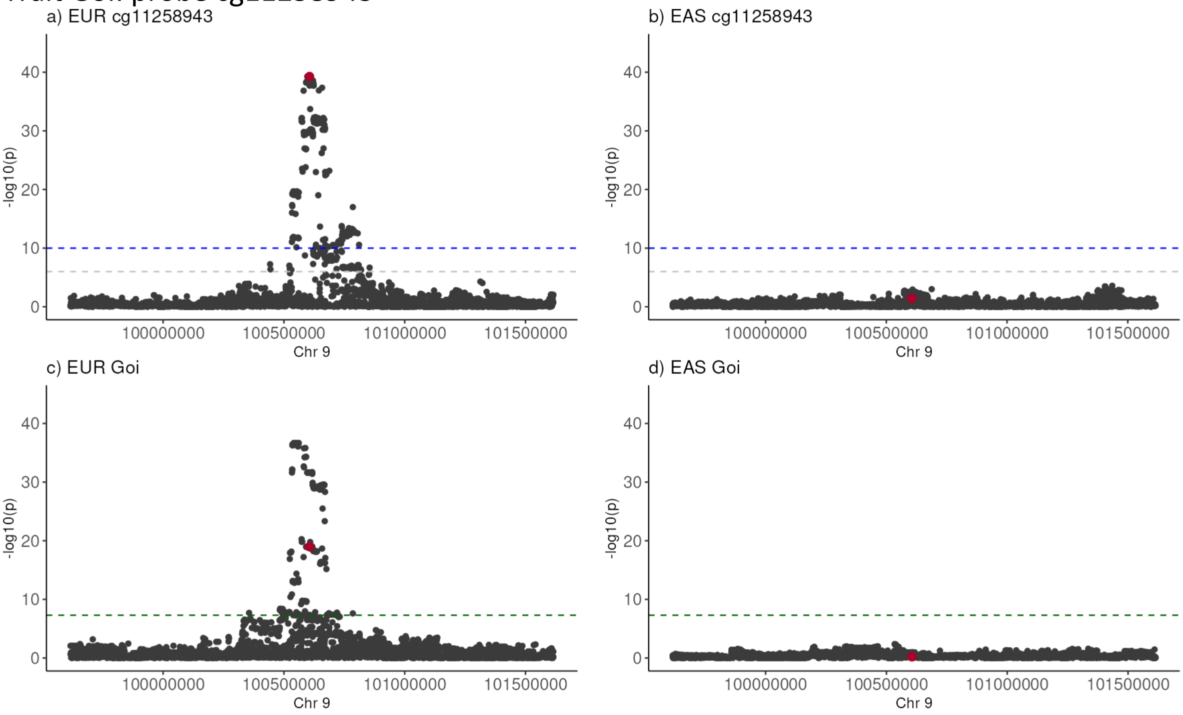

Trait Goi: probe cg13827859

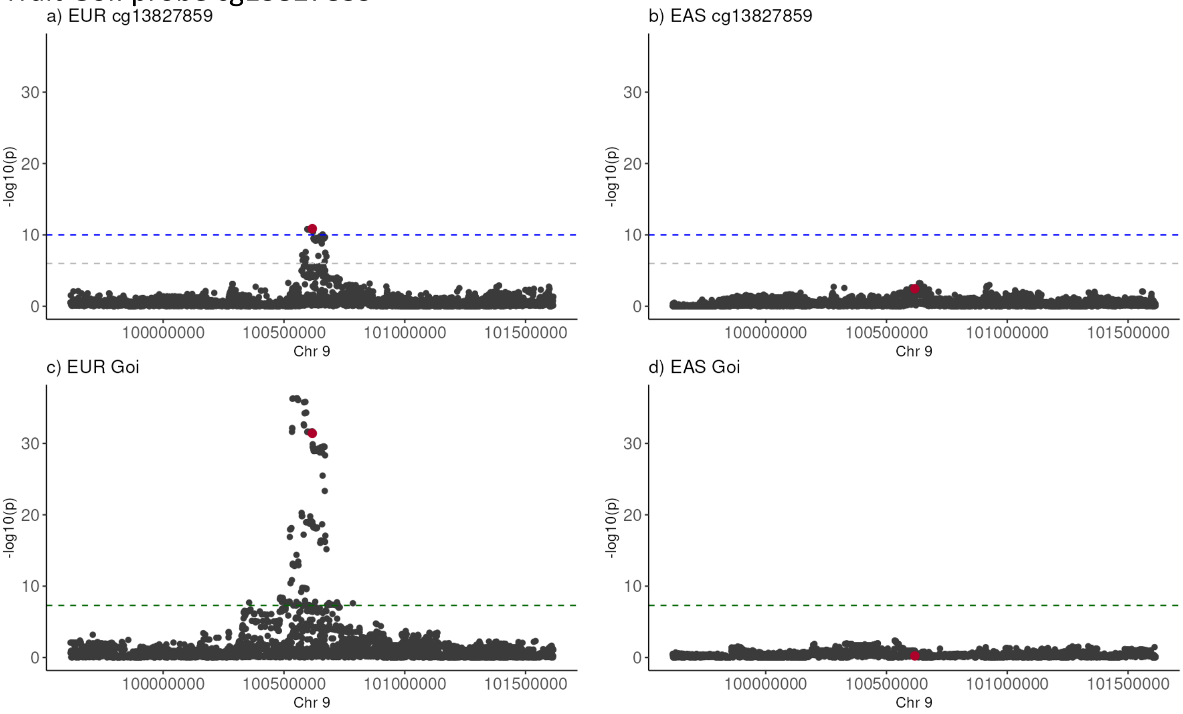

Trait H03A: probe cg19084075

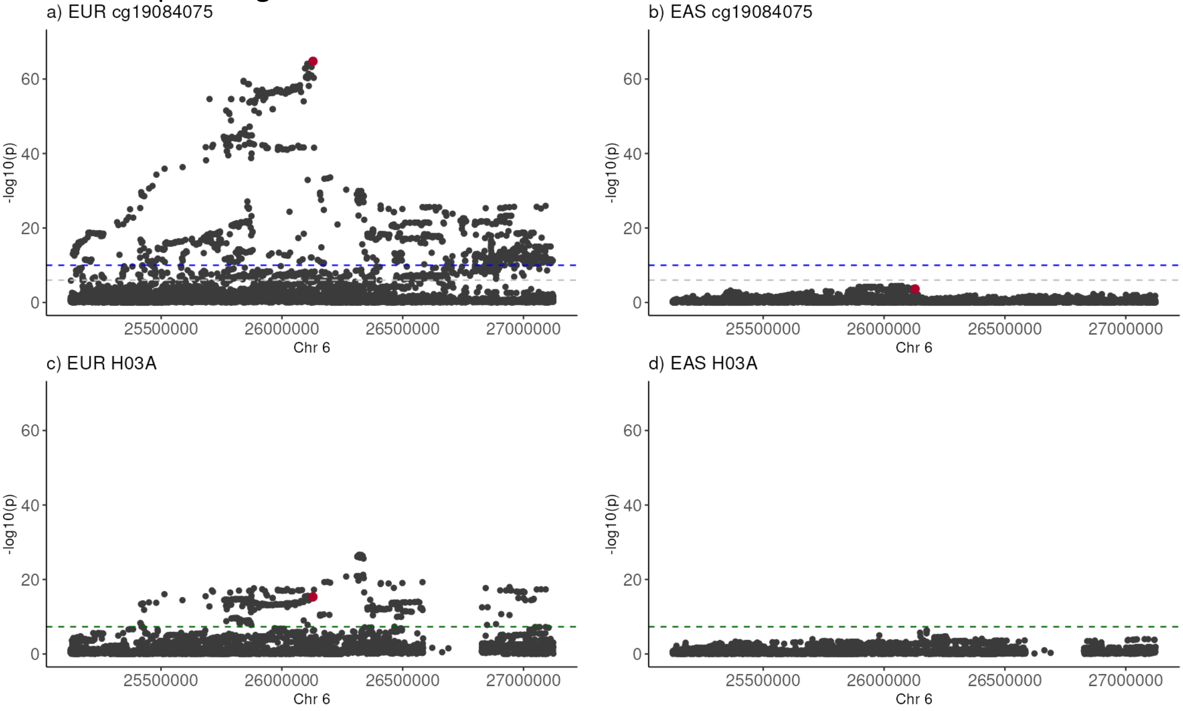

Trait H03A: probe cg10458080

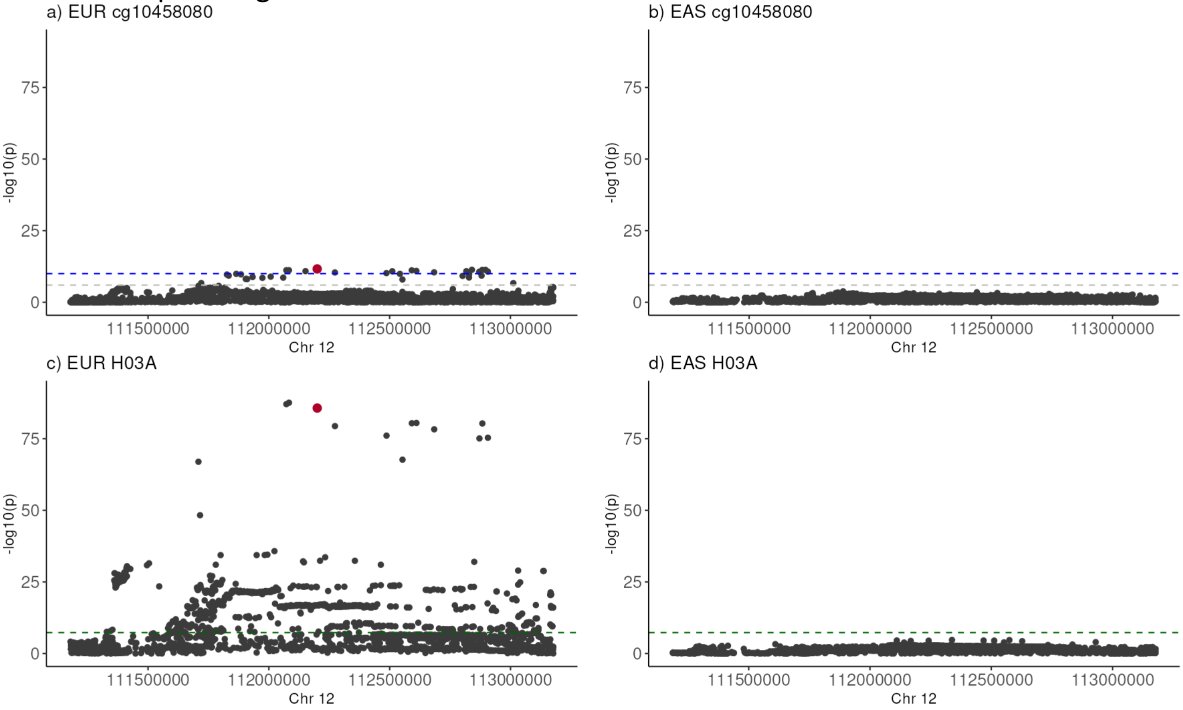

Trait H03A: probe cg03982355

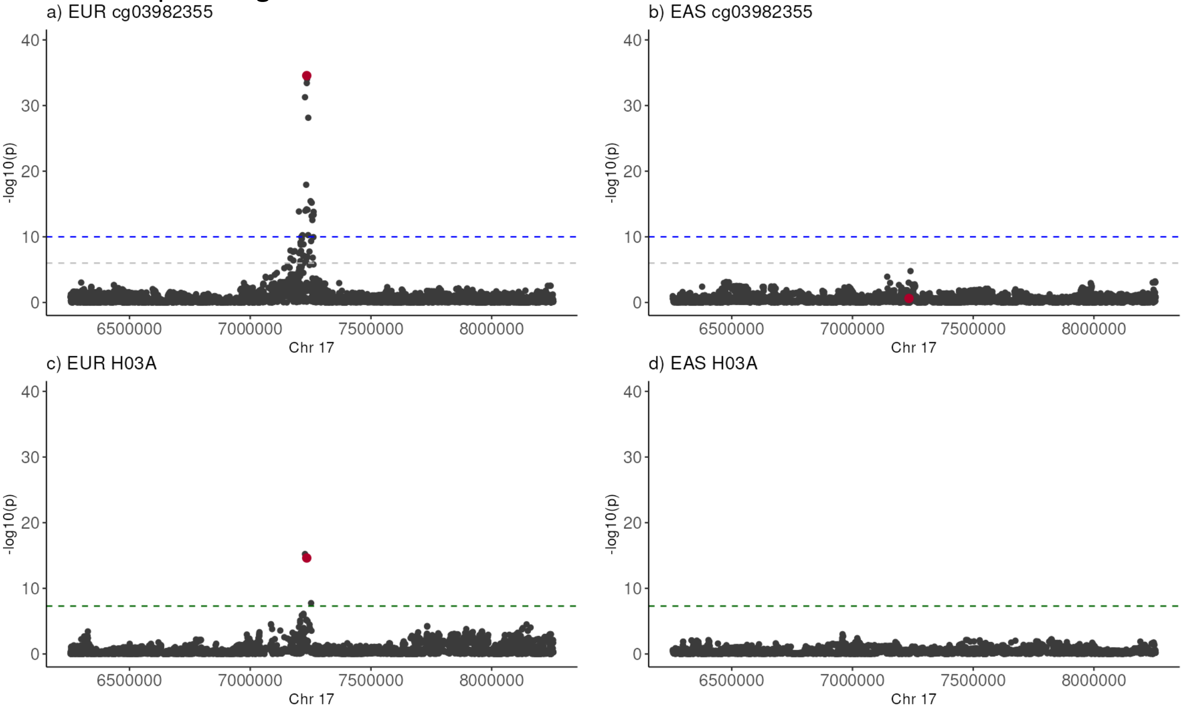

Trait H03A: probe cg19175364

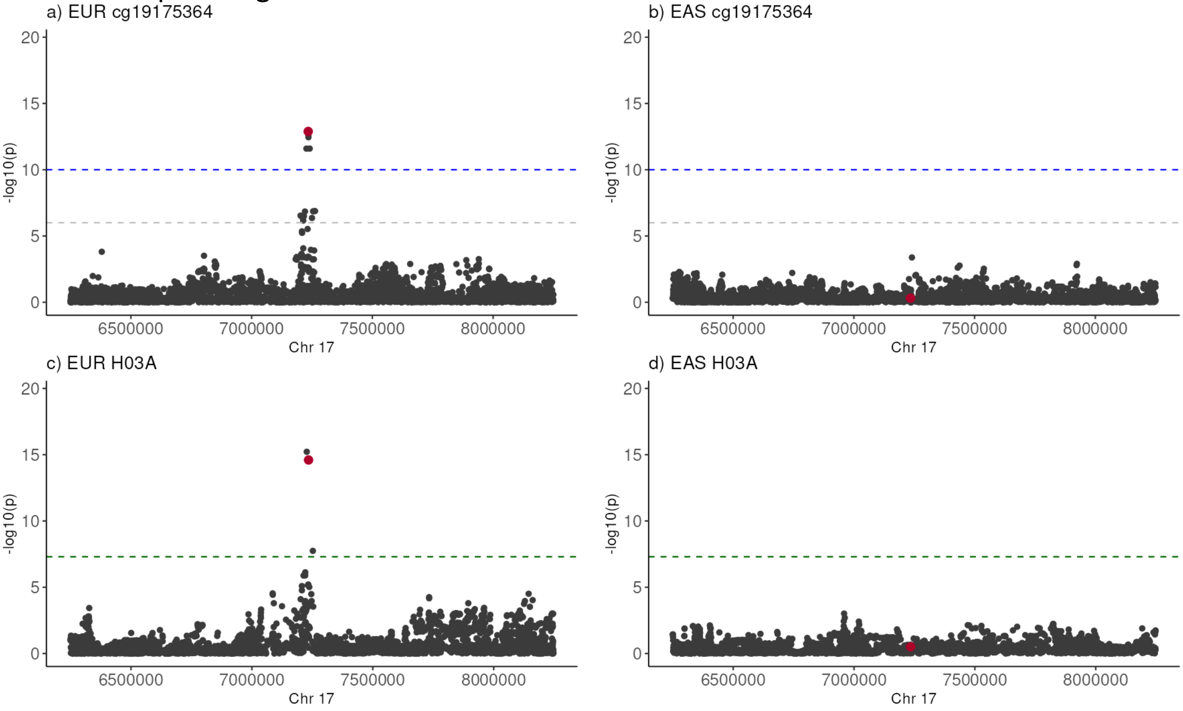

Trait H03A: probe cg00507154

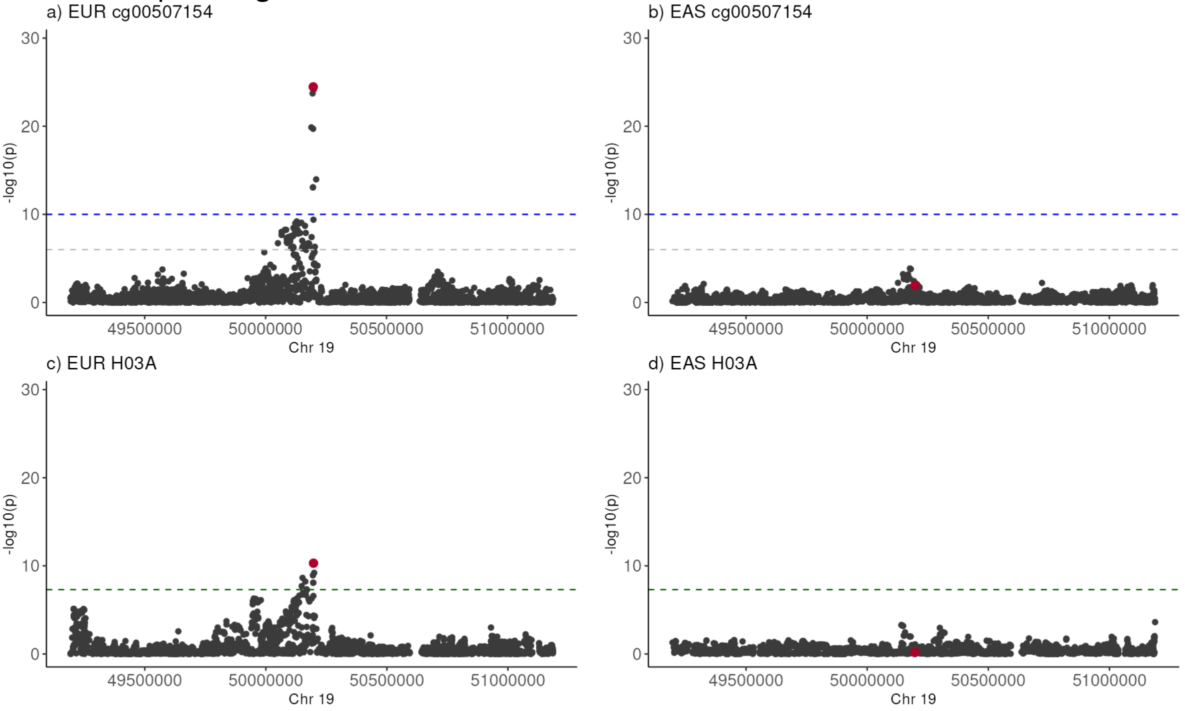

Trait Hb: probe cg07140482

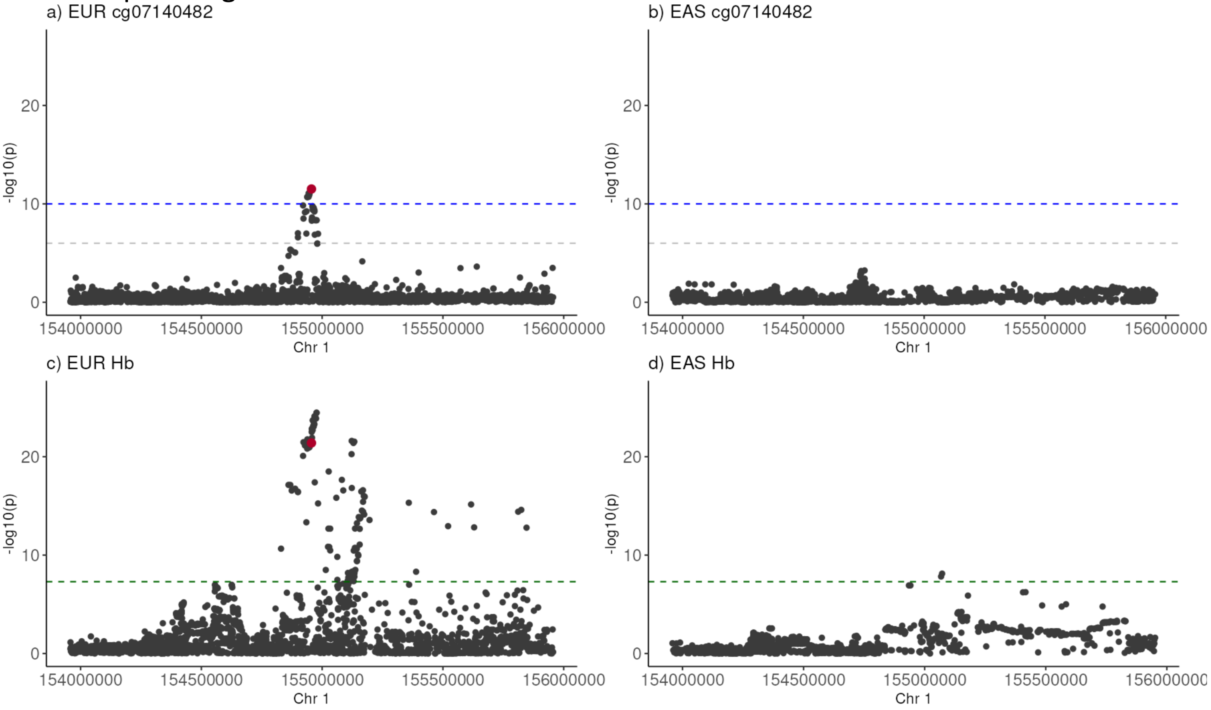

Trait Hb: probe cg08360599

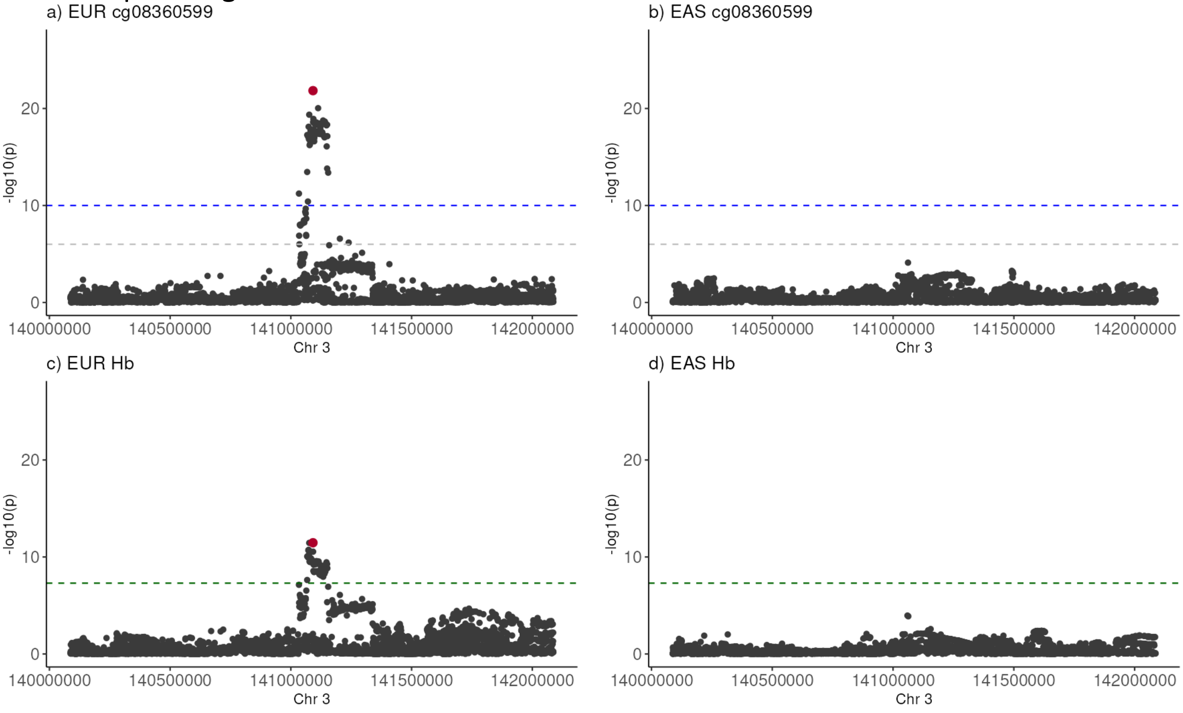

Trait Hb: probe cg13029400

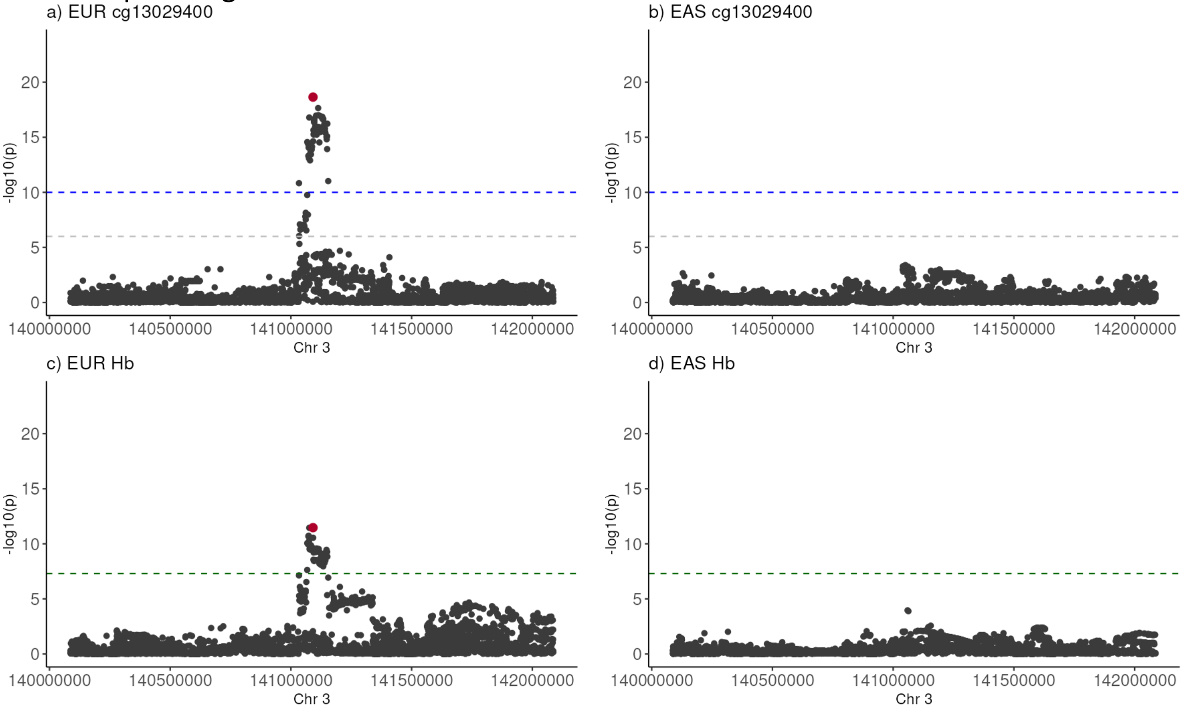

Trait Hb: probe cg13787933

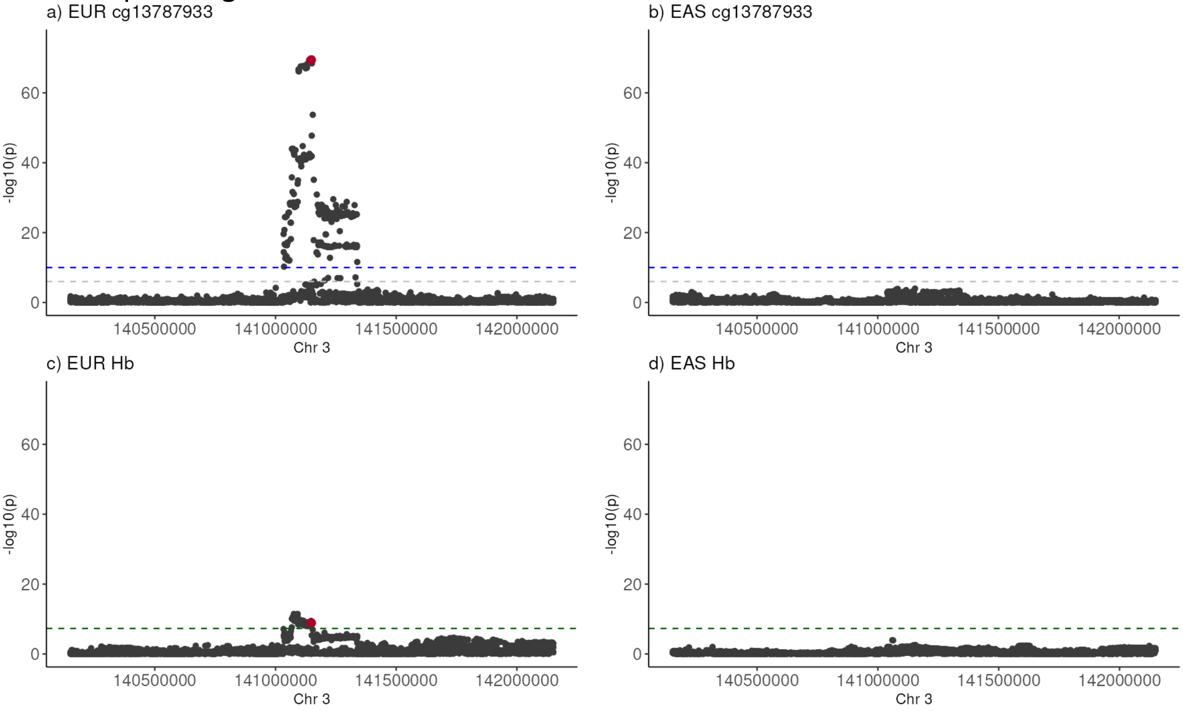

Trait Hb: probe cg21370924

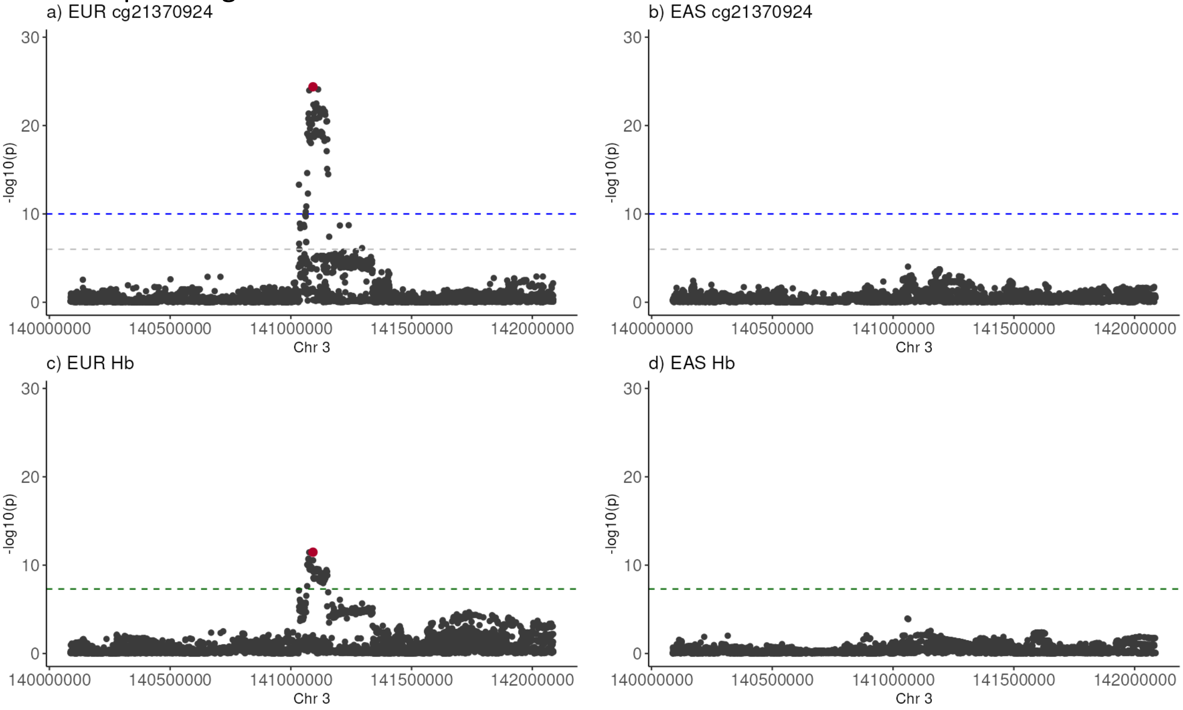

Trait Hb: probe cg07226470

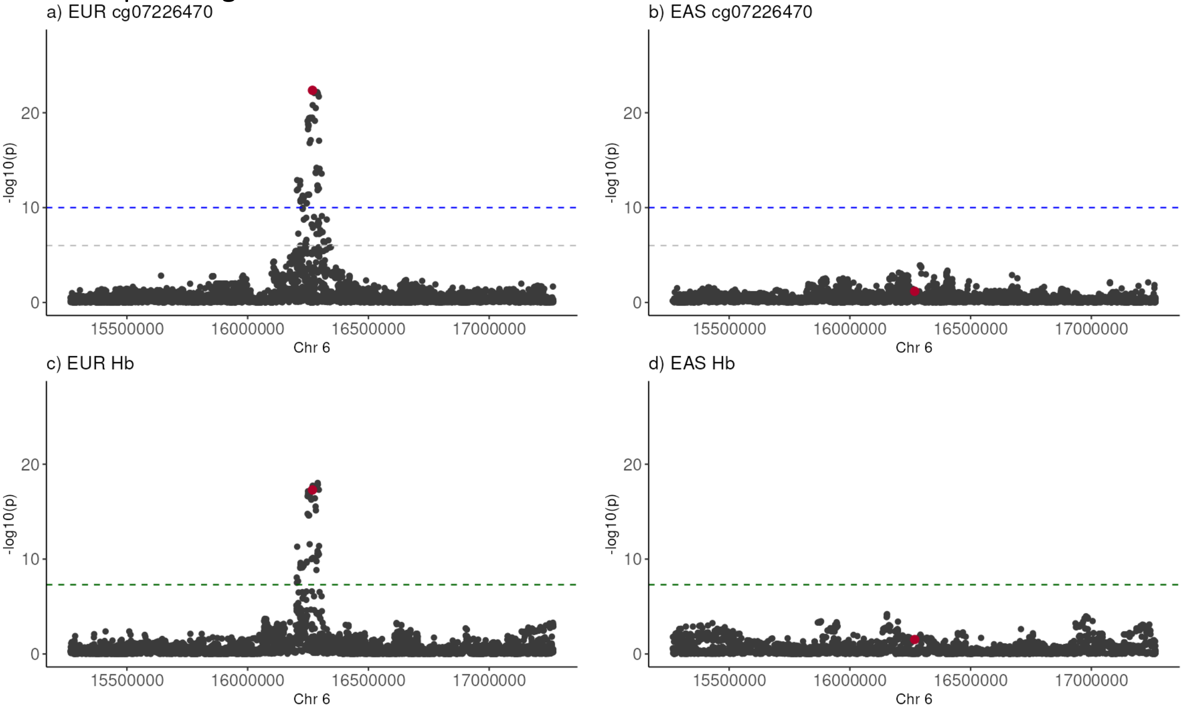

Trait Hb: probe cg21959090

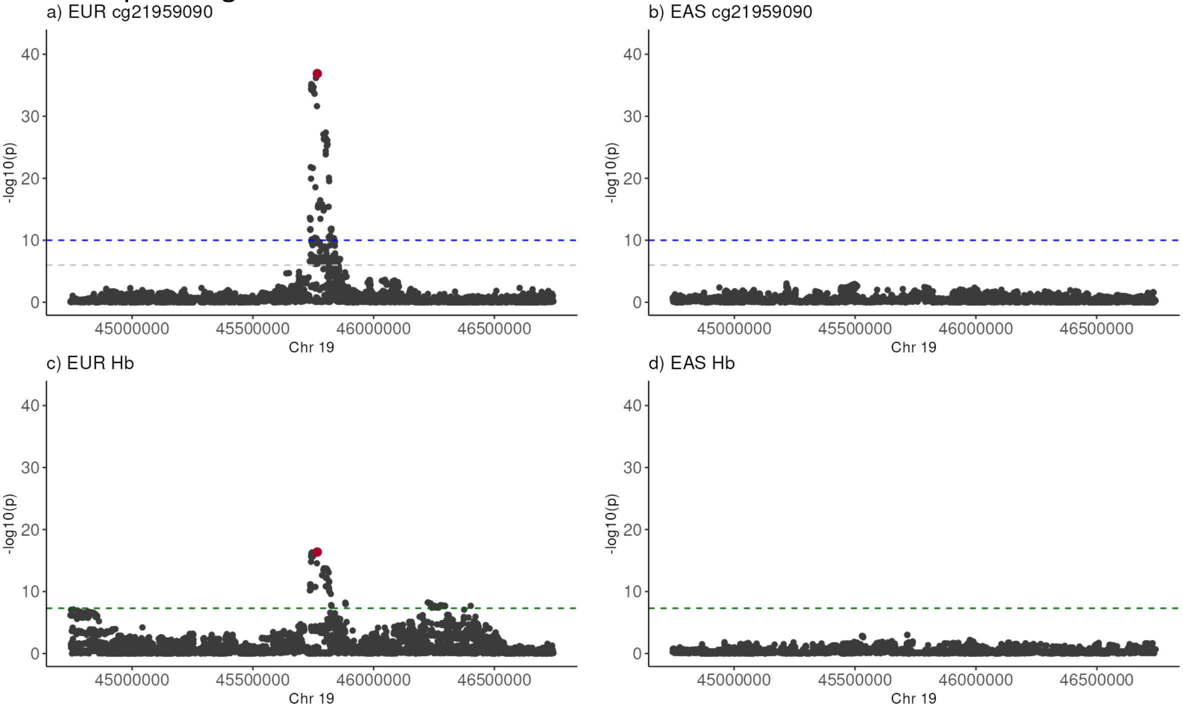

Trait HbA1c: probe cg04781916

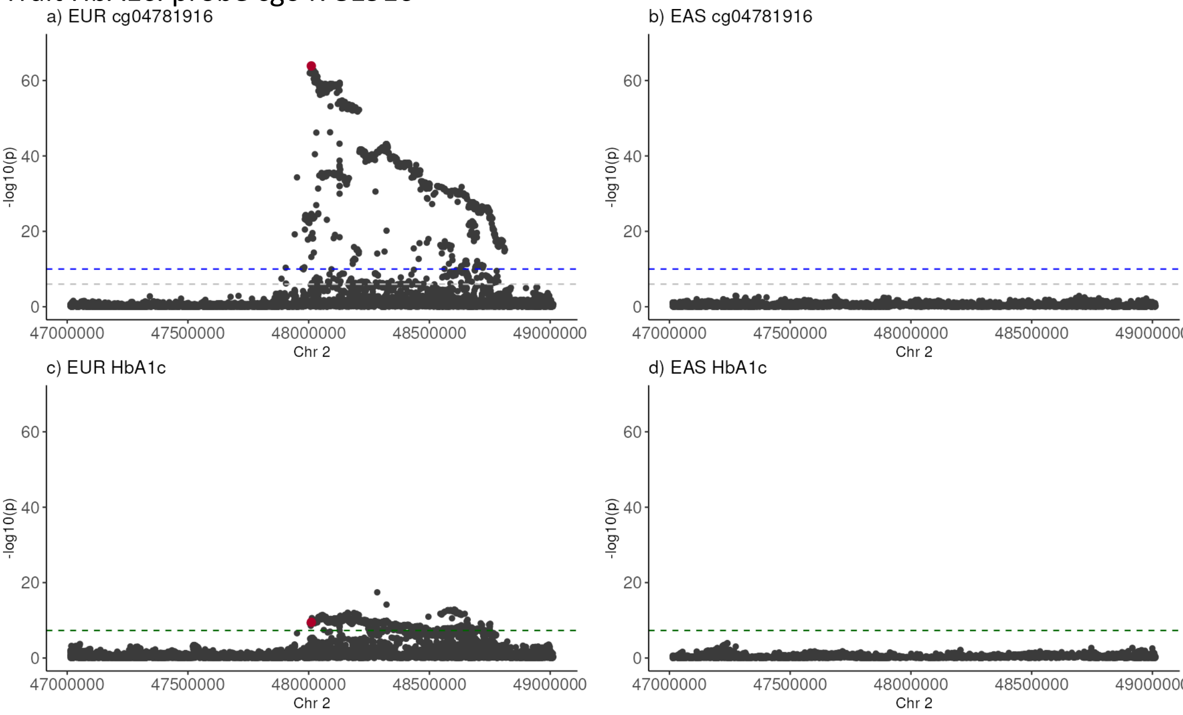

Trait HbA1c: probe cg25173392

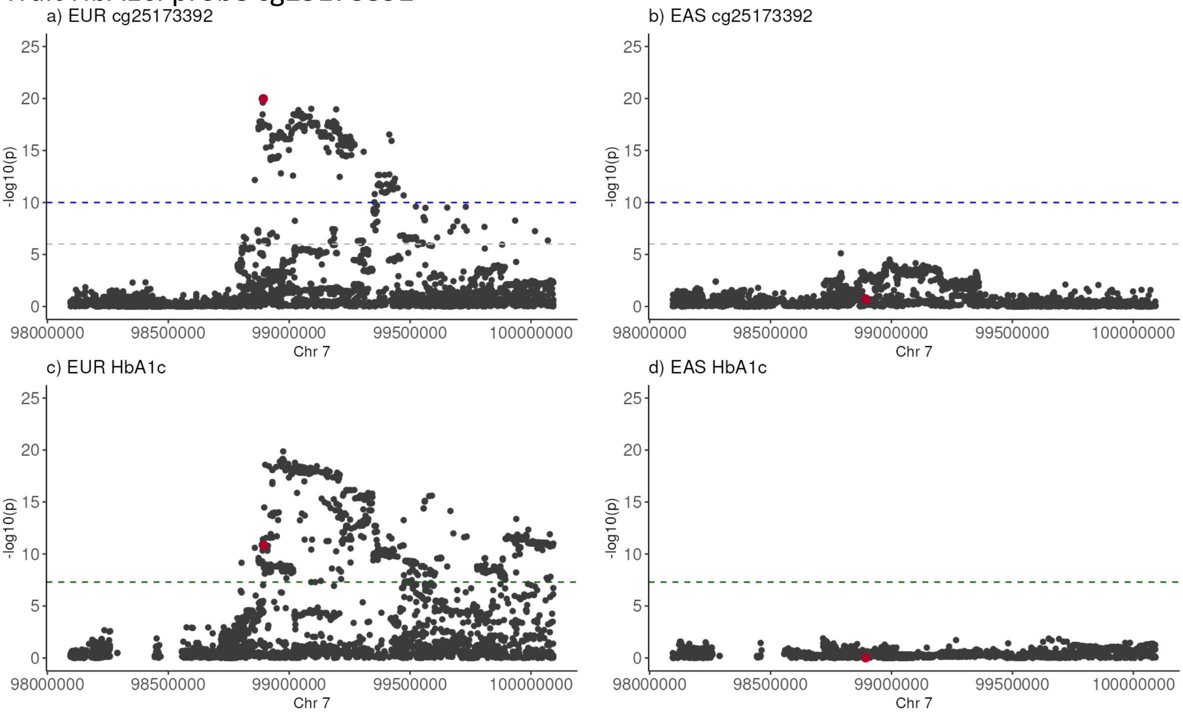

Trait HbA1c: probe cg25721818

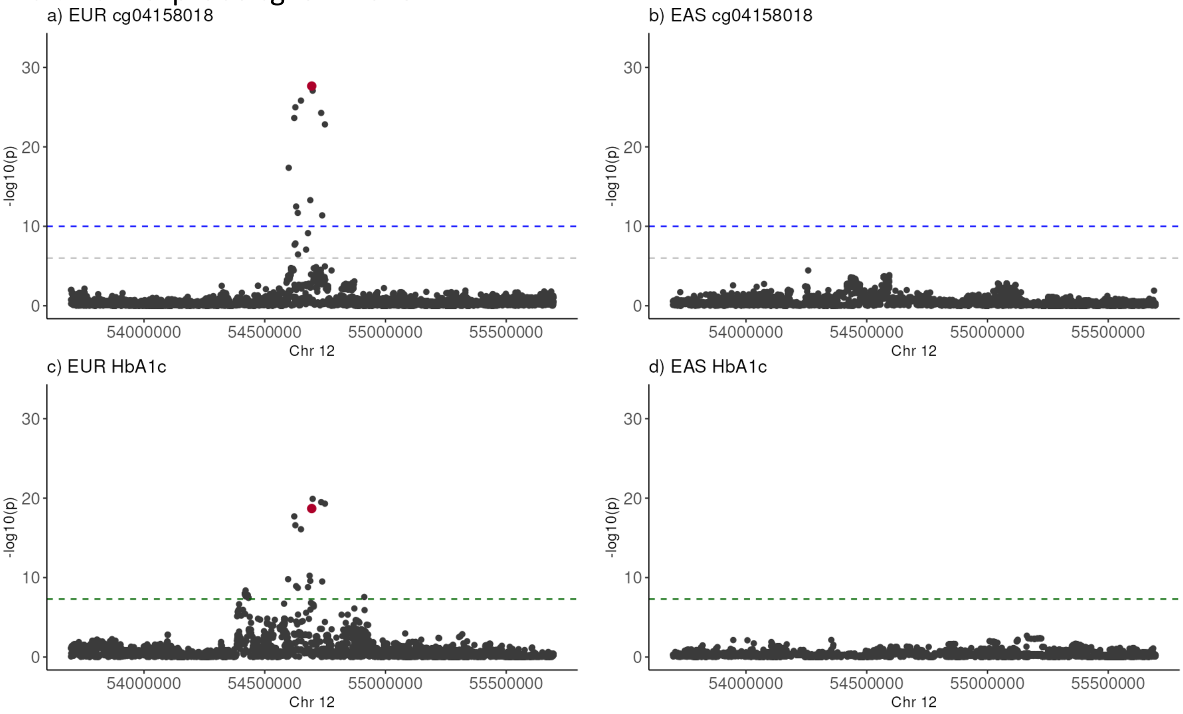

Trait HbA1c: probe cg14872454

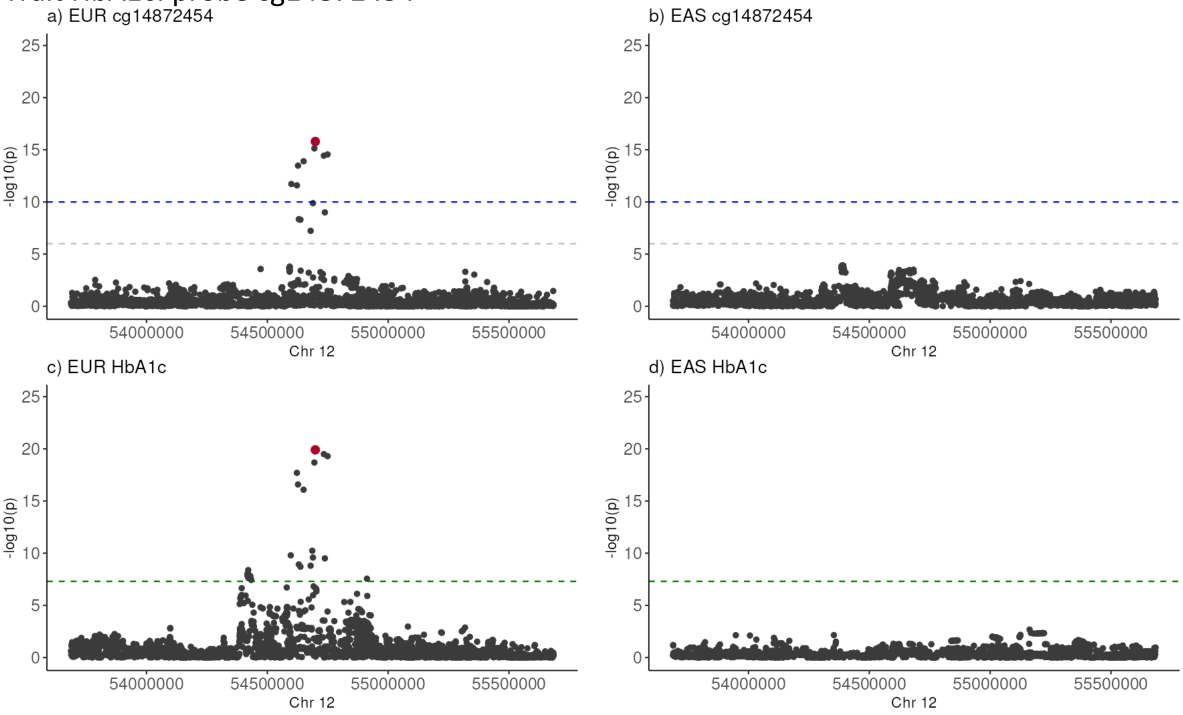

Trait HbA1c: probe cg17230002

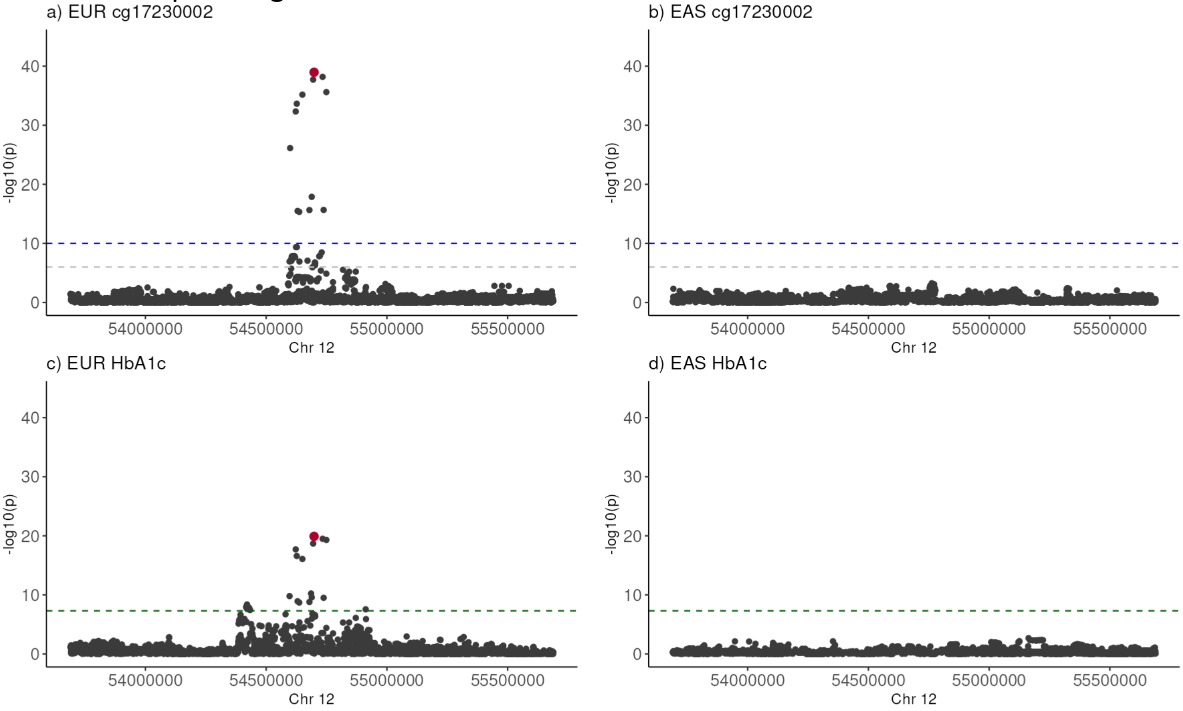

Trait HbA1c: probe cg17869315

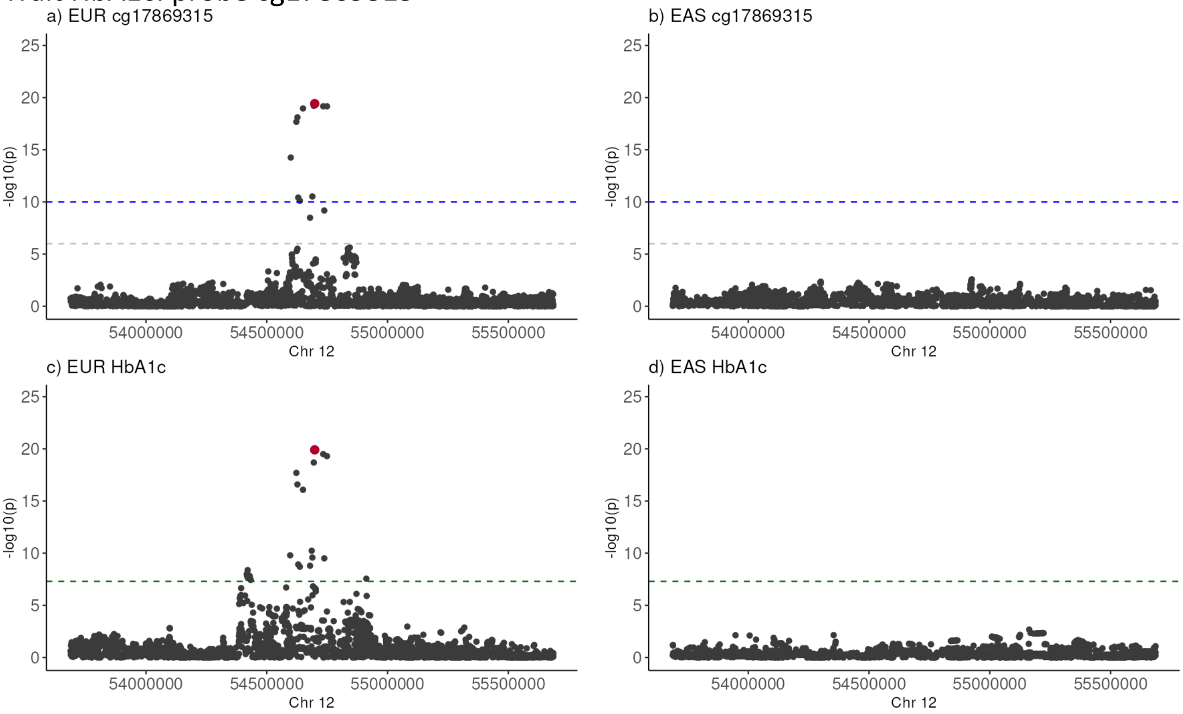

Trait HbA1c: probe cg21376004

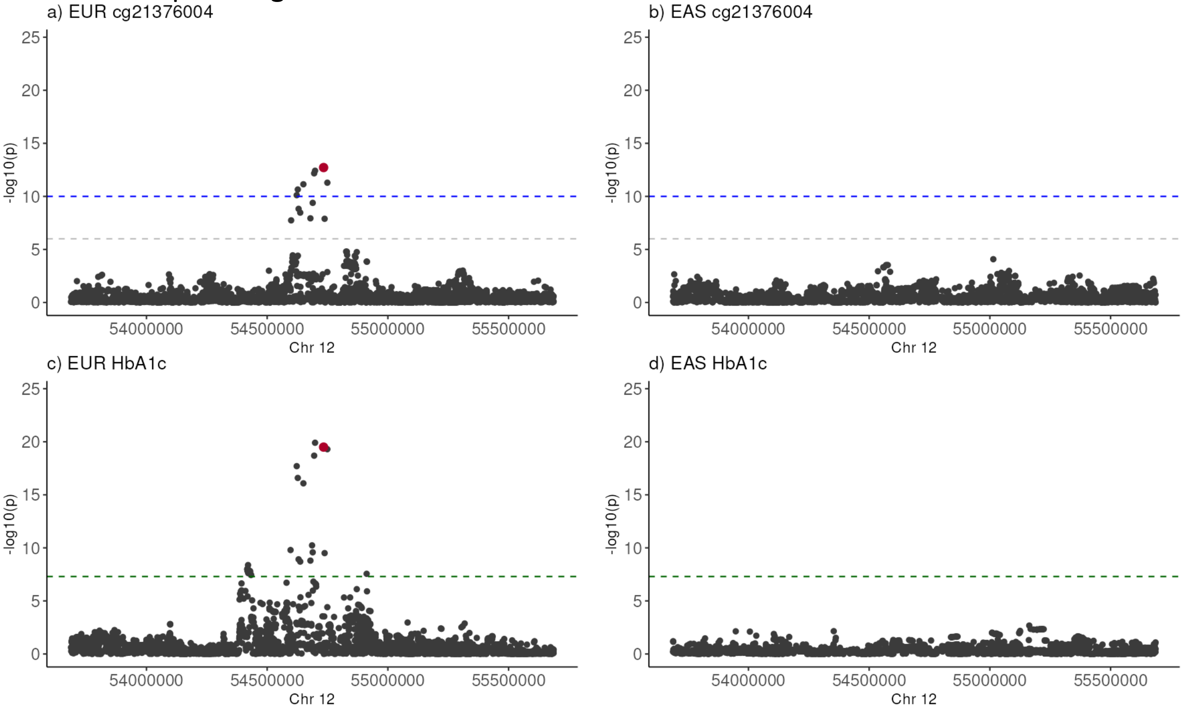

Trait HbA1c: probe cg23140706

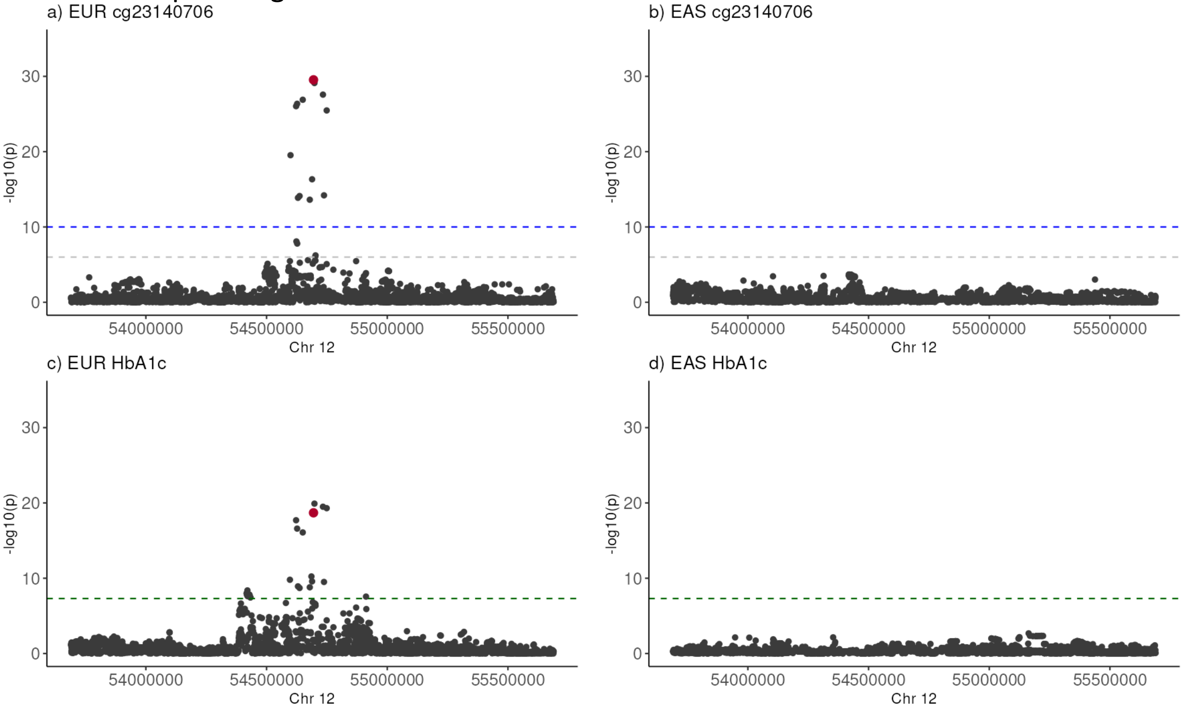

Trait HbA1c: probe cg04158018

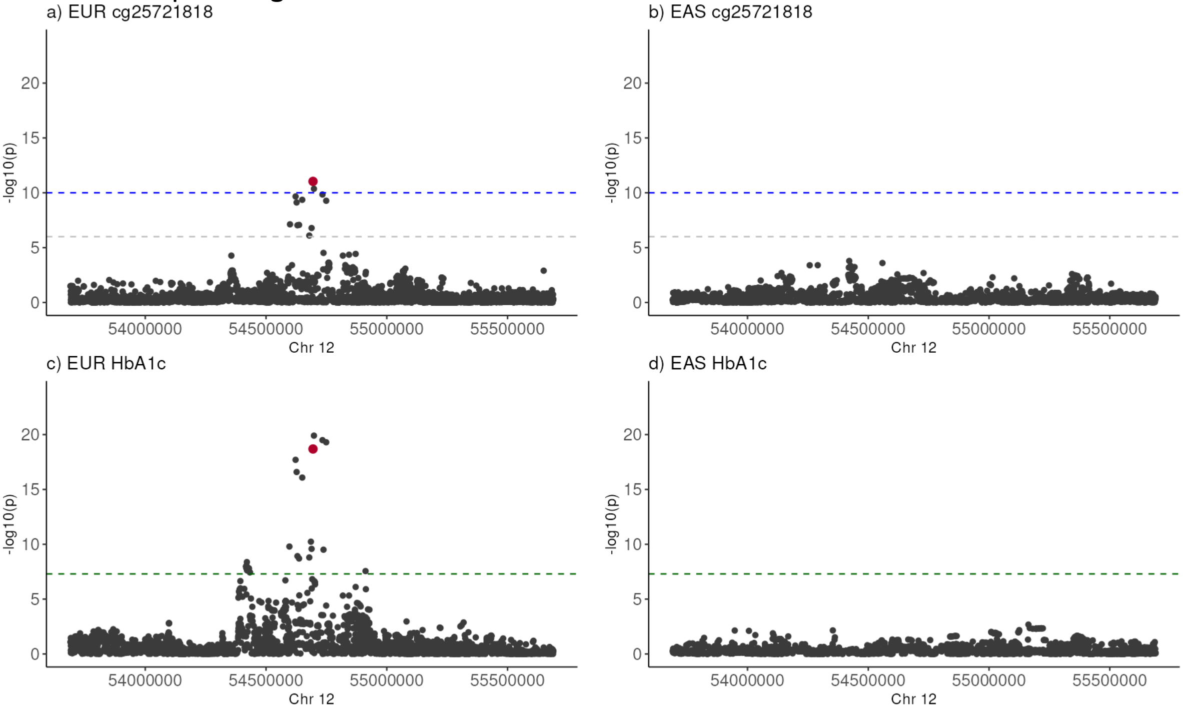

Trait HD: probe cg11258943

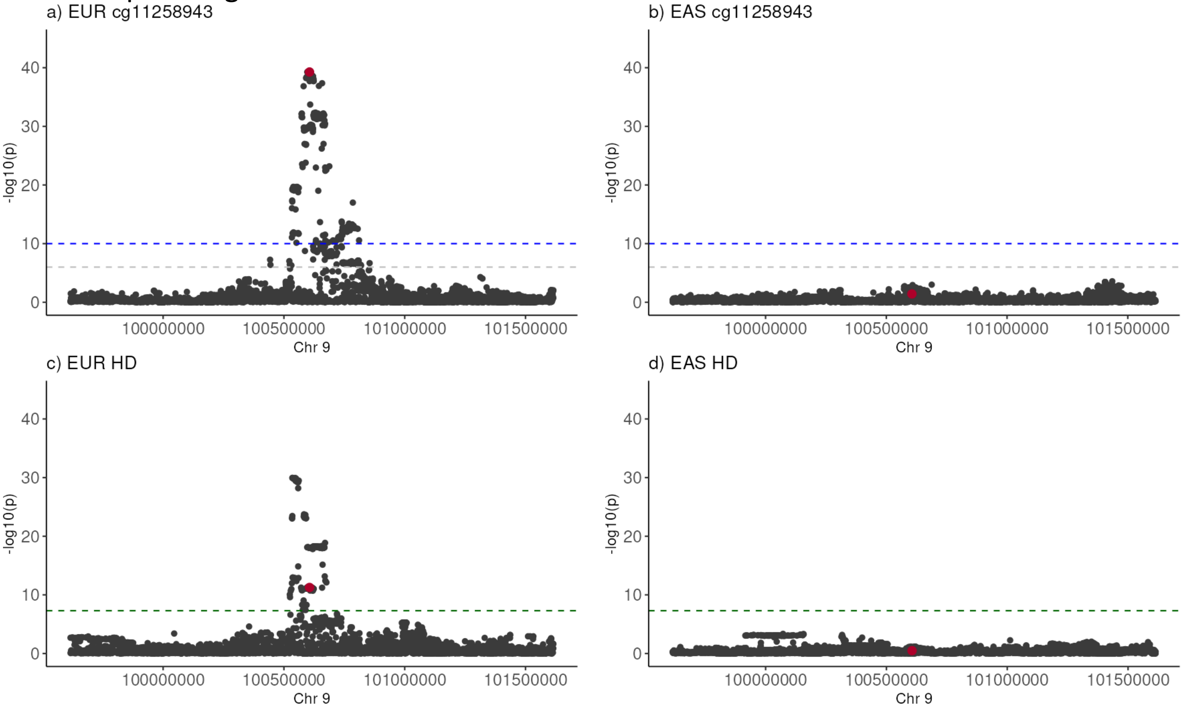

Trait HD: probe cg13827859

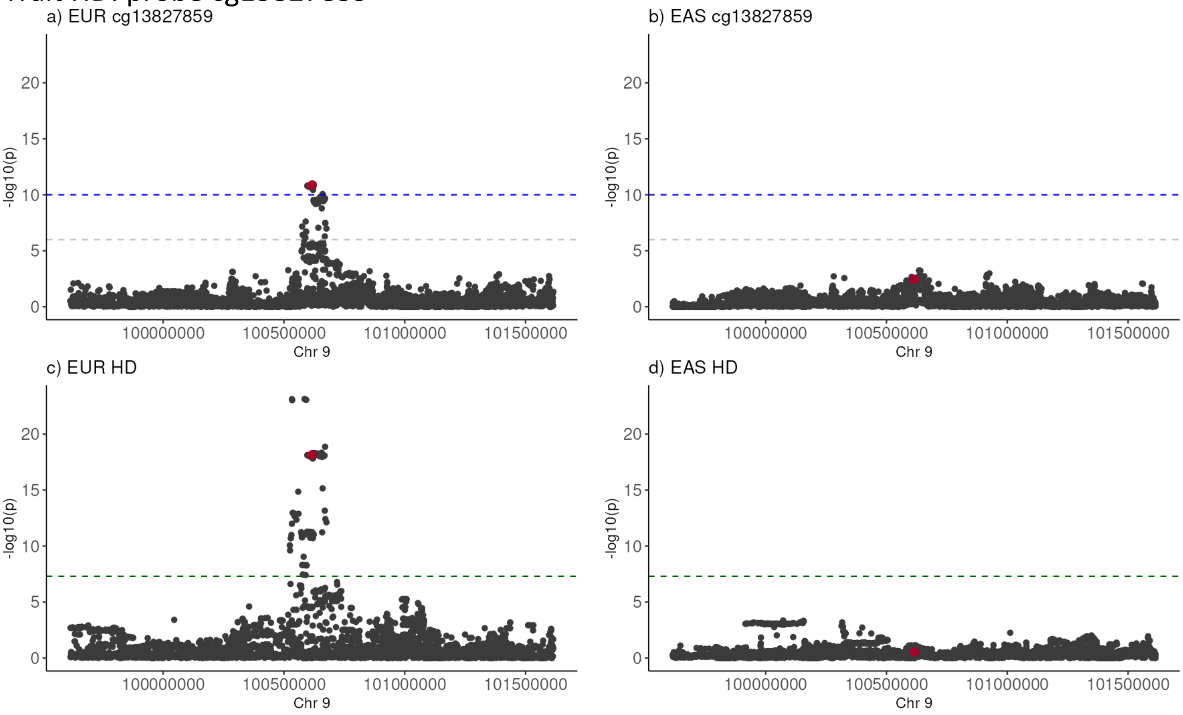

Trait HD: probe cg10458080

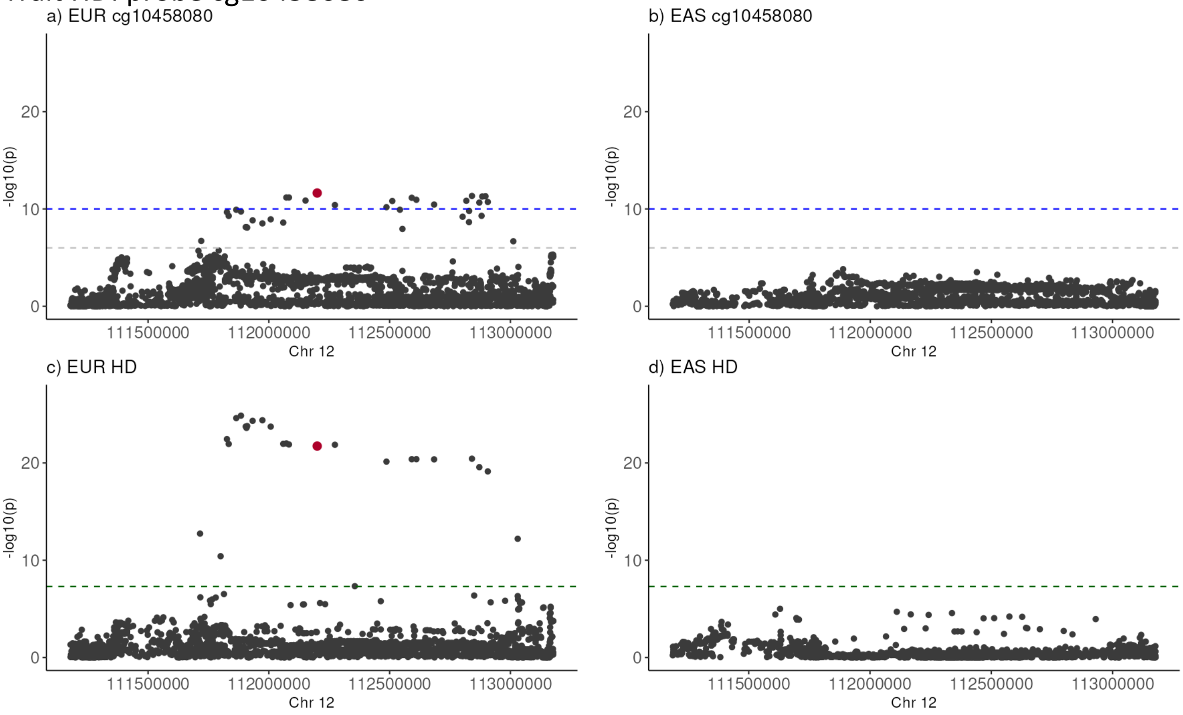

Trait Ht: probe cg04996089

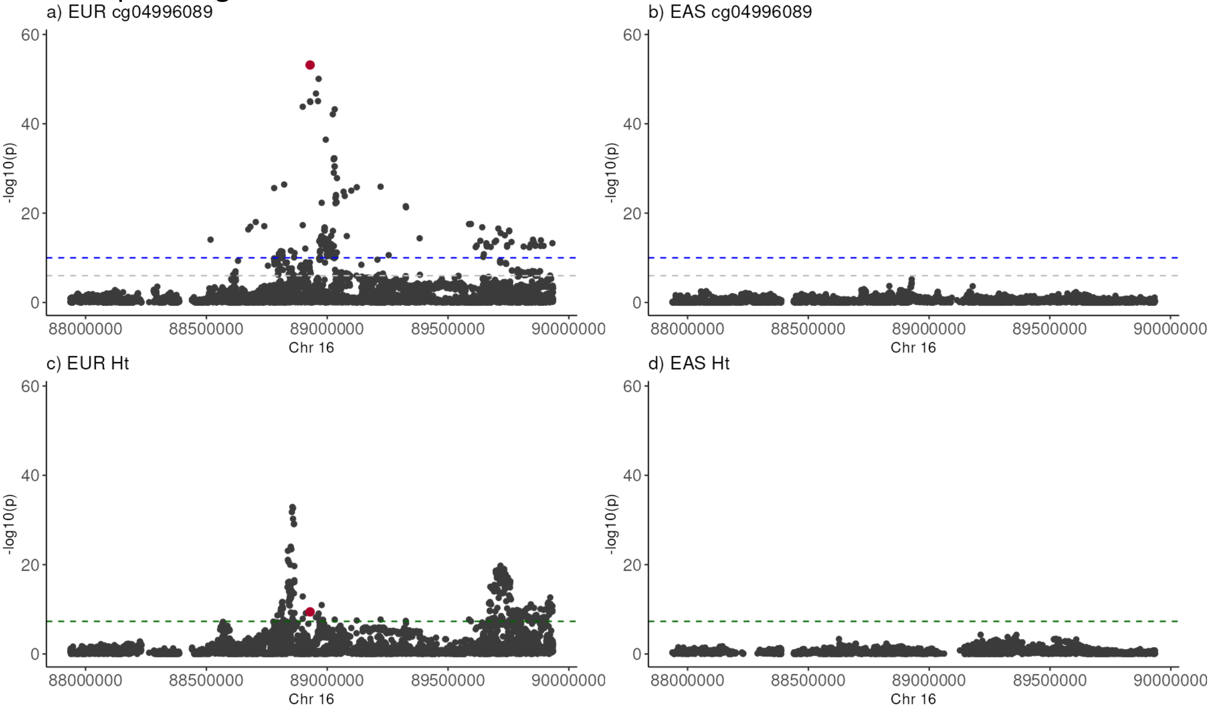

Trait Ht: probe cg07716131

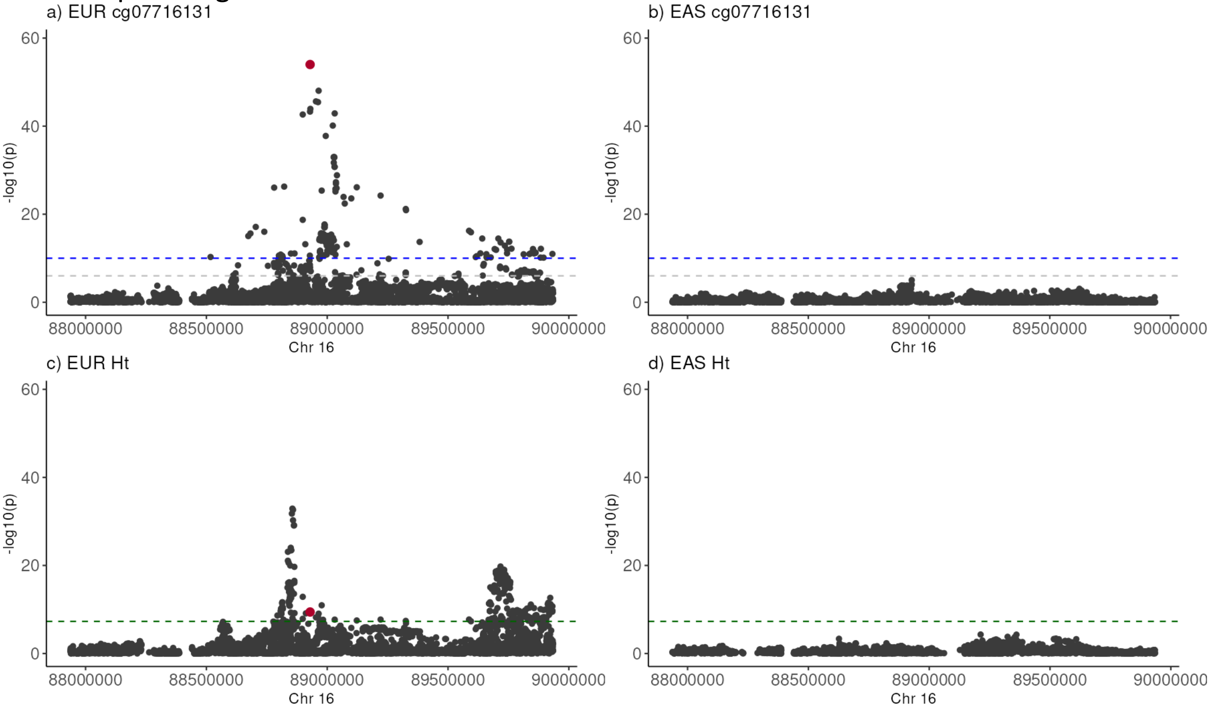

Trait Ht: probe cg08144157

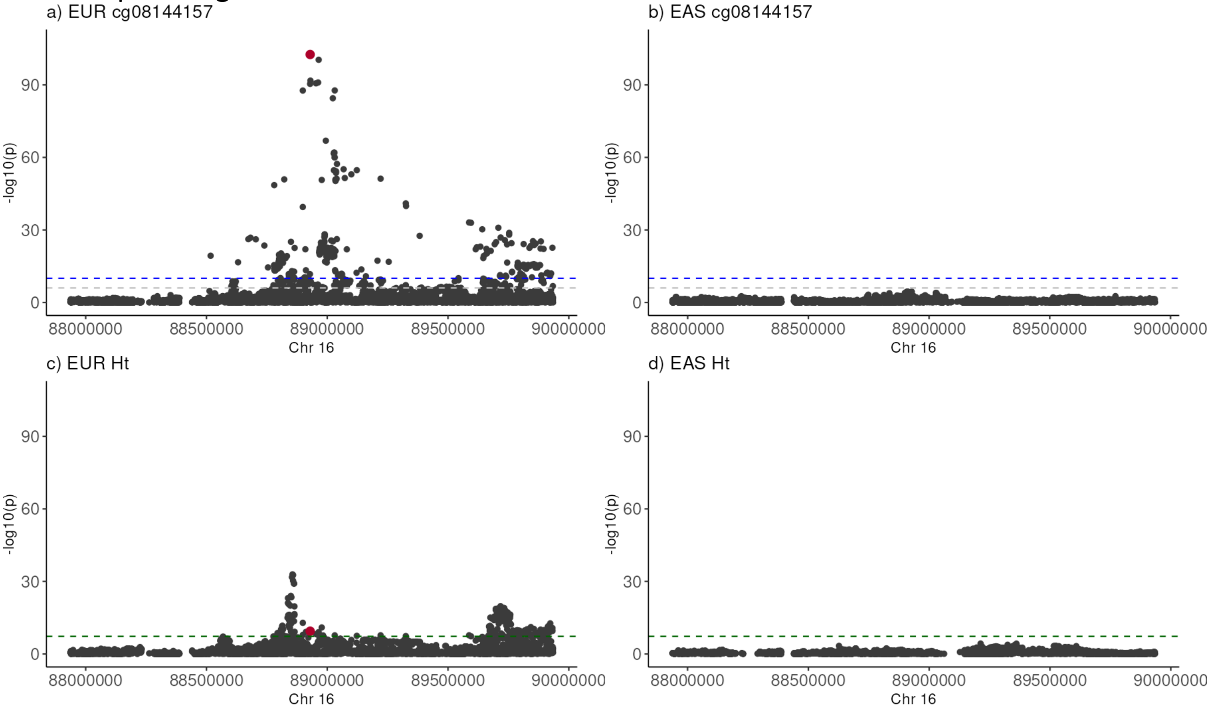

Trait Ht: probe cg09493966

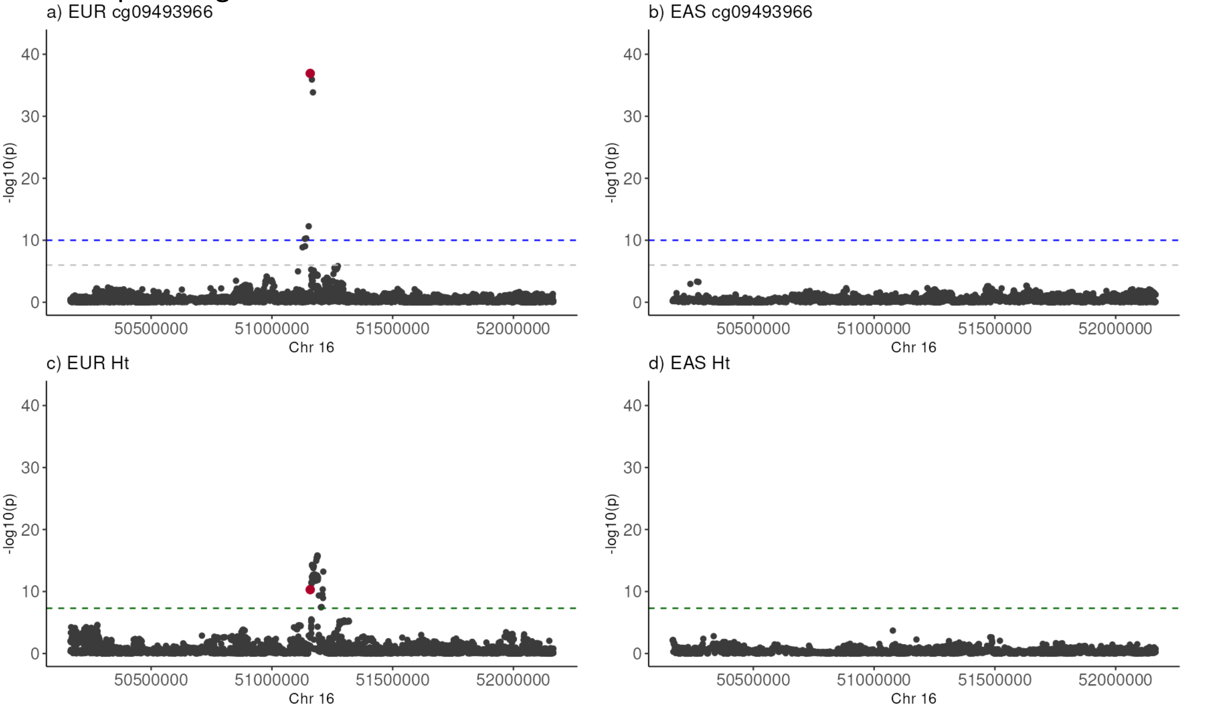

Trait Ht: probe cg22728782

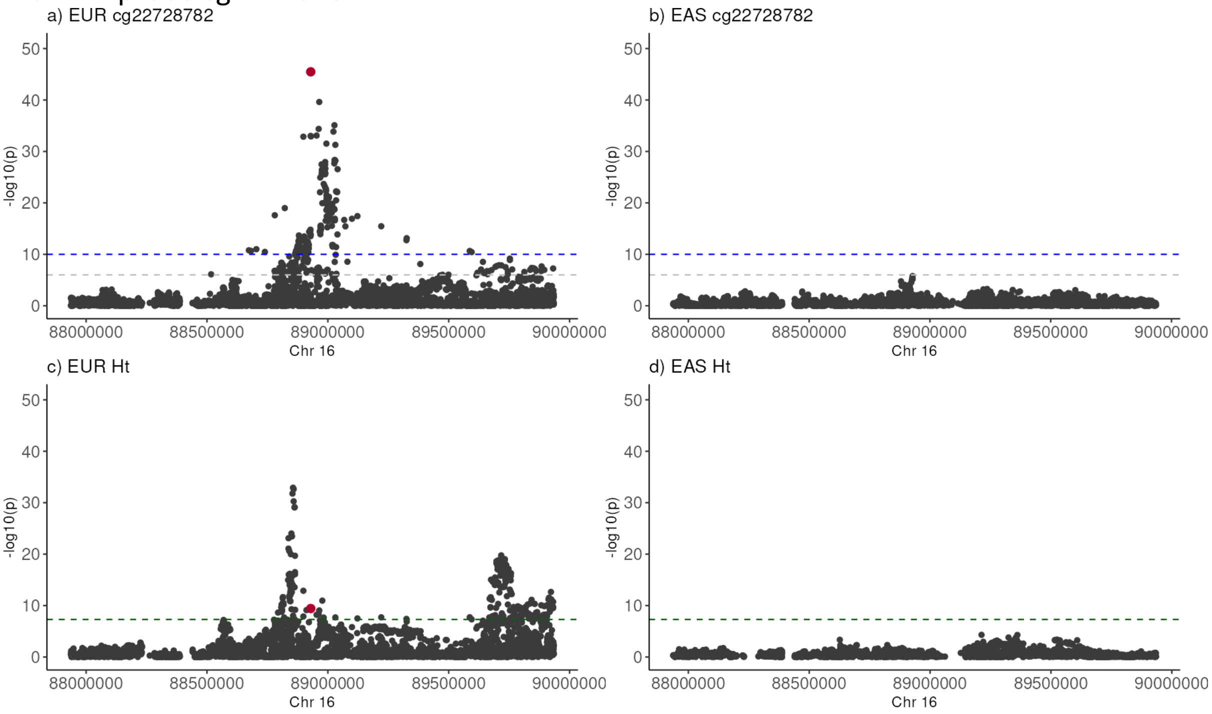

Trait Ht: probe cg27483694

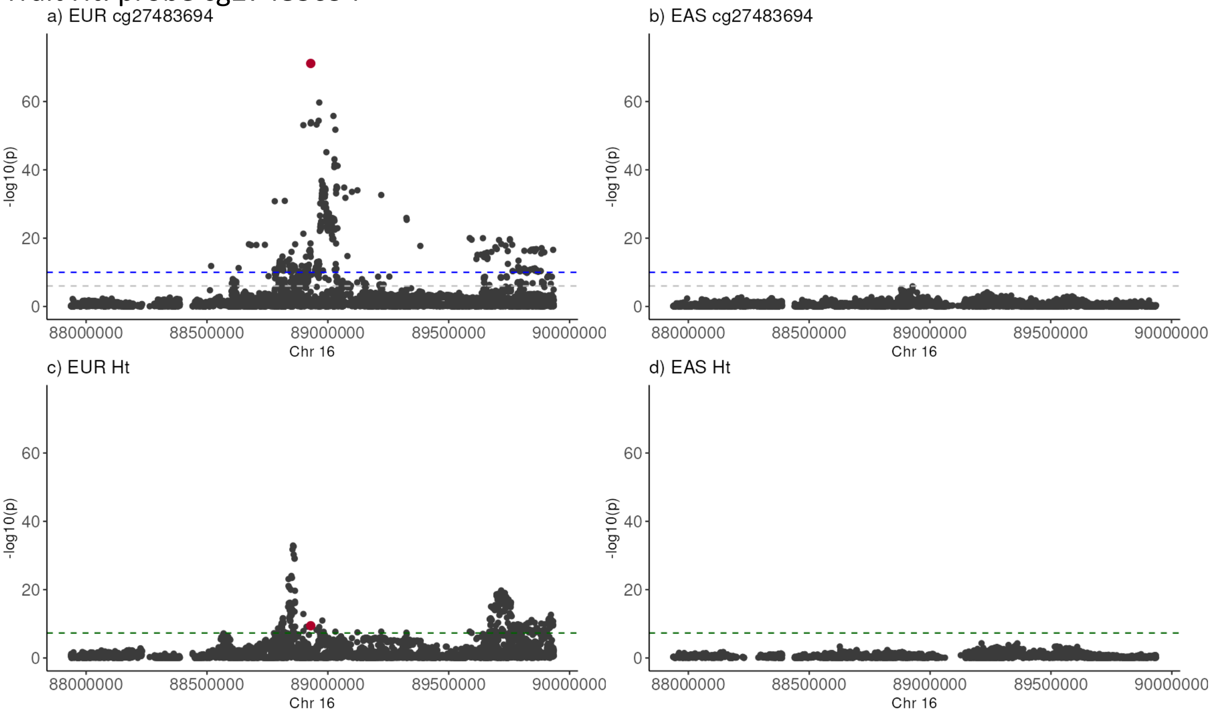

Trait Ht: probe cg21959090

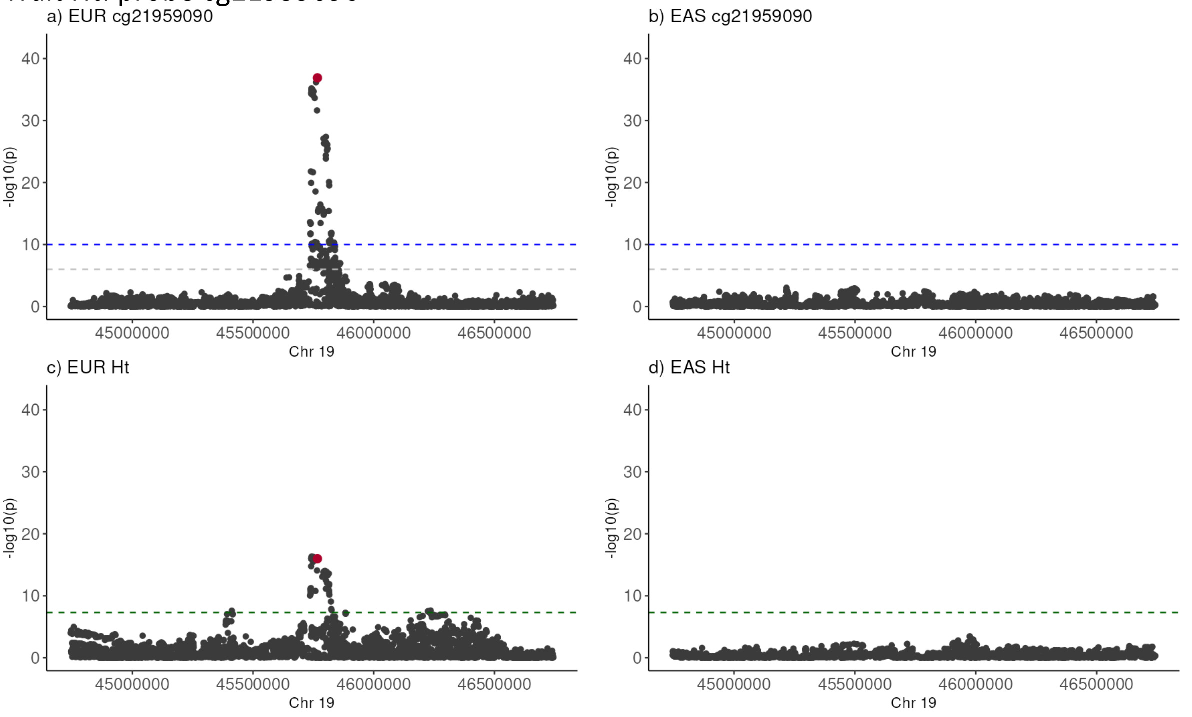

Trait Hypo: probe cg25204543

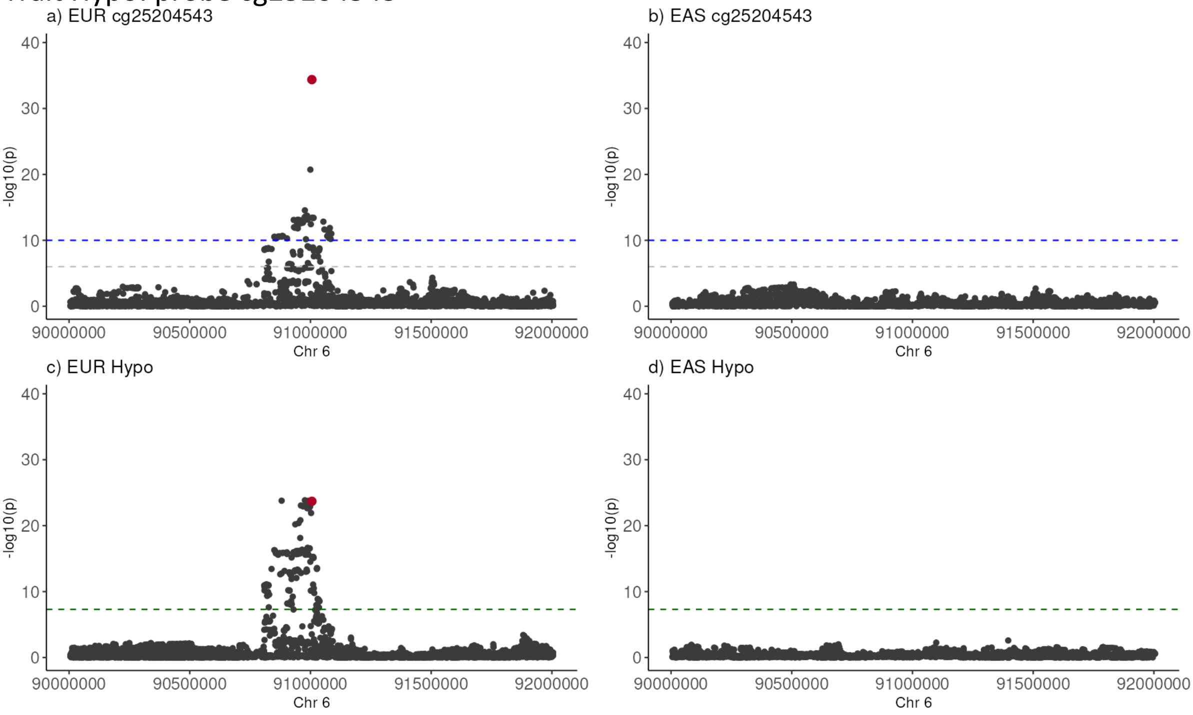

Trait Hypo: probe cg10568624

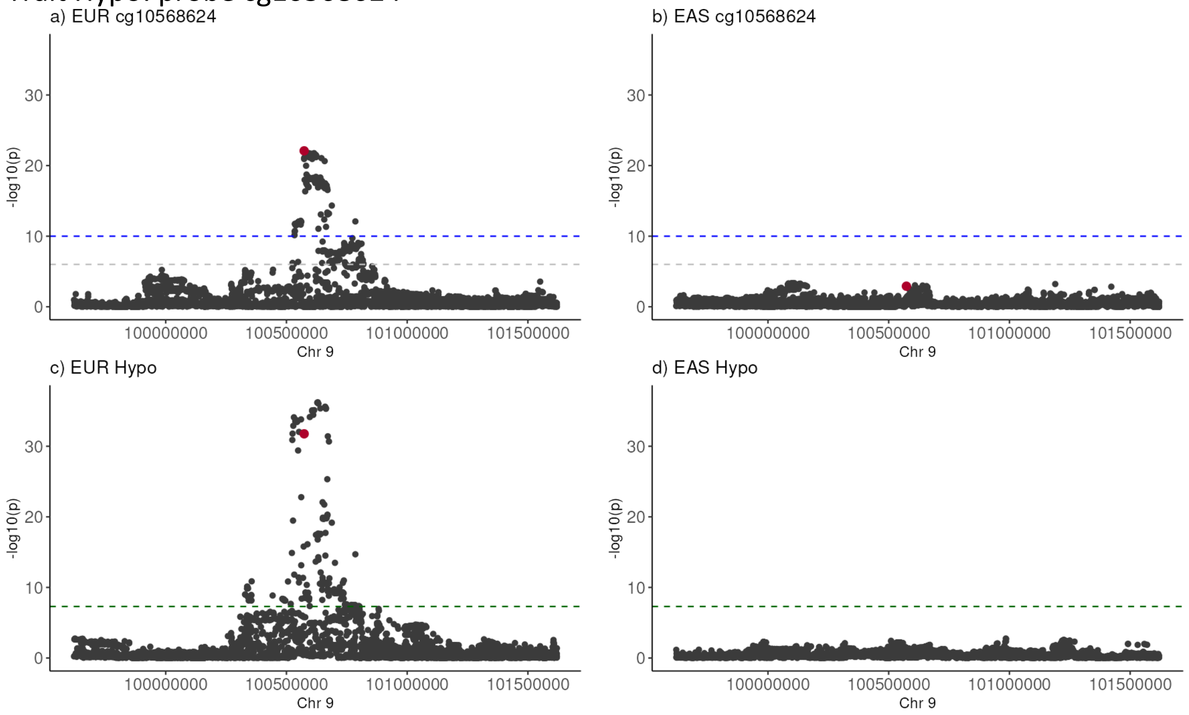

Trait Hypo: probe cg00507154

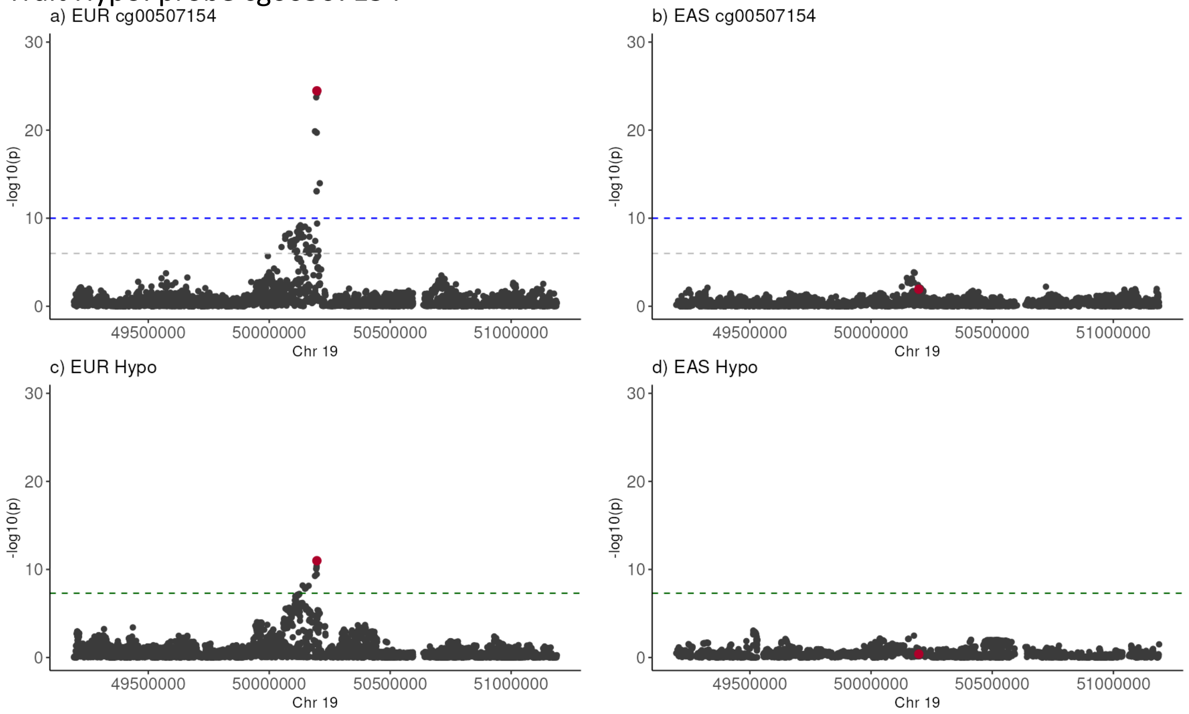

Trait IN: probe cg12351042

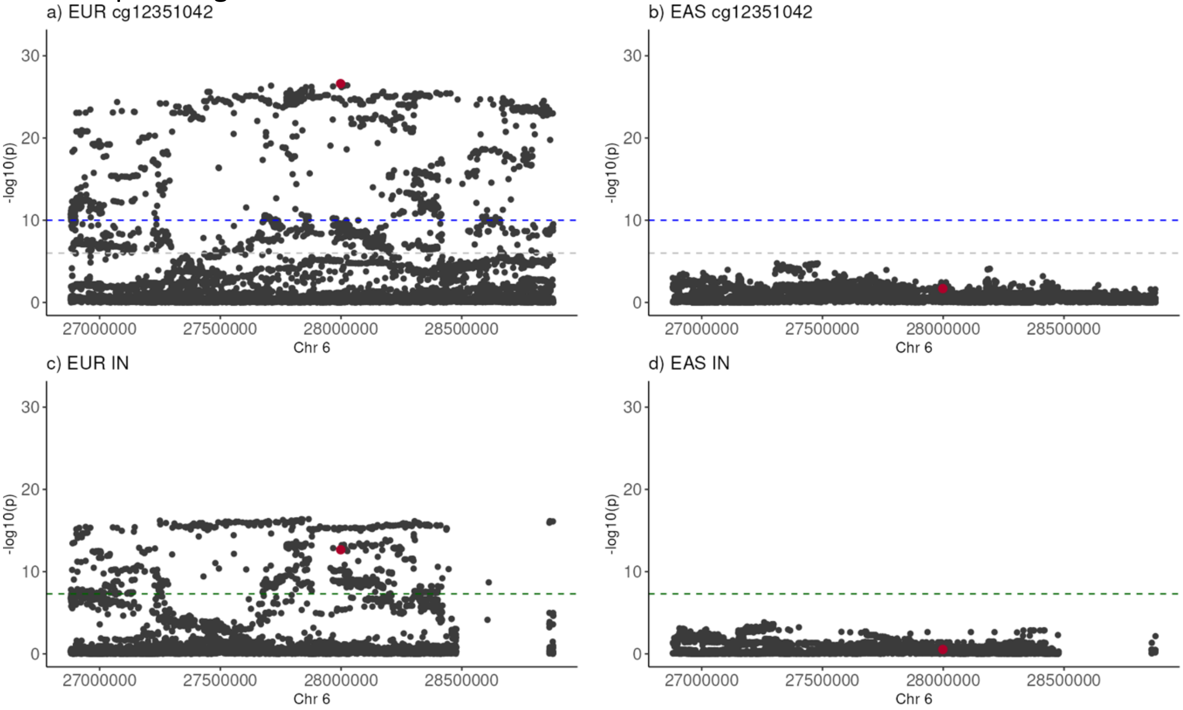

Trait IN: probe cg19084075

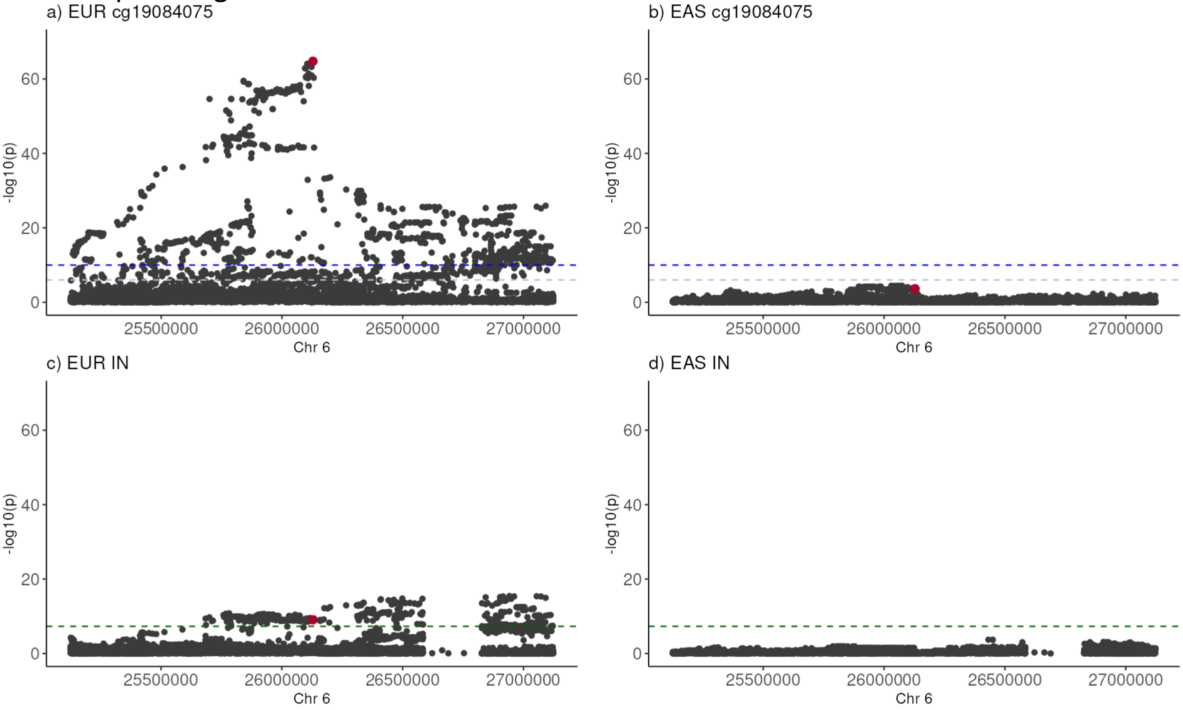

Trait LuC: probe cg13714459

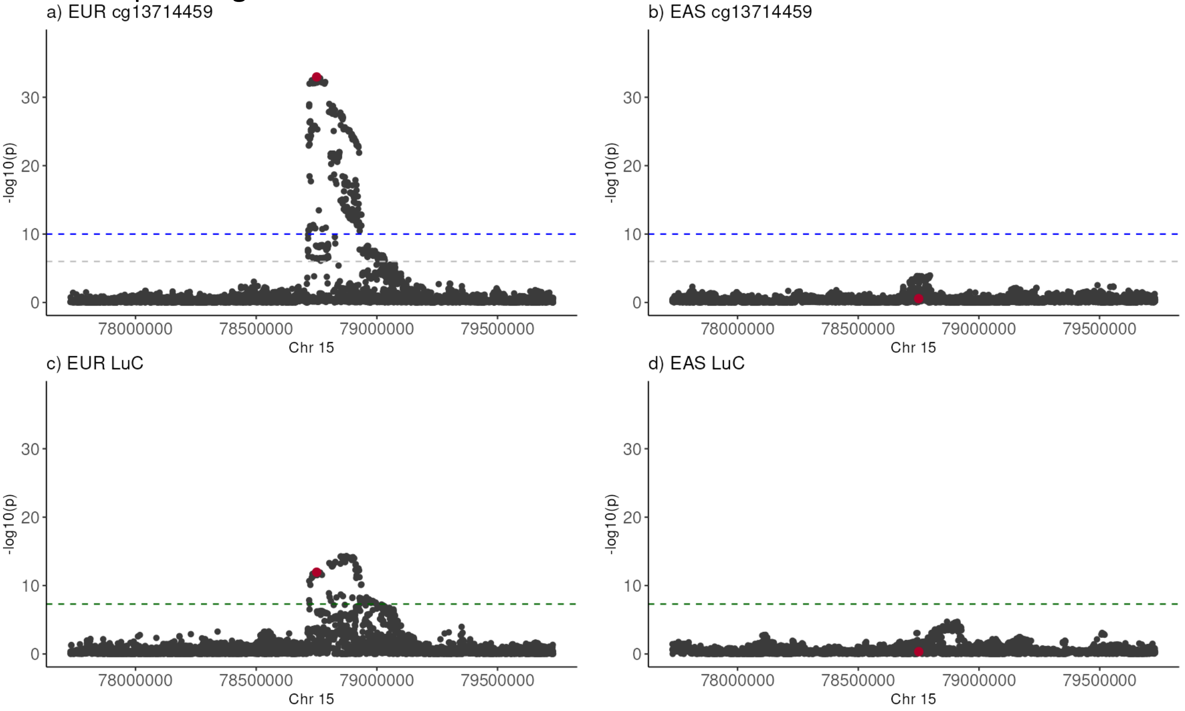

Trait Lym: probe cg20660171

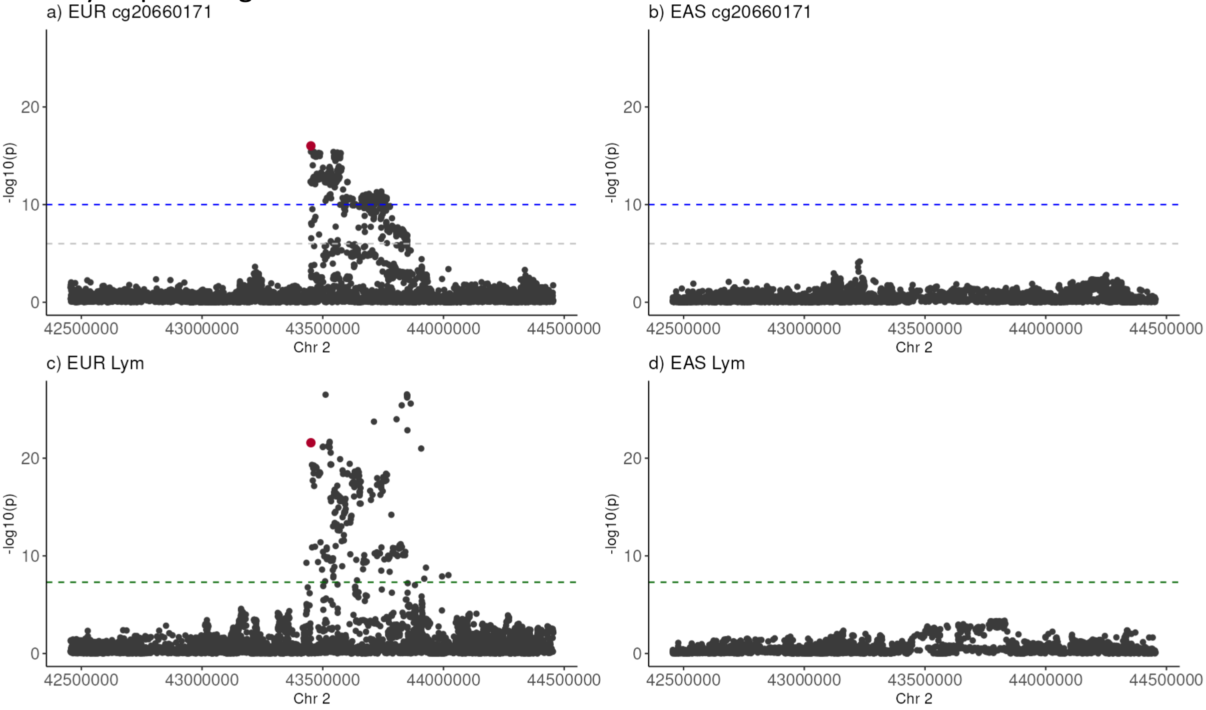

Trait Lym: probe cg21748751

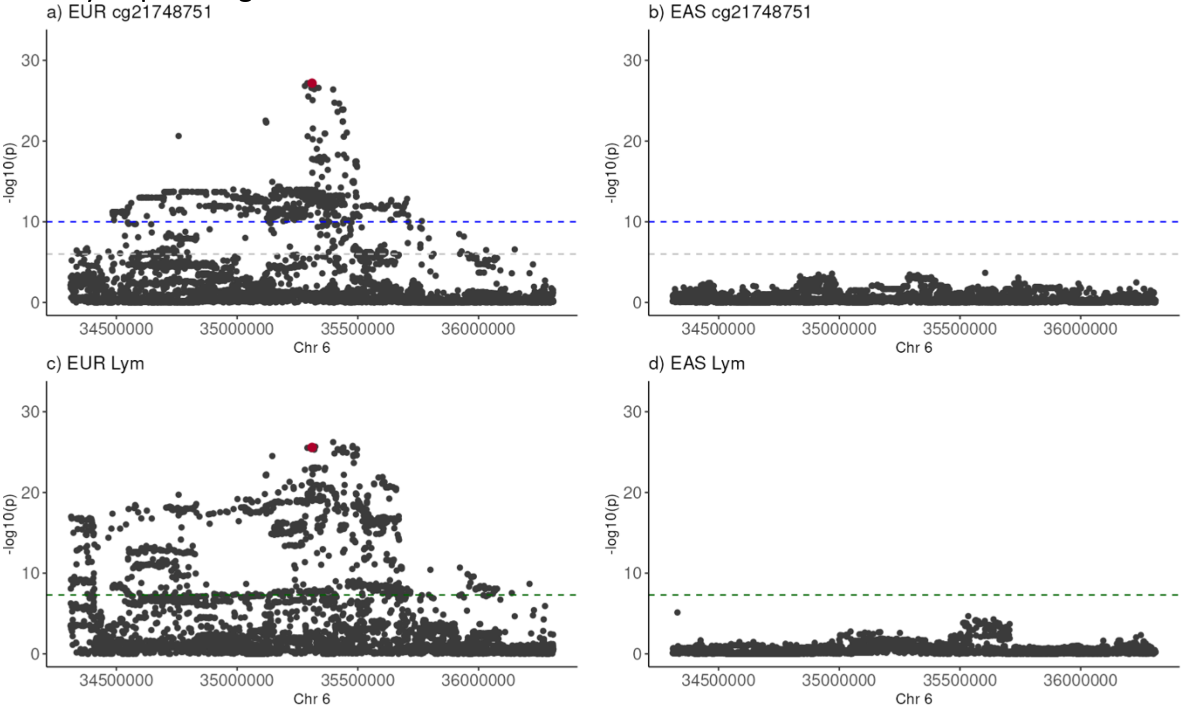

Trait Lym: probe cg25204543

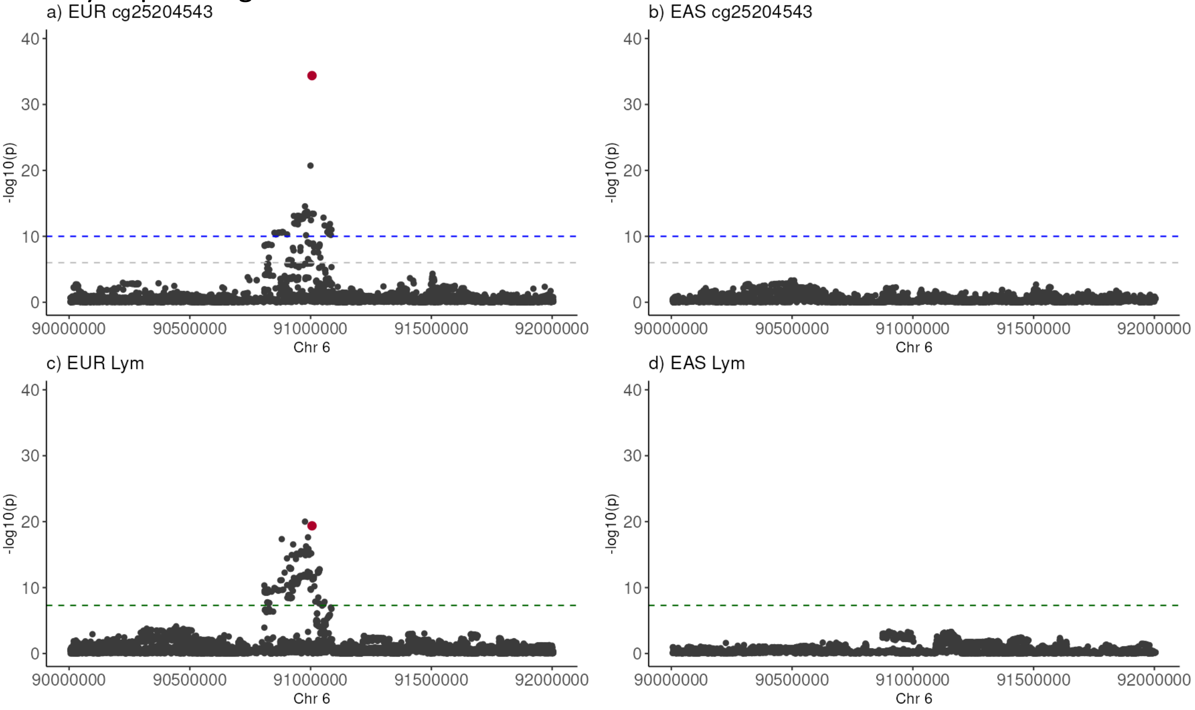

Trait MCV: probe cg13719746

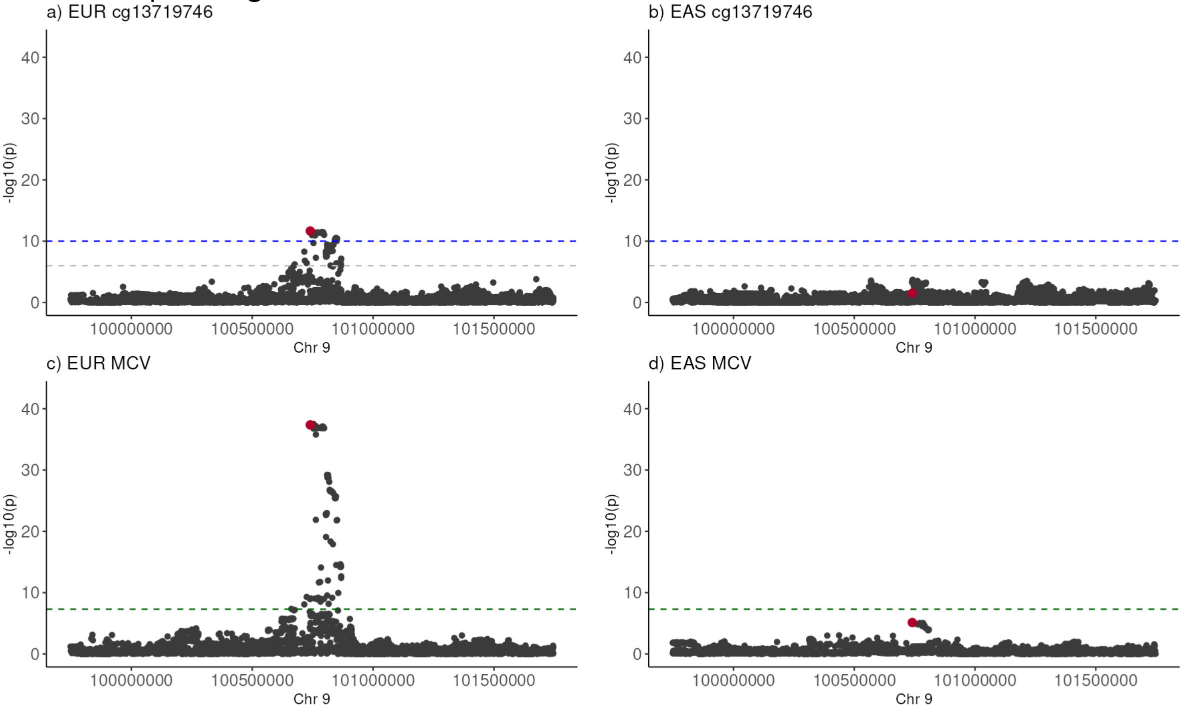

Trait MCV: probe cg07809909

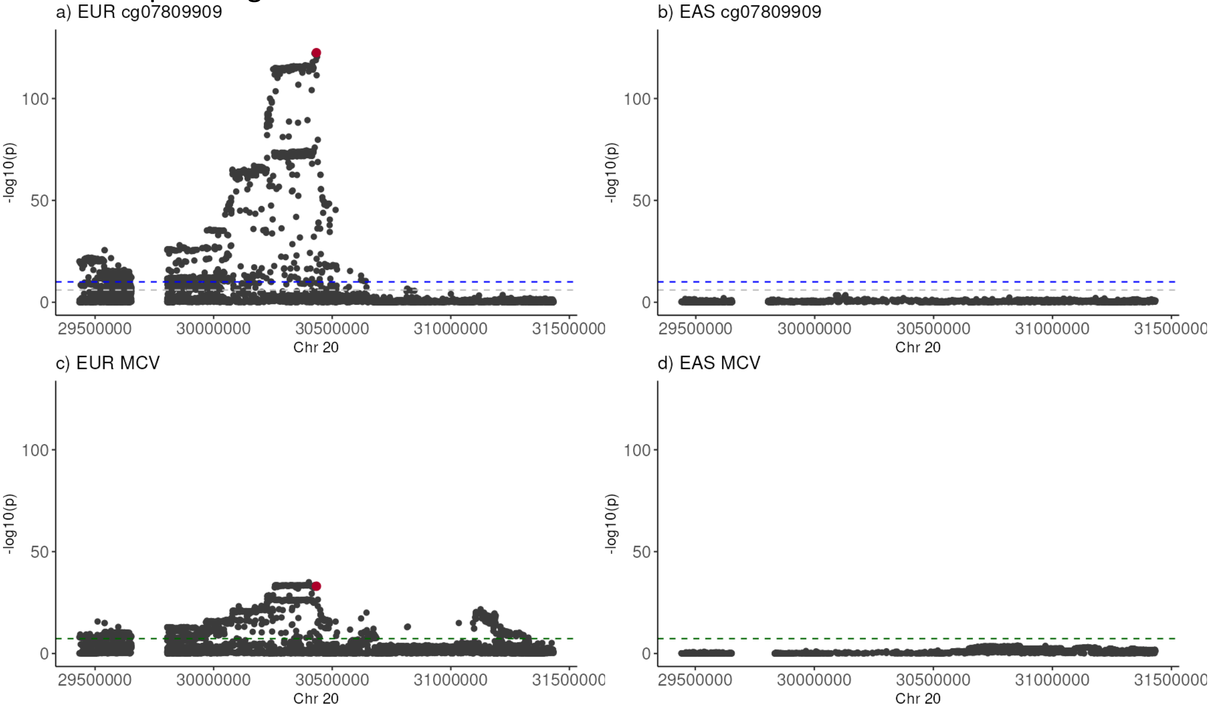

Trait MCV: probe cg11815991

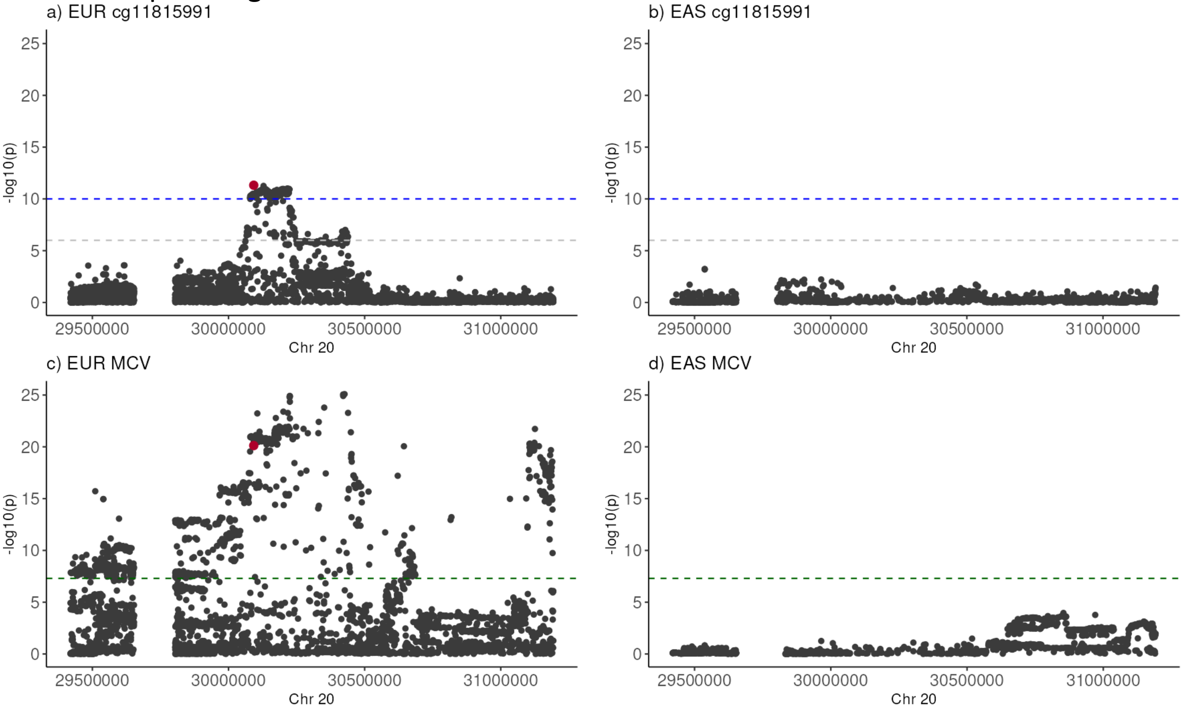

Trait MCV: probe cg12033622

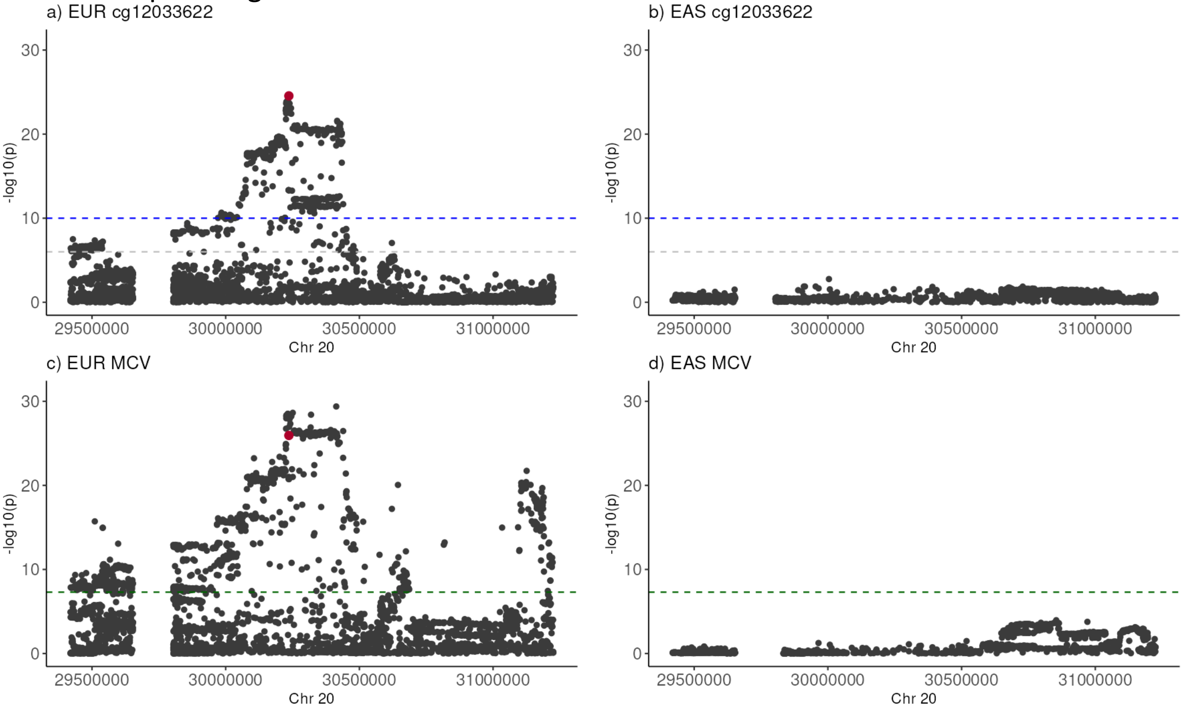

Trait MCV: probe cg26070540

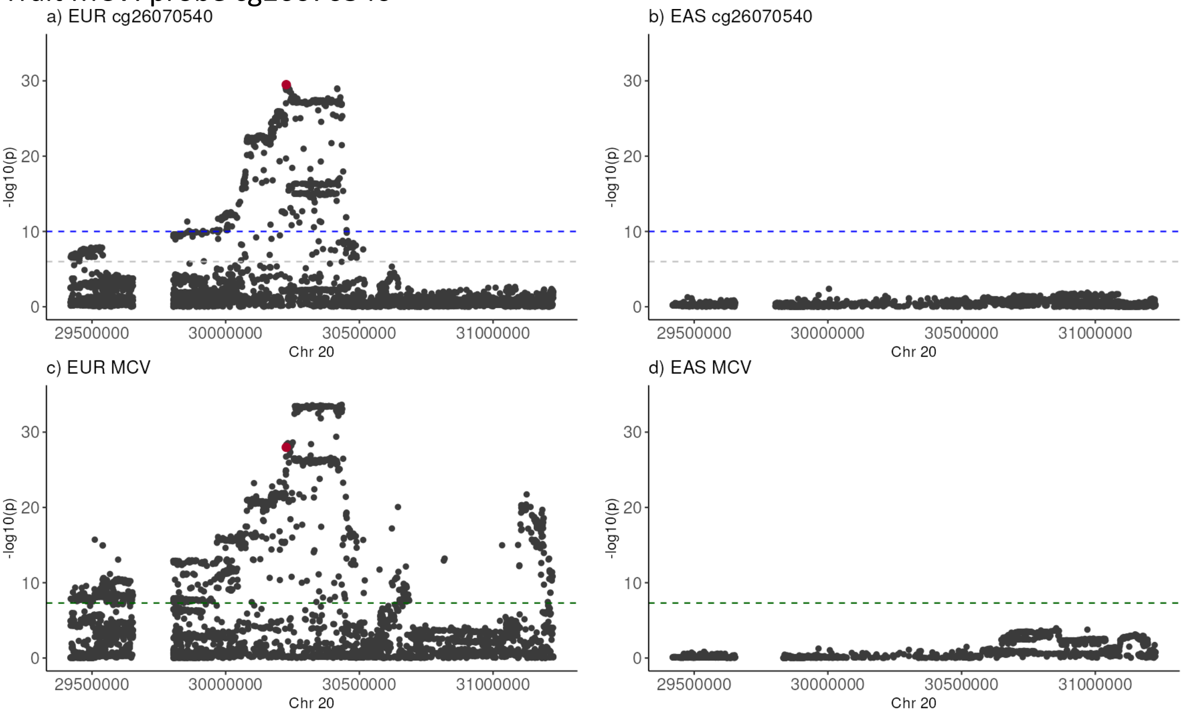

Trait MCV: probe cg01109867

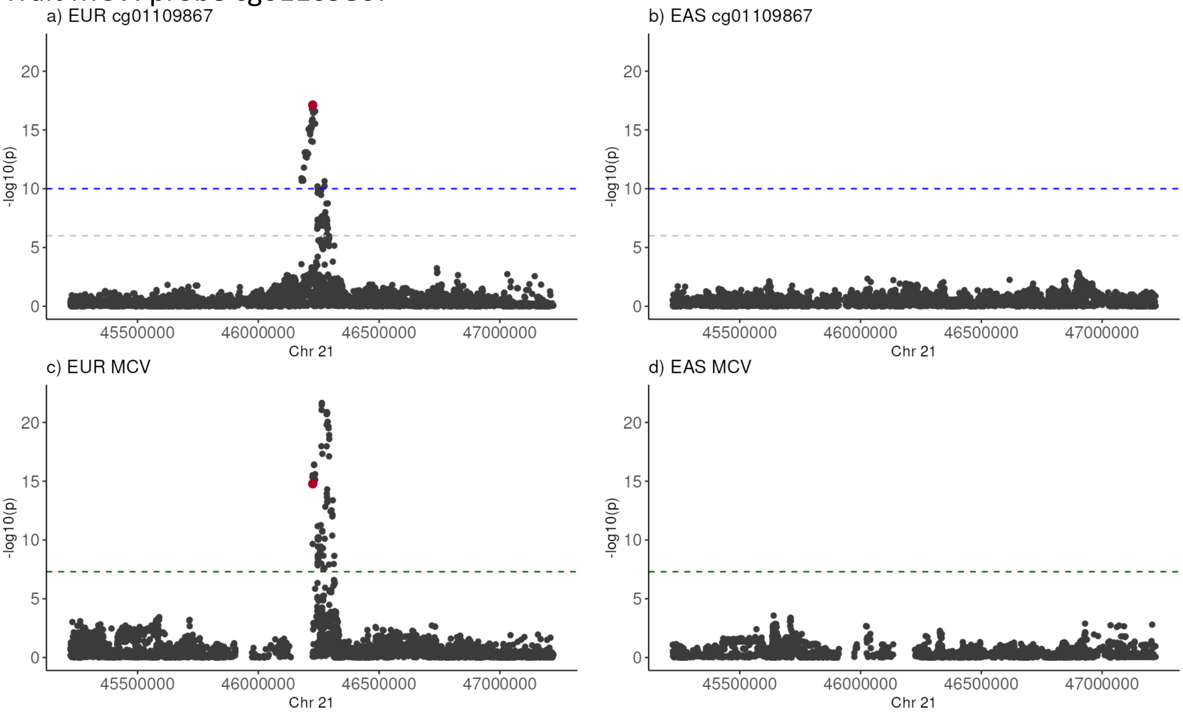

Trait ML: probe cg04387059

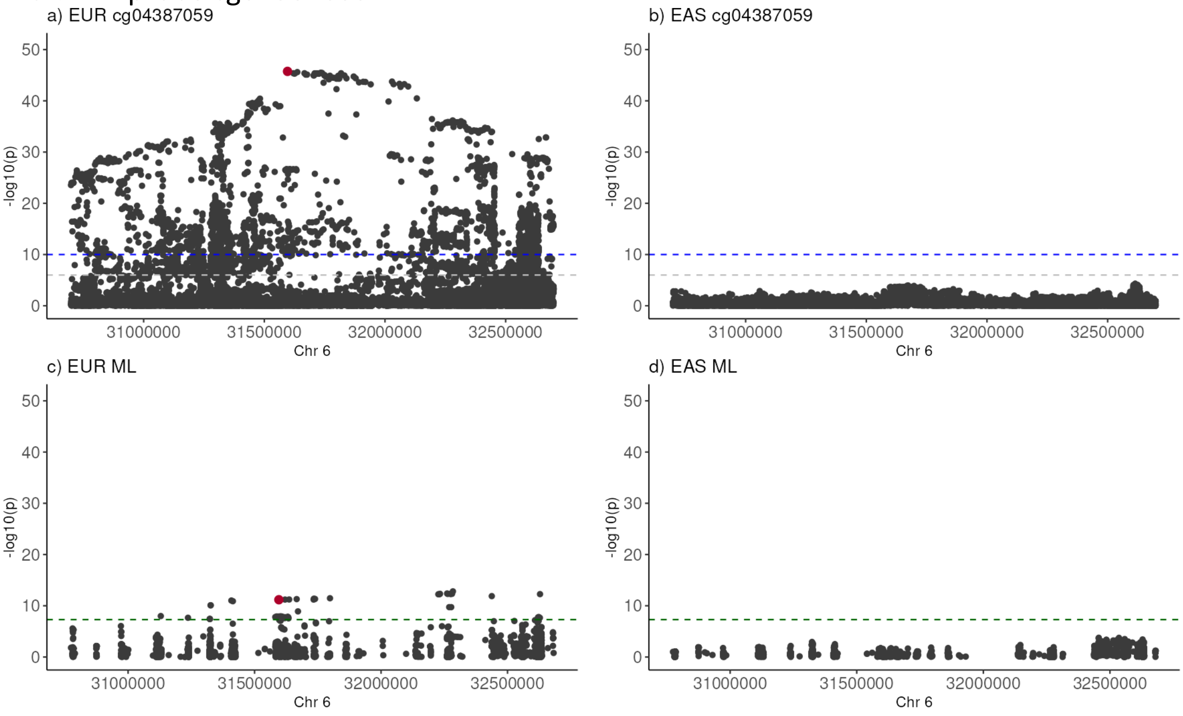

Trait N02BE: probe cg04387059

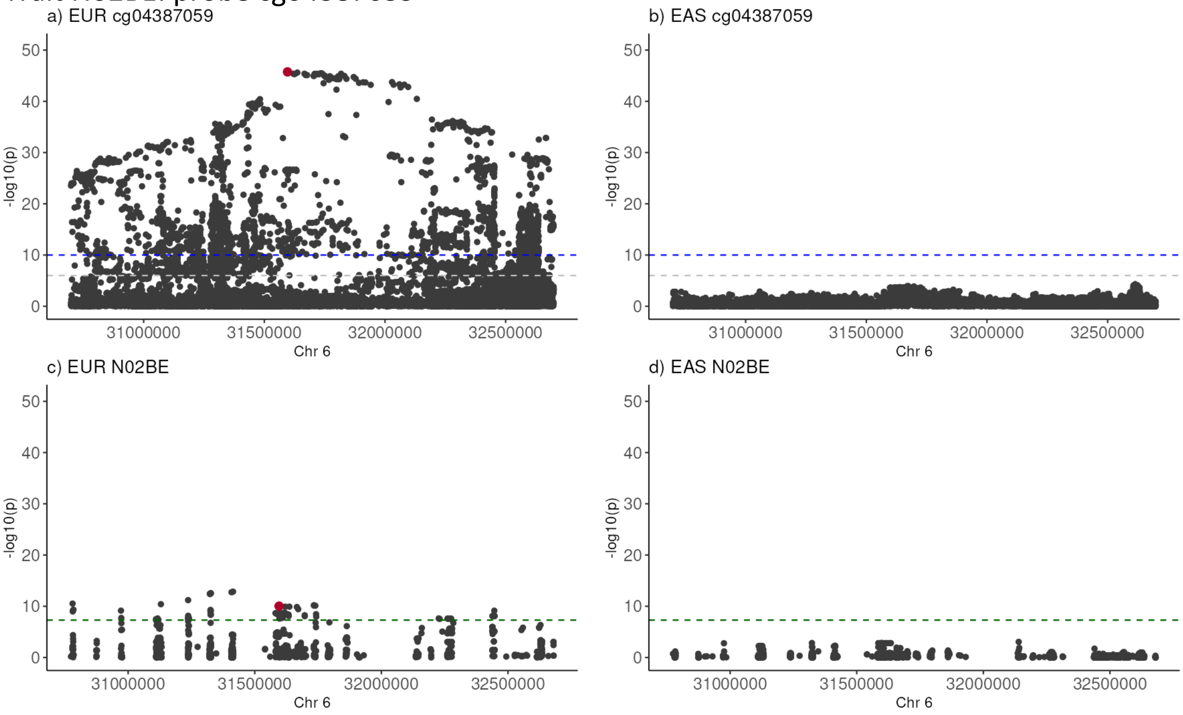

Trait NP: probe cg09611620

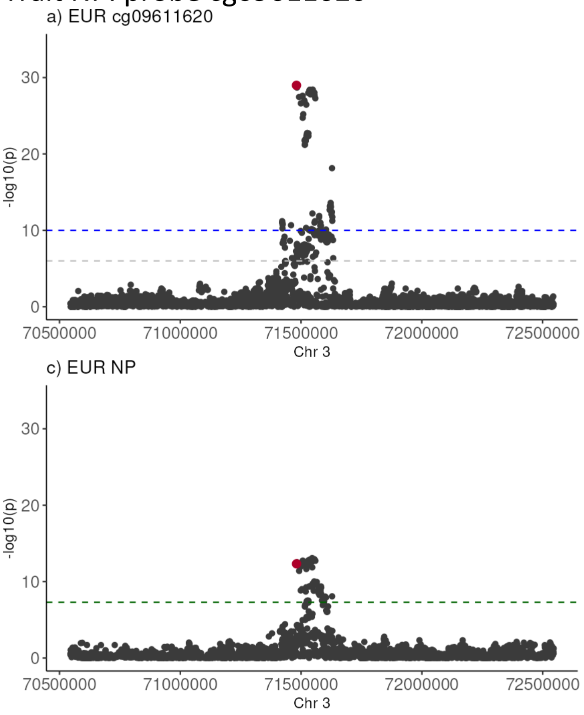

Trait NP: probe cg18318560

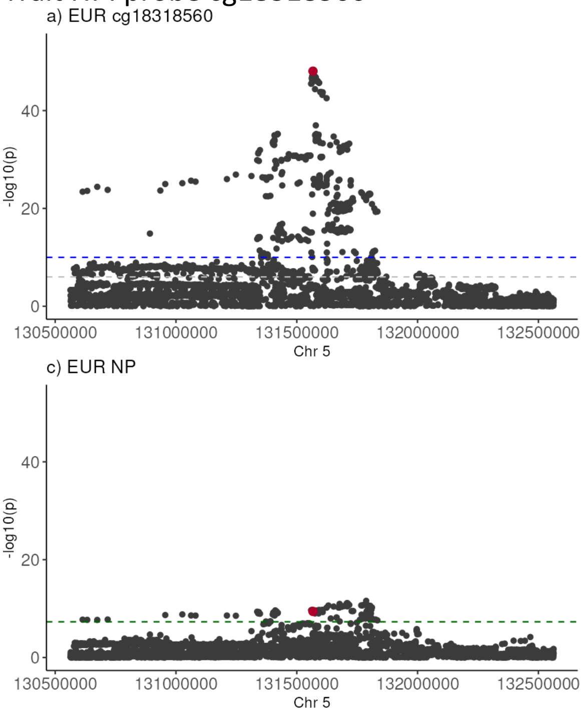

Trait NP: probe cg22598563

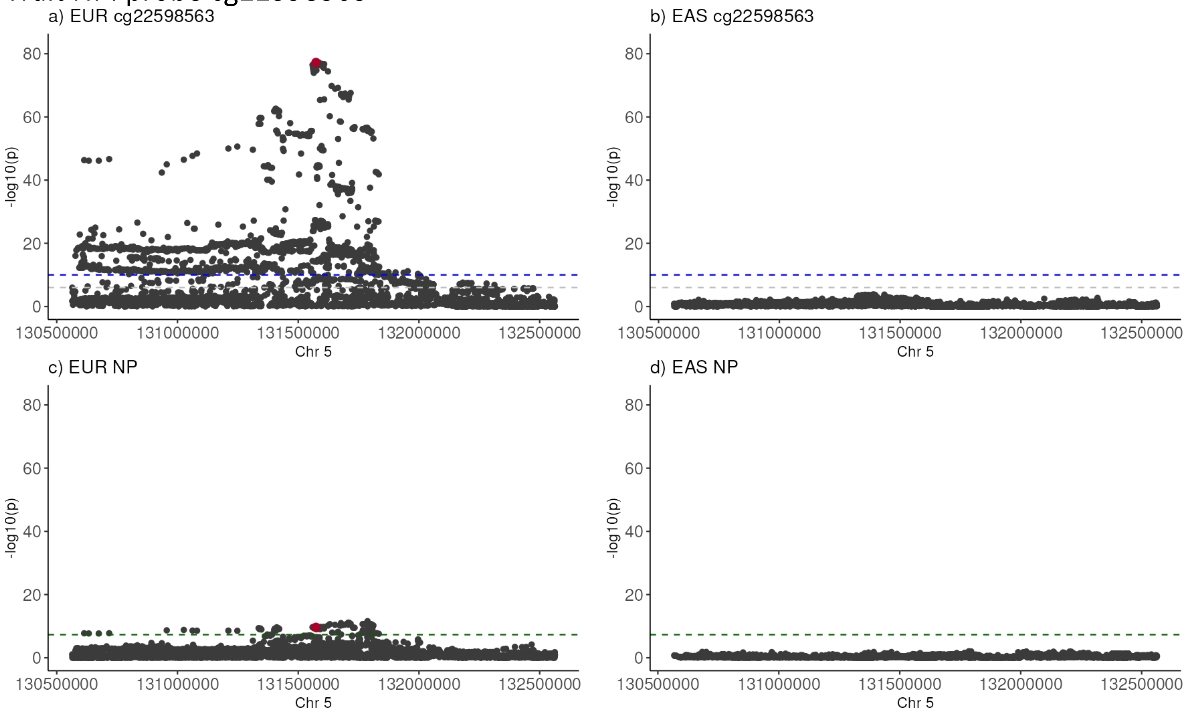

Trait NS: probe cg04387059

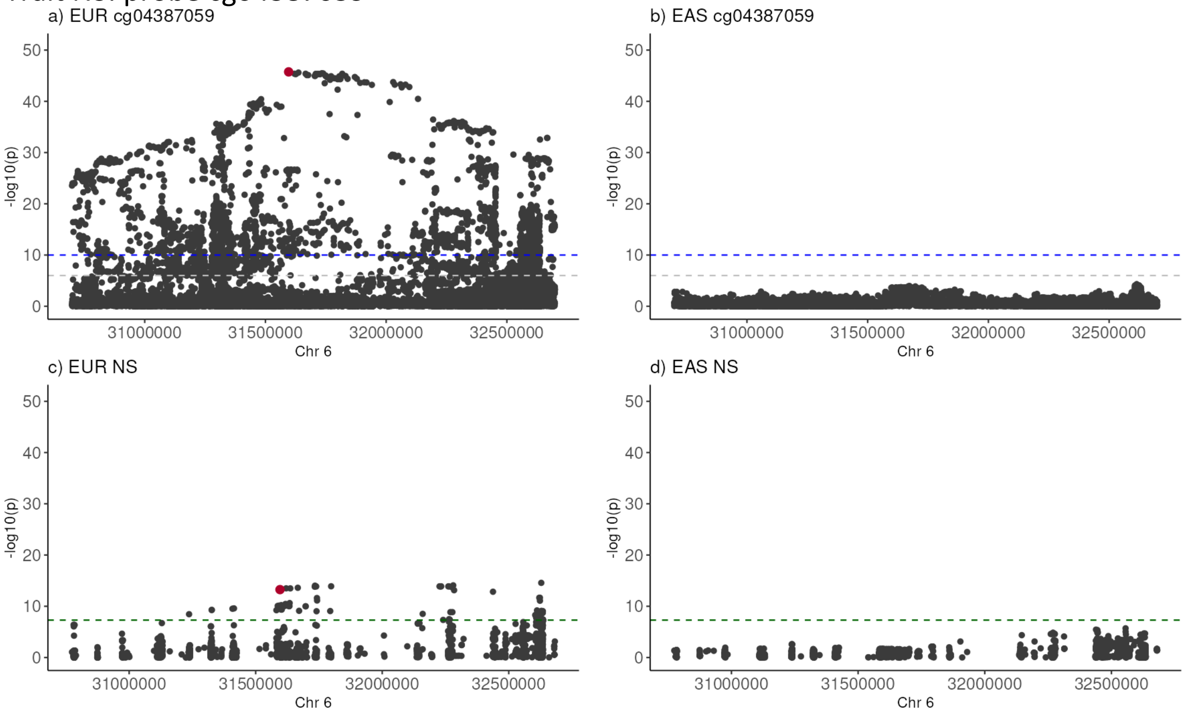

Trait PA: probe cg19197236

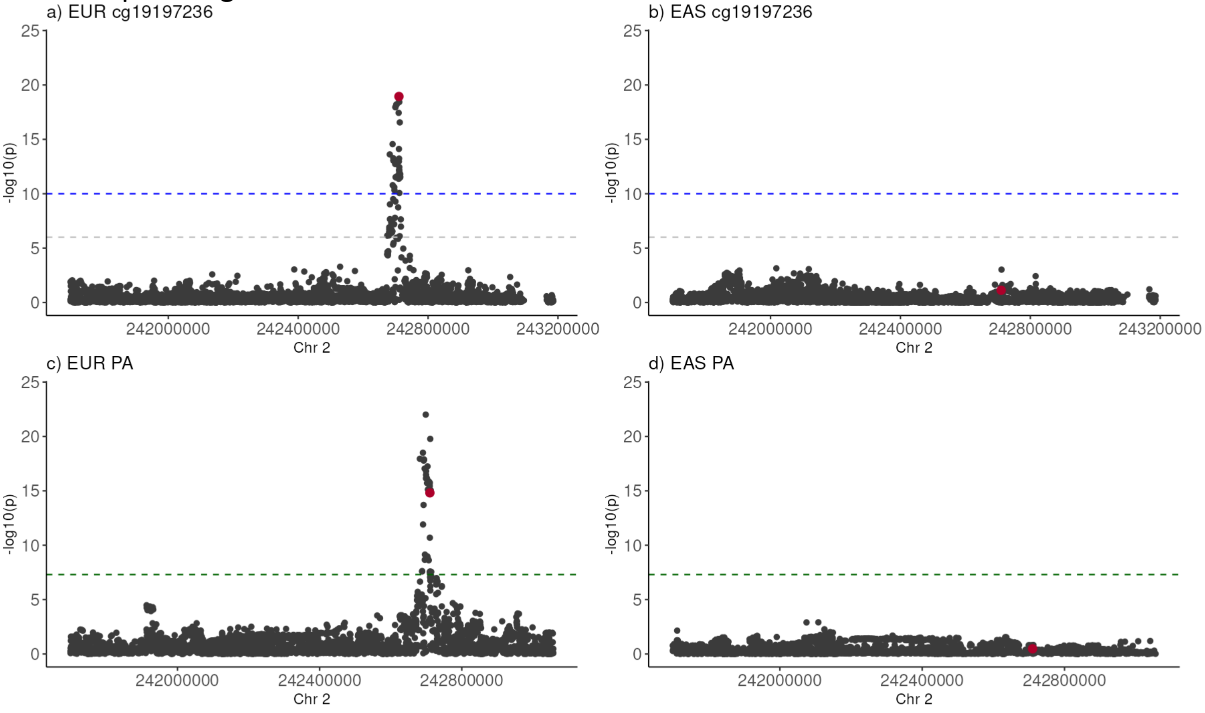

Trait PA: probe cg04387059

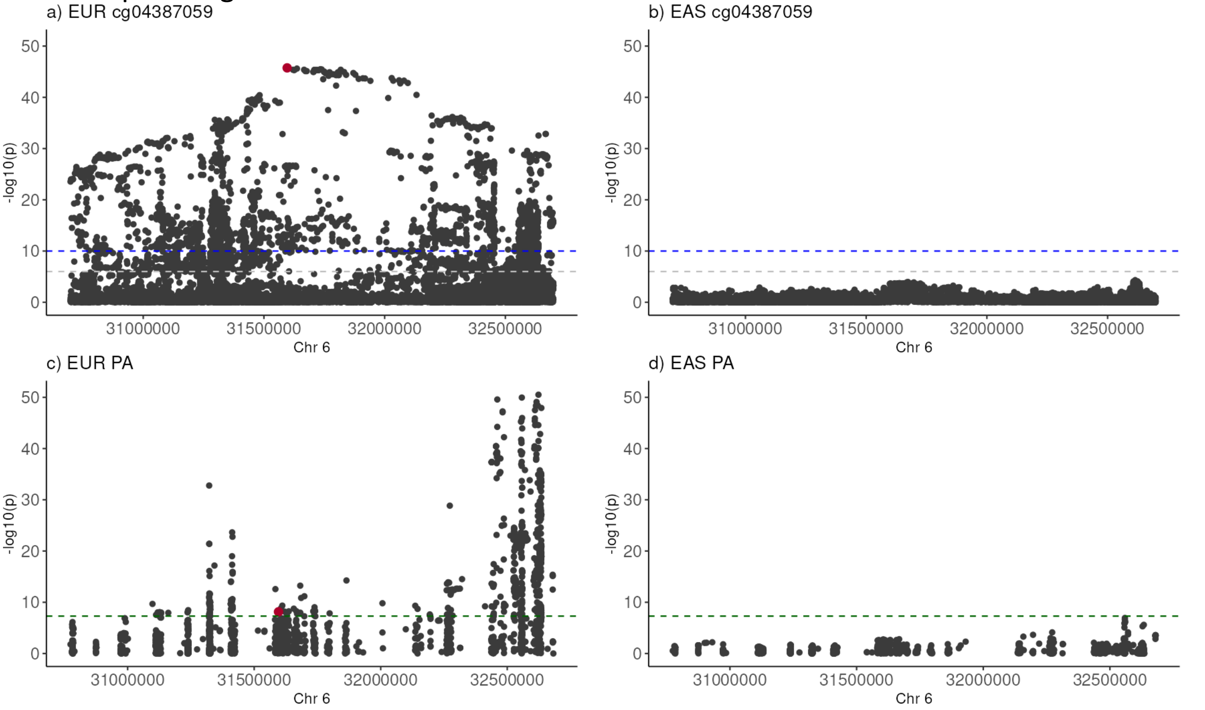

Trait PA: probe cg24032190

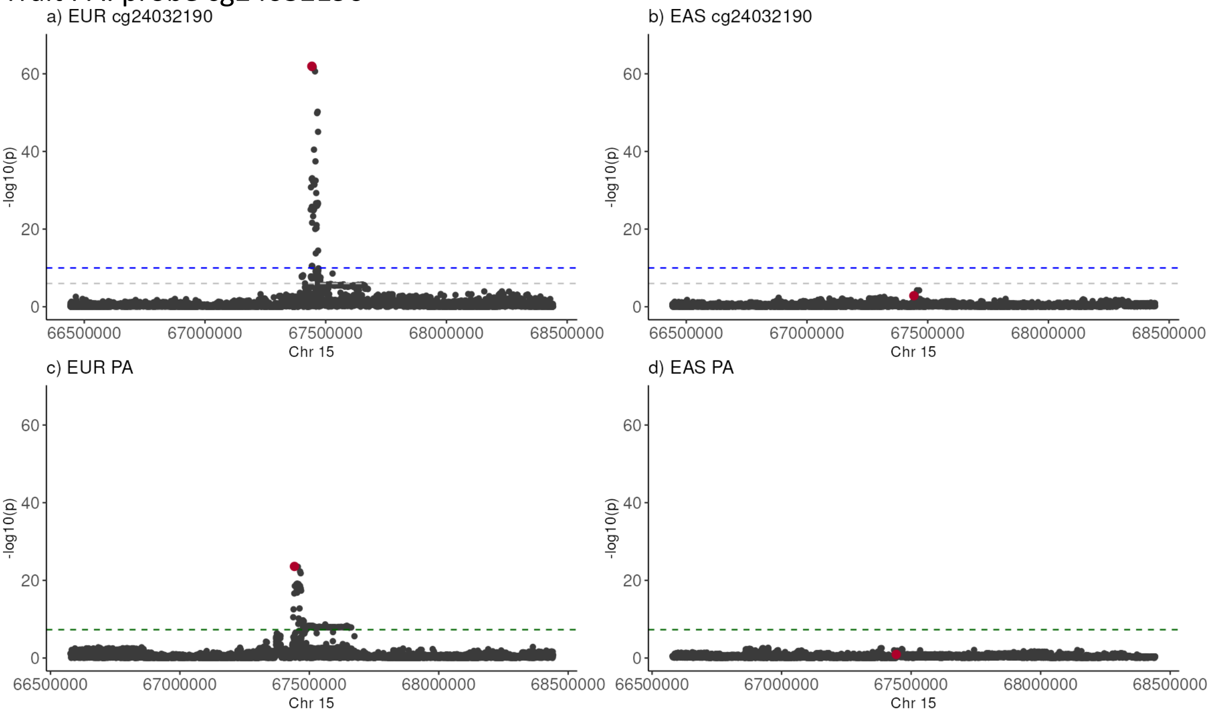

Trait PrC: probe cg08112188

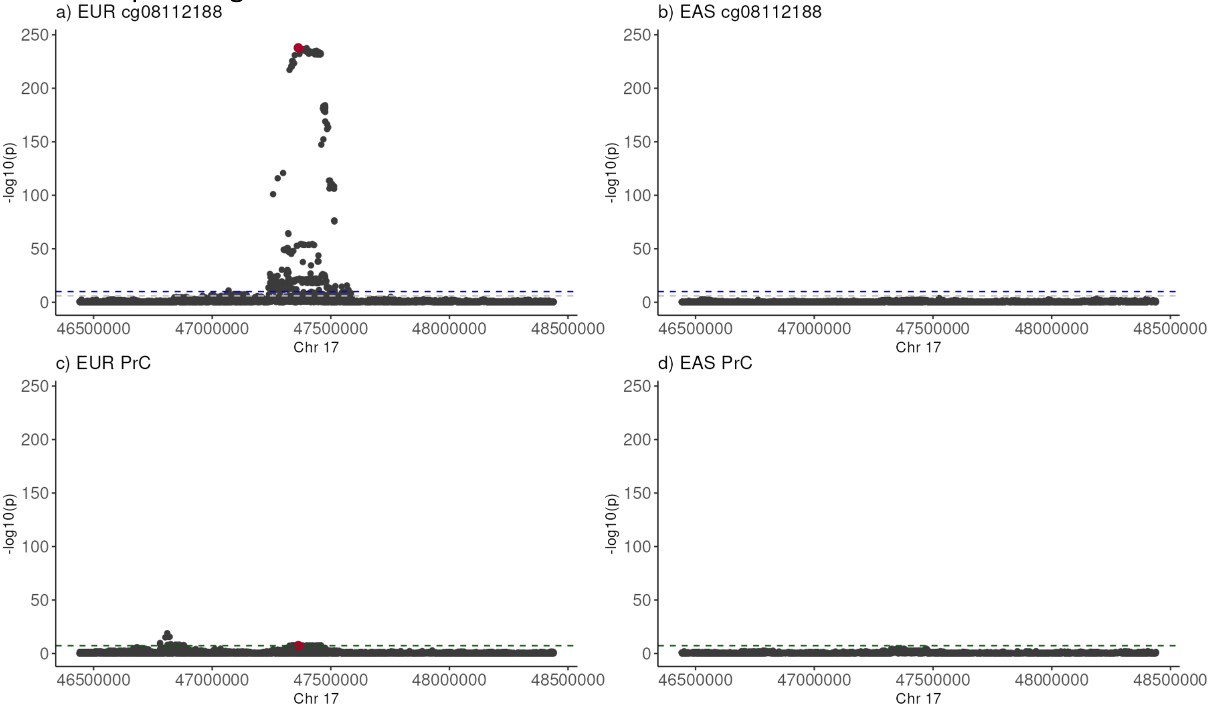

Trait R03A: probe cg19197236

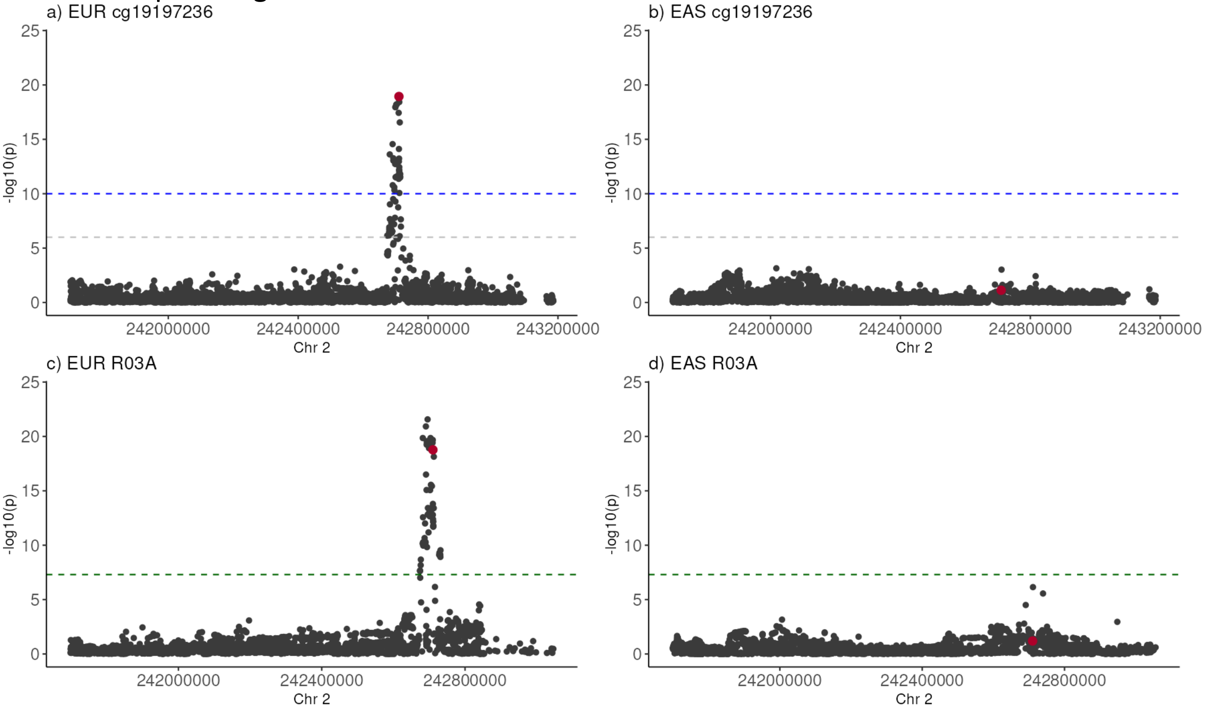

Trait R03A: probe cg18318560

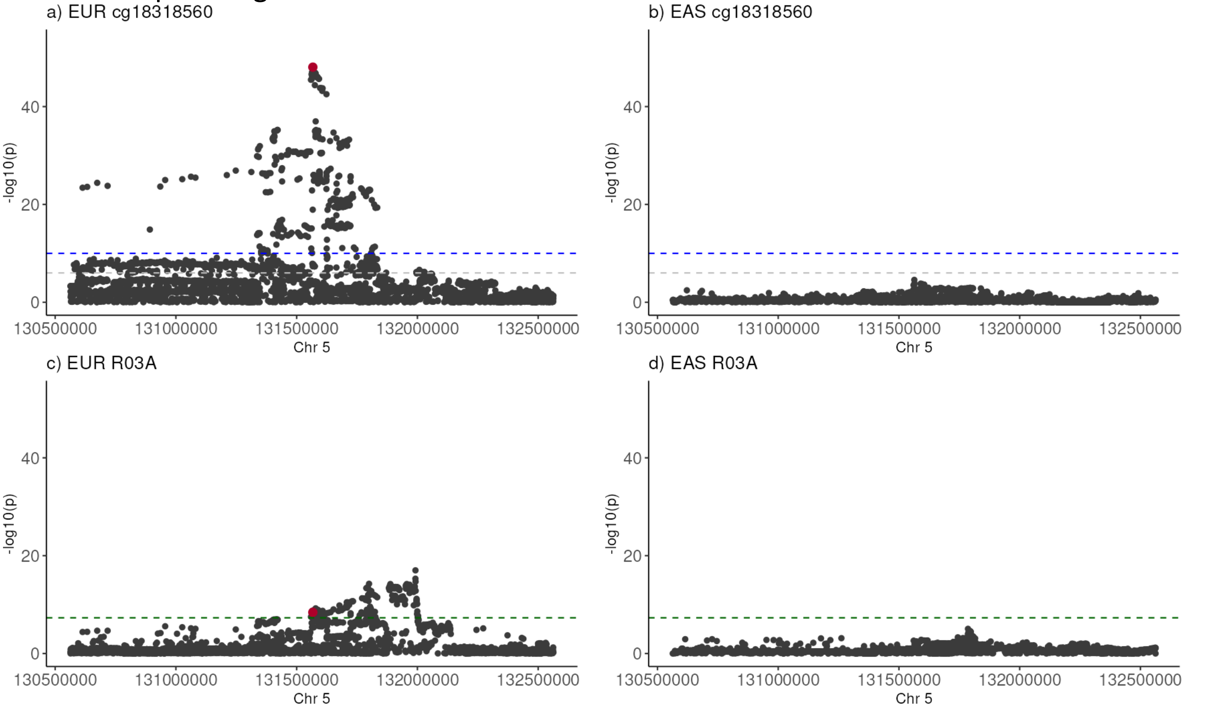

Trait R03A: probe cg25204543

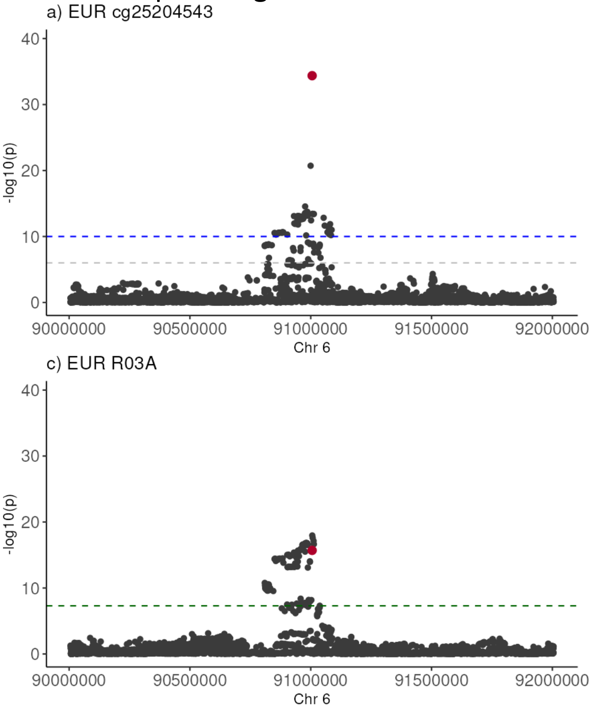

Trait R03A: probe cg24032190

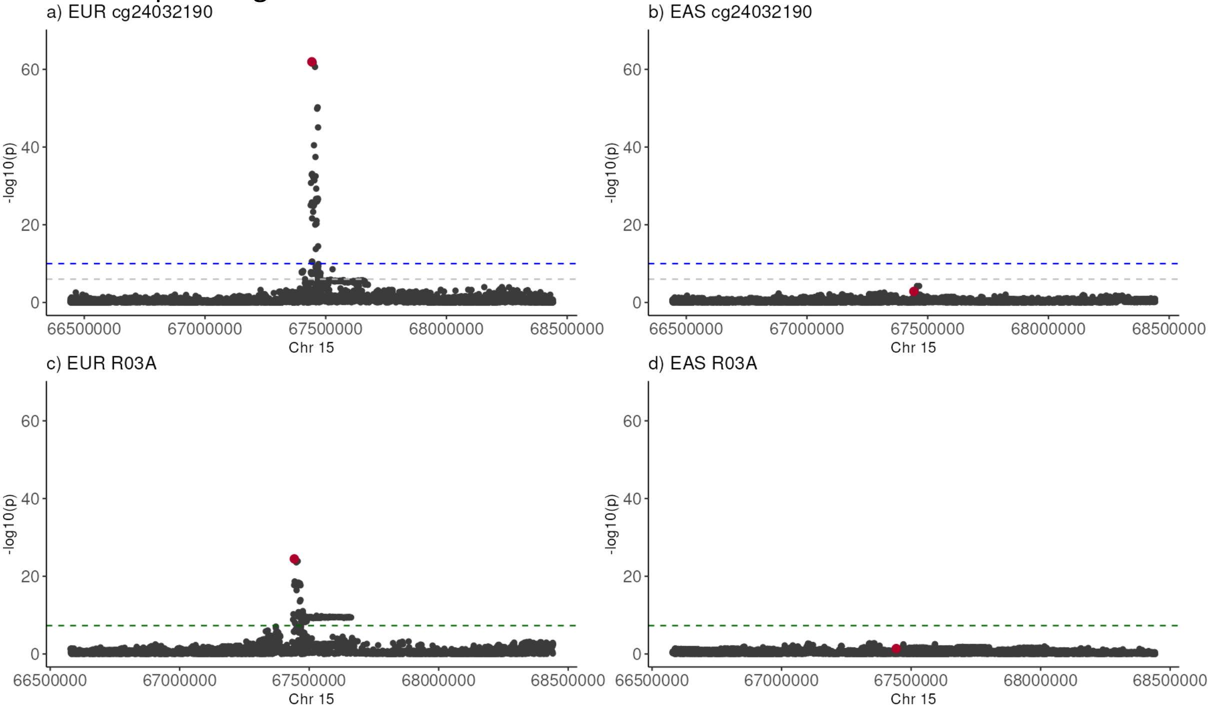

Trait R03A: probe cg15636887

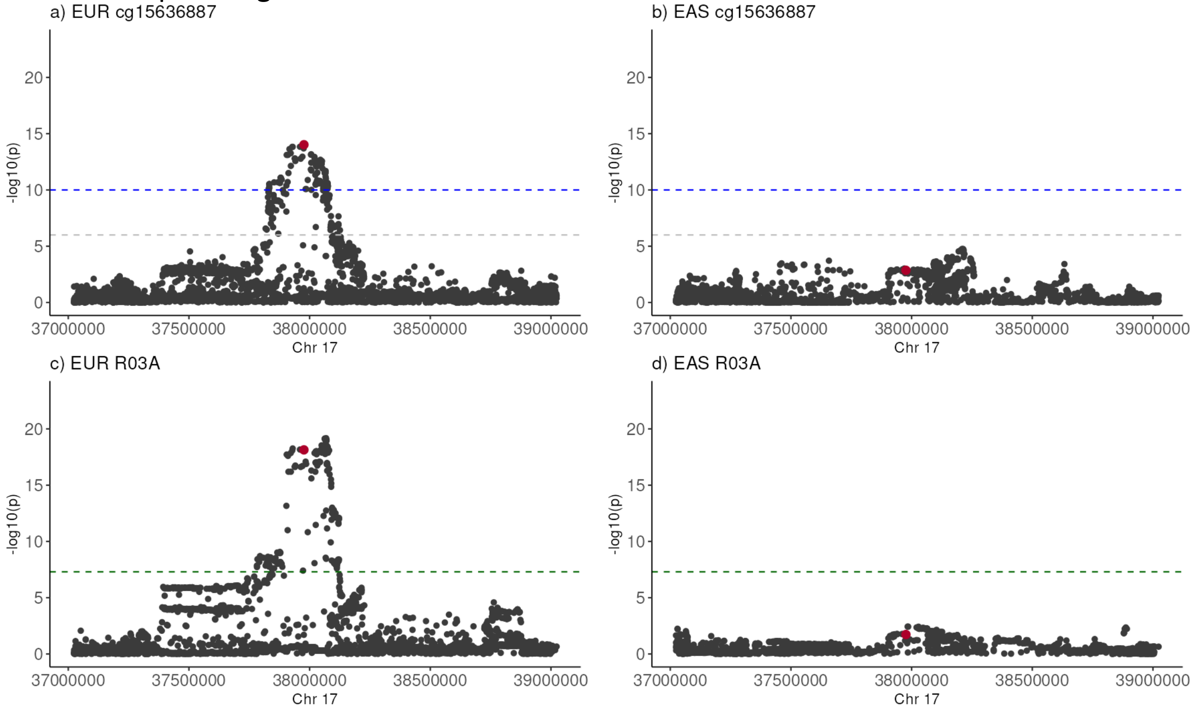

Trait R03BA: probe cg19197236

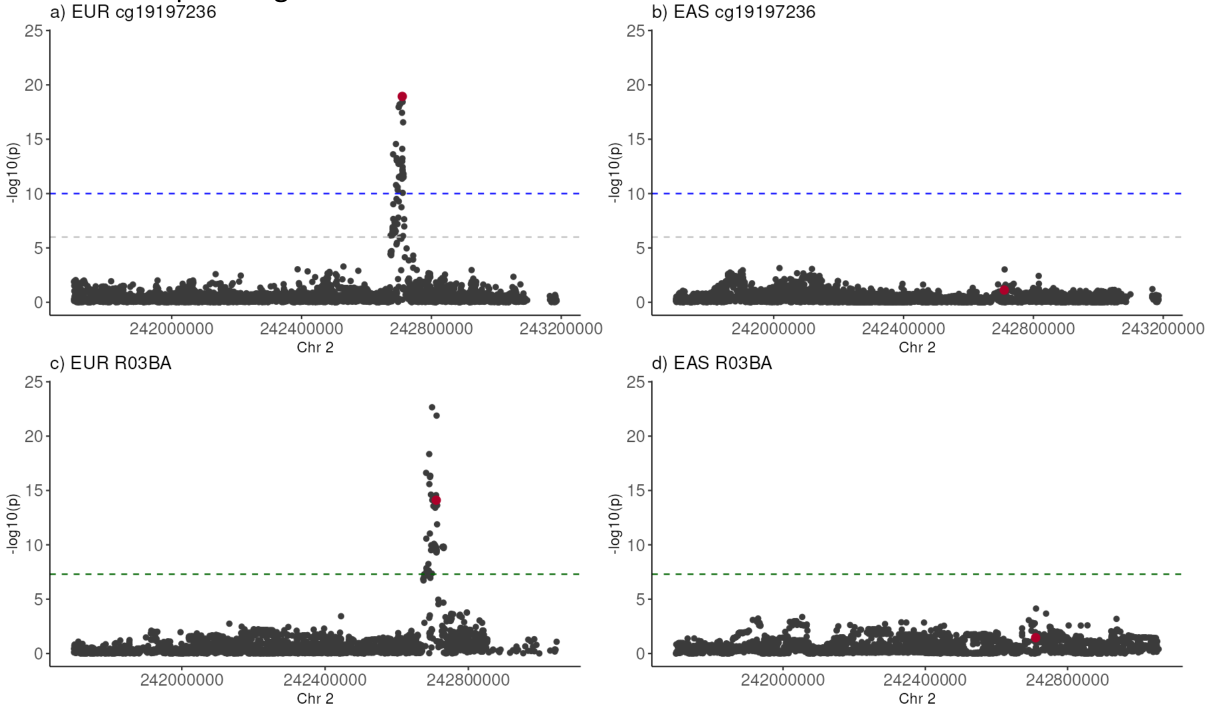

Trait R03BA: probe cg25204543

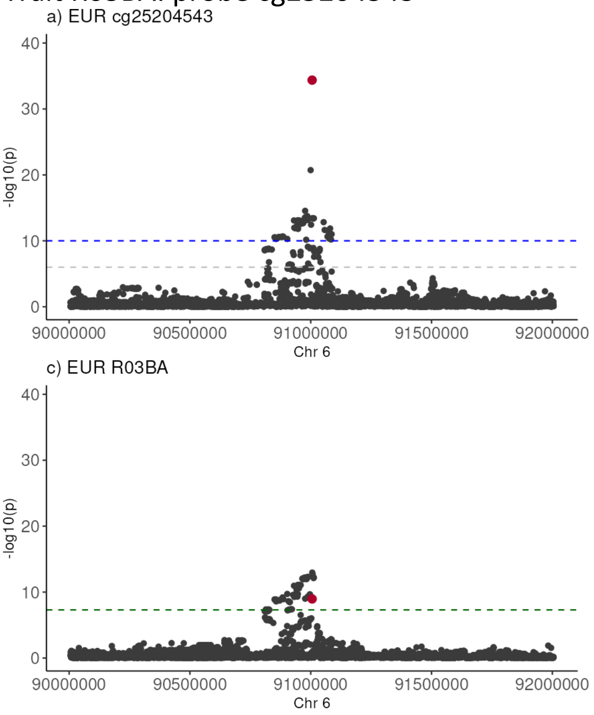

Trait R03BA: probe cg24032190

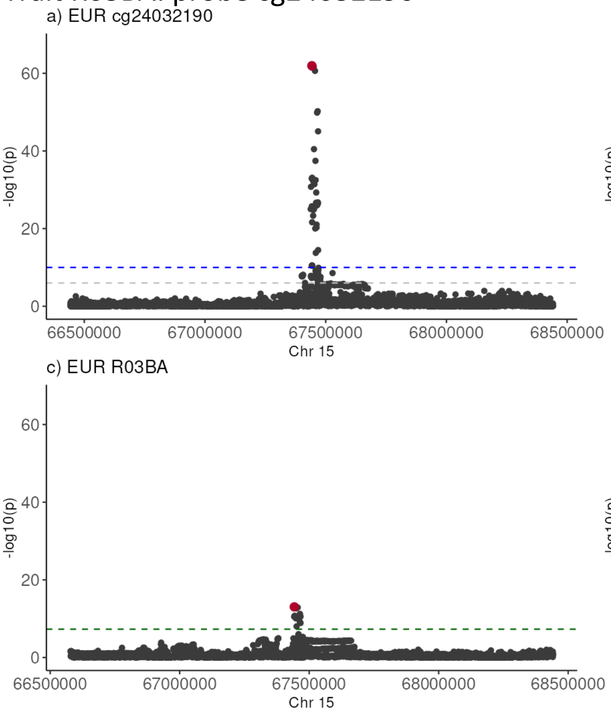

Trait SAP: probe cg12427321

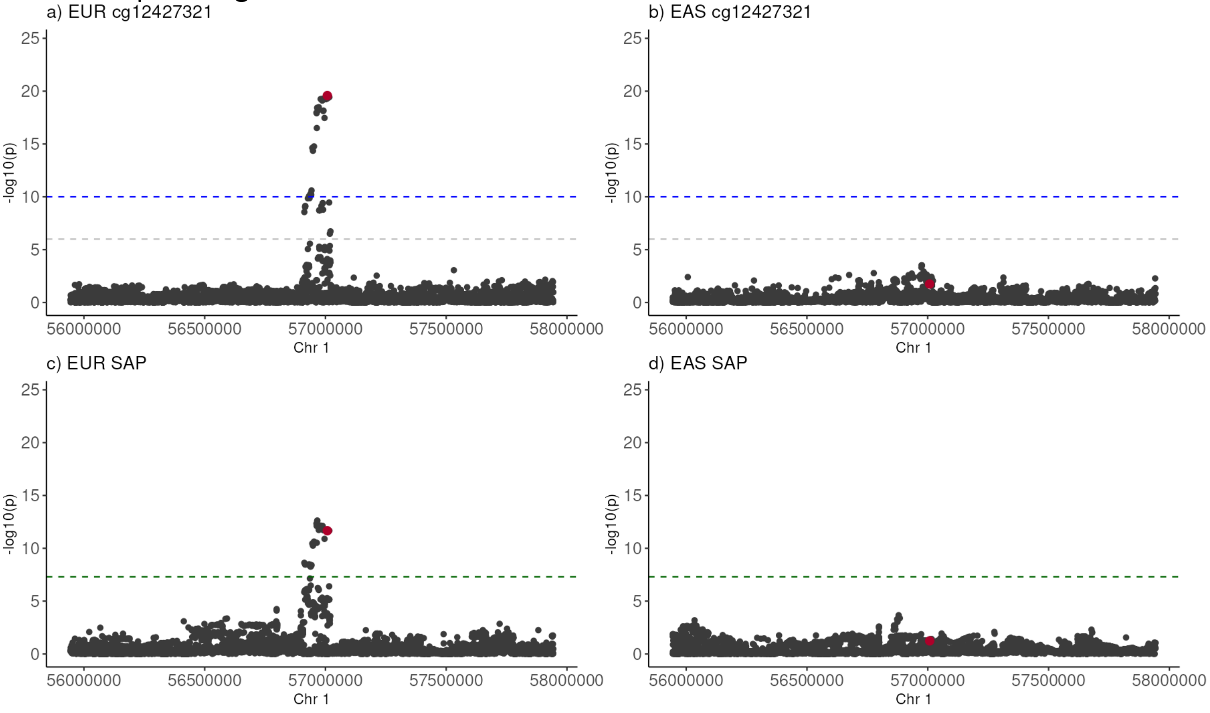

Trait sCr: probe cg16413030

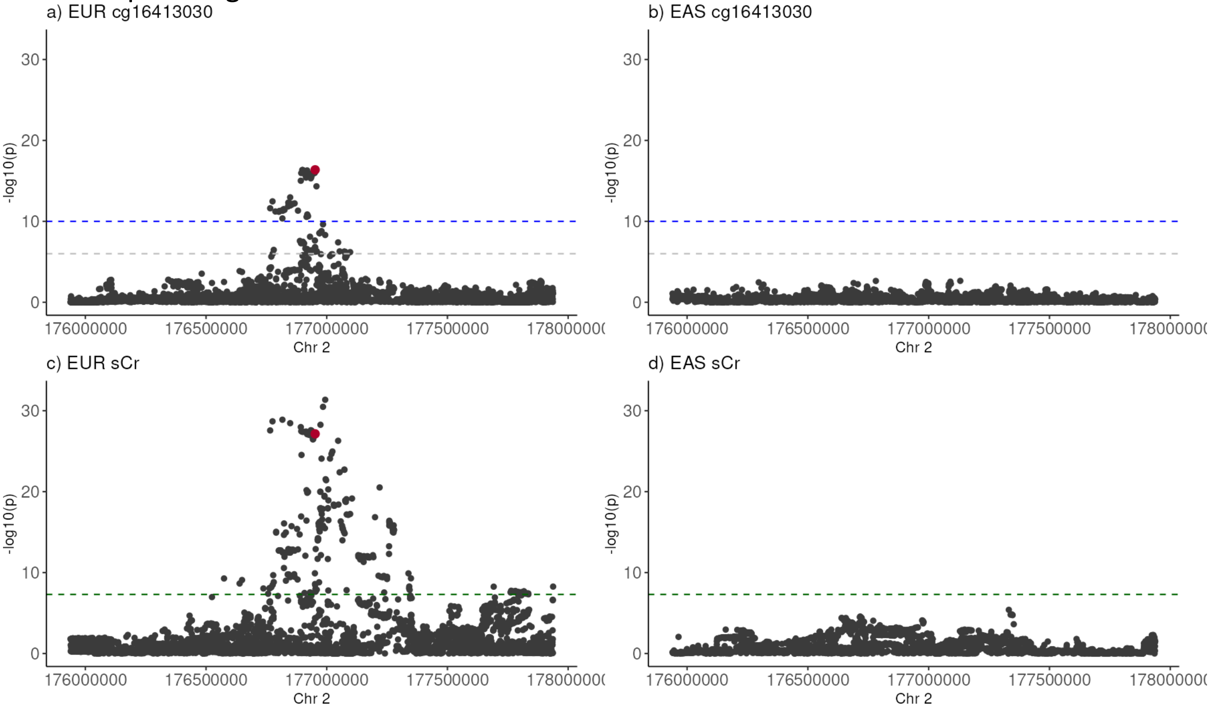

Trait sCr: probe cg15640734

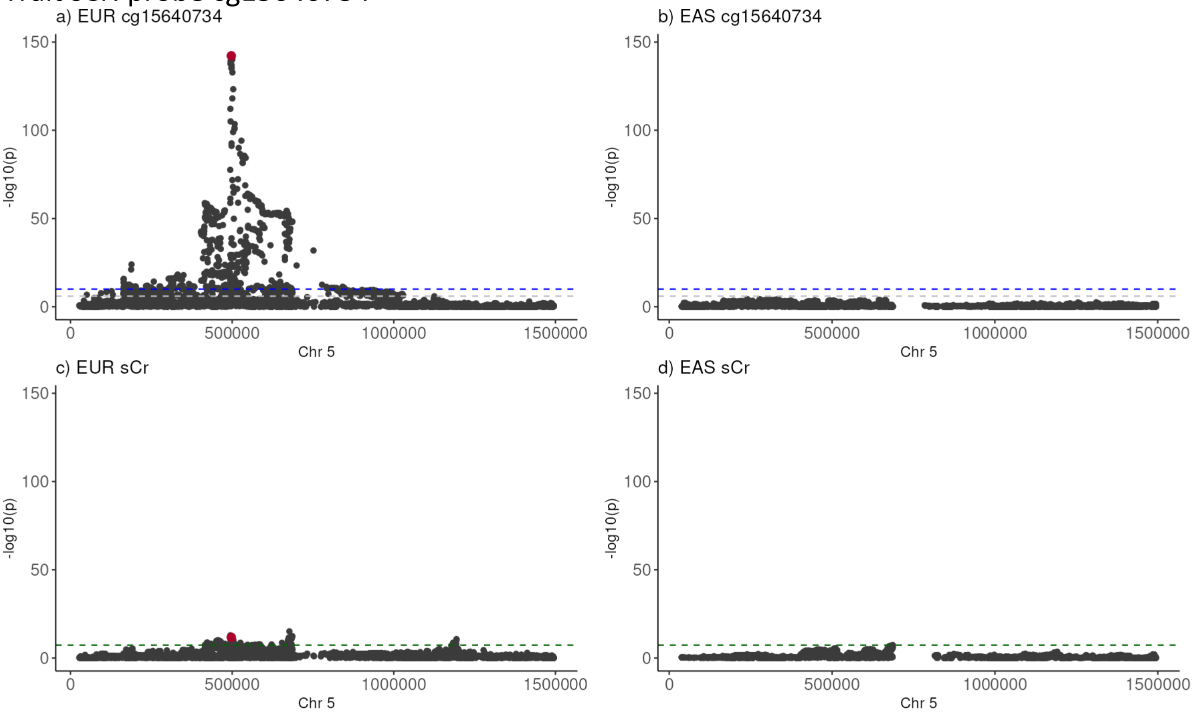

Trait SkC: probe cg25204543

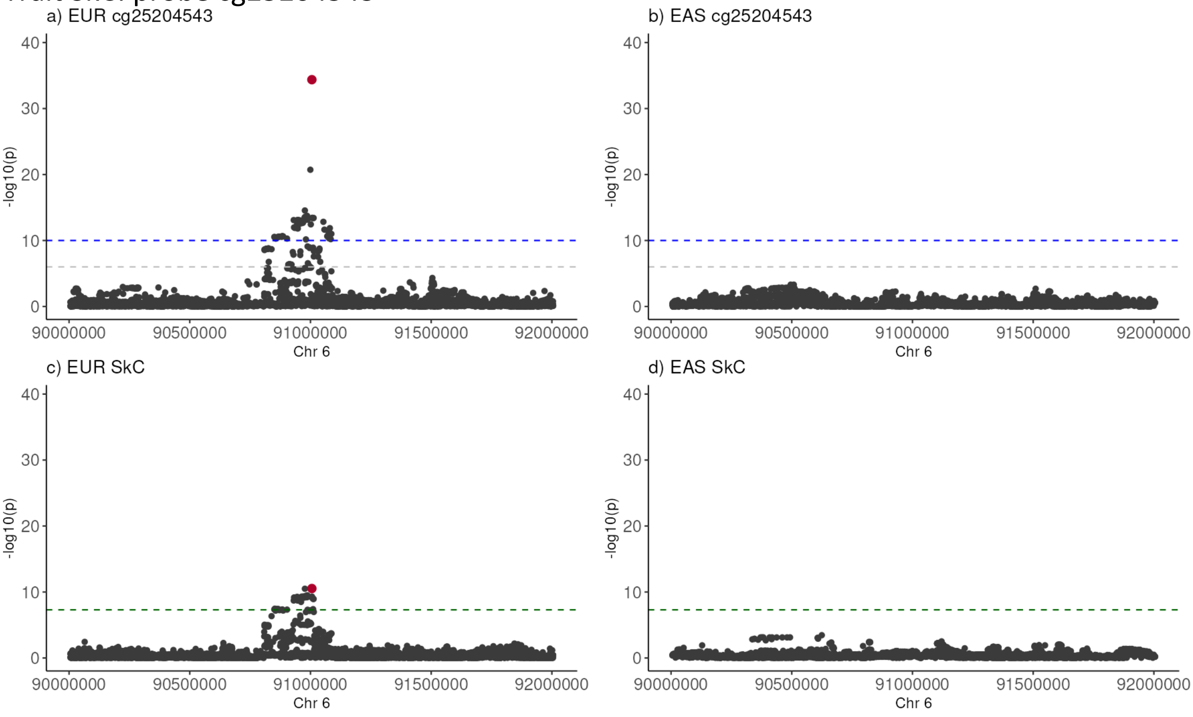

Trait SkC: probe cg03258727

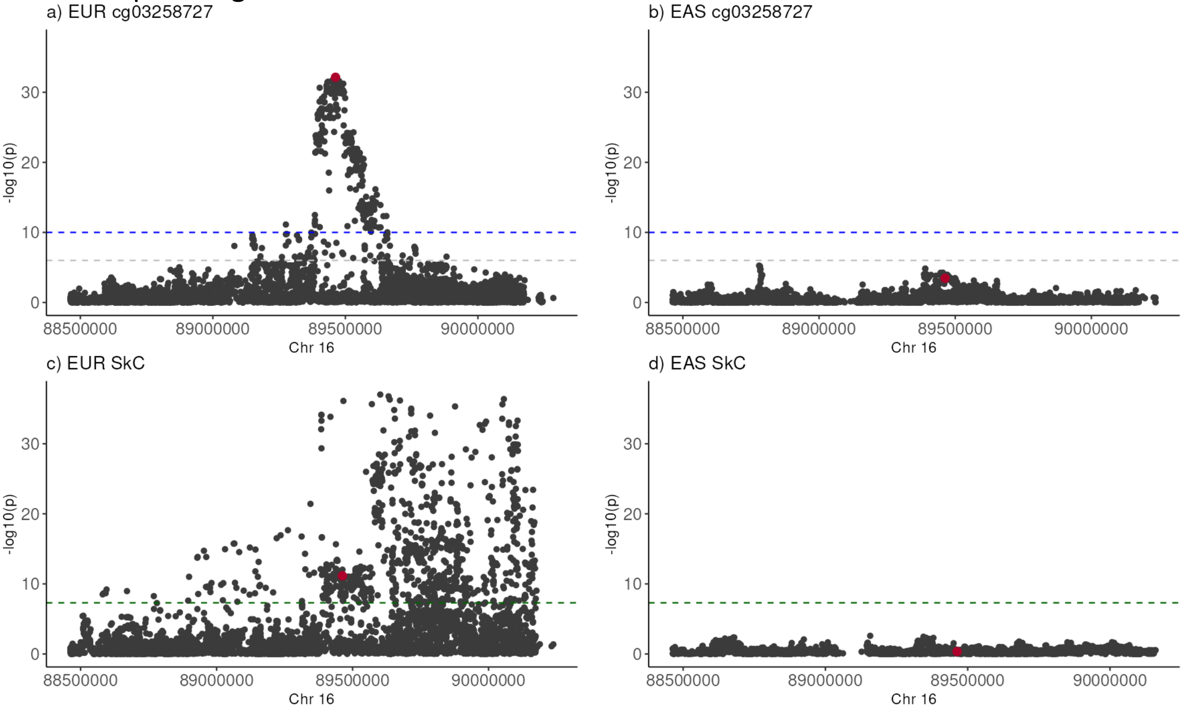

Trait SkC: probe cg09313938

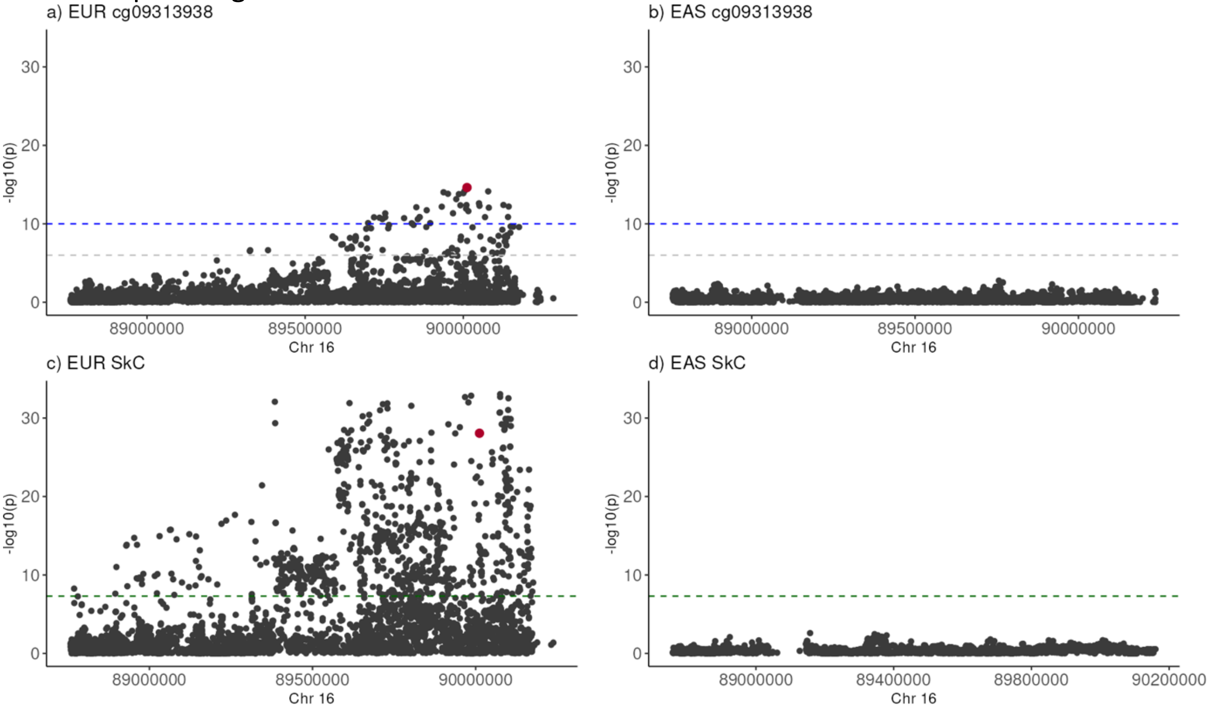

Trait SkC: probe cg23200841

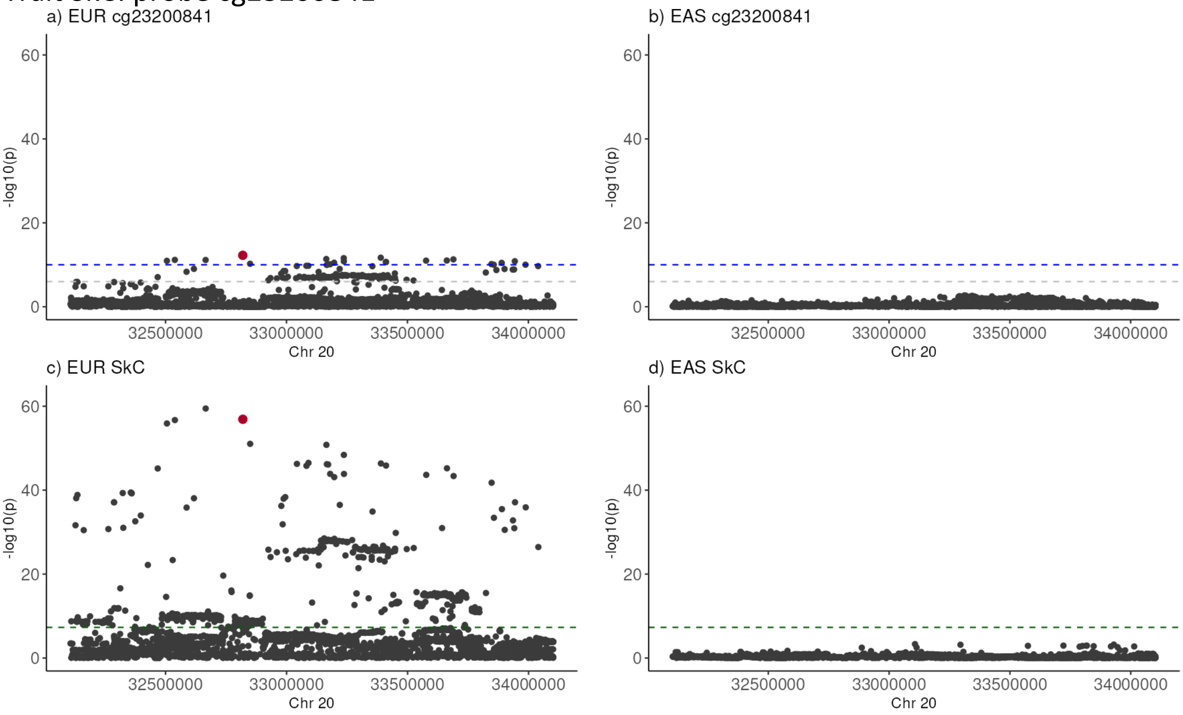

Trait SLE: probe cg04387059

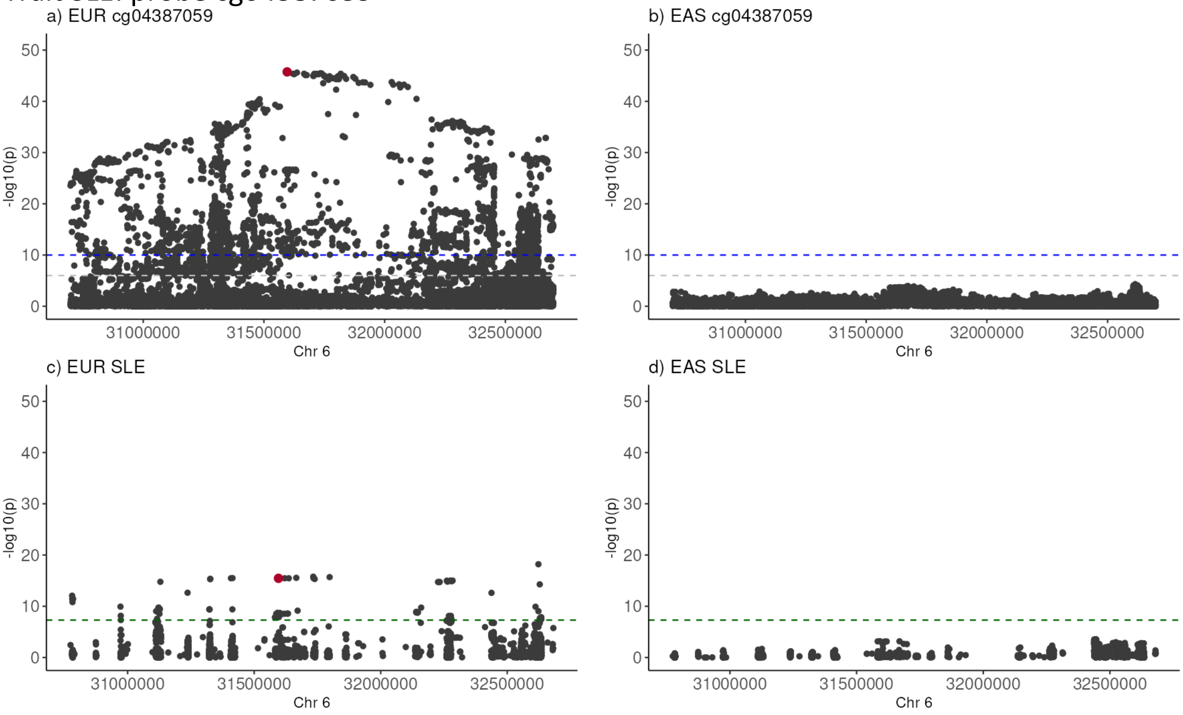

Trait SLE: probe cg12315302

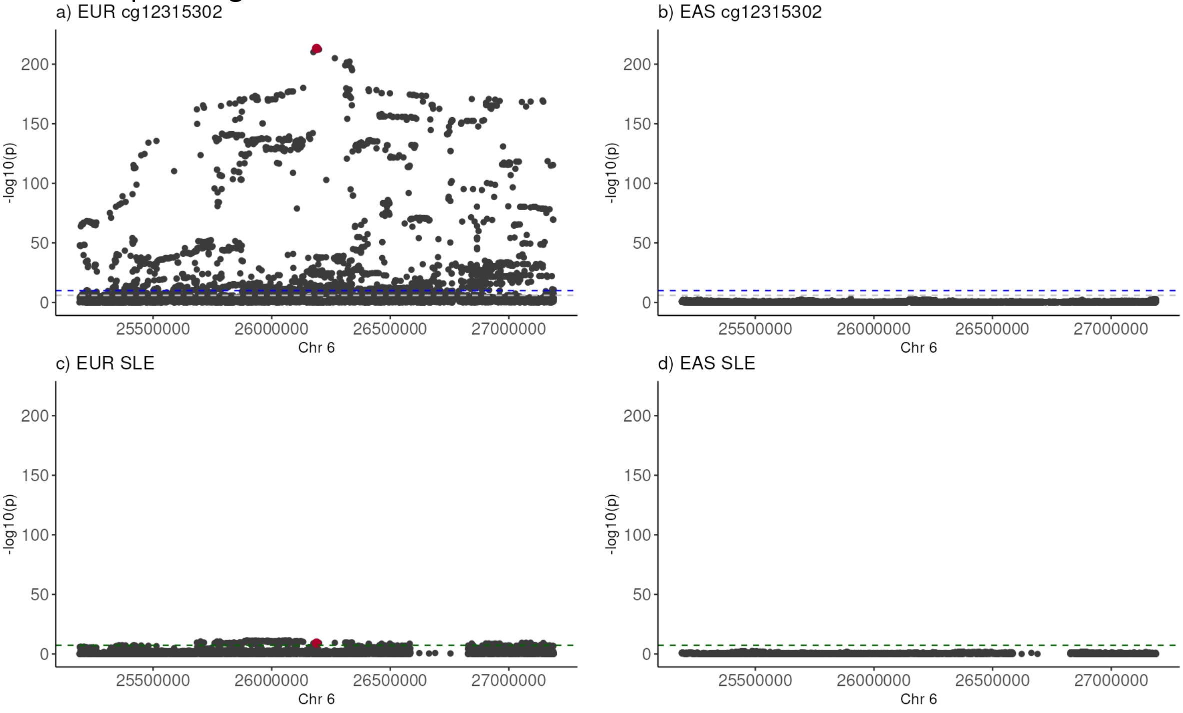

Trait SLE: probe cg12351042

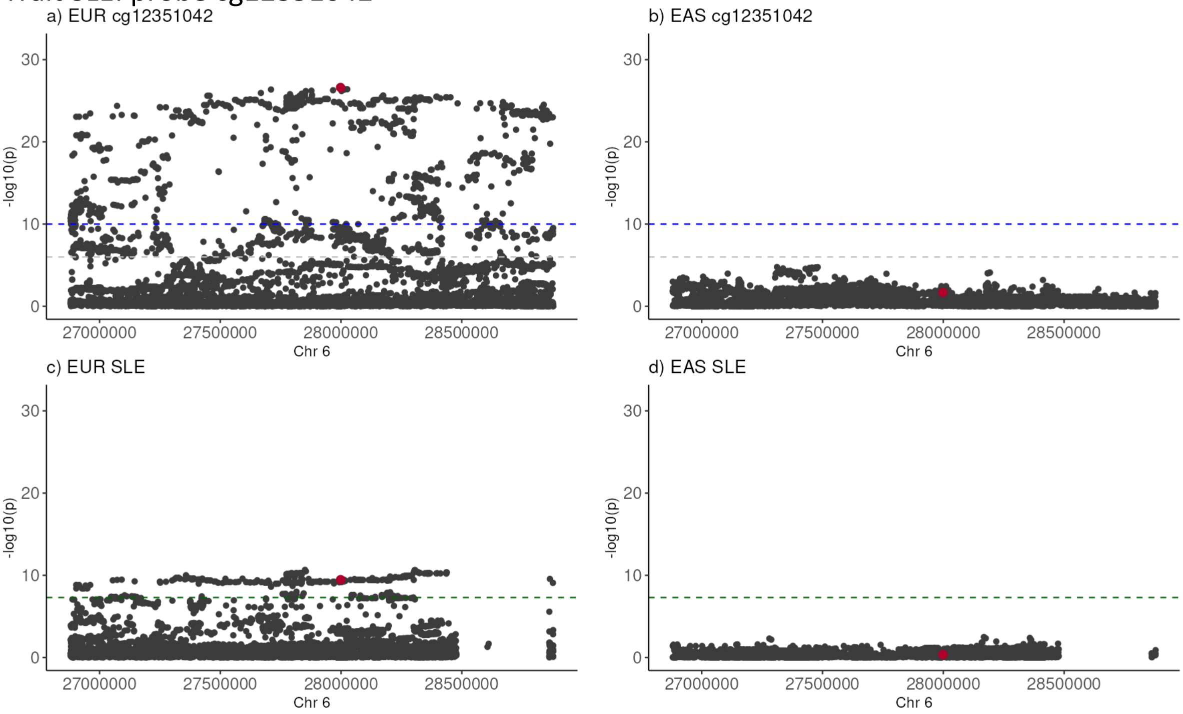

Trait SLE: probe cg19084075

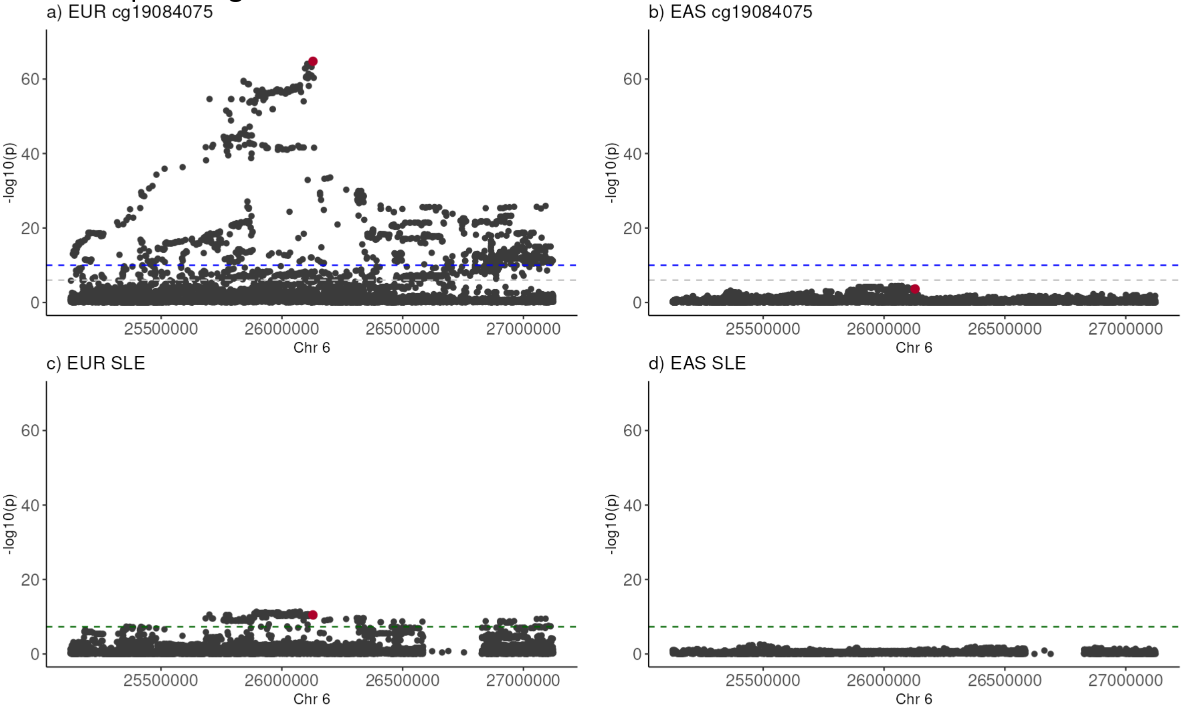

Trait SS: probe cg04387059

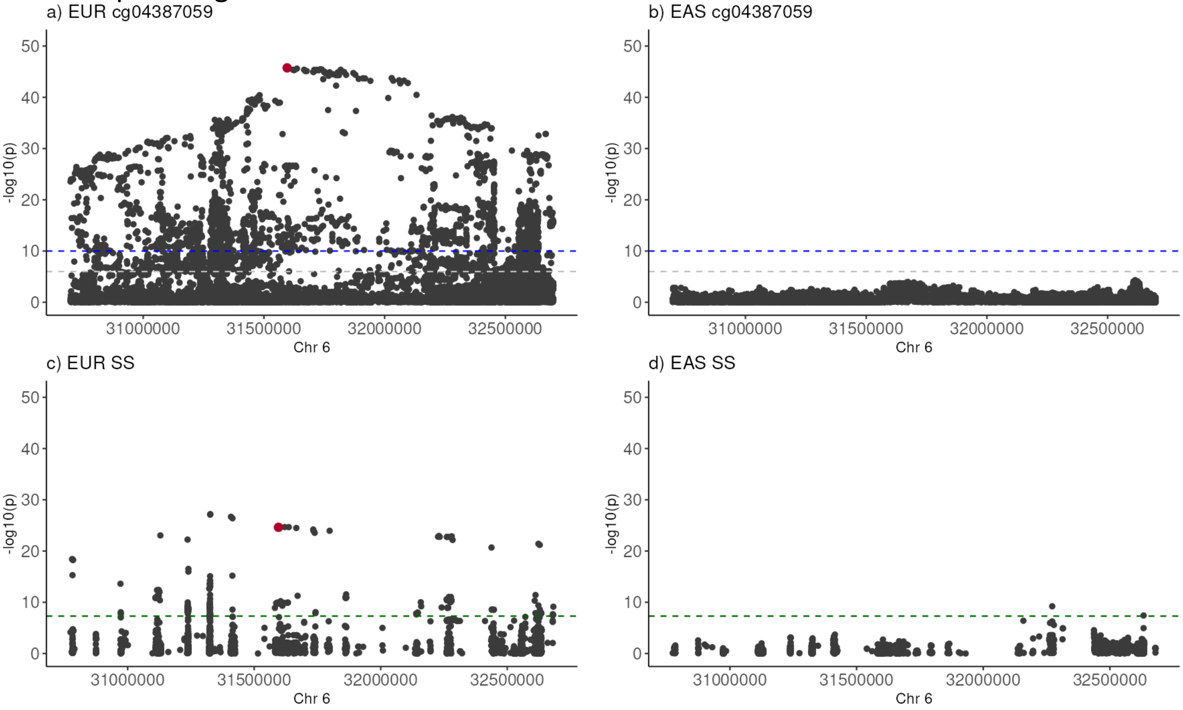

Trait T1D: probe cg12351042

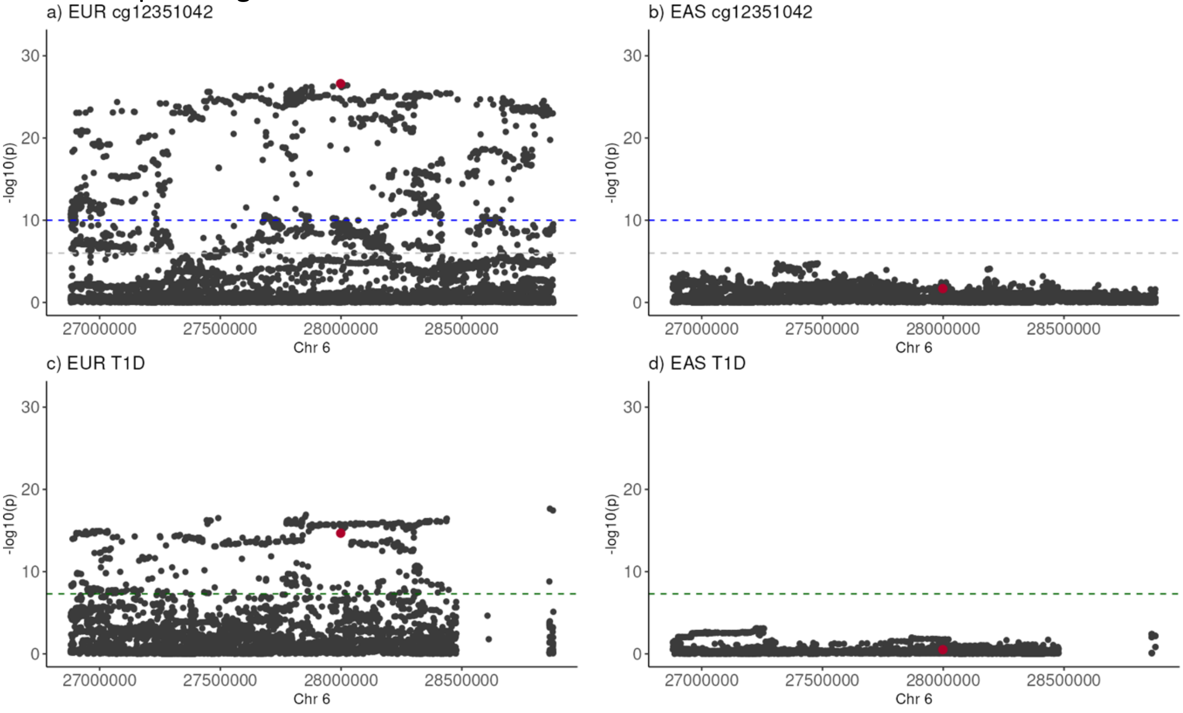

Trait T2D: probe cg21034289

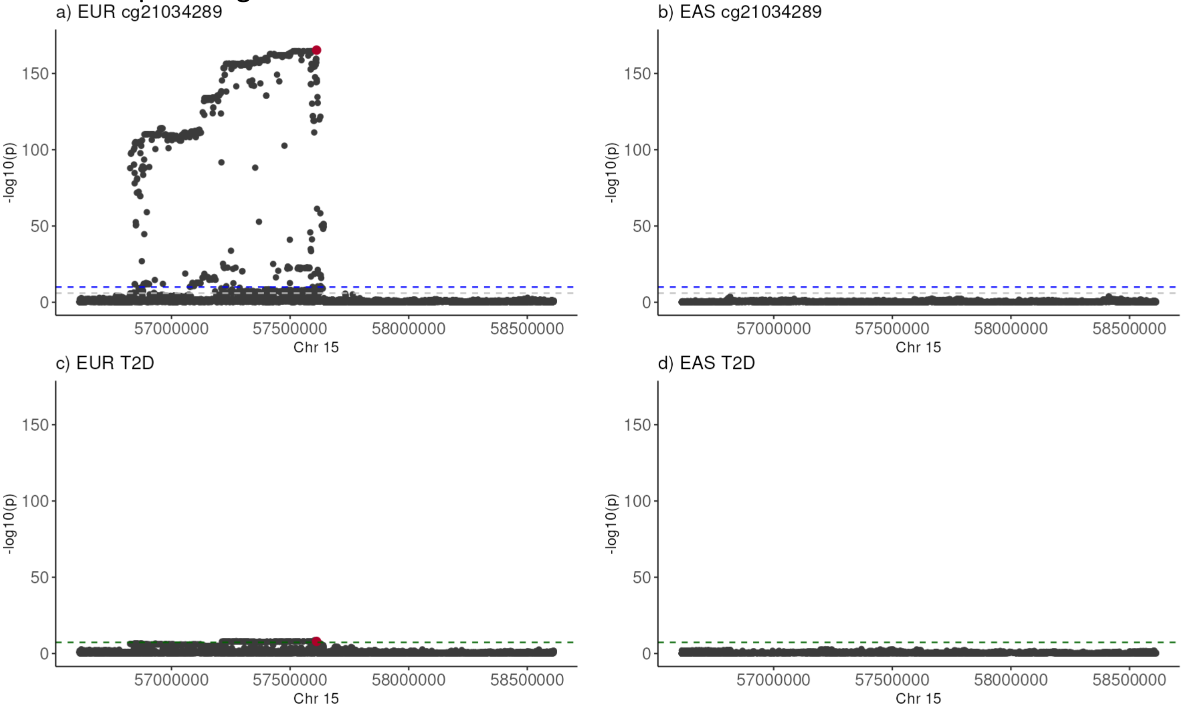

Trait TBil: probe cg15967709

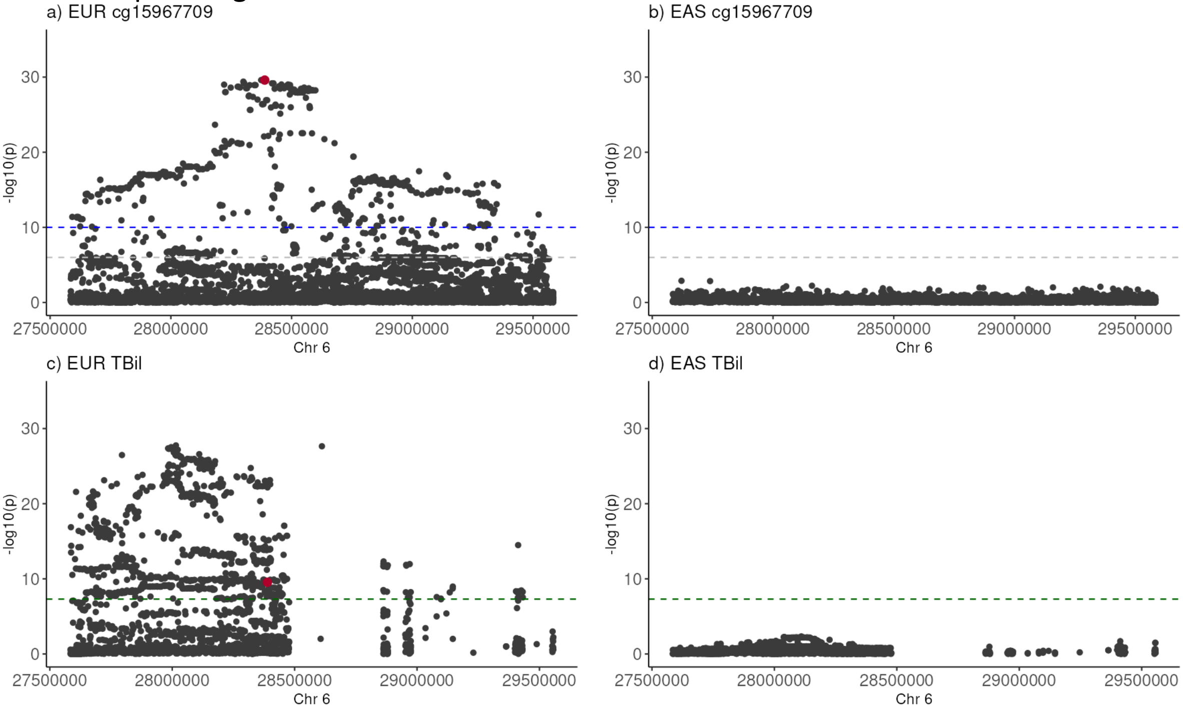

Trait ThC: probe cg11258943

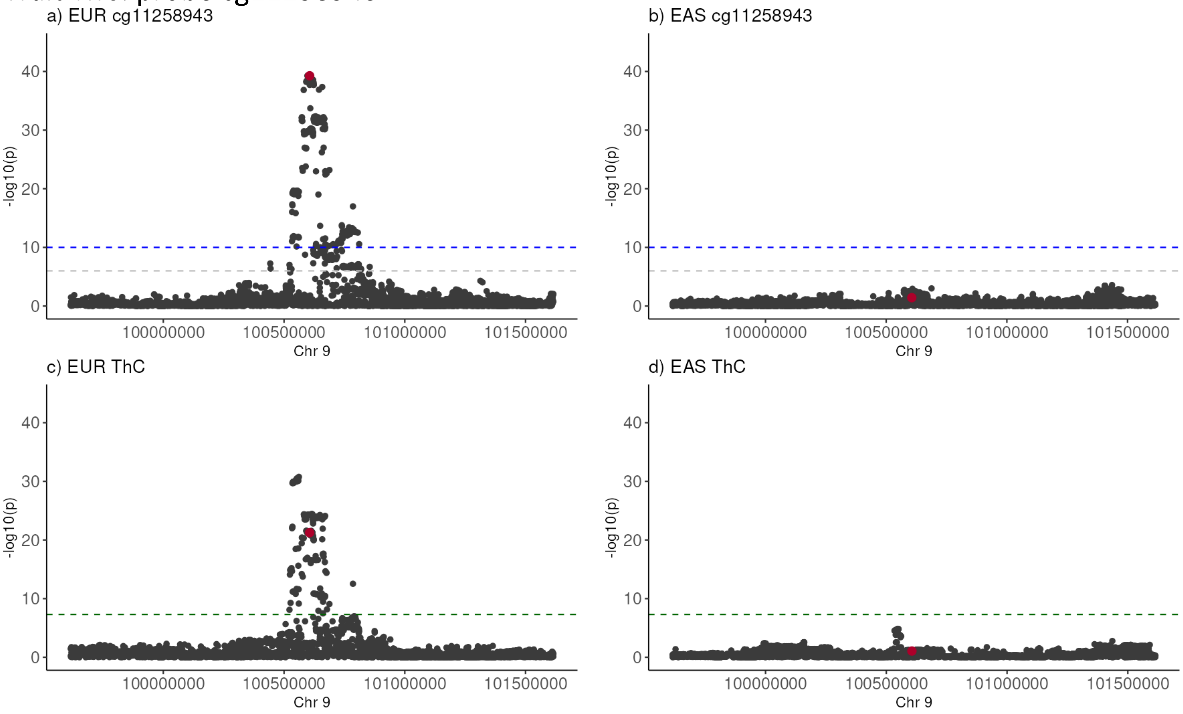

Trait UA: probe cg05790579

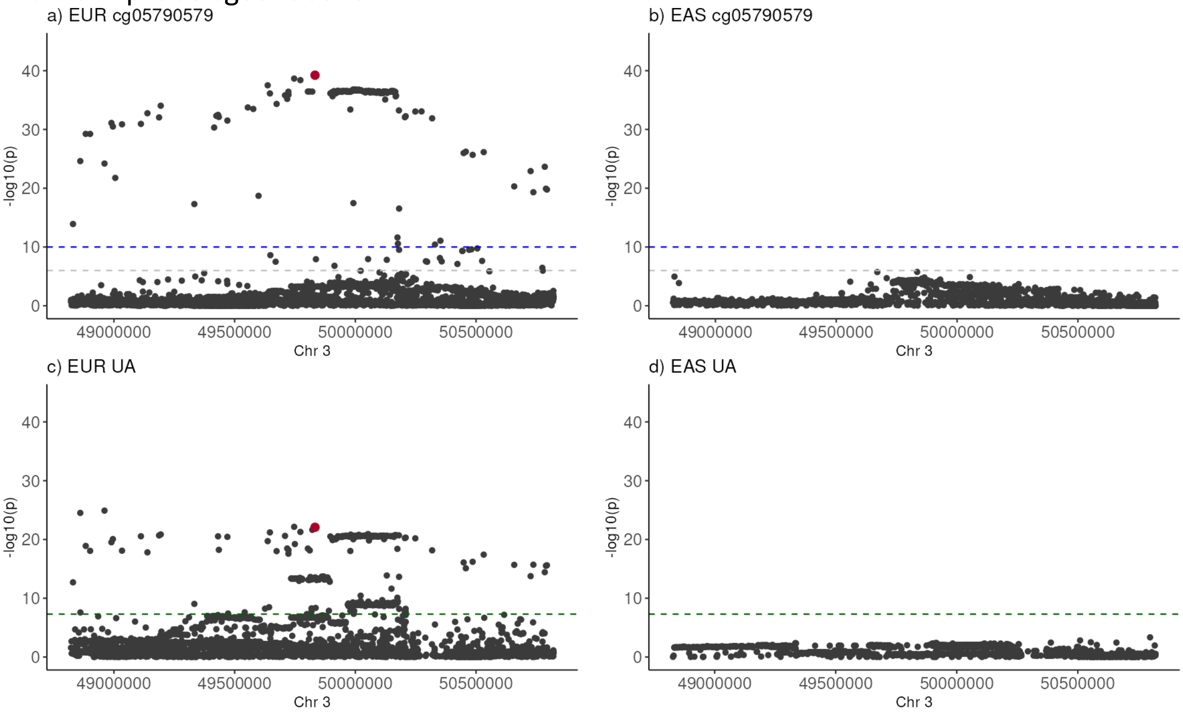

Trait Var: probe cg27220401

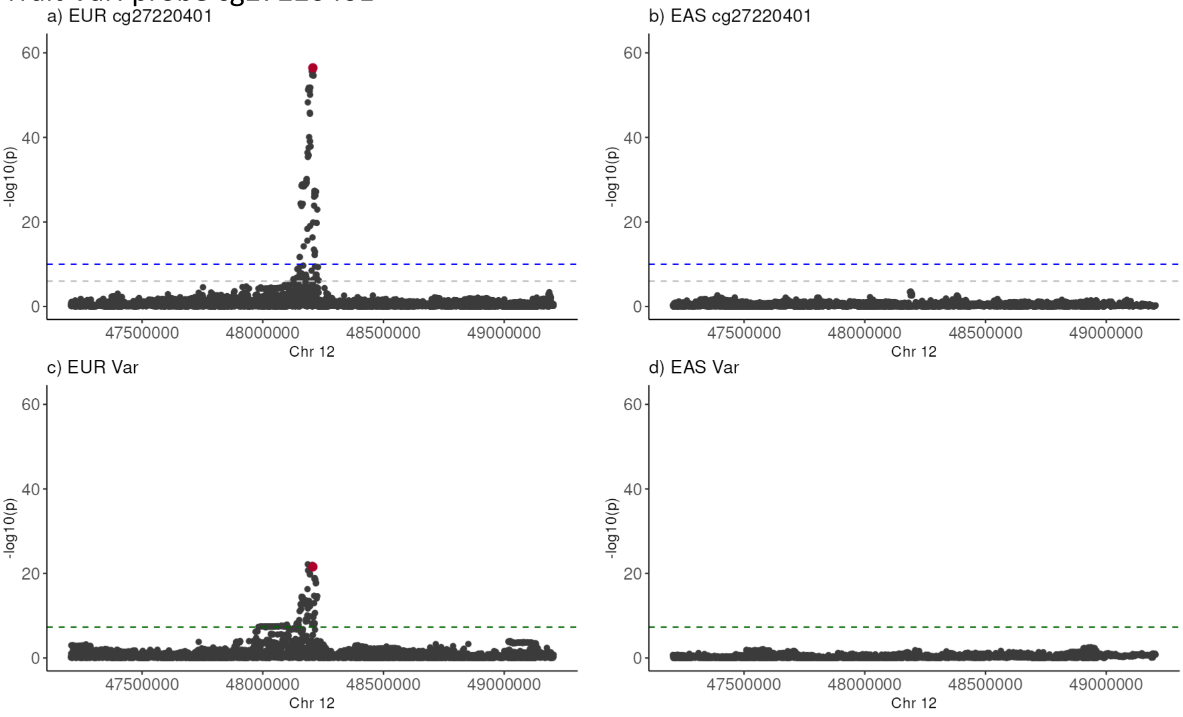

Trait Var: probe cg03699074

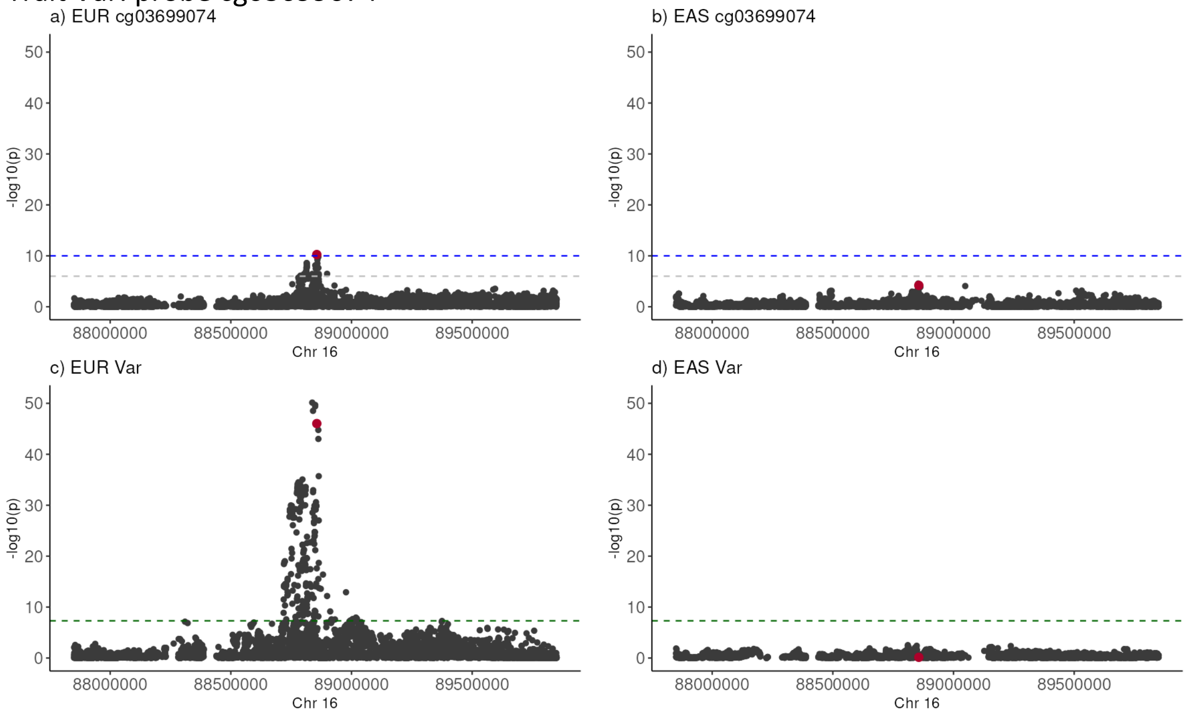

Trait Var: probe cg01656853

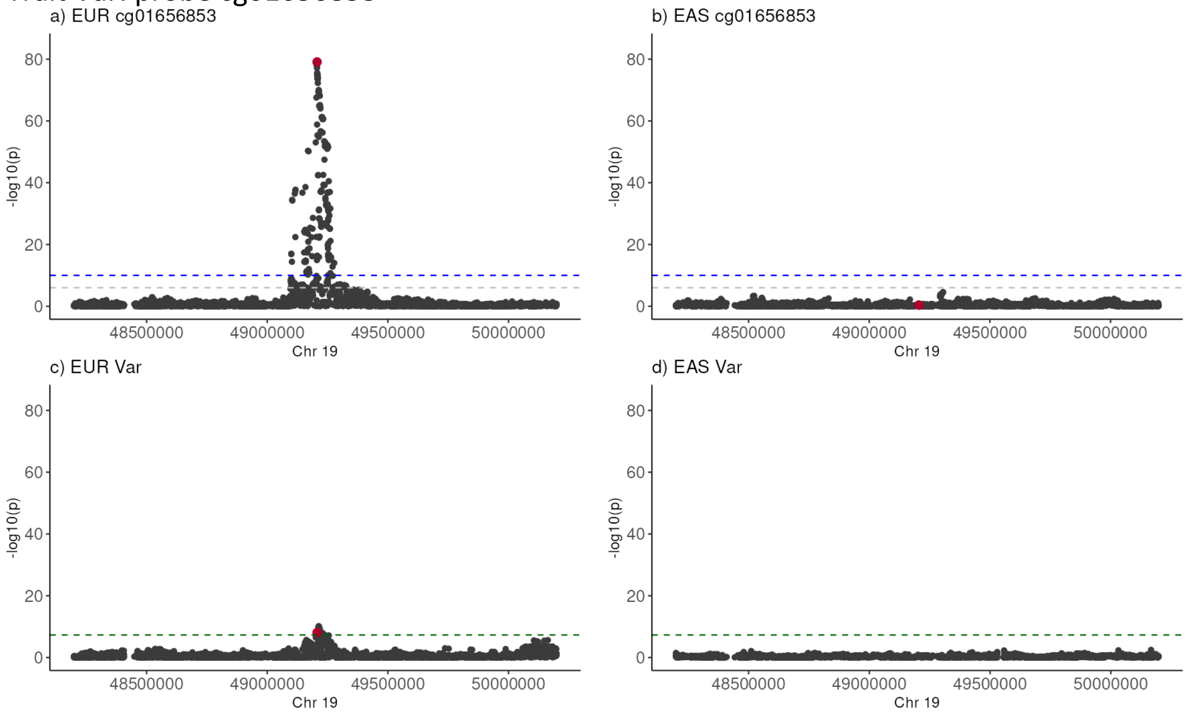

Trait Var: probe cg08873673

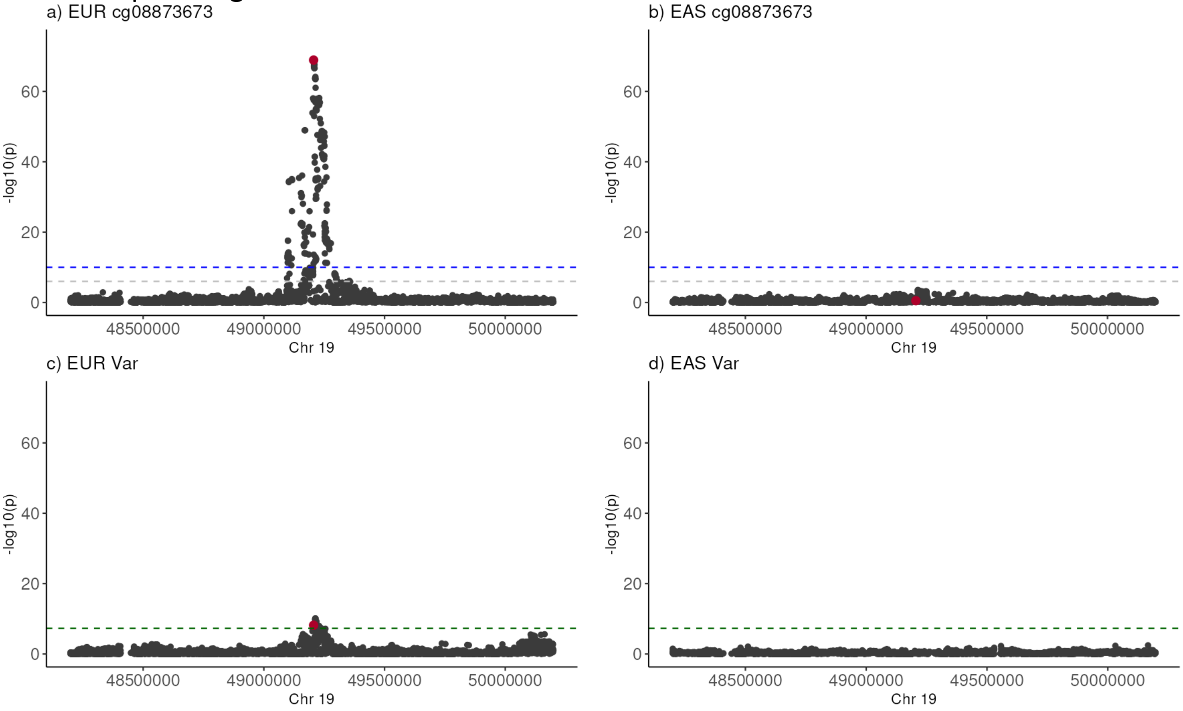

Trait WBC: probe cg06760507

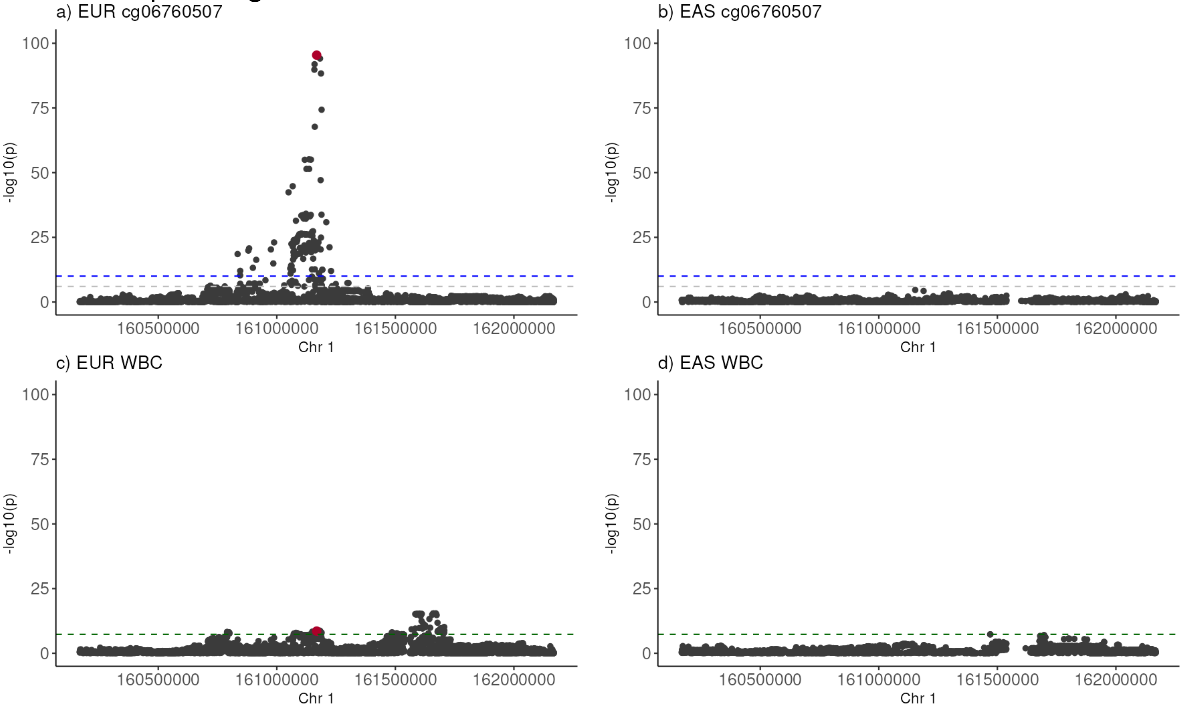

Trait WBC: probe cg11369338

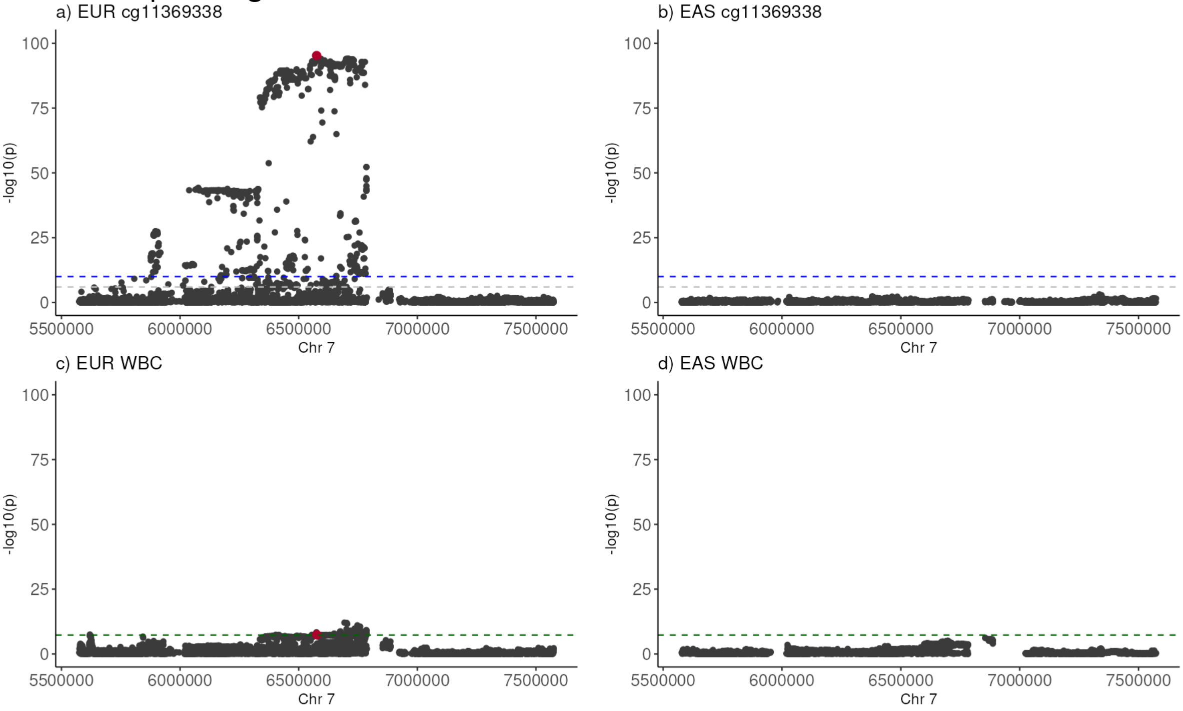

Trait WBC: probe cg07591205

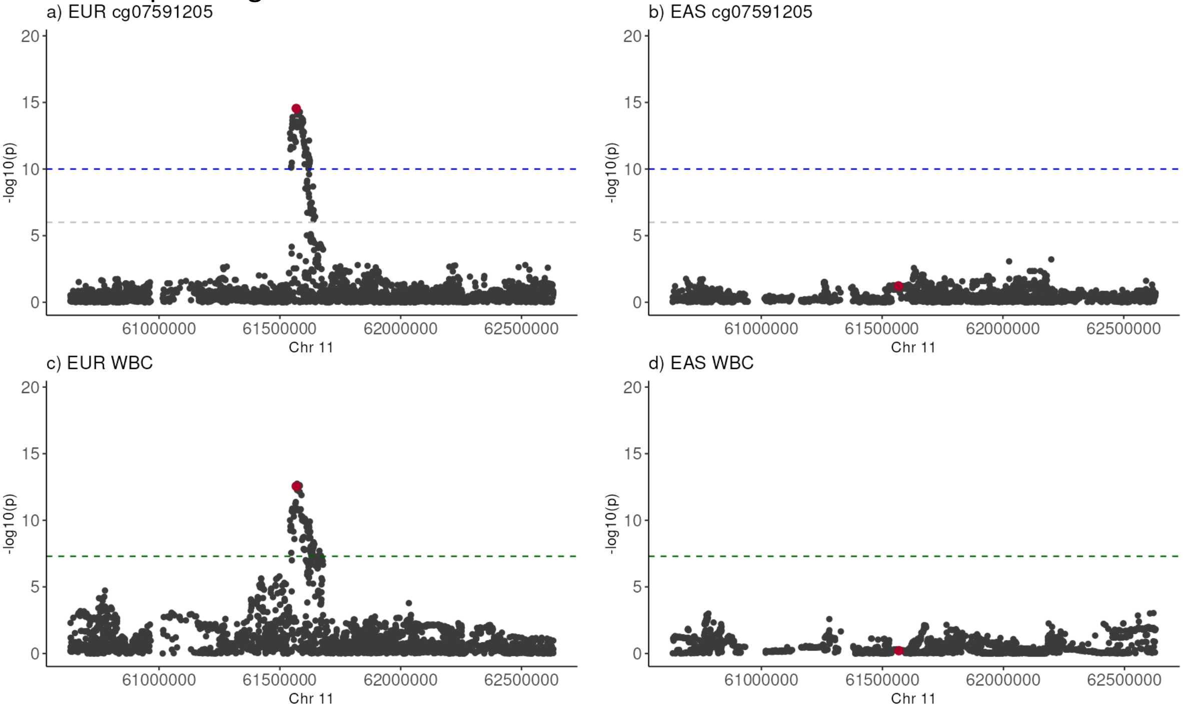

Trait WBC: probe cg19175364

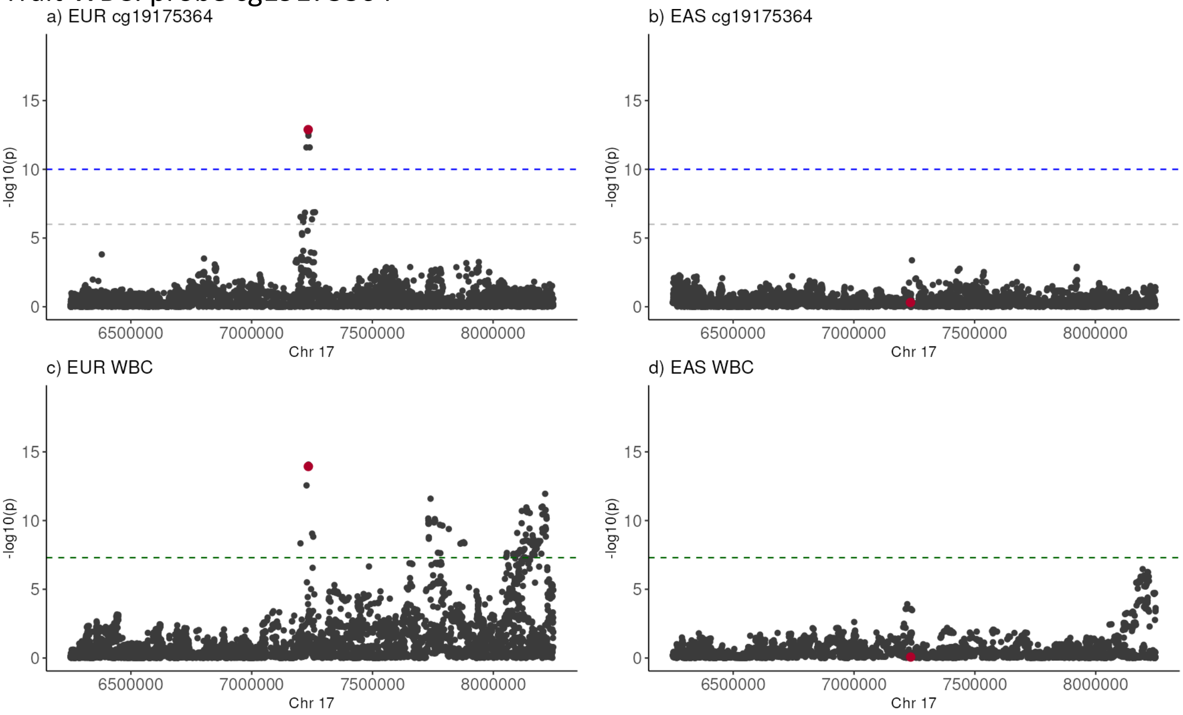

Supplement: Supplementary file 1 — Supplementary Information [file 41467_2024_47005_MOESM1_ESM.pdf]
