# Supplementary material for: Genetic control of DNA methylation is largely shared across European and East Asian populations
Source: Nat Commun. 2024 Mar 28;15:2713. doi: 10.1038/s41467-024-47005-0 (PMC10978881; doi:10.1038/s41467-024-47005-0)
Supplement: Supplementary file 3 — Description of Additional Supplementary Files [file 41467_2024_47005_MOESM3_ESM.pdf]

Description of Additional Supplementary Files

File Name: Supplementary Data 1
Description: **220 traits used to identify pleiotropic associations with ancestry-specific mQTLs.** Listed are the 220 traits reported in Sakaue et al (2021) for which GWAS data was provided in both EUR and EAS ancestries from the UK Biobank and FinnGen, and Biobank Japan, respectively.

File Name: Supplementary Data 2
Description: **SMR associations for ancestry-specific mQTLs across 220 traits.** Ancestry-specific mQTL is defined as DNAm probe where the lead SNP reached the stringent significance threshold of $p<10^{-10}$ in the given ancestry and was unable to reach the replication threshold of $p<10^{-6}$ in the other. SMR was performed at lead SNPs for 28,925 ancestry-specific mQTLs, of which 21,084 were EUR-mQTLs and 7,841 EAS-mQTLs.

Traits abbreviations are listed in Supplemental Data 1.
