# Supplementary material for: Genetic control of DNA methylation is largely shared across European and East Asian populations
Source: Nat Commun. 2024 Mar 28;15:2713. doi: 10.1038/s41467-024-47005-0 (PMC10978881; doi:10.1038/s41467-024-47005-0)
Supplement: Supplementary file 5 — Reporting Summary [file 41467_2024_47005_MOESM5_ESM.pdf]

nature portfolio

Corresponding author(s): Alesha hatton

Last updated by author(s): 2024/01/18

Reporting Summary

Nature Portfolio wishes to improve the reproducibility of the work that we publish. This form provides structure for consistency and transparency in reporting. For further information on Nature Portfolio policies, see our Editorial Policies and the Editorial Policy Checklist.

Statistics

For all statistical analyses, confirm that the following items are present in the figure legend, table legend, main text, or Methods section.

n/a	Confirmed	
☐	☒	The exact sample size (n) for each experimental group/condition, given as a discrete number and unit of measurement
☒	☐	A statement on whether measurements were taken from distinct samples or whether the same sample was measured repeatedly
☐	☒	The statistical test(s) used AND whether they are one- or two-sided<br>*Only common tests should be described solely by name; describe more complex techniques in the Methods section.*
☐	☒	A description of all covariates tested
☐	☒	A description of any assumptions or corrections, such as tests of normality and adjustment for multiple comparisons
☐	☒	A full description of the statistical parameters including central tendency (e.g. means) or other basic estimates (e.g. regression coefficient) AND variation (e.g. standard deviation) or associated estimates of uncertainty (e.g. confidence intervals)
☐	☒	For null hypothesis testing, the test statistic (e.g. F, t, r) with confidence intervals, effect sizes, degrees of freedom and P value noted<br>*Give P values as exact values whenever suitable.*
☒	☐	For Bayesian analysis, information on the choice of priors and Markov chain Monte Carlo settings
☒	☐	For hierarchical and complex designs, identification of the appropriate level for tests and full reporting of outcomes
☐	☒	Estimates of effect sizes (e.g. Cohen's d, Pearson's r), indicating how they were calculated

Our web collection on statistics for biologists contains articles on many of the points above.

Software and code

Policy information about availability of computer code

Data collection	No software were used for data collection
Data analysis	Data analysis was performed as described in the Methods section of the manuscript. Quality control of genetic data was performed using PLINK (v2.0) and the GCTA (v1.93.2beta) software. Phasing was performed using EAGLE2 (v2.0.5)+PBWT and imputation using the Sanger Imputation Service. Quality control of DNA methylation data was performed using the R package meffil.<br>Association analyses were performed using PLINK (v2.0) and the GCTA (v1.93.2beta) software.<br>Meta analysis was performed using the OSCA (v0.46) software and conditional and joint analysis using the GCTA (v1.93.2beta) software. Fine-mapping was performed using SuSiE and SuSiEx. Simulations were performed using PLINK(v2.0) and GCTA (v1.93.2beta) and SMR (v1.03) was performed using the SMR software. The web links for this software is available in the code availability statement. All other analysis was performed using R version 4.0.0.

For manuscripts utilizing custom algorithms or software that are central to the research but not yet described in published literature, software must be made available to editors and reviewers. We strongly encourage code deposition in a community repository (e.g. GitHub). See the Nature Portfolio guidelines for submitting code & software for further information.

Data

Policy information about availability of data

All manuscripts must include a data availability statement. This statement should provide the following information, where applicable:

- Accession codes, unique identifiers, or web links for publicly available datasets
- A description of any restrictions on data availability
- For clinical datasets or third party data, please ensure that the statement adheres to our policy

The mQTL summary data from the meta-analysis of samples of each European (n=3,071) and East Asian (n=2,099) ancestry generated in this study are available at https://yanglab.westlake.edu.cn/software/smr/#mQTLsummarydata. These results have been provided in SMR BESD format (see https://yanglab.westlake.edu.cn/software/smr/#BESDformat).
Access to individual level data for each of the cohorts is as follows: The SGPD DNAm data are available from the Gene Expression Omnibus (GEO) under accession code GSE145361. The LBC DNAm data is available at the European Genome-phenome Archive under accession number EGAS00001000910. DNAm data for the BSGS is available from the Gene Expression Omnibus under accession code GSE56105. Deposit of CHNMND DNAm data in a repository does not comply with the consent process and ethics approval, but sharing data is possible by emailing the corresponding author of the cohort publication. Primary data of the Tibetan and Han Chinese subjects are available through application at https://www.wmubiobank.org. Analysis of the UK Biobank resource was conducted under the application number 12505. The genotype and phenotype data are available upon application to the UKB [http://www.ukbiobank.ac.uk/]. Health and Retirement Study data was accessed from dbGaP (accessions: phs000428).
The web links for the publicly available datasets used in the study are as follows: The 1000 Genomes Phase 3 data available at https://ftp.1000genomes.ebi.ac.uk/vol1/ftp/phase3/; mQTL data from Hawe at al 2022 [31]: https://zenodo.org/record/5196216#.YRZ3TfJxeUk; mQTL data from Min et al 2021 [83] data: http://mqtldb.godmc.org.uk; GWAS summary statistics for 220 traits used for SMR from Sakaue et al 2021 [41]: https://pheweb.jp/downloads; Annotation of Infinium DNA Methylation BeadChip probes: https://zwdzwd.github.io/InfiniumAnnotation

Research involving human participants, their data, or biological material

Policy information about studies with human participants or human data. See also policy information about sex, gender (identity/presentation), and sexual orientation and race, ethnicity and racism.

Reporting on sex and gender	Sex was used as a covariate for mQTL analysis. Sex was based on self-reported data and was confirmed using genetically determined sex.
Reporting on race, ethnicity, or other socially relevant groupings	In order to classify cohorts into genetically similar groups we inferred ancestry based on principle component analysis using genetic data. We note that genetically determined ancestry may not reflect an individual's self-reported ethnicity and that we are examining a limited spectrum of the ancestral diversity.
Population characteristics	The SGPD cohort includes PD cases and age-matched controls recruited from three different studies across Australia and New Zealand (n=1659). Participants in this cohort had a mean age of 67 years (range 11-96) of which 728 were female (43.9%). The Lothian Birth Cohorts of 1921 and 1936 are are follow-up studies of the Scottish Mental Health Surveys of 1932 and 1947 (n=1437). Participants in this cohort had a mean age of 72.8 years (range 68-81) of which 763 were female (50.1%). The Brisbane Systems Genomics Study is a family-based study, consisting of adolescent monozygotic and dizygotic twins, their siblings and parents (n=614 from 177 families). Participants in this cohort had a mean age of 21.4 years (range 10-75) of which 295 were female (48.0%). The Chinese Motor Neuron Disease Cohort is an Amyotrophic Lateral Sclerosis (ALS) case-control cohort (n=651). Participants in this cohort had a mean age of 47.2 years (range 17-76) of which 273 were female (41.9%). The Tibetan-Han Chinese high-altitude is a study on high-altitude adaptation comprised of three groups of EAS ancestry recruited from two sites in the Tibetan Plateau and Wenzhou (n=1448). Participants in this cohort had a mean age of 42 years (range 11-90) of which 1090 were female (75.0%) .<br>The full release of the UKB data consisted of genotype and phenotype data for ~500,000 participants across the United Kingdom. This study utilised a random subset of 9,800 individuals with a mean age of 56.7 years (range 40-70) of which 5504 were female (56.2%).
Recruitment	Our study did not directly involve the recruitment of participants. This data had been collected previously for other purposes.
Ethics oversight	*Identify the organization(s) that approved the study protocol.*

Note that full information on the approval of the study protocol must also be provided in the manuscript.

Field-specific reporting

Please select the one below that is the best fit for your research. If you are not sure, read the appropriate sections before making your selection.

☒ Life sciences ☐ Behavioural & social sciences ☐ Ecological, evolutionary & environmental sciences

For a reference copy of the document with all sections, see nature.com/documents/nr-reporting-summary-flat.pdf

Life sciences study design

All studies must disclose on these points even when the disclosure is negative.

Sample size	We performed mQTL analysis on 3,701 individuals of European ancestry and 2,099 of East Asian ancestry based on existing DNA methylation data that was available to us (of which had been collected previously for other purposes).
Data exclusions	Samples were excluded based on genotype and DNAm quality control and genetically determined ancestry as described in the methods section.
Replication	We performed analysis on three independent cohorts with genetically determined European ancestry and two independent cohorts with genetically determined East Asian ancestry. We performed analyses separably in all cohorts following a uniformed protocol and meta-analysed the studies identified to be of the same genetically determined ancestry. We assessed replication of shared mQTLs using mQTL data from Hawe at al. (2022). Of the 65,522 of the DNAm probes that were in common with our analysis set, 46,332 (70.7% of the DNAm probes) were also significant in both ancestries in our study.
Randomization	Not applicable, this is not an experimental (i.e. interventional) study.
Blinding	Not applicable, this is not an experimental (i.e. interventional) study.

Reporting for specific materials, systems and methods

We require information from authors about some types of materials, experimental systems and methods used in many studies. Here, indicate whether each material, system or method listed is relevant to your study. If you are not sure if a list item applies to your research, read the appropriate section before selecting a response.

Materials & experimental systems

n/a	Involved in the study	
☒	☐	Antibodies
☒	☐	Eukaryotic cell lines
☒	☐	Palaeontology and archaeology
☒	☐	Animals and other organisms
☒	☐	Clinical data
☒	☐	Dual use research of concern
☒	☐	Plants

Methods

n/a	Involved in the study	
☒	☐	ChIP-seq
☒	☐	Flow cytometry
☒	☐	MRI-based neuroimaging

Plants

Seed stocks	*Report on the source of all seed stocks or other plant material used. If applicable, state the seed stock centre and catalogue number. If plant specimens were collected from the field, describe the collection location, date and sampling procedures.*
Novel plant genotypes	*Describe the methods by which all novel plant genotypes were produced. This includes those generated by transgenic approaches, gene editing, chemical/radiation-based mutagenesis and hybridization. For transgenic lines, describe the transformation method, the number of independent lines analyzed and the generation upon which experiments were performed. For gene-edited lines, describe the editor used, the endogenous sequence targeted for editing, the targeting guide RNA sequence (if applicable) and how the editor was applied.*
Authentication	*Describe any authentication procedures for each seed stock used or novel genotype generated. Describe any experiments used to assess the effect of a mutation and, where applicable, how potential secondary effects (e.g. second site T-DNA insertions, mosiacism, off-target gene editing) were examined.*
